# Prognostic and predictive value of super-enhancer-derived signatures for survival and lung metastasis in osteosarcoma

**DOI:** 10.1186/s12967-024-04902-8

**Published:** 2024-01-22

**Authors:** Guanyu Huang, Xuelin Zhang, Yu Xu, Shuo Chen, Qinghua Cao, Weihai Liu, Yiwei Fu, Qiang Jia, Jingnan Shen, Junqiang Yin, Jiajun Zhang

**Affiliations:** 1 Department of Musculoskeletal Oncology, The First Affiliated Hospital of Sun Yat-Sen University, Guangzhou, 510080 China; 2 Department of Orthopedics, Jishuitan Hospital of Beijing, Beijing, China; 3 Department of Pathology, The First Affiliated Hospital of Sun Yat-Sen University, Guangzhou, China; 4 Guangzhou City Polytechnic, Guangzhou, China; 5 Guangdong Provincial Key Laboratory of Orthopedics and Traumatology, The First Affiliated Hospital of Sun Yat-Sen University, Guangzhou, China

**Keywords:** Osteosarcoma, Super-enhancer, Prognostic signature, Survival, Lung metastasis

## Abstract

**Background:**

Risk stratification and personalized care are crucial in managing osteosarcoma due to its complexity and heterogeneity. However, current prognostic prediction using clinical variables has limited accuracy. Thus, this study aimed to explore potential molecular biomarkers to improve prognostic assessment.

**Methods:**

High-throughput inhibitor screening of 150 compounds with broad targeting properties was performed and indicated a direction towards super-enhancers (SEs). Bulk RNA-seq, scRNA-seq, and immunohistochemistry (IHC) were used to investigate SE-associated gene expression profiles in osteosarcoma cells and patient tissue specimens. Data of 212 osteosarcoma patients who received standard treatment were collected and randomized into training and validation groups for retrospective analysis. Prognostic signatures and nomograms for overall survival (OS) and lung metastasis-free survival (LMFS) were developed using Cox regression analyses. The discriminatory power, calibration, and clinical value of nomograms were evaluated.

**Results:**

High-throughput inhibitor screening showed that SEs significantly contribute to the oncogenic transcriptional output in osteosarcoma. Based on this finding, focus was given to 10 SE-associated genes with distinct characteristics and potential oncogenic function. With multi-omics approaches, the hyperexpression of these genes was observed in tumor cell subclusters of patient specimens, which were consistently correlated with poor outcomes and rapid metastasis, and the majority of these identified SE-associated genes were confirmed as independent risk factors for poor outcomes. Two molecular signatures were then developed to predict survival and occurrence of lung metastasis: the SE-derived OS-signature (comprising *LACTB*, *CEP55*, *SRSF3*, *TCF7L2*, and *FOXP1*) and the SE-derived LMFS-signature (comprising *SRSF3*, *TCF7L2*, *FOXP1*, and *APOLD1*). Both signatures significantly improved prognostic accuracy beyond conventional clinical factors.

**Conclusions:**

Oncogenic transcription driven by SEs exhibit strong associations with osteosarcoma outcomes. The SE-derived signatures developed in this study hold promise as prognostic biomarkers for predicting OS and LMFS in patients undergoing standard treatments. Integrative prognostic models that combine conventional clinical factors with these SE-derived signatures demonstrate substantially improved accuracy, and have the potential to facilitate patient counseling and individualized management.

**Supplementary Information:**

The online version contains supplementary material available at 10.1186/s12967-024-04902-8.

## Background

Osteosarcoma is the most common primary bone malignancy in children and adolescents worldwide [1]. Rapid tumor progression and early lung metastasis are the leading causes of treatment failure and death in osteosarcoma patients [2–4]. Individualized treatment regimens including chemotherapy regimens, surgery type, resection margins, radiotherapy, etc. should be administered according to the prognostic stratification to improve the efficacy of clinical interventions [5, 6]. With the deepening understanding of clinical medicine and cytology, novel clinical variables (such as microbial infections, application of therapeutic antibiotics, etc.) and molecular markers have been discovered are intricately linked to cancer progression, and effectively contribute to the outcome prediction [7]. Due to the rapid progression and heterogeneity of osteosarcoma, conventional clinical prognostic variables, such as histological type, tumor site, Enneking staging, tumor size, and alkaline phosphatase (ALP), are not effective enough for predicting the survival and lung metastasis of osteosarcoma patients [8, 9]. Thus there is a need to increase the prognostic and predictive value of staging systems, and this may be achieved with validated molecular biomarkers [10, 11].

Clinical parameters and potential molecular predictors, such as genomic and transcriptional alterations, have been investigated to stratify patients into different prognostic groups, but all still require validation and none have entered clinical practice [9, 12–14]. The rarity of the disease considerably limits the overall sample size and the collection of biometric data for the construction of prognostic model.

Super-enhancers (SEs) are large groups of cis-regulatory DNA elements that play vital roles in defining cell identity and fate [15, 16]. In various types of cancers, SEs control the expression of prominent tumor-promoting genes such as MYC [17], TAL1 [18], STAT3 [19] and EGFR [20], and mediate transcription dysregulation of cancer cells. Our previous work identified a catalog of SEs in osteosarcoma, and found that the genes associated with these DNA elements were tissue-specific and significantly involved in the malignancy of osteosarcoma [21]. Based on lineage-specific characteristics, SE-associated genes are considered potential diagnostic and prognostic biomarkers for cancer patients [16]. Tsang et al. revealed that the cis-acting SE landscape undergoes extensive reprogramming during liver carcinogenesis. Hepatocellular carcinoma (HCC) cells acquired SEs in multiple prominent oncogenes, promoting their vigorous expression [22]. He et al. identified the specific SEs in normal cholangiocyte cells, and found they have a close relation with OS and progression-free survival (PFS) in intrahepatic cholangiocarcinoma patients [23]. However, the clinical significance of genes driven by SEs in osteosarcoma has rarely been reported.

In this study, high-throughput screening of various targets with small molecule inhibitors suggested that SEs play an important role in the progression of osteosarcoma. By integrating and analyzing multiple transcriptomics data of patient specimens from our institution as well as public databases, we found that a set of SE-associated genes in cancer cells, which as the major cellular component in OS specimen, showed particular efficacy in risk stratification and prognostic prediction. As an easy-to-use pathological technique with well clinical application value, immunohistochemistry (IHC) was used to detect the expressions of these genes. We developed 2 nomograms for clinical use that integrated the SE-derived signatures and clinicopathological risk factors to predict survival and lung metastasis of osteosarcoma patients. Subsequently, the prognostic accuracy and clinical benefit were assessed and validated, and compared with that of traditional risk factors.

## Methods and materials

### Human cell lines

Human osteosarcoma cell lines SJSA-1 and U2-OS were obtained from American Type Cell Collection (ATCC). The ZOS-M cell lines, derived from a primary osteosarcoma tumor and metastasis, respectively, have been described previously [24]. U2OS/MTX300 cells, a methotrexate-resistant derivative of the U2-OS human osteosarcoma cell line, were kindly provided by Dr. M. Serra (Instituti Ortopedici Rizzoli, Bologna, Italy). All of the cells used were authenticated before experiments, and were cultured according to instructions from ATCC.

### Patients and clinical database

The present study included scRNA-seq data of 8 tumor specimens of patients diagnosed with OS. Four samples (No-met, Met-quickly) were collected at The First Affiliated Hospital of Guangzhou Medical University, Guangzhou. The patients provided written informed consent, and agreed to donate specimens for the present study. The data of the rest 4 sample were retrieved from public data (GSE152048). Osteosarcoma tissue specimens from 212 patients with complete follow-up data treated at the First Affiliated Hospital of Guangzhou Medical University between March 1, 2003, and December 31, 2018 were retrospectively examined. The inclusion criteria were pathologically confirmed osteosarcoma, and received standard treatment as described previously [25]. After reviewing the medical records and contacting the patients or their relatives by telephone, follow-up information was available up to March 1, 2022. Data collected included sex, age, surgery type, primary tumor site, Enneking stage, histological type, tumor size, blood indices at first visit (e.g., alkaline phosphatase [ALP] and lactate dehydrogenase [LDH]), the presence of distant metastasis, and survival. Because age influences ALP expression, 150 U/L was regarded as the upper serum ALP limit in patients less than 18 years, while 110 U/L was considered the limit in those 18 years and older [26]. Overall survival (OS) was defined as the time from diagnosis to death from any cause, or time to the last follow-up visit. Lung metastasis-free survival (LMFS) was defined as the time from diagnosis to the detection of lung metastasis. All 212 patients were included in the OS analysis, and 188 of the patients without detection of lung metastasis at the first visit were included in the LMFS analysis. Formalin-fixed and paraffin-embedded surgical tumor samples were obtained for immunohistochemistry, osteosarcoma was confirmed prior to our experiments by pathologists from the Clinical Pathology Department of the Hospital. This study was approved by Medical Ethics Committee of the First Affiliated Hospital of Sun Yat-sen University.

### High-throughput small-molecule inhibitor screening

Four osteosarcoma cell lines (termed U2-OS, SJSA-1, ZOSM, and U2-OS/MTX300) were screened for sensitivity against a panel of 150 small-molecule inhibitors. Briefly, cells were seeded in a 384-well format with a seeding density of 800 cells per well and treated with a concentration of 30 μM of individual compounds before evaluating cell viability after 72 h using a CCK-8 assay. Further evaluation of compounds which allowed for more than 80% inhibition of cell viability was performed in a eight-point five-fold dilution series of each compound (including the no-drug control) before evaluating cell viability after 72 h. Mean inhibitory concentration allowing for 50% reduction in cell viability (IC50) was calculated using non-linear regression analysis. Compounds were ranked for potency using mean IC50 values for the four cell lines.

### Microarray sample preparation and analysis

Total RNA was extracted from U2-OS or SJSA-1 cells treated with DMSO (control) or various doses of THZ531, respectively, using TRIzol reagent (Invitrogen, Carlsbad, CA, USA) according to manufacturer’s instructions. An Affymetrix GeneChip® PrimeView™ Human Gene Expression Array was used for the microarray analysis. The hybridization data were analyzed using Affymetrix GeneChip Command Console Software (AGCC). Microarray data was normalized using the Robust Multiarray Average (RMA) method, and expression values were calculated with the Affy Suite of the Bioconductor Package (http://www.bioconductor.org), using the quantile normalization of Robust Multiarray Average method (each calculation performed at the individual probe level). Fold-changes were calculated by subtracting average log2 DMSO signal from average log2 THZ531 treatment signal. Active transcripts of each cell were defined as average log2(expression) > log2(100) in the corresponding DMSO sample.

### scRNA-seq data processing and cell annotation

Four samples for scRNA-seq were derived from the primary tumor sites of patients diagnosed with OS in our institution. The single cells ultimately obtained from each sample were loaded onto a 10 × Genomics Chromium Single-Cell Chip. Raw data were processed using Cell Ranger (v3.0.2) to align reads, generate feature-barcode matrices, and perform gene expression analysis. Individual data were merged, and low-quality cells were excluded based on the types of genes detected, total number of detected genes, and percentage of mitochondrial genes. The eligible data were normalized, and batch effects were removed. Uniform manifold approximation and projection (UMAP) was performed for unsupervised clustering and cell-type markers were used for the identification of specific cell types.

### Immunohistochemical staining, evaluation, and analysis

Sections of formalin-fixed, paraffin-embedded tissue specimens were incubated with corresponding antibodies overnight at 4 °C, and then incubated with Dako EnVision secondary antibody (Dako, Glostrup, Denmark) for 30 min at room temperature. The intensity and proportion of staining cells was evaluated and scored by 2 independent pathologists without knowledge of patient data. The intensity of staining was scored as: 0 = no staining; 1 = weak staining; 2 = moderate staining; and 3 = strong staining. The extent of staining was scored as: 0 = no positive staining; 1 = positive staining in 1 to 25% of cells; 2 = positive staining in 26 to 50% of cells; 3 = positive staining in 51 to 75% of cells; 4 = positive staining in 75 to 100% of cells. For statistical analyses, the final score was defined as the product of the intensity and extent of staining scores, with a value ranging from 0 to 12. Low/high-expression was defined on the basis of the final score by the X-tile [27].

For OS analyses, the ratios of *LACTB* low expression (score 0–2)/high expression (score 3–12), *CEP55* low expression (score 0–4)/high expression (score 5–12), *SRSF3* low expression (score 0)/high expression (score 1–12), *TCF7L2* low expression (score 0–9)/high expression (score 10–12), and *FOXP1* low expression (score 0–4)/high expression (score 5–12) were used. For LMFS analyses, the ratios of *CEP55* low expression (score 0–4)/high expression (score 5–12), *SRSF3* low expression (score 0)/high expression (score 1–12), *TCF7L2* low expression (score 0–9)/high expression (score 10–12), *FOXP1* low expression (score 0–3)/high expression (score 4–12), *APOLD1* low expression (score 0–4)/high expression (score 5–12), and *DNAJB12* low expression (score 0)/high expression (score 1–12) were used.

### Statistical analysis

Statistical analyses were performed with SPSS version 26.0 software (SPSS Inc., Chicago, IL, USA) and R version 4.1.3 (The R Foundation for Statistical Computing). We compared two groups using the t-test for continuous variables and chi-square test for categorical variables. For the analysis of survival time, OS and LMFS curves were generated by the Kaplan–Meier method and compared with the log-rank test. The independent prognostic value of different factors was evaluated by univariate and multivariate analyses. Significant variables in the univariate analysis (P < 0.05) included in the multivariate analysis based on the Cox proportional hazards regression model. The predictive value of the variables was examined by receiver operating characteristic (ROC) curve analysis. Nomograms of OS and LMFS were established in combination with corresponding clinical factors and the scores of studied genes using the R package “rms”. Calibration plots were derived based on the regression analysis. The “ggDCA” package was used to perform decision curve analysis (DCA) and assess the clinical utility of the nomogram.

## Results

### Integrated analysis revealed the critical role of SEs in osteosarcoma

**Fig. 1:**
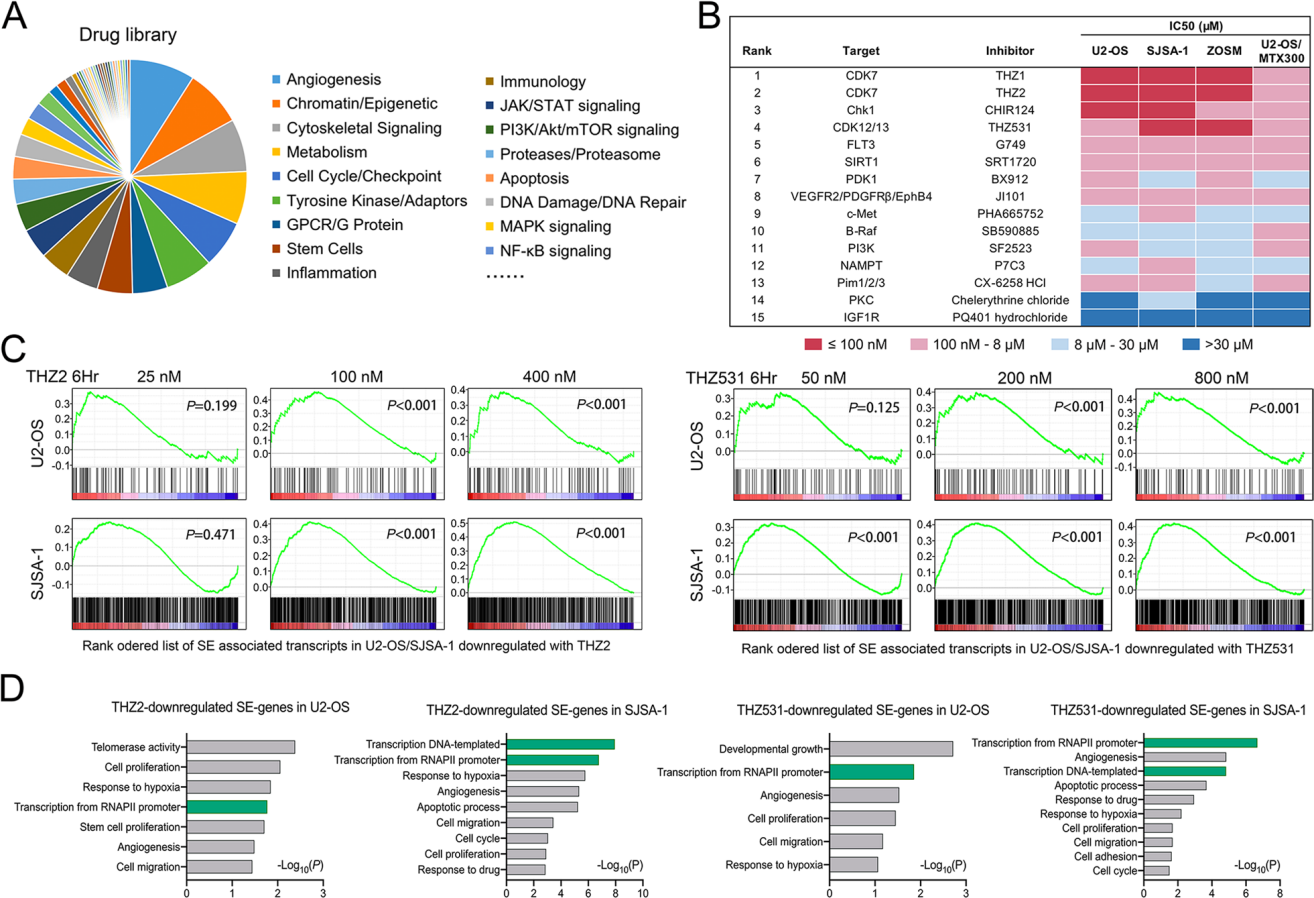
Suppression of SEs induced prominent anti-osteosarcoma effects. **A** Distribution of putative targeting pathways of 150 examined drugs in the library. **B** Summary of top-15 ranked inhibitors and corresponding targets as represented in an ‘IC50 heatmap’ format. **C** Gene Set Enrichment analysis (GSEA) showing the super-enhancer-associated transcript signature enriched in THZ2/THZ531-treated cells versus DMSO-treated cells. **D** Enrichment p-values for selected Gene ontology (GO) functional categories of downregulated SE-associated genes in U2-OS and SJSA-1 cells following treatment with 100 nM THZ1 or 200 nM THZ531

Unbiased high-throughput inhibitor screening was performed in osteosarcoma cell lines using 150 compounds with broad targeting properties (Fig. 1A). High-throughput screening was performed on 2 widely used osteosarcoma cell lines, U2-OS and SJSA-1, a primary cell ZOSM, and an MTX-resistant cell, U2-OS/MTX300. As a result, 15 compounds with marked anticancer effects were identified, and their anti-proliferation IC50 values were measured using a multi-dose assay (Fig. 1B). These top-ranked inhibitors are involved in a number of putative inhibitor classes, including cell cycle, angiogenesis, and cytoskeletal signaling. Notably, we discovered that 3 small molecule inhibitors, THZ1, THZ2, and THZ531, which are thought to target SEs, topped the list and all 4 cell lines were susceptible to them [20, 28]. Among them, THZ2 is an analog of THZ1, with improved pharmacokinetic properties [20].

Our previous work identified the SE catalogue of osteosarcoma cells using the ROSE algorithm (https://bitbucket.org/young_computation/rose), and assigned SEs to their corresponding associated transcripts. With expression microarray analysis and retrieved data from the GEO database (GSE134605), we detected the THZ2- and THZ531-induced selective repression of transcripts driven by osteosarcoma-specific SEs [21] (Fig. 1C; Additional file 1: Figure S1A and B). Gene ontology (GO) analysis showed that down-regulated SE-transcripts were significantly enriched in SE-specific transcriptional activity and various essential cancer-related biological processes (Fig. 1D). These results indicate that SEs have a important role in the malignancy of osteosarcoma, and that SE-associated genes may have potential clinical predictive value for disease progression.

### Classical SE-associated genes are strongly associated with disease progression in patients with osteosarcoma

**Fig. 2:**
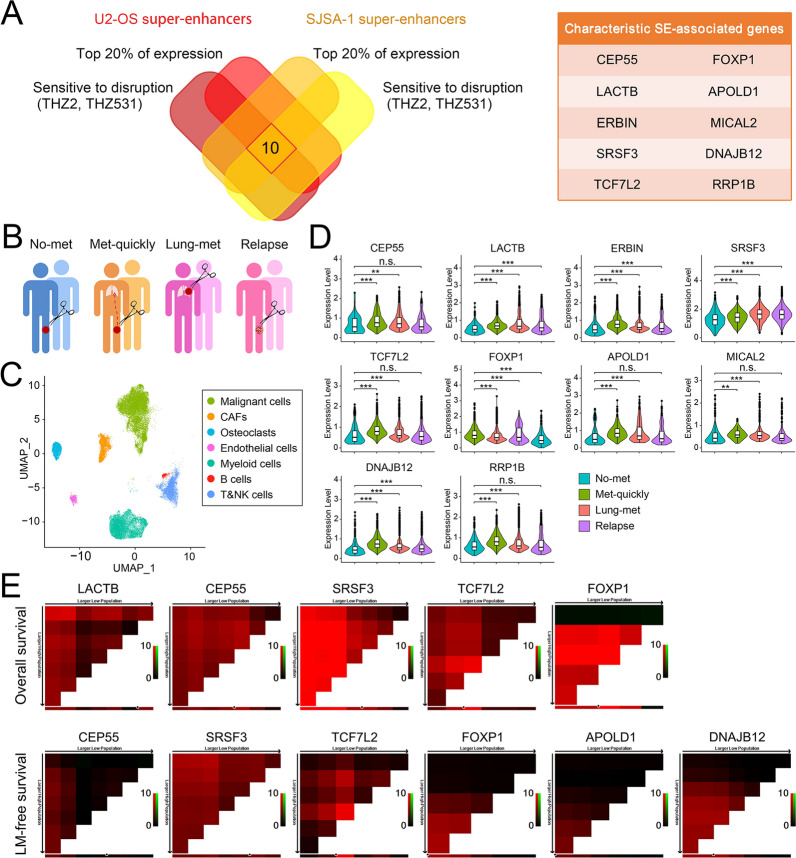
Identification of classical SE-associated genes with prognostic value. **A** Venn diagram showing overlap between super-enhancer-associated genes with characteristic features in U2-OS and SJSA-1 cell lines. Gene names are listed in right panel. **B** Graphical view of sample collection. **C** UMAP plot of all the single cells, with each color-coded for the 7 main cell types in OS lesions. **D** Violin plots showing the normalized expression levels of identified SE-associated genes across the different OS lesions in malignant cells. **E** X-tile plots of candidate SE-associated genes for overall survival and lung metastasis-free survival in 70 cases.

To identify novel prognostic factors, we sought to select genes driven by the most contributing SE elements, and to assess their correlation with outcomes. Given that SEs are hyper-promoted to transcription of associated genes, and are susceptible to disruption by bound transcription factors (e.g., CDK-7, -12, and -13), the following screening criteria were established [19, 28, 29]: (1) associated with shared SEs in U2-OS and SJSA-1 cells, (2) highly sensitive to treatment with both THZ2 and THZ531, (3) ranked among the top 20% of all transcripts in the expression microarray results. Consequently, 10 candidate genes associated with SEs were identified based on SE characteristics (Fig. 2A).

Reports have indicated that SEs drive lineage-specific key genes in somatic cells, and our analysis revealed that most of these 10 genes had a lineage-specific expression pattern, with a higher or the highest expression levels in osteosarcoma (Additional file 1: Figure S2). We retrieved the bulk RNA-seq profiles of 88 osteosarcoma specimens from the GEO database (GSE42352), and found that these candidate SE-associated genes were potentially predictive of patient survival and pulmonary metastasis (Additional file 1: Figure S3). We then performed scRNA-seq analysis on 8 tumor specimens from OS patients (2 local mass without metastasis for more than 2 years, 2 local masses followed by lung metastasis within 6 months, 2 lung metastasis, 2 recurrent tumor) (Fig. 2B). Uniform manifold approximation and projection (UMAP) analysis was performed to the identified main clusters in these specimens and we found that malignant cells, which considered with abnormal infer copy number variation (CNV), make the biggest contribution to the expression signals of these genes (Fig. 2C; Additional file 1: Figure S4 and S5). Their increased signal intensities in malignant cells were strongly associated with the malignancy and disease progression (Fig. 2D). The aforementioned results indicate that the identified SE-associated genes that are expressed in tumor cells of osteosarcoma patients’ primary lesions might be promising prognostic predictors.

As a widely used technology in clinical diagnosis, IHC stains make it easy for pathologists to confirm the biomarker signal of a particular type of cells in complex tissue mass. To further determine the clinical prognostic value of these identified SE-associated genes, we used IHC staining to examine the expression of identified 10 genes in 70 osteosarcoma samples with long-term follow-up data. The X-tile correlation analysis showed that high expression of 5 genes (*LACTB, CEP55, SRSF3, TCF7L2,* and *FOXP1*) predicted poor overall survival (OS), and high expression of 6 genes (*CEP55, SRSF3, TCF7L2, FOXP1, APOLD1,* and *DNAJB12*) predicted poor lung metastasis-free survival (LMFS) (Fig. 2E; Additional file 1: Figure S6).

### Development of the SE-derived IHC signatures for OS and LMFS

To further assess the predictive efficacy and clinical value of these identified SE-associated genes, we examined the IHC data, clinical characteristics, and follow-up information of 212 patients with osteosarcoma who received standard treatment. The mean of the age of the patients were 19.1 years (range, 6–67 years), and 11 (5.2%) patients were > 40 years of age. The median follow-up time was 103.9 months (interquartile range [IQR] 78.2–128.3 months). A total of 159 patients were assigned to the training set and 53 patients to the validation set. There were no significant differences in the clinicopathological characteristics between the 2 groups (Additional file 1: Table S1). IHC scores for the SE-associated genes were calculated by pathologists, and divided into high and low expression groups. In the training set, the optimal cut-off scores for OS and LMFS were generated from the X-tile plots.

Univariate analysis showed that Enneking stage, tumor size, type of surgery, ALP level, and all 5 SE-associated genes were significant prognostic factors for OS. Tumor size, surgery type, ALP level, and 4 SE-associated genes (excluding *CEP55* and *DNAJB12*) were significant prognostic factors for LMFS (all, *P* < 0.05, Table 1). We hypothesized that postoperative risk stratification and prediction of OS or LMFS could be improved if the expression patterns of multiple SE-associated genes were combined into a single index. Multivariate Cox proportional hazards regression analysis was performed using factors with a P-value < 0.05 in the univariate analysis to calculate the prognostic index. A prognostic signature for each group was created as follows: SE-derived OS signature = (0.352 × *LACTB* + 0.195 × *CEP55* + 0.577 × *SRSF3* + 0.546 × *TCF7L2* + 0.591 × *FOXP1*)/2.261; SE-derived LMFS signature = (0.711 × *SRSF3* + 0.324 × *TCF7L2* + 0.374 × *APOLD1* + 0.305 × *FOXP1*)/1.714. The coefficients were calculated by Cox regression analysis, and gene names represents their normalized IHC scores (0 to 1). The location of the primary tumor had a high hazard ratio (HR) but a P value of 0.077; however, because it is a well-recognized prognostic factor it was included in the analysis. Multivariate analysis showed that primary site, Enneking stage, tumor size, ALP level, and SE-derived OS signature were independent prognostic factors for OS. Tumor size, ALP level, and SE-derived LMFS signature were independent prognostic factors for LMFS (Table 2). In particular, the HR of both SE-derived signatures was relatively higher in the OS and LMFS groups.

**Table 1:** Univariate analysis of clinical factors and SE-associated genes for overall survival and lung metastasis-free survival

	OS (Training Set, n = 159)			LMFS (Training Set, n = 141)		
	Hazard Ratio	95% CI of ratio	p-value	Hazard Ratio	95% CI of ratio	p-value
Gender (female vs male)	1.33	0.87 to 2.03	0.20	1.13	0.68 to 1.86	0.65
Age (< 18y vs ≥ 18y)	1.04	0.68 to 1.59	0.88	1.01	0.61 to 1.67	0.99
Primary Site (extremities vs non-extremities)	1.90	0.73 to 4.97	0.077	1.88	0.62 to 5.64	0.13
Histological Type	–	–	0.37	–	–	0.60
Enneking Stage (I/II vs III)	4.56	1.63 to 12.75	< 0.0001	–	–	–
Tumor Size (≤ 8 cm vs > 8 cm)	1.84	1.20 to 2.81	0.0040	3.08	1.89 to 5.04	< 0.0001
Surgery Type (amputation vs limb sparing)	0.64	0.40 to 1.03	0.041	0.52	0.30 to 0.91	0.0096
ALP (< 110/150 vs ≥ 110/150)	2.09	1.34 to 3.25	0.0051	2.36	1.41 to 3.94	0.0056
LDH (< 240 vs ≥ 240)	1.35	0.87 to 2.11	0.16	1.02	0.60 to 1.73	0.95
LACTB (low vs high expression)	2.59	1.41 to 4.77	0.032	–	–	–
CEP55 (low vs high expression)	1.76	1.10 to 2.83	0.042	1.82	1.05 to 3.15	0.067
SRSF3 (low vs high expression)	2.70	1.54 to 4.73	0.014	2.44	1.27 to 4.67	0.047
TCF7L2 (low vs high expression)	2.39	1.32 to 4.35	0.0001	1.86	0.90 to 3.83	0.037
FOXP1 (low vs high expression)	2.60	1.35 to 5.03	< 0.0001	1.84	0.95 to 3.57	0.028
APOLD1 (low vs high expression)	–	–	–	2.07	0.88 to 4.85	0.025
DNAJB12 (low vs high expression)	–	–	–	1.59	0.97 to 2.59	0.067

**Table 2:** Multivariate analysis of clinical factors and SE-derived signatures for overall survival and lung metastasis-free survival

	Overall survival			Lung metastasis-free survival			
	Hazard Ratio	95% CI of ratio	p-value		Hazard Ratio	95% CI of ratio	p-value
Primary site (extremities vs non-extremities)	2.77	1.29 to 5.96	0.009	Tumor Size (≤ 8 cm vs > 8 cm)	2.70	1.49 to 4.91	0.001
Enneking Stage (I/II vs III)	4.44	2.43 to 8.15	< 0.001	ALP (< 110/150 vs ≥ 110/150)	2.19	1.16 to 4.12	0.015
Tumor Size (≤ 8 cm vs > 8 cm)	1.64	1.02 to 2.63	0.042	SE-derived LMFS-signature (low vs high expression)	4.85	1.61 to 14.61	0.005
ALP (< 110/150 vs ≥ 110/150)	1.91	1.10 to 3.30	0.022	–	–	–	–
SE-derived OS-signature (low vs high expression)	6.38	2.38 to 17.08	< 0.001	–	–	–	–

Statistical analysis: the multivariate Cox proportional-hazards regression

### Validation and prediction accuracy of the signatures and constructed models

**Fig. 3:**
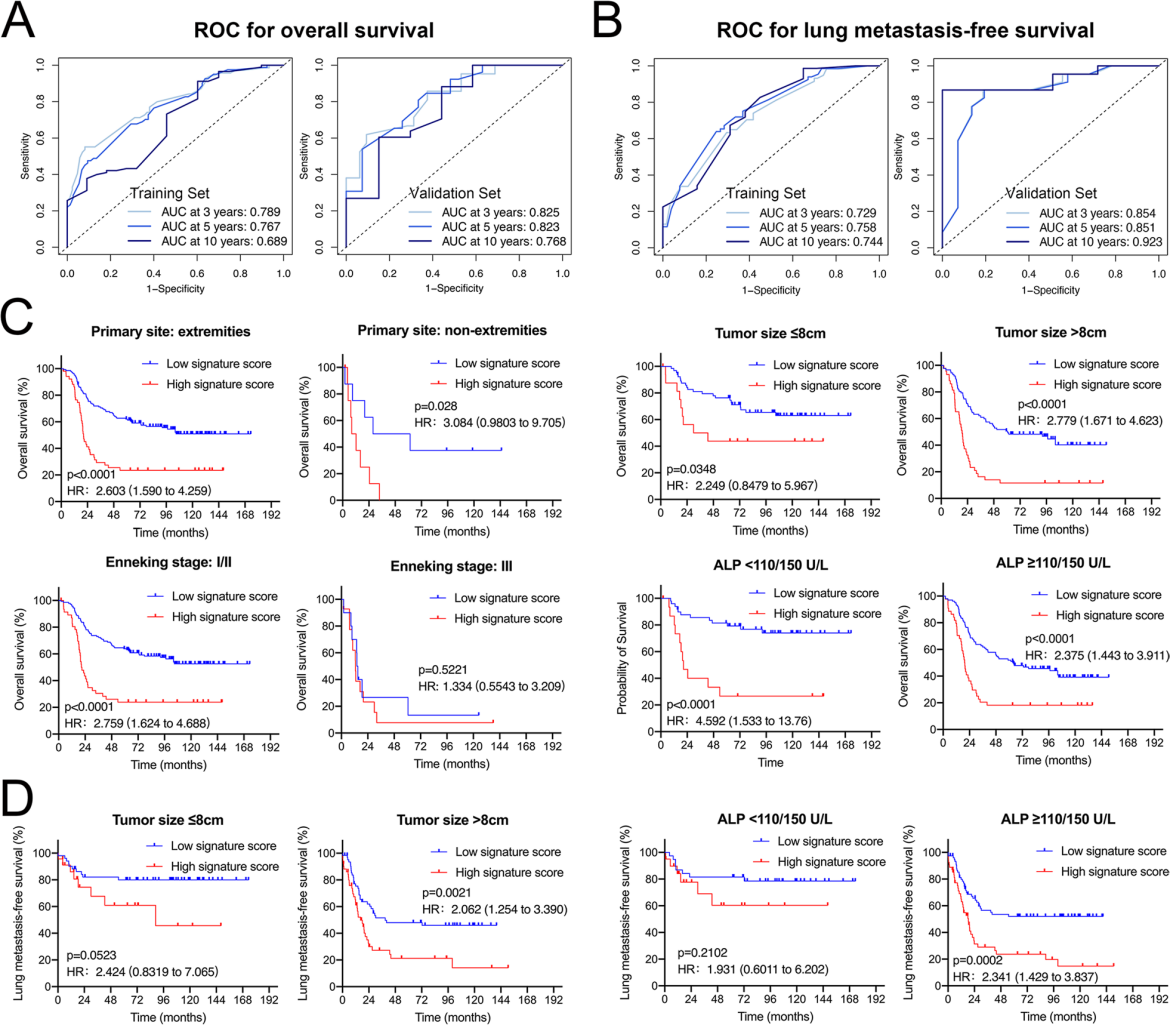
Predictive accuracy of the developed models, and subgroup analysis of SE-derived signatures. Time-dependent ROC curves of developed models for overall survival (**A**) and lung metastasis-free survival (**B**) at 3-, 5-, 10-year in the training and validation sets respectively. Kaplan-Meier analysis of overall survival (**C**) and lung metastasis-free survival (**D**) for all 212 patients according to the SE-derived signatures stratified by clinicopathological risk factors.

**Fig. 4:**
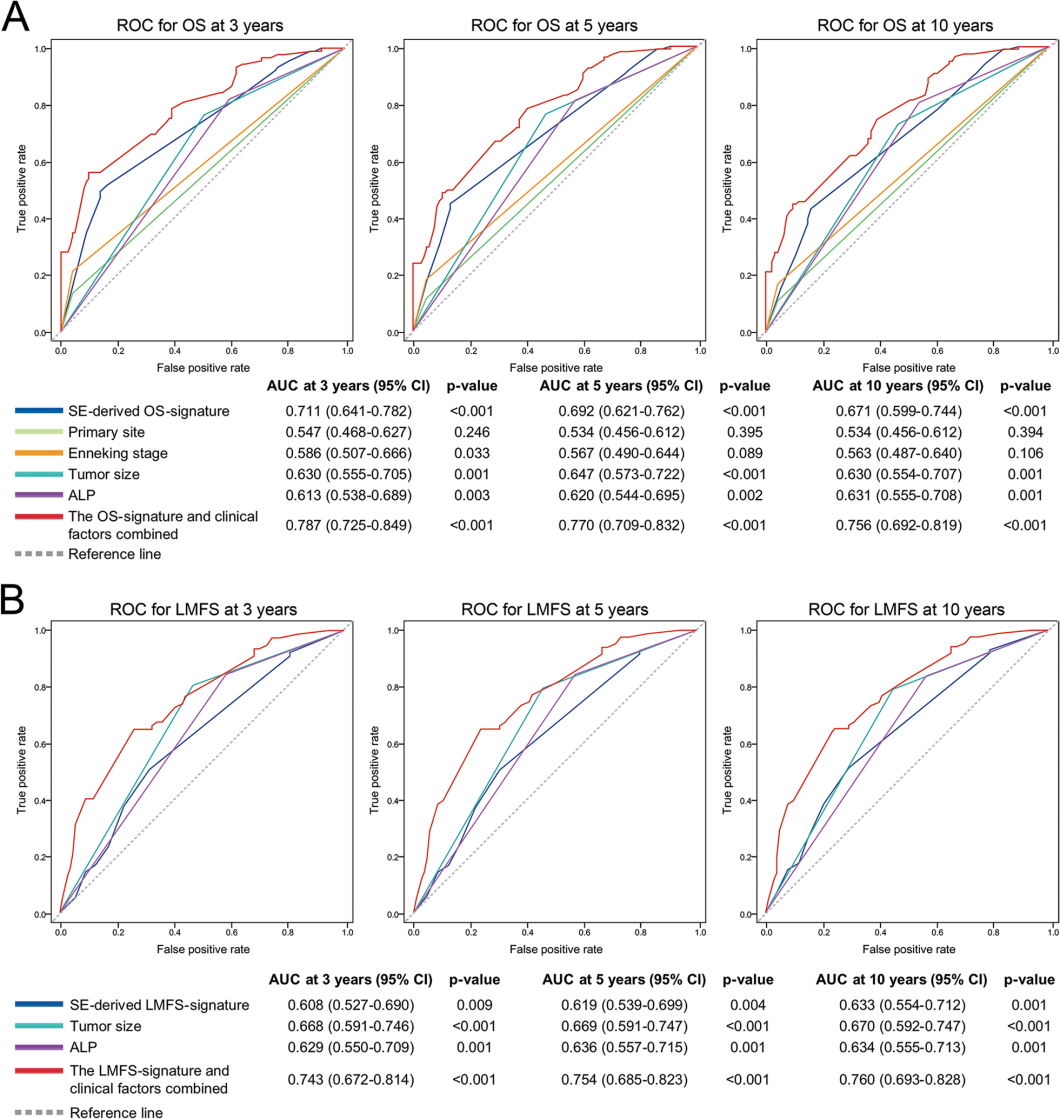
Time-dependent ROC curves comparing the prognostic accuracy of SE-derived signatures for survival (**A**) and lung metastasis (**B**) with corresponding clinicopathological risk factors in all patients

Time-dependent receiver operating characteristic (ROC) curve analysis indicated that the 2 SE-derived signature-based prognostic models exhibited substantial predictive accuracy for OS and LMFS at different follow-up times in the training set and validation set (Fig. 3A and B). When stratified by clinicopathological risk factors, OS was much shorter in the high-risk signature group than in the low-risk signature group in the subgroup analysis, as was LMFS (Fig. 3C and D). The insignificant difference in the Enneking stage III subgroup is likely due to the small number of cases. In the ROC analysis of 212 patients, the SE-derived OS signature showed higher predictive accuracy than any clinicopathological risk factor or single SE-associated gene alone at 3, 5, and 10 years (Fig. 4A; Additional file 1: Figure S7A). The SE-derived LMFS signature also had good predictive accuracy, higher than any single gene alone (Fig. 4B; Additional file 1: Figure S7B). As such, the SE-derived signatures have good predictive performance for OS and LMFS in patients with osteosarcoma, and can add prognostic value to clinicopathological prognostic features.

## Nomogram creation and clinical utility assessment

**Fig. 5:**
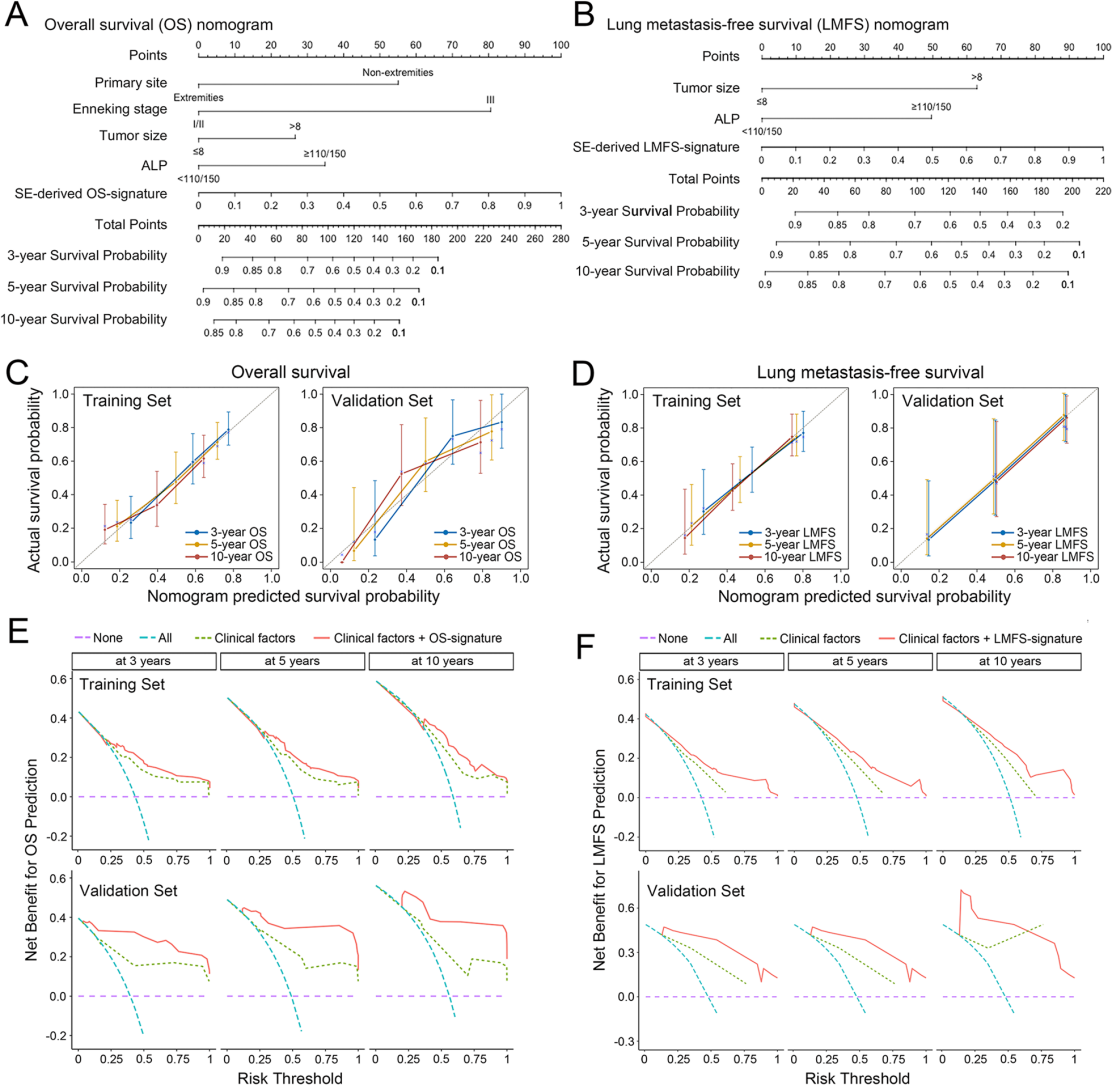
Development and evaluation of nomograms for predicting osteosarcoma survival and lung metastasis. Nomograms for predicting the 3-, 5-, and 10-year overall survival (**A**) and lung metastasis-free survival (**B**) of patients with osteosarcoma. Calibration plots (**C**, **D**) and decision curve analysis (**E**, **F**) of these two proposed nomograms in both the training and validation sets.

To provide clinicians with a quantitative method to predict survival time and lung metastasis risk in patients with osteosarcoma, we constructed nomograms for OS and LMFS that integrated the SE-derived IHC signatures and clinical risk factors examined above (Fig. 5A and B). The calibration plots showed that the predictive accuracy of the 2 nomograms compared favorably with ideal models at 3, 5, and 10 years (Fig. 5C and D). Finally, the clinical value of these nomograms was evaluated by decision curve analysis (DCA). Both nomograms had promising clinical value. The threshold probability ranges for OS and LMFS at 3, 5, and 10 years indicated that the nomograms provided a better net benefit than all-or-none treatment in both the training and validation sets (Fig. 5E and F). Therefore, the identified SE-derived signatures can substantially improve the net benefit of the clinical model. To facilitate clinical use of these nomograms, free web interfaces for their implementation are provided (https://superenhancer-fahsysu.shinyapps.io/PredictOS-Osteosarcoma/, https://superenhancer-fahsysu.shinyapps.io/PredictLMFS-Osteosarcoma/).

## Discussion

**Fig. 6:**
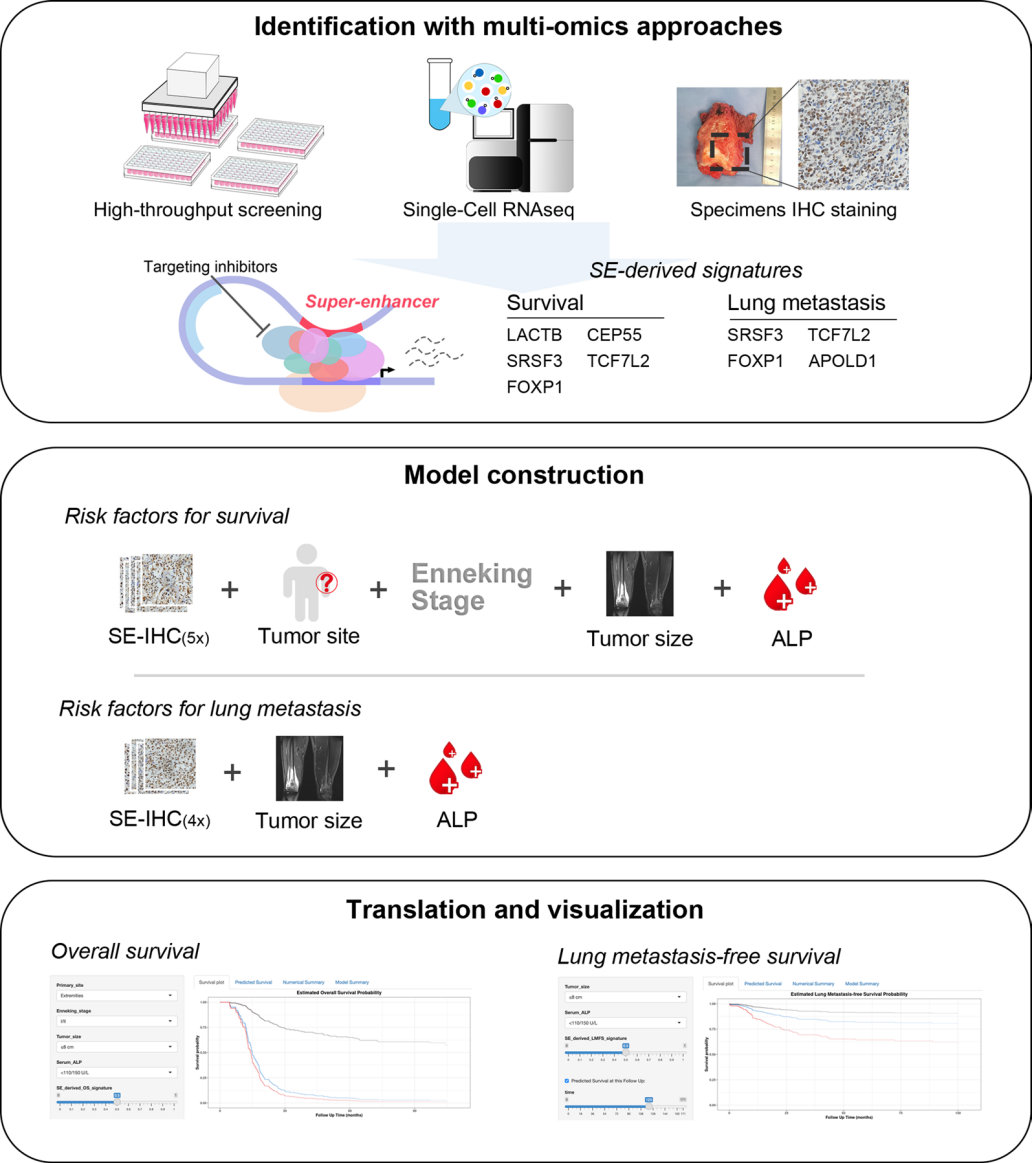
Schematic depiction of the study process

This study identified a cluster of screening genes driven by classical SEs with promising prognostic value in osteosarcoma. We then developed and validated 2 novel SE-based prognostic signatures based on IHC analysis to improve the prediction of OS and LMFS after surgery in patients with osteosarcoma. Our results indicated that these signatures can accurately classify patients into high-risk and low-risk groups with significant differences in mortality and lung metastases at 3, 5, and 10 years. Furthermore, both SE-derived signatures exhibited sound predictive performance, even better than well-known clinicopathological risk factors. When stratified by clinicopathological factors, these signatures remained significant prognostic indicators and provided prognostic value, complementing the clinical model. Finally, prognostic models using well-proven clinical variables for predicting OS and LMFS combined with the signatures were provided (Fig. 6).

Current treatment for newly diagnosed osteosarcoma patients consists of a neoadjuvant chemotherapy cycle composed of 3 to 4 cytotoxic agents followed by surgical resection of the tumor and an additional postoperative chemotherapy cycle [30]. Despite advances in the treatment of osteosarcoma, 5-year survival rates remain far from satisfactory with rates as low as 20% for patients with metastatic disease [29, 31]. Recent studies have indicated there is no significant difference in survival between patients who undergo limb salvage or amputation, which may be due to reliable preoperative evaluation and accurate selection of the type of surgery [6, 32]. Postoperative treatment, including adjuvant chemotherapy, radiotherapy, and gene therapy, can eliminate residual cancer cells and maintain disease-free survival [33]. However, due to the diversity of treatment combinations, unavoidable toxicities, and high treatment costs, there is an urgent need for effective predictive tools to determine the prognosis for individual patients and thus help guide postoperative management decisions.

The relations between prognosis and SE-driven gene expression have been reported in various other cancers, including breast cancer, neuroblastoma, and gastric cancer [34–36]. Based on this study and our prior work, we hypothesized that genes driven by key osteosarcoma-specific SEs have potential prognostic value [21]. The present study showed that IHC scores of the identified SE-associated genes are strongly associated with OS and LMFS in osteosarcoma patients. Through a retrospective analyses of 212 patients with long-term follow-up data, the identified SE-associated genes showed significant predictive performance for mortality and lung metastasis. The SE-derived signatures that were developed add substantial prognostic value to the clinicopathological prognostic signature. Thus, our prognostic model is a novel and reliable predictive tool for the postoperative assessment of patients with osteosarcoma.

Several RNA-based classifiers with prognostic value in osteosarcoma have been identified in previous studies, and the nomograms constructed with these classifiers exhibit strong predictive performance. For example, Fu et al. developed a prognostic signature associated with the inflammatory response, and built a nomogram that included sex, age, and metastatic status [37]. Lei et al. identified a cluster of ferroptosis-related genes strongly associated with immune status, and built a nomogram that included sex, age, tumor site, and metastatic status [38]. In addition, Ouyang et al. identified a cluster of SE-associated genes with prognostic values and established a nomogram for OS that included age, sex, necrosis, and recurrence [39]. However, due to the low incidence of osteosarcoma and the scarcity of patient specimens, almost all reported prognostic analyses are based on existing open databases, namely the GEO database and the TARGET database, and are inevitably limited by small sample sizes, lack of clinical information, and heterogeneity of data from diverse sources. Predictors of adverse outcomes, including detectable primary metastases, axial tumor sites, larger tumor sizes, and higher ALP and lactate dehydrogenase (LDH) levels have been well documented in previous large-scale clinical trials [6, 40, 41]. More comprehensive clinical information was considered to develop signatures with significant promotional value. Consistent with previous large-scale trials, most of the proven clinicopathological factors for predicting mortality and lung metastasis showed significant prognostic performance in our results, and were incorporated into the nomograms. Furthermore, IHC is an inexpensive and easy-to-perform pathological technique, which is routinely used in clinical practice. As protein-based factors, the SE-derived signatures can be easily calculated conveniently and have clinical utility.

As a large cluster of active enhancers, SEs can promote higher levels of transcription of their target genes and are more sensitive to perturbation than typical enhancers [19]. In the current study, we identified classical SEs and associated genes in osteosarcoma cells according to these unique features. Their close correlations with disease progression were determined through integrating multi-omics data. The cancer-related biological functions of SE-associated genes included in our signatures have been investigated previously. The *SRSF3* gene (serine and arginine rich splicing factor 3) has been reported to play a critical role in cell proliferation by promoting the G2/M transition, and preventing the death of apoptotic cells in cancers where it functions as an oncogene [42]. *TCF7L2* (transcription factor 7 like 2) is a key factor in the Wnt signaling pathway, one of the 3 main cancer stem cell (CSC) pathways, and functions as an oncogene in osteosarcoma [43]. Transactivated by ERK/JNK-c-JUN/c-FOS, *FOXP1* (forkhead box P1) drives osteosarcoma development by regulating the cascade of p53-P21/RB signaling [44]. As a centrosomal protein, CEP55 (centrosomal protein 55) is a key regulator of cytokinesis and promotes osteosarcoma malignancy through the AKT pathway [45]. *APOLD1* (apolipoprotein L domain containing 1) is an endothelial cell early response protein that may play an important role in the regulation of endothelial signaling pathways and vascular function [46]. *LACTB* (lactamase beta) is evolutionarily related to bacterial penicillin-binding/B-lactamase proteins, and its role in tumor biology remains controversial [47]. Keckesova et al. observed that *LACTB* inhibits the proliferation of breast cancer cells by altering mitochondrial lipid metabolism [48]. Zeng et al. observed that *LACTB* inhibits the progression of colorectal cancer by modulating the stability of p53 [49]. However, Peng et al. reported that *LACTB* promotes metastasis in nasopharyngeal carcinoma by activating of ERBB3/EGFR-ERK signaling [50]. Xie et al. reported a relation between high levels of *LACTB* expression and a poor prognosis in pancreatic adenocarcinoma [51]. Notably, the oncogenic properties of *LACTB* were observed in our preliminary experiments (data not shown). In our literature review, we found that genes such as *SRSF3, TCF7L2, FOXP1,* and *CEP55* have been documented to be associated with epithelial-mesenchymal transition (EMT) in tumor cells [52–55]. EMT is the process in which polarized epithelial cells lose their adhesive characteristics to acquire a mesenchymal cell functional phenotype, and it has been demonstrated to impact various aspects of tumor behavior, including invasion, metastasis, and drug resistance [56]. This implies that SEs may support the EMT pathway in osteosarcoma cells and thus play an important role in disease progression.

This study is limited because it is retrospective design and single-centered. It ensures consistency of treatment but also potentially limits the external validity of our findings and makes them less relevant globally. Furthermore, although we collected as much clinical information as possible, our study lacks some reported clinical features, such as histological response to chemotherapy and body mass index (BMI), primarily due to the extended duration of our follow-up.

## Conclusions

Combined with high-throughput screening and multi-omics assays, the current study revealed a correlation of SE-associated gene expression in malignant cells of osteosarcoma specimens and prognosis. SE-derived signatures, which constructed based on the IHC scores of multiple SE-associated genes, have significant efficiencies on predicting overall survival and lung metastasis in patients with osteosarcoma undergoing standard treatment. As independent risk factors, these signatures can effectively classify osteosarcoma patients into low-risk and high-risk groups, thus adding prognostic value to traditional clinicopathological risk factors. Integrative prognostic models respectively for predicting OS and LMFS were developed, and both showed robust accuracy. In recent years, researchers have used various data, including clinical, molecular, and imaging result to analyze and identify valuable prognostic indicators for osteosarcoma patients. However, limitations such as a small number of cases, incomplete external data information, and high usage costs have constrained the development and widespread clinical application of prognostic models. Our prognostic models might facilitate patient counselling and more individualized management of patients with osteosarcoma.

### Supplementary Information

**Additional file 1: Figure S1.** Selective suppression of super-enhancer-associated genes by THZ531. **A** Heatmap showing the change of global active transcripts in U2-OS and SJSA-1 cells following treatment with 50, 200, and 800 nM THZ531 for 6 hours. **B** Box plots showing log2-fold changes of transcripts associated with the total pool of all enhancers (ALL), typical-enhancers (TE), and super-enhancers (SE) upon treatment with THZ531. **Figure S2.** mRNA expression level of 7 identified SE-associated genes across various types of human cancer cells. Data were retrieved from CCLE project from Broad Institute. **Figure S3.** Kaplan–Meier survival analysis for 88 patients with osteosarcoma classified with mRNA expression levels of representative SE-associated genes. **Figure S4.** Heatmap showing specific marker gene expressions for each of the 7 clusters indicated. Colors from yellow to purple indicate the relative expression levels from high to low. **Figure S5.** Identification of malignant cells and distribution of SE-gene expressed cells in subclusters. **A** The hierarchical heatmap showing large-scale CNVs of the malignant cells. **B** Proportion of each subcluster and SE-gene expressed cells in whole OS samples. **Table S1.** Characteristics of the included patients. **Figure S7.** Time-dependent ROC curves compare the prognostic accuracy of the SE-derived signatures with single SE-associated genes in all patients.

## Data Availability

The gene expression microarray data have been deposited in the NCBI Gene Expression Omnibus database under the accession code GSE250566. All the other data supporting the findings of this study are available within the article and its Additional files without any restrictions or can be obtained from the corresponding author upon reasonable request.

## Abbreviations

SE: Super-enhancer
OS: Overall survival
LMFS: Lung metastasis-free survival
LACTB: Lactamase beta
CEP55: Centrosomal protein 55
SRSF3: Serine and arginine rich splicing factor 3
TCF7L2: Transcription factor 7 like 2
FOXP1: Forkhead box P1
APOLD1: Apolipoprotein L domain containing 1
PFS: Progression-free Survival
IHC: Immunohistochemistry
ATCC: American Type Cell Collection
ALP: Alkaline phosphatase
LDH: Lactate dehydrogenase
IC50: Half-maximal inhibitory concentration
DMSO: Dimethyl sulfoxide
UMAP: Uniform manifold approximation and projection
ROC: Receiver operating characteristic
DCA: Decision curve analysis
MTX: Methotrexate
CDK7/12/13: Cyclin dependent kinase 7/12/13
scRNA-seq: Single cell RNA sequencing
CNV: Copy number variation
HR: Hazard ratio
EMT: Epithelial–mesenchymal transition
GSEA: Gene set enrichment analysis
