# Supplementary material for: Prognostic and predictive value of super-enhancer-derived signatures for survival and lung metastasis in osteosarcoma
Source: J Transl Med. 2024 Jan 22;22:88. doi: 10.1186/s12967-024-04902-8 (PMC10801997; doi:10.1186/s12967-024-04902-8)
Supplement: Supplementary file 1 — Additional file 1: Figure S1. Selective suppression of super-enhancer-associated genes by THZ531. A Heatmap showing the change of global active transcripts in U2-OS and SJSA-1 cells following treatment with 50, 200, and 800 nM THZ531 for 6 hours. B Box plots showing log2-fold changes of transcripts associated with the total pool of all enhancers (ALL), typical-enhancers (TE), and super-enhancers (SE) upon treatment with THZ531. Figure S2. mRNA expression level of 7 identified SE-associated genes across various types of human cancer cells. Data were retrieved from CCLE project from Broad Institute. Figure S3. Kaplan–Meier survival analysis for 88 patients with osteosarcoma classified with mRNA expression levels of representative SE-associated genes. Figure S4. Heatmap showing specific marker gene expressions for each of the 7 clusters indicated. Colors from yellow to purple indicate the relative expression levels from high to low. Figure S5. Identification of malignant cells and distribution of SE-gene expressed cells in subclusters. A The hierarchical heatmap showing large-scale CNVs of the malignant cells. B Proportion of each subcluster and SE-gene expressed cells in whole OS samples. Table S1. Characteristics of the included patients. Figure S7. Time-dependent ROC curves compare the prognostic accuracy of the SE-derived signatures with single SE-associated genes in all patients. [file 12967_2024_4902_MOESM1_ESM.docx]

**Additional Results**


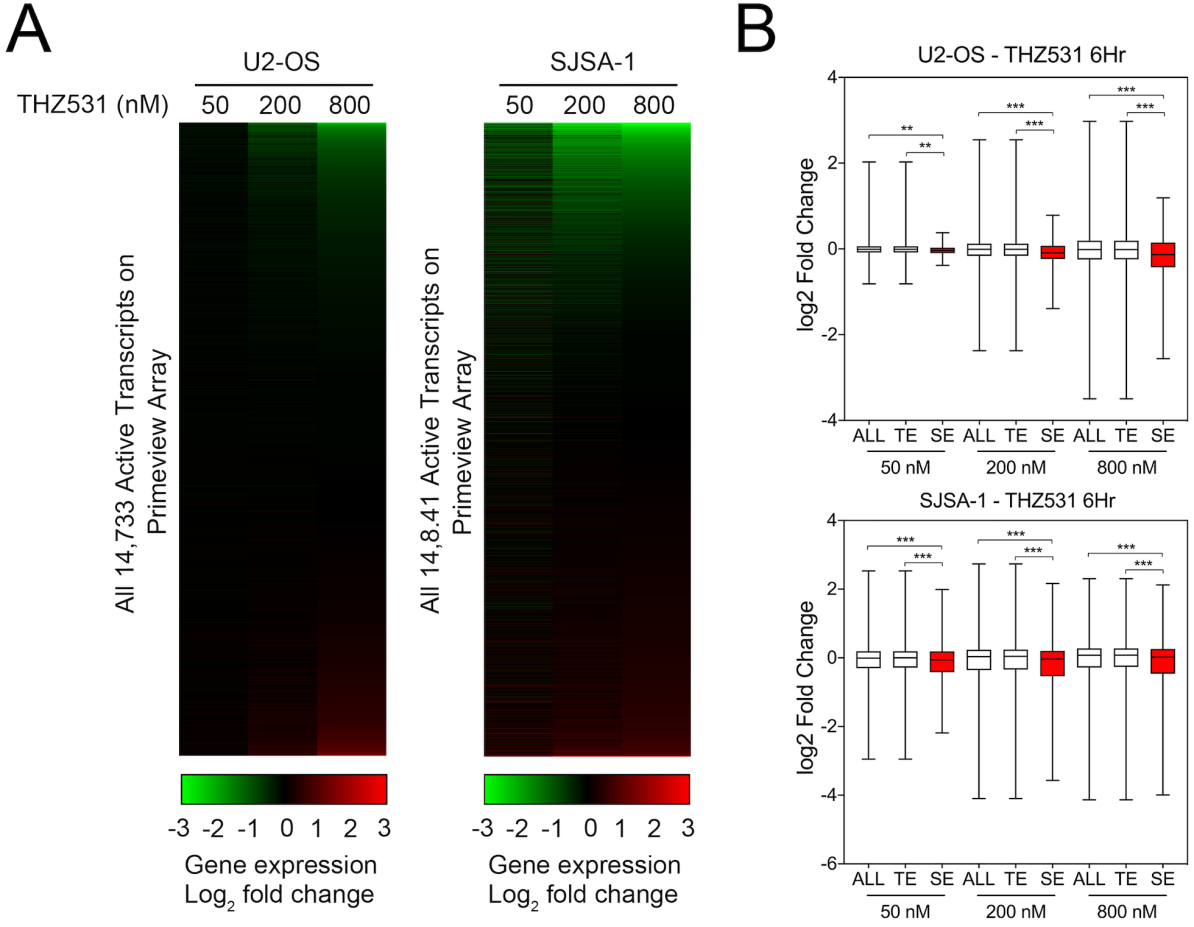


Additional file 1: Figure S1.Selective suppression of super-enhancer-associated genes by THZ531

(A) Heatmap showing the change of global active transcripts in U2-OS and SJSA-1 cells following treatment with 50, 200, and 800 nM THZ531 for 6 hours. (B) Box plots showing log2-fold changes of transcripts associated with the total pool of all enhancers (ALL), typical-enhancers (TE), and super-enhancers (SE) upon treatment with THZ531.


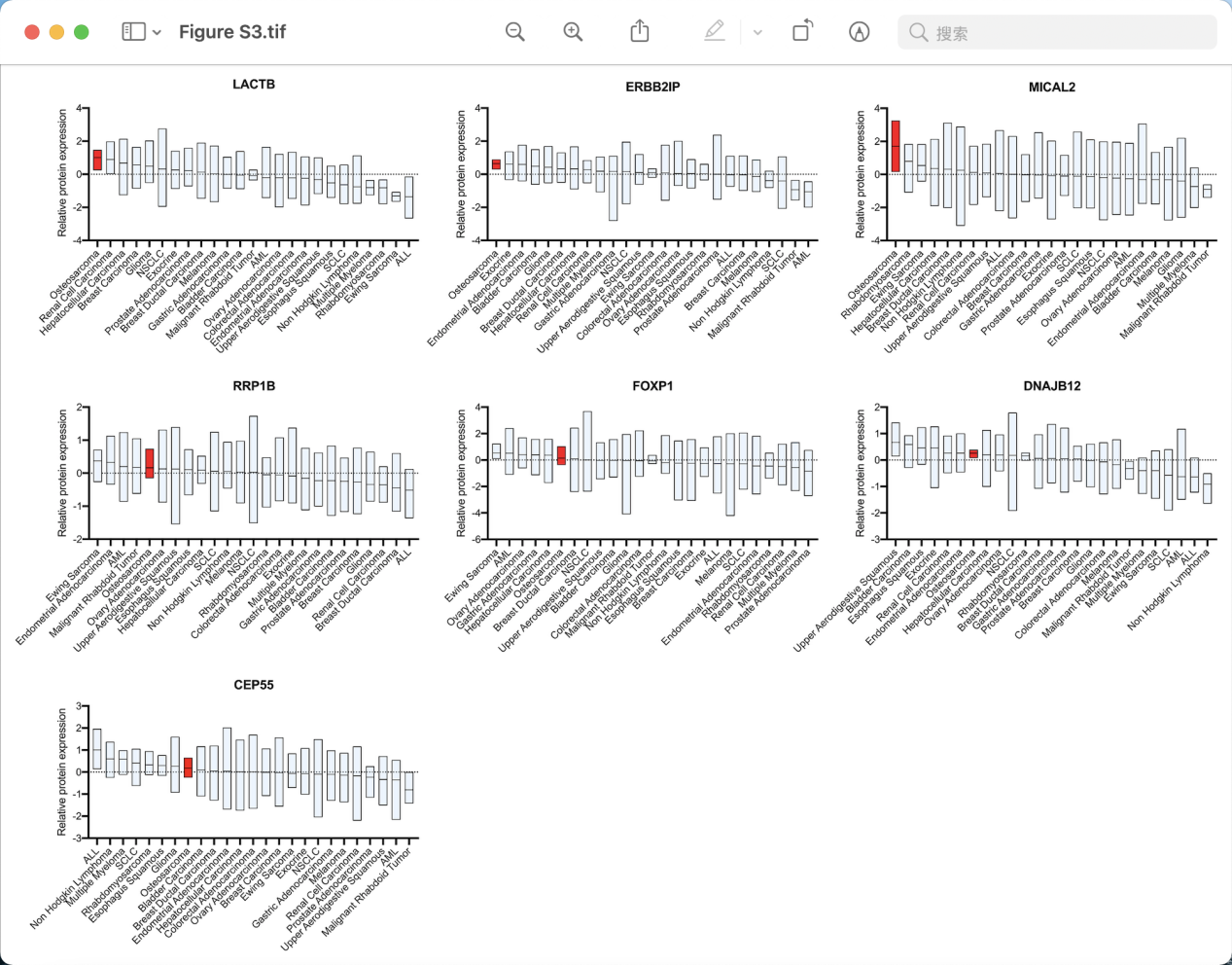


Additional file 1: Figure S2. mRNA expression level of 7 identified SE-associated genes across various types of human cancer cells. Data were retrieved from CCLE project from Broad Institute.


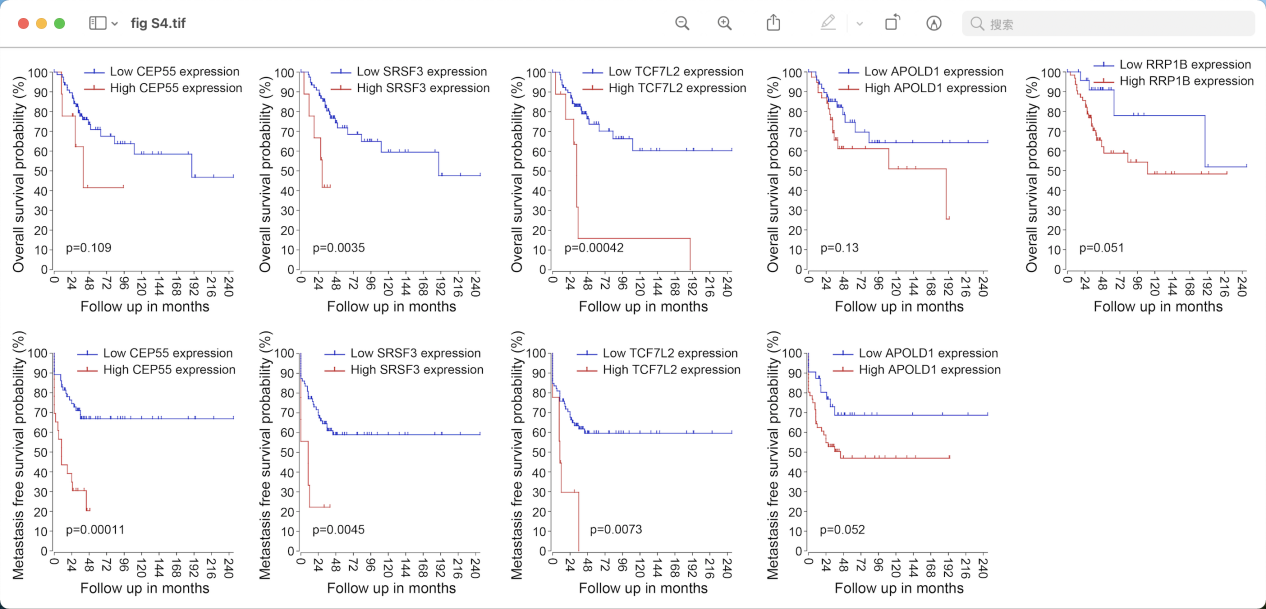


Additional file 1: Figure S3. Kaplan-Meier survival analysis for 88 patients with osteosarcoma classified with mRNA expression levels of representative SE-associated genes.


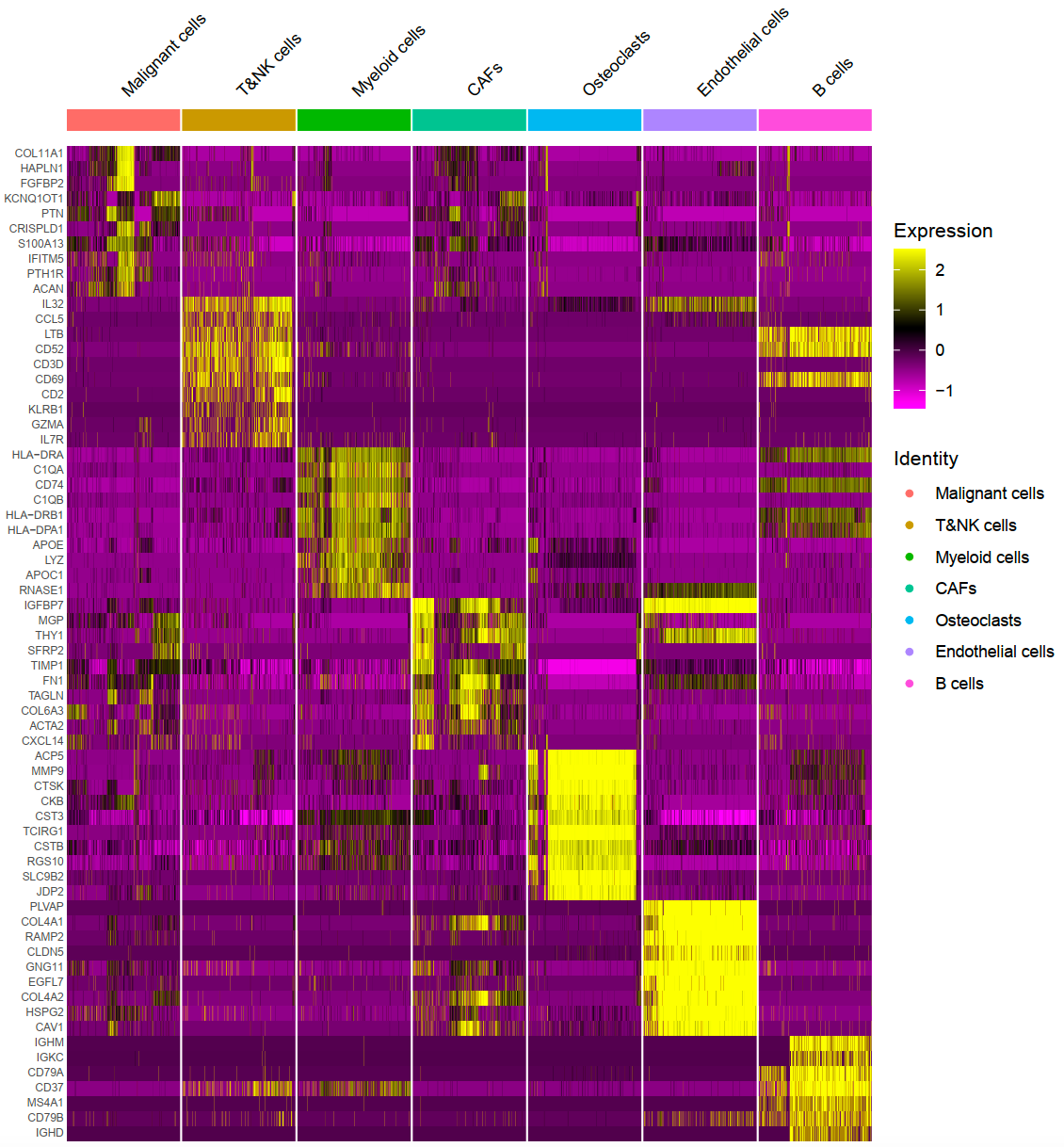


Additional file 1: Figure S4. Heatmap showing specific marker gene expressions for each of the 7 clusters indicated. Colors from yellow to purple indicate the relative expression levels from high to low.


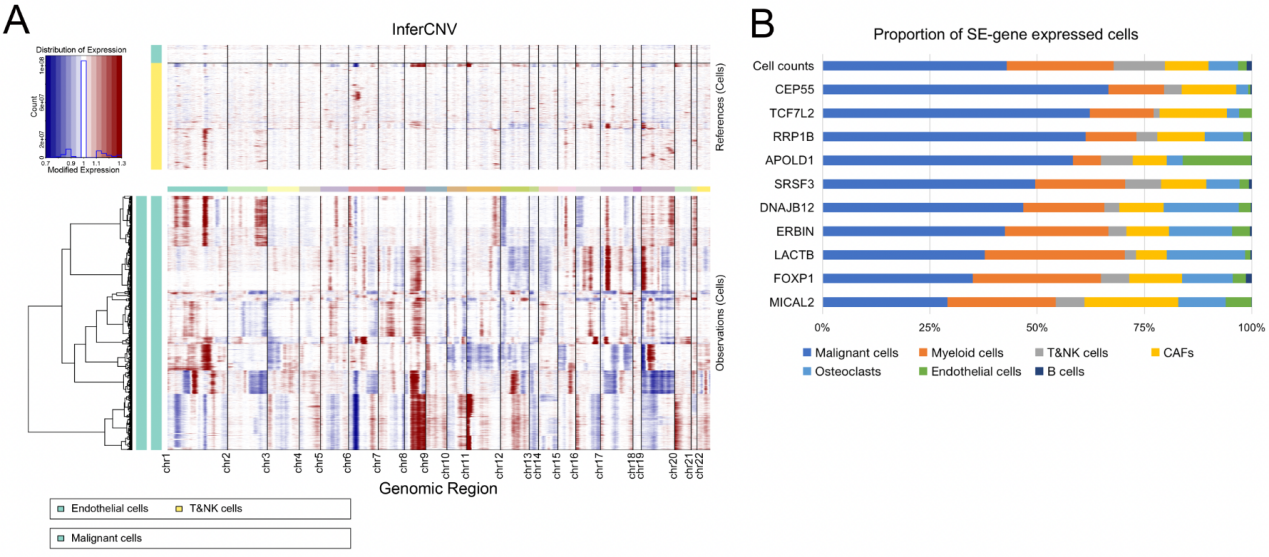


Additional file 1: Figure S5. Identification of malignant cells and distribution of SE-gene expressed cells in subclusters.

(A) The hierarchical heatmap showing large-scale CNVs of the malignant cells. (B) Proportion of each subcluster and SE-gene expressed cells in whole OS samples.


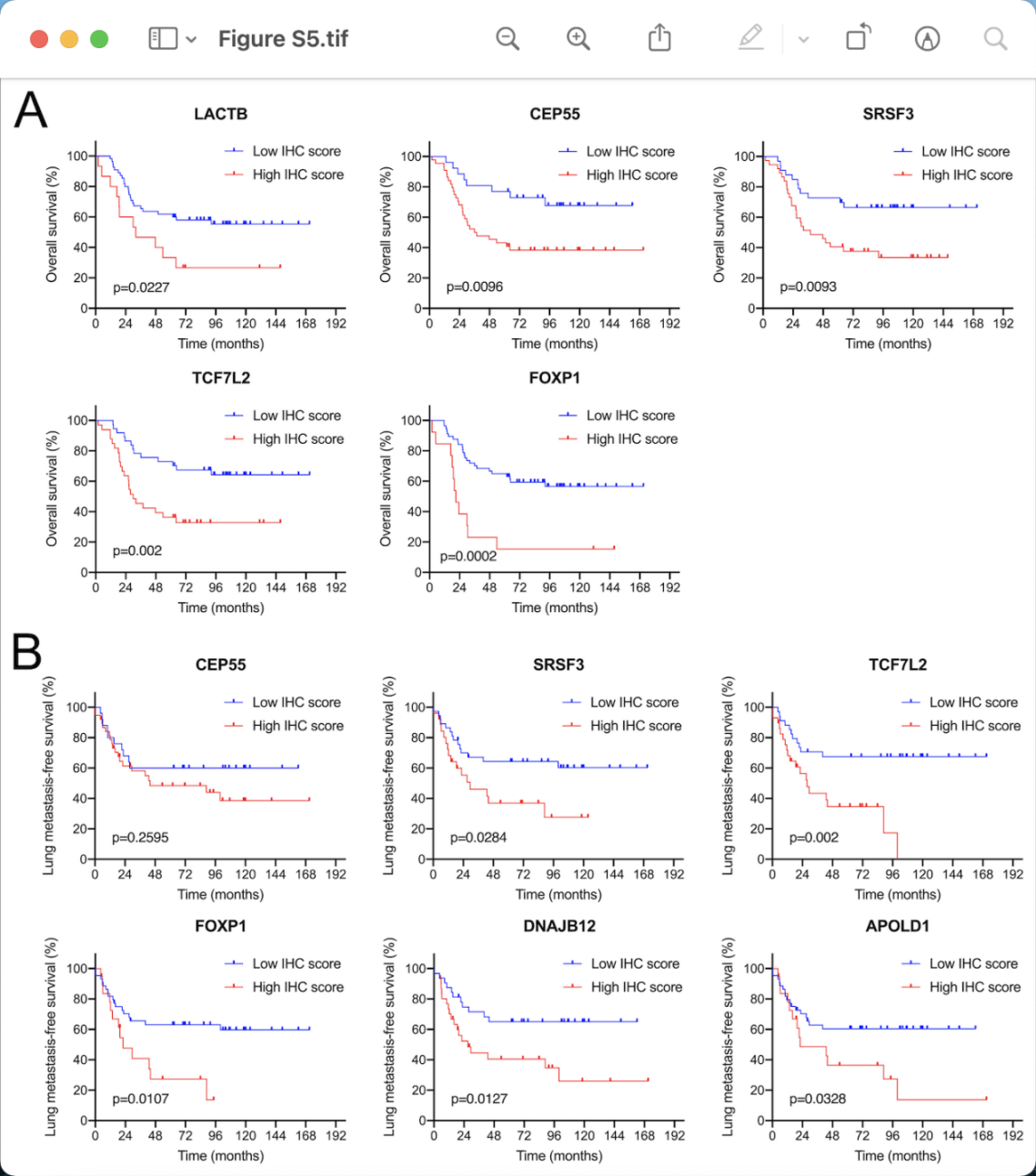


Additional file 1: Figure S6. Kaplan-Meier overall survival and lung metastasis-free survival curves for 70 patients with osteosarcoma classified with optimum cutoff IHC scores of indicated SE-associated genes.


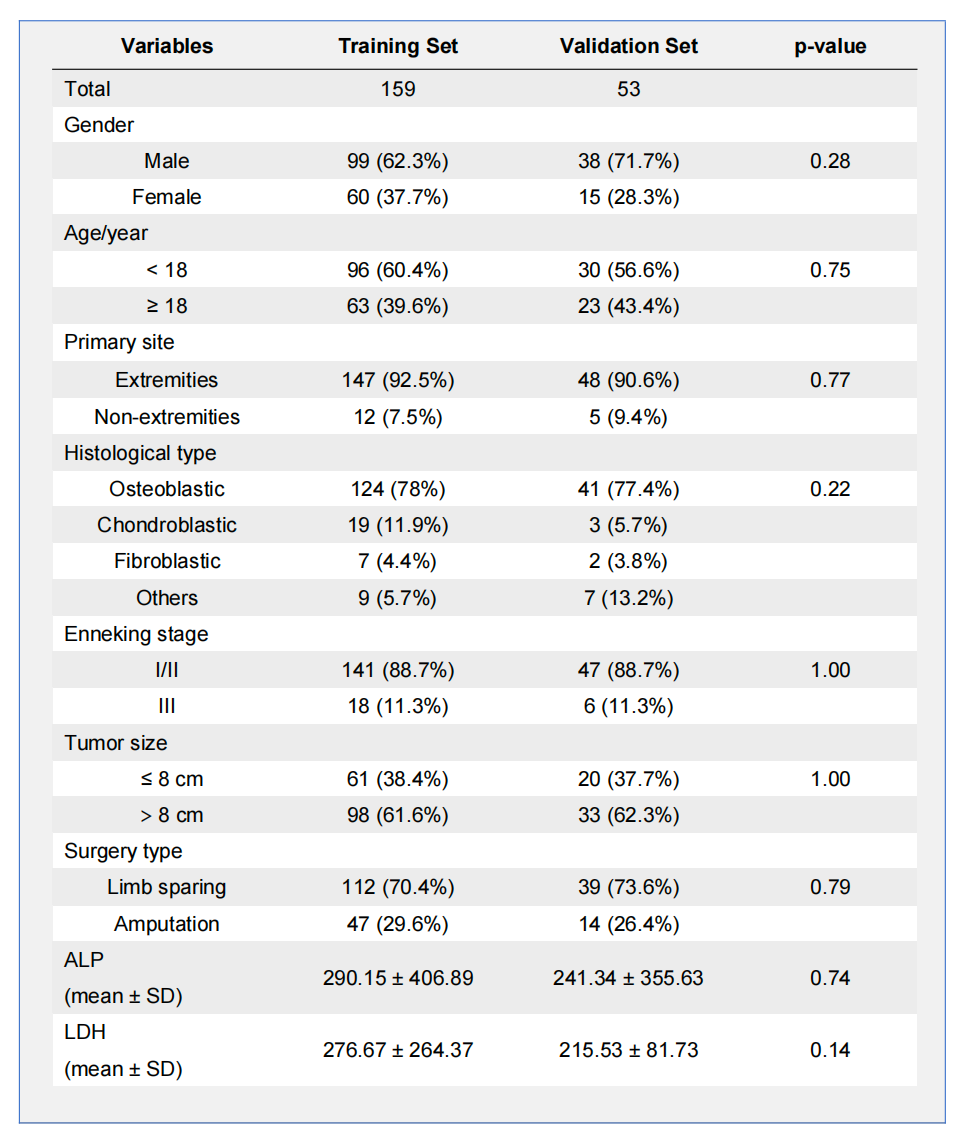


Additional file 1: Table S1. Characteristics of the included patients.


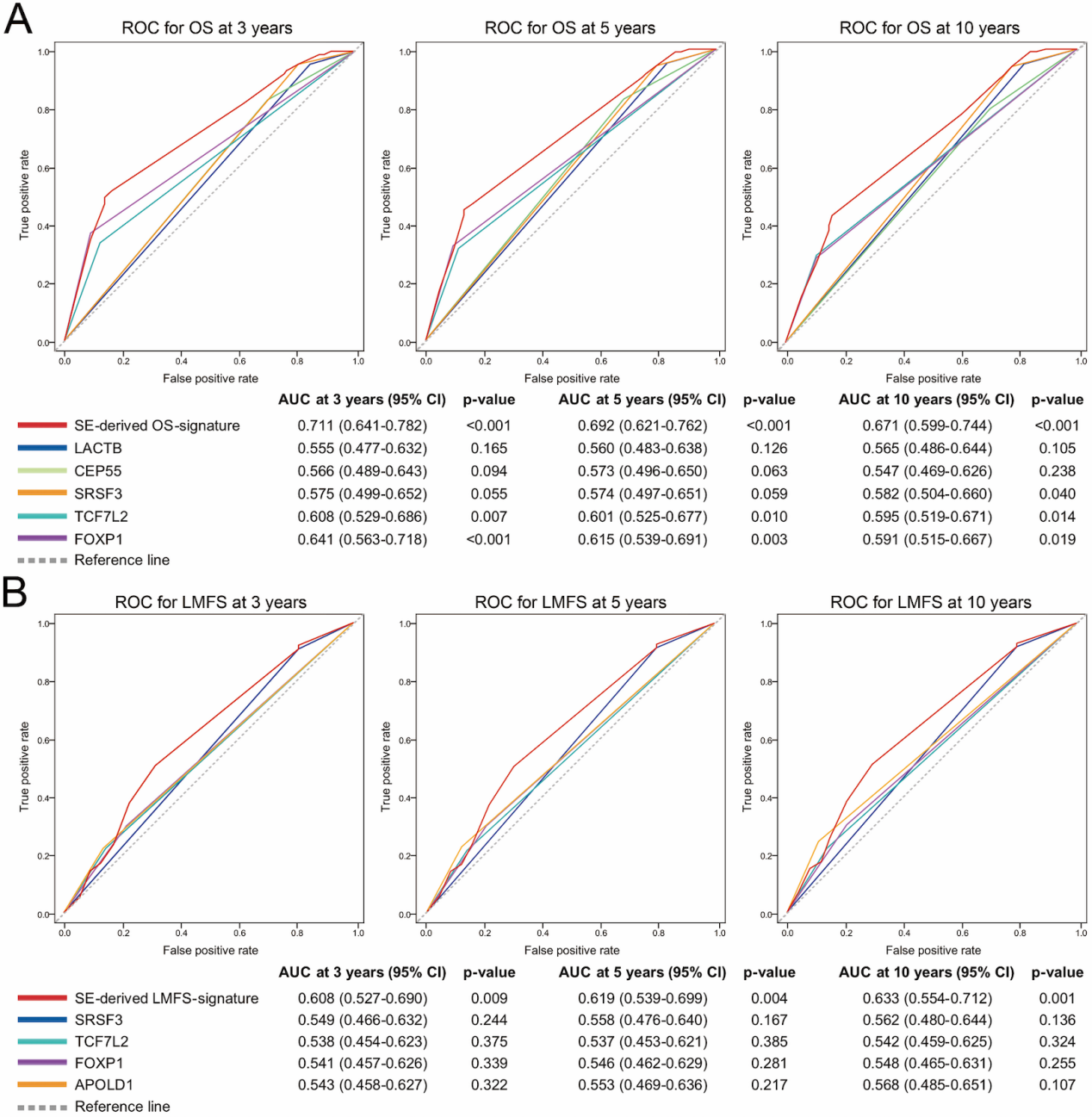


Additional file 1: Figure S7. Time-dependent ROC curves compare the prognostic accuracy of the SE-derived signatures with single SE-associated genes in all patients.

1. Isakoff MS, Bielack SS, Meltzer P, Gorlick R. Osteosarcoma: Current Treatment and a Collaborative Pathway to Success. J Clin Oncol. 2015;33(27):3029-35.

1. Isakoff MS, Bielack SS, Meltzer P, Gorlick R. Osteosarcoma: Current Treatment and a Collaborative Pathway to Success. J Clin Oncol. 2015;33(27):3029-35.

2. Ferrari S, Smeland S, Mercuri M, Bertoni F, Longhi A, Ruggieri P, et al. Neoadjuvant chemotherapy with high-dose Ifosfamide, high-dose methotrexate, cisplatin, and doxorubicin for patients with localized osteosarcoma of the extremity: a joint study by the Italian and Scandinavian Sarcoma Groups. J Clin Oncol. 2005;23(34):8845-52.

1. Isakoff MS, Bielack SS, Meltzer P, Gorlick R. Osteosarcoma: Current Treatment and a Collaborative Pathway to Success. J Clin Oncol. 2015;33(27):3029-35.

2. Ferrari S, Smeland S, Mercuri M, Bertoni F, Longhi A, Ruggieri P, et al. Neoadjuvant chemotherapy with high-dose Ifosfamide, high-dose methotrexate, cisplatin, and doxorubicin for patients with localized osteosarcoma of the extremity: a joint study by the Italian and Scandinavian Sarcoma Groups. J Clin Oncol. 2005;23(34):8845-52.

3. Siegel RL, Miller KD, Jemal A. Cancer statistics, 2019. CA Cancer J Clin. 2019;69(1):7-34.

1. Isakoff MS, Bielack SS, Meltzer P, Gorlick R. Osteosarcoma: Current Treatment and a Collaborative Pathway to Success. J Clin Oncol. 2015;33(27):3029-35.

2. Ferrari S, Smeland S, Mercuri M, Bertoni F, Longhi A, Ruggieri P, et al. Neoadjuvant chemotherapy with high-dose Ifosfamide, high-dose methotrexate, cisplatin, and doxorubicin for patients with localized osteosarcoma of the extremity: a joint study by the Italian and Scandinavian Sarcoma Groups. J Clin Oncol. 2005;23(34):8845-52.

3. Siegel RL, Miller KD, Jemal A. Cancer statistics, 2019. CA Cancer J Clin. 2019;69(1):7-34.

4. Ritter J, Bielack SS. Osteosarcoma. Ann Oncol. 2010;21 Suppl 7:vii320-5.

1. Isakoff MS, Bielack SS, Meltzer P, Gorlick R. Osteosarcoma: Current Treatment and a Collaborative Pathway to Success. J Clin Oncol. 2015;33(27):3029-35.

2. Ferrari S, Smeland S, Mercuri M, Bertoni F, Longhi A, Ruggieri P, et al. Neoadjuvant chemotherapy with high-dose Ifosfamide, high-dose methotrexate, cisplatin, and doxorubicin for patients with localized osteosarcoma of the extremity: a joint study by the Italian and Scandinavian Sarcoma Groups. J Clin Oncol. 2005;23(34):8845-52.

3. Siegel RL, Miller KD, Jemal A. Cancer statistics, 2019. CA Cancer J Clin. 2019;69(1):7-34.

4. Ritter J, Bielack SS. Osteosarcoma. Ann Oncol. 2010;21 Suppl 7:vii320-5.

5. Gill J, Gorlick R. Advancing therapy for osteosarcoma. Nat Rev Clin Oncol. 2021;18(10):609-24.

1. Isakoff MS, Bielack SS, Meltzer P, Gorlick R. Osteosarcoma: Current Treatment and a Collaborative Pathway to Success. J Clin Oncol. 2015;33(27):3029-35.

2. Ferrari S, Smeland S, Mercuri M, Bertoni F, Longhi A, Ruggieri P, et al. Neoadjuvant chemotherapy with high-dose Ifosfamide, high-dose methotrexate, cisplatin, and doxorubicin for patients with localized osteosarcoma of the extremity: a joint study by the Italian and Scandinavian Sarcoma Groups. J Clin Oncol. 2005;23(34):8845-52.

3. Siegel RL, Miller KD, Jemal A. Cancer statistics, 2019. CA Cancer J Clin. 2019;69(1):7-34.

4. Ritter J, Bielack SS. Osteosarcoma. Ann Oncol. 2010;21 Suppl 7:vii320-5.

5. Gill J, Gorlick R. Advancing therapy for osteosarcoma. Nat Rev Clin Oncol. 2021;18(10):609-24.

6. Bielack SS, Kempf-Bielack B, Delling G, Exner GU, Flege S, Helmke K, et al. Prognostic factors in high-grade osteosarcoma of the extremities or trunk: an analysis of 1,702 patients treated on neoadjuvant cooperative osteosarcoma study group protocols. J Clin Oncol. 2002;20(3):776-90.

1. Isakoff MS, Bielack SS, Meltzer P, Gorlick R. Osteosarcoma: Current Treatment and a Collaborative Pathway to Success. J Clin Oncol. 2015;33(27):3029-35.

2. Ferrari S, Smeland S, Mercuri M, Bertoni F, Longhi A, Ruggieri P, et al. Neoadjuvant chemotherapy with high-dose Ifosfamide, high-dose methotrexate, cisplatin, and doxorubicin for patients with localized osteosarcoma of the extremity: a joint study by the Italian and Scandinavian Sarcoma Groups. J Clin Oncol. 2005;23(34):8845-52.

3. Siegel RL, Miller KD, Jemal A. Cancer statistics, 2019. CA Cancer J Clin. 2019;69(1):7-34.

4. Ritter J, Bielack SS. Osteosarcoma. Ann Oncol. 2010;21 Suppl 7:vii320-5.

5. Gill J, Gorlick R. Advancing therapy for osteosarcoma. Nat Rev Clin Oncol. 2021;18(10):609-24.

6. Bielack SS, Kempf-Bielack B, Delling G, Exner GU, Flege S, Helmke K, et al. Prognostic factors in high-grade osteosarcoma of the extremities or trunk: an analysis of 1,702 patients treated on neoadjuvant cooperative osteosarcoma study group protocols. J Clin Oncol. 2002;20(3):776-90.

1. Isakoff MS, Bielack SS, Meltzer P, Gorlick R. Osteosarcoma: Current Treatment and a Collaborative Pathway to Success. J Clin Oncol. 2015;33(27):3029-35.

2. Ferrari S, Smeland S, Mercuri M, Bertoni F, Longhi A, Ruggieri P, et al. Neoadjuvant chemotherapy with high-dose Ifosfamide, high-dose methotrexate, cisplatin, and doxorubicin for patients with localized osteosarcoma of the extremity: a joint study by the Italian and Scandinavian Sarcoma Groups. J Clin Oncol. 2005;23(34):8845-52.

3. Siegel RL, Miller KD, Jemal A. Cancer statistics, 2019. CA Cancer J Clin. 2019;69(1):7-34.

4. Ritter J, Bielack SS. Osteosarcoma. Ann Oncol. 2010;21 Suppl 7:vii320-5.

5. Gill J, Gorlick R. Advancing therapy for osteosarcoma. Nat Rev Clin Oncol. 2021;18(10):609-24.

6. Bielack SS, Kempf-Bielack B, Delling G, Exner GU, Flege S, Helmke K, et al. Prognostic factors in high-grade osteosarcoma of the extremities or trunk: an analysis of 1,702 patients treated on neoadjuvant cooperative osteosarcoma study group protocols. J Clin Oncol. 2002;20(3):776-90.

7. Han Z, Peng X, Yang Y, Yi J, Zhao D, Bao Q, et al. Integrated microfluidic-SERS for exosome biomarker profiling and osteosarcoma diagnosis. Biosens Bioelectron. 2022;217:114709.

8. Wu Y, Xu L, Yang P, Lin N, Huang X, Pan W, et al. Survival Prediction in High-grade Osteosarcoma Using Radiomics of Diagnostic Computed Tomography. EBioMedicine. 2018;34:27-34.

1. Isakoff MS, Bielack SS, Meltzer P, Gorlick R. Osteosarcoma: Current Treatment and a Collaborative Pathway to Success. J Clin Oncol. 2015;33(27):3029-35.

2. Ferrari S, Smeland S, Mercuri M, Bertoni F, Longhi A, Ruggieri P, et al. Neoadjuvant chemotherapy with high-dose Ifosfamide, high-dose methotrexate, cisplatin, and doxorubicin for patients with localized osteosarcoma of the extremity: a joint study by the Italian and Scandinavian Sarcoma Groups. J Clin Oncol. 2005;23(34):8845-52.

3. Siegel RL, Miller KD, Jemal A. Cancer statistics, 2019. CA Cancer J Clin. 2019;69(1):7-34.

4. Ritter J, Bielack SS. Osteosarcoma. Ann Oncol. 2010;21 Suppl 7:vii320-5.

5. Gill J, Gorlick R. Advancing therapy for osteosarcoma. Nat Rev Clin Oncol. 2021;18(10):609-24.

6. Bielack SS, Kempf-Bielack B, Delling G, Exner GU, Flege S, Helmke K, et al. Prognostic factors in high-grade osteosarcoma of the extremities or trunk: an analysis of 1,702 patients treated on neoadjuvant cooperative osteosarcoma study group protocols. J Clin Oncol. 2002;20(3):776-90.

7. Han Z, Peng X, Yang Y, Yi J, Zhao D, Bao Q, et al. Integrated microfluidic-SERS for exosome biomarker profiling and osteosarcoma diagnosis. Biosens Bioelectron. 2022;217:114709.

8. Wu Y, Xu L, Yang P, Lin N, Huang X, Pan W, et al. Survival Prediction in High-grade Osteosarcoma Using Radiomics of Diagnostic Computed Tomography. EBioMedicine. 2018;34:27-34.

1. Isakoff MS, Bielack SS, Meltzer P, Gorlick R. Osteosarcoma: Current Treatment and a Collaborative Pathway to Success. J Clin Oncol. 2015;33(27):3029-35.

2. Ferrari S, Smeland S, Mercuri M, Bertoni F, Longhi A, Ruggieri P, et al. Neoadjuvant chemotherapy with high-dose Ifosfamide, high-dose methotrexate, cisplatin, and doxorubicin for patients with localized osteosarcoma of the extremity: a joint study by the Italian and Scandinavian Sarcoma Groups. J Clin Oncol. 2005;23(34):8845-52.

3. Siegel RL, Miller KD, Jemal A. Cancer statistics, 2019. CA Cancer J Clin. 2019;69(1):7-34.

4. Ritter J, Bielack SS. Osteosarcoma. Ann Oncol. 2010;21 Suppl 7:vii320-5.

5. Gill J, Gorlick R. Advancing therapy for osteosarcoma. Nat Rev Clin Oncol. 2021;18(10):609-24.

6. Bielack SS, Kempf-Bielack B, Delling G, Exner GU, Flege S, Helmke K, et al. Prognostic factors in high-grade osteosarcoma of the extremities or trunk: an analysis of 1,702 patients treated on neoadjuvant cooperative osteosarcoma study group protocols. J Clin Oncol. 2002;20(3):776-90.

7. Han Z, Peng X, Yang Y, Yi J, Zhao D, Bao Q, et al. Integrated microfluidic-SERS for exosome biomarker profiling and osteosarcoma diagnosis. Biosens Bioelectron. 2022;217:114709.

8. Wu Y, Xu L, Yang P, Lin N, Huang X, Pan W, et al. Survival Prediction in High-grade Osteosarcoma Using Radiomics of Diagnostic Computed Tomography. EBioMedicine. 2018;34:27-34.

9. Chen W, Lin Y, Huang J, Yan Z, Cao H. A novel risk score model based on glycolysis-related genes and a prognostic model for predicting overall survival of osteosarcoma patients. J Orthop Res. 2022;40(10):2372-81.

1. Isakoff MS, Bielack SS, Meltzer P, Gorlick R. Osteosarcoma: Current Treatment and a Collaborative Pathway to Success. J Clin Oncol. 2015;33(27):3029-35.

2. Ferrari S, Smeland S, Mercuri M, Bertoni F, Longhi A, Ruggieri P, et al. Neoadjuvant chemotherapy with high-dose Ifosfamide, high-dose methotrexate, cisplatin, and doxorubicin for patients with localized osteosarcoma of the extremity: a joint study by the Italian and Scandinavian Sarcoma Groups. J Clin Oncol. 2005;23(34):8845-52.

3. Siegel RL, Miller KD, Jemal A. Cancer statistics, 2019. CA Cancer J Clin. 2019;69(1):7-34.

4. Ritter J, Bielack SS. Osteosarcoma. Ann Oncol. 2010;21 Suppl 7:vii320-5.

5. Gill J, Gorlick R. Advancing therapy for osteosarcoma. Nat Rev Clin Oncol. 2021;18(10):609-24.

6. Bielack SS, Kempf-Bielack B, Delling G, Exner GU, Flege S, Helmke K, et al. Prognostic factors in high-grade osteosarcoma of the extremities or trunk: an analysis of 1,702 patients treated on neoadjuvant cooperative osteosarcoma study group protocols. J Clin Oncol. 2002;20(3):776-90.

7. Han Z, Peng X, Yang Y, Yi J, Zhao D, Bao Q, et al. Integrated microfluidic-SERS for exosome biomarker profiling and osteosarcoma diagnosis. Biosens Bioelectron. 2022;217:114709.

8. Wu Y, Xu L, Yang P, Lin N, Huang X, Pan W, et al. Survival Prediction in High-grade Osteosarcoma Using Radiomics of Diagnostic Computed Tomography. EBioMedicine. 2018;34:27-34.

9. Chen W, Lin Y, Huang J, Yan Z, Cao H. A novel risk score model based on glycolysis-related genes and a prognostic model for predicting overall survival of osteosarcoma patients. J Orthop Res. 2022;40(10):2372-81.

10. Zhang M, Liu Y, Kong D. Identifying biomolecules and constructing a prognostic risk prediction model for recurrence in osteosarcoma. J Bone Oncol. 2021;26:100331.

1. Isakoff MS, Bielack SS, Meltzer P, Gorlick R. Osteosarcoma: Current Treatment and a Collaborative Pathway to Success. J Clin Oncol. 2015;33(27):3029-35.

2. Ferrari S, Smeland S, Mercuri M, Bertoni F, Longhi A, Ruggieri P, et al. Neoadjuvant chemotherapy with high-dose Ifosfamide, high-dose methotrexate, cisplatin, and doxorubicin for patients with localized osteosarcoma of the extremity: a joint study by the Italian and Scandinavian Sarcoma Groups. J Clin Oncol. 2005;23(34):8845-52.

3. Siegel RL, Miller KD, Jemal A. Cancer statistics, 2019. CA Cancer J Clin. 2019;69(1):7-34.

4. Ritter J, Bielack SS. Osteosarcoma. Ann Oncol. 2010;21 Suppl 7:vii320-5.

5. Gill J, Gorlick R. Advancing therapy for osteosarcoma. Nat Rev Clin Oncol. 2021;18(10):609-24.

6. Bielack SS, Kempf-Bielack B, Delling G, Exner GU, Flege S, Helmke K, et al. Prognostic factors in high-grade osteosarcoma of the extremities or trunk: an analysis of 1,702 patients treated on neoadjuvant cooperative osteosarcoma study group protocols. J Clin Oncol. 2002;20(3):776-90.

7. Han Z, Peng X, Yang Y, Yi J, Zhao D, Bao Q, et al. Integrated microfluidic-SERS for exosome biomarker profiling and osteosarcoma diagnosis. Biosens Bioelectron. 2022;217:114709.

8. Wu Y, Xu L, Yang P, Lin N, Huang X, Pan W, et al. Survival Prediction in High-grade Osteosarcoma Using Radiomics of Diagnostic Computed Tomography. EBioMedicine. 2018;34:27-34.

9. Chen W, Lin Y, Huang J, Yan Z, Cao H. A novel risk score model based on glycolysis-related genes and a prognostic model for predicting overall survival of osteosarcoma patients. J Orthop Res. 2022;40(10):2372-81.

10. Zhang M, Liu Y, Kong D. Identifying biomolecules and constructing a prognostic risk prediction model for recurrence in osteosarcoma. J Bone Oncol. 2021;26:100331.

11. Li L, Wang Y, He X, Li Z, Lu M, Gong T, et al. Hematological Prognostic Scoring System Can Predict Overall Survival and Can Indicate Response to Immunotherapy in Patients With Osteosarcoma. Front Immunol. 2022;13:879560.

1. Isakoff MS, Bielack SS, Meltzer P, Gorlick R. Osteosarcoma: Current Treatment and a Collaborative Pathway to Success. J Clin Oncol. 2015;33(27):3029-35.

2. Ferrari S, Smeland S, Mercuri M, Bertoni F, Longhi A, Ruggieri P, et al. Neoadjuvant chemotherapy with high-dose Ifosfamide, high-dose methotrexate, cisplatin, and doxorubicin for patients with localized osteosarcoma of the extremity: a joint study by the Italian and Scandinavian Sarcoma Groups. J Clin Oncol. 2005;23(34):8845-52.

3. Siegel RL, Miller KD, Jemal A. Cancer statistics, 2019. CA Cancer J Clin. 2019;69(1):7-34.

4. Ritter J, Bielack SS. Osteosarcoma. Ann Oncol. 2010;21 Suppl 7:vii320-5.

5. Gill J, Gorlick R. Advancing therapy for osteosarcoma. Nat Rev Clin Oncol. 2021;18(10):609-24.

6. Bielack SS, Kempf-Bielack B, Delling G, Exner GU, Flege S, Helmke K, et al. Prognostic factors in high-grade osteosarcoma of the extremities or trunk: an analysis of 1,702 patients treated on neoadjuvant cooperative osteosarcoma study group protocols. J Clin Oncol. 2002;20(3):776-90.

7. Han Z, Peng X, Yang Y, Yi J, Zhao D, Bao Q, et al. Integrated microfluidic-SERS for exosome biomarker profiling and osteosarcoma diagnosis. Biosens Bioelectron. 2022;217:114709.

8. Wu Y, Xu L, Yang P, Lin N, Huang X, Pan W, et al. Survival Prediction in High-grade Osteosarcoma Using Radiomics of Diagnostic Computed Tomography. EBioMedicine. 2018;34:27-34.

9. Chen W, Lin Y, Huang J, Yan Z, Cao H. A novel risk score model based on glycolysis-related genes and a prognostic model for predicting overall survival of osteosarcoma patients. J Orthop Res. 2022;40(10):2372-81.

10. Zhang M, Liu Y, Kong D. Identifying biomolecules and constructing a prognostic risk prediction model for recurrence in osteosarcoma. J Bone Oncol. 2021;26:100331.

11. Li L, Wang Y, He X, Li Z, Lu M, Gong T, et al. Hematological Prognostic Scoring System Can Predict Overall Survival and Can Indicate Response to Immunotherapy in Patients With Osteosarcoma. Front Immunol. 2022;13:879560.

12. Pelish HE, Liau BB, Nitulescu, II, Tangpeerachaikul A, Poss ZC, Da Silva DH, et al. Mediator kinase inhibition further activates super-enhancer-associated genes in AML. Nature. 2015;526(7572):273-6.

1. Isakoff MS, Bielack SS, Meltzer P, Gorlick R. Osteosarcoma: Current Treatment and a Collaborative Pathway to Success. J Clin Oncol. 2015;33(27):3029-35.

2. Ferrari S, Smeland S, Mercuri M, Bertoni F, Longhi A, Ruggieri P, et al. Neoadjuvant chemotherapy with high-dose Ifosfamide, high-dose methotrexate, cisplatin, and doxorubicin for patients with localized osteosarcoma of the extremity: a joint study by the Italian and Scandinavian Sarcoma Groups. J Clin Oncol. 2005;23(34):8845-52.

3. Siegel RL, Miller KD, Jemal A. Cancer statistics, 2019. CA Cancer J Clin. 2019;69(1):7-34.

4. Ritter J, Bielack SS. Osteosarcoma. Ann Oncol. 2010;21 Suppl 7:vii320-5.

5. Gill J, Gorlick R. Advancing therapy for osteosarcoma. Nat Rev Clin Oncol. 2021;18(10):609-24.

6. Bielack SS, Kempf-Bielack B, Delling G, Exner GU, Flege S, Helmke K, et al. Prognostic factors in high-grade osteosarcoma of the extremities or trunk: an analysis of 1,702 patients treated on neoadjuvant cooperative osteosarcoma study group protocols. J Clin Oncol. 2002;20(3):776-90.

7. Han Z, Peng X, Yang Y, Yi J, Zhao D, Bao Q, et al. Integrated microfluidic-SERS for exosome biomarker profiling and osteosarcoma diagnosis. Biosens Bioelectron. 2022;217:114709.

8. Wu Y, Xu L, Yang P, Lin N, Huang X, Pan W, et al. Survival Prediction in High-grade Osteosarcoma Using Radiomics of Diagnostic Computed Tomography. EBioMedicine. 2018;34:27-34.

9. Chen W, Lin Y, Huang J, Yan Z, Cao H. A novel risk score model based on glycolysis-related genes and a prognostic model for predicting overall survival of osteosarcoma patients. J Orthop Res. 2022;40(10):2372-81.

10. Zhang M, Liu Y, Kong D. Identifying biomolecules and constructing a prognostic risk prediction model for recurrence in osteosarcoma. J Bone Oncol. 2021;26:100331.

11. Li L, Wang Y, He X, Li Z, Lu M, Gong T, et al. Hematological Prognostic Scoring System Can Predict Overall Survival and Can Indicate Response to Immunotherapy in Patients With Osteosarcoma. Front Immunol. 2022;13:879560.

12. Pelish HE, Liau BB, Nitulescu, II, Tangpeerachaikul A, Poss ZC, Da Silva DH, et al. Mediator kinase inhibition further activates super-enhancer-associated genes in AML. Nature. 2015;526(7572):273-6.

13. Whyte WA, Orlando DA, Hnisz D, Abraham BJ, Lin CY, Kagey MH, et al. Master transcription factors and mediator establish super-enhancers at key cell identity genes. Cell. 2013;153(2):307-19.

1. Isakoff MS, Bielack SS, Meltzer P, Gorlick R. Osteosarcoma: Current Treatment and a Collaborative Pathway to Success. J Clin Oncol. 2015;33(27):3029-35.

2. Ferrari S, Smeland S, Mercuri M, Bertoni F, Longhi A, Ruggieri P, et al. Neoadjuvant chemotherapy with high-dose Ifosfamide, high-dose methotrexate, cisplatin, and doxorubicin for patients with localized osteosarcoma of the extremity: a joint study by the Italian and Scandinavian Sarcoma Groups. J Clin Oncol. 2005;23(34):8845-52.

3. Siegel RL, Miller KD, Jemal A. Cancer statistics, 2019. CA Cancer J Clin. 2019;69(1):7-34.

4. Ritter J, Bielack SS. Osteosarcoma. Ann Oncol. 2010;21 Suppl 7:vii320-5.

5. Gill J, Gorlick R. Advancing therapy for osteosarcoma. Nat Rev Clin Oncol. 2021;18(10):609-24.

6. Bielack SS, Kempf-Bielack B, Delling G, Exner GU, Flege S, Helmke K, et al. Prognostic factors in high-grade osteosarcoma of the extremities or trunk: an analysis of 1,702 patients treated on neoadjuvant cooperative osteosarcoma study group protocols. J Clin Oncol. 2002;20(3):776-90.

7. Han Z, Peng X, Yang Y, Yi J, Zhao D, Bao Q, et al. Integrated microfluidic-SERS for exosome biomarker profiling and osteosarcoma diagnosis. Biosens Bioelectron. 2022;217:114709.

8. Wu Y, Xu L, Yang P, Lin N, Huang X, Pan W, et al. Survival Prediction in High-grade Osteosarcoma Using Radiomics of Diagnostic Computed Tomography. EBioMedicine. 2018;34:27-34.

9. Chen W, Lin Y, Huang J, Yan Z, Cao H. A novel risk score model based on glycolysis-related genes and a prognostic model for predicting overall survival of osteosarcoma patients. J Orthop Res. 2022;40(10):2372-81.

10. Zhang M, Liu Y, Kong D. Identifying biomolecules and constructing a prognostic risk prediction model for recurrence in osteosarcoma. J Bone Oncol. 2021;26:100331.

11. Li L, Wang Y, He X, Li Z, Lu M, Gong T, et al. Hematological Prognostic Scoring System Can Predict Overall Survival and Can Indicate Response to Immunotherapy in Patients With Osteosarcoma. Front Immunol. 2022;13:879560.

12. Pelish HE, Liau BB, Nitulescu, II, Tangpeerachaikul A, Poss ZC, Da Silva DH, et al. Mediator kinase inhibition further activates super-enhancer-associated genes in AML. Nature. 2015;526(7572):273-6.

13. Whyte WA, Orlando DA, Hnisz D, Abraham BJ, Lin CY, Kagey MH, et al. Master transcription factors and mediator establish super-enhancers at key cell identity genes. Cell. 2013;153(2):307-19.

14. Tögel L, Nightingale R, Chueh AC, Jayachandran A, Tran H, Phesse T, et al. Dual Targeting of Bromodomain and Extraterminal Domain Proteins, and WNT or MAPK Signaling, Inhibits c-MYC Expression and Proliferation of Colorectal Cancer Cells. Mol Cancer Ther. 2016;15(6):1217-26.

1. Isakoff MS, Bielack SS, Meltzer P, Gorlick R. Osteosarcoma: Current Treatment and a Collaborative Pathway to Success. J Clin Oncol. 2015;33(27):3029-35.

2. Ferrari S, Smeland S, Mercuri M, Bertoni F, Longhi A, Ruggieri P, et al. Neoadjuvant chemotherapy with high-dose Ifosfamide, high-dose methotrexate, cisplatin, and doxorubicin for patients with localized osteosarcoma of the extremity: a joint study by the Italian and Scandinavian Sarcoma Groups. J Clin Oncol. 2005;23(34):8845-52.

3. Siegel RL, Miller KD, Jemal A. Cancer statistics, 2019. CA Cancer J Clin. 2019;69(1):7-34.

4. Ritter J, Bielack SS. Osteosarcoma. Ann Oncol. 2010;21 Suppl 7:vii320-5.

5. Gill J, Gorlick R. Advancing therapy for osteosarcoma. Nat Rev Clin Oncol. 2021;18(10):609-24.

6. Bielack SS, Kempf-Bielack B, Delling G, Exner GU, Flege S, Helmke K, et al. Prognostic factors in high-grade osteosarcoma of the extremities or trunk: an analysis of 1,702 patients treated on neoadjuvant cooperative osteosarcoma study group protocols. J Clin Oncol. 2002;20(3):776-90.

7. Han Z, Peng X, Yang Y, Yi J, Zhao D, Bao Q, et al. Integrated microfluidic-SERS for exosome biomarker profiling and osteosarcoma diagnosis. Biosens Bioelectron. 2022;217:114709.

8. Wu Y, Xu L, Yang P, Lin N, Huang X, Pan W, et al. Survival Prediction in High-grade Osteosarcoma Using Radiomics of Diagnostic Computed Tomography. EBioMedicine. 2018;34:27-34.

9. Chen W, Lin Y, Huang J, Yan Z, Cao H. A novel risk score model based on glycolysis-related genes and a prognostic model for predicting overall survival of osteosarcoma patients. J Orthop Res. 2022;40(10):2372-81.

10. Zhang M, Liu Y, Kong D. Identifying biomolecules and constructing a prognostic risk prediction model for recurrence in osteosarcoma. J Bone Oncol. 2021;26:100331.

11. Li L, Wang Y, He X, Li Z, Lu M, Gong T, et al. Hematological Prognostic Scoring System Can Predict Overall Survival and Can Indicate Response to Immunotherapy in Patients With Osteosarcoma. Front Immunol. 2022;13:879560.

12. Pelish HE, Liau BB, Nitulescu, II, Tangpeerachaikul A, Poss ZC, Da Silva DH, et al. Mediator kinase inhibition further activates super-enhancer-associated genes in AML. Nature. 2015;526(7572):273-6.

13. Whyte WA, Orlando DA, Hnisz D, Abraham BJ, Lin CY, Kagey MH, et al. Master transcription factors and mediator establish super-enhancers at key cell identity genes. Cell. 2013;153(2):307-19.

14. Tögel L, Nightingale R, Chueh AC, Jayachandran A, Tran H, Phesse T, et al. Dual Targeting of Bromodomain and Extraterminal Domain Proteins, and WNT or MAPK Signaling, Inhibits c-MYC Expression and Proliferation of Colorectal Cancer Cells. Mol Cancer Ther. 2016;15(6):1217-26.

15. Mansour MR, Abraham BJ, Anders L, Berezovskaya A, Gutierrez A, Durbin AD, et al. Oncogene regulation. An oncogenic super-enhancer formed through somatic mutation of a noncoding intergenic element. Science. 2014;346(6215):1373-7.

1. Isakoff MS, Bielack SS, Meltzer P, Gorlick R. Osteosarcoma: Current Treatment and a Collaborative Pathway to Success. J Clin Oncol. 2015;33(27):3029-35.

2. Ferrari S, Smeland S, Mercuri M, Bertoni F, Longhi A, Ruggieri P, et al. Neoadjuvant chemotherapy with high-dose Ifosfamide, high-dose methotrexate, cisplatin, and doxorubicin for patients with localized osteosarcoma of the extremity: a joint study by the Italian and Scandinavian Sarcoma Groups. J Clin Oncol. 2005;23(34):8845-52.

3. Siegel RL, Miller KD, Jemal A. Cancer statistics, 2019. CA Cancer J Clin. 2019;69(1):7-34.

4. Ritter J, Bielack SS. Osteosarcoma. Ann Oncol. 2010;21 Suppl 7:vii320-5.

5. Gill J, Gorlick R. Advancing therapy for osteosarcoma. Nat Rev Clin Oncol. 2021;18(10):609-24.

6. Bielack SS, Kempf-Bielack B, Delling G, Exner GU, Flege S, Helmke K, et al. Prognostic factors in high-grade osteosarcoma of the extremities or trunk: an analysis of 1,702 patients treated on neoadjuvant cooperative osteosarcoma study group protocols. J Clin Oncol. 2002;20(3):776-90.

7. Han Z, Peng X, Yang Y, Yi J, Zhao D, Bao Q, et al. Integrated microfluidic-SERS for exosome biomarker profiling and osteosarcoma diagnosis. Biosens Bioelectron. 2022;217:114709.

8. Wu Y, Xu L, Yang P, Lin N, Huang X, Pan W, et al. Survival Prediction in High-grade Osteosarcoma Using Radiomics of Diagnostic Computed Tomography. EBioMedicine. 2018;34:27-34.

9. Chen W, Lin Y, Huang J, Yan Z, Cao H. A novel risk score model based on glycolysis-related genes and a prognostic model for predicting overall survival of osteosarcoma patients. J Orthop Res. 2022;40(10):2372-81.

11. Li L, Wang Y, He X, Li Z, Lu M, Gong T, et al. Hematological Prognostic Scoring System Can Predict Overall Survival and Can Indicate Response to Immunotherapy in Patients With Osteosarcoma. Front Immunol. 2022;13:879560.

12. Pelish HE, Liau BB, Nitulescu, II, Tangpeerachaikul A, Poss ZC, Da Silva DH, et al. Mediator kinase inhibition further activates super-enhancer-associated genes in AML. Nature. 2015;526(7572):273-6.

13. Whyte WA, Orlando DA, Hnisz D, Abraham BJ, Lin CY, Kagey MH, et al. Master transcription factors and mediator establish super-enhancers at key cell identity genes. Cell. 2013;153(2):307-19.

14. Tögel L, Nightingale R, Chueh AC, Jayachandran A, Tran H, Phesse T, et al. Dual Targeting of Bromodomain and Extraterminal Domain Proteins, and WNT or MAPK Signaling, Inhibits c-MYC Expression and Proliferation of Colorectal Cancer Cells. Mol Cancer Ther. 2016;15(6):1217-26.

15. Mansour MR, Abraham BJ, Anders L, Berezovskaya A, Gutierrez A, Durbin AD, et al. Oncogene regulation. An oncogenic super-enhancer formed through somatic mutation of a noncoding intergenic element. Science. 2014;346(6215):1373-7.

16. Lovén J, Hoke HA, Lin CY, Lau A, Orlando DA, Vakoc CR, et al. Selective inhibition of tumor oncogenes by disruption of super-enhancers. Cell. 2013;153(2):320-34.

1. Isakoff MS, Bielack SS, Meltzer P, Gorlick R. Osteosarcoma: Current Treatment and a Collaborative Pathway to Success. J Clin Oncol. 2015;33(27):3029-35.

2. Ferrari S, Smeland S, Mercuri M, Bertoni F, Longhi A, Ruggieri P, et al. Neoadjuvant chemotherapy with high-dose Ifosfamide, high-dose methotrexate, cisplatin, and doxorubicin for patients with localized osteosarcoma of the extremity: a joint study by the Italian and Scandinavian Sarcoma Groups. J Clin Oncol. 2005;23(34):8845-52.

3. Siegel RL, Miller KD, Jemal A. Cancer statistics, 2019. CA Cancer J Clin. 2019;69(1):7-34.

4. Ritter J, Bielack SS. Osteosarcoma. Ann Oncol. 2010;21 Suppl 7:vii320-5.

5. Gill J, Gorlick R. Advancing therapy for osteosarcoma. Nat Rev Clin Oncol. 2021;18(10):609-24.

6. Bielack SS, Kempf-Bielack B, Delling G, Exner GU, Flege S, Helmke K, et al. Prognostic factors in high-grade osteosarcoma of the extremities or trunk: an analysis of 1,702 patients treated on neoadjuvant cooperative osteosarcoma study group protocols. J Clin Oncol. 2002;20(3):776-90.

7. Han Z, Peng X, Yang Y, Yi J, Zhao D, Bao Q, et al. Integrated microfluidic-SERS for exosome biomarker profiling and osteosarcoma diagnosis. Biosens Bioelectron. 2022;217:114709.

8. Wu Y, Xu L, Yang P, Lin N, Huang X, Pan W, et al. Survival Prediction in High-grade Osteosarcoma Using Radiomics of Diagnostic Computed Tomography. EBioMedicine. 2018;34:27-34.

9. Chen W, Lin Y, Huang J, Yan Z, Cao H. A novel risk score model based on glycolysis-related genes and a prognostic model for predicting overall survival of osteosarcoma patients. J Orthop Res. 2022;40(10):2372-81.

10. Zhang M, Liu Y, Kong D. Identifying biomolecules and constructing a prognostic risk prediction model for recurrence in osteosarcoma. J Bone Oncol. 2021;26:100331.

11. Li L, Wang Y, He X, Li Z, Lu M, Gong T, et al. Hematological Prognostic Scoring System Can Predict Overall Survival and Can Indicate Response to Immunotherapy in Patients With Osteosarcoma. Front Immunol. 2022;13:879560.

12. Pelish HE, Liau BB, Nitulescu, II, Tangpeerachaikul A, Poss ZC, Da Silva DH, et al. Mediator kinase inhibition further activates super-enhancer-associated genes in AML. Nature. 2015;526(7572):273-6.

13. Whyte WA, Orlando DA, Hnisz D, Abraham BJ, Lin CY, Kagey MH, et al. Master transcription factors and mediator establish super-enhancers at key cell identity genes. Cell. 2013;153(2):307-19.

14. Tögel L, Nightingale R, Chueh AC, Jayachandran A, Tran H, Phesse T, et al. Dual Targeting of Bromodomain and Extraterminal Domain Proteins, and WNT or MAPK Signaling, Inhibits c-MYC Expression and Proliferation of Colorectal Cancer Cells. Mol Cancer Ther. 2016;15(6):1217-26.

15. Mansour MR, Abraham BJ, Anders L, Berezovskaya A, Gutierrez A, Durbin AD, et al. Oncogene regulation. An oncogenic super-enhancer formed through somatic mutation of a noncoding intergenic element. Science. 2014;346(6215):1373-7.

16. Lovén J, Hoke HA, Lin CY, Lau A, Orlando DA, Vakoc CR, et al. Selective inhibition of tumor oncogenes by disruption of super-enhancers. Cell. 2013;153(2):320-34.

17. Wang Y, Zhang T, Kwiatkowski N, Abraham BJ, Lee TI, Xie S, et al. CDK7-dependent transcriptional addiction in triple-negative breast cancer. Cell. 2015;163(1):174-86.

1. Isakoff MS, Bielack SS, Meltzer P, Gorlick R. Osteosarcoma: Current Treatment and a Collaborative Pathway to Success. J Clin Oncol. 2015;33(27):3029-35.

2. Ferrari S, Smeland S, Mercuri M, Bertoni F, Longhi A, Ruggieri P, et al. Neoadjuvant chemotherapy with high-dose Ifosfamide, high-dose methotrexate, cisplatin, and doxorubicin for patients with localized osteosarcoma of the extremity: a joint study by the Italian and Scandinavian Sarcoma Groups. J Clin Oncol. 2005;23(34):8845-52.

3. Siegel RL, Miller KD, Jemal A. Cancer statistics, 2019. CA Cancer J Clin. 2019;69(1):7-34.

4. Ritter J, Bielack SS. Osteosarcoma. Ann Oncol. 2010;21 Suppl 7:vii320-5.

5. Gill J, Gorlick R. Advancing therapy for osteosarcoma. Nat Rev Clin Oncol. 2021;18(10):609-24.

6. Bielack SS, Kempf-Bielack B, Delling G, Exner GU, Flege S, Helmke K, et al. Prognostic factors in high-grade osteosarcoma of the extremities or trunk: an analysis of 1,702 patients treated on neoadjuvant cooperative osteosarcoma study group protocols. J Clin Oncol. 2002;20(3):776-90.

7. Han Z, Peng X, Yang Y, Yi J, Zhao D, Bao Q, et al. Integrated microfluidic-SERS for exosome biomarker profiling and osteosarcoma diagnosis. Biosens Bioelectron. 2022;217:114709.

8. Wu Y, Xu L, Yang P, Lin N, Huang X, Pan W, et al. Survival Prediction in High-grade Osteosarcoma Using Radiomics of Diagnostic Computed Tomography. EBioMedicine. 2018;34:27-34.

9. Chen W, Lin Y, Huang J, Yan Z, Cao H. A novel risk score model based on glycolysis-related genes and a prognostic model for predicting overall survival of osteosarcoma patients. J Orthop Res. 2022;40(10):2372-81.

10. Zhang M, Liu Y, Kong D. Identifying biomolecules and constructing a prognostic risk prediction model for recurrence in osteosarcoma. J Bone Oncol. 2021;26:100331.

11. Li L, Wang Y, He X, Li Z, Lu M, Gong T, et al. Hematological Prognostic Scoring System Can Predict Overall Survival and Can Indicate Response to Immunotherapy in Patients With Osteosarcoma. Front Immunol. 2022;13:879560.

12. Pelish HE, Liau BB, Nitulescu, II, Tangpeerachaikul A, Poss ZC, Da Silva DH, et al. Mediator kinase inhibition further activates super-enhancer-associated genes in AML. Nature. 2015;526(7572):273-6.

13. Whyte WA, Orlando DA, Hnisz D, Abraham BJ, Lin CY, Kagey MH, et al. Master transcription factors and mediator establish super-enhancers at key cell identity genes. Cell. 2013;153(2):307-19.

14. Tögel L, Nightingale R, Chueh AC, Jayachandran A, Tran H, Phesse T, et al. Dual Targeting of Bromodomain and Extraterminal Domain Proteins, and WNT or MAPK Signaling, Inhibits c-MYC Expression and Proliferation of Colorectal Cancer Cells. Mol Cancer Ther. 2016;15(6):1217-26.

15. Mansour MR, Abraham BJ, Anders L, Berezovskaya A, Gutierrez A, Durbin AD, et al. Oncogene regulation. An oncogenic super-enhancer formed through somatic mutation of a noncoding intergenic element. Science. 2014;346(6215):1373-7.

16. Lovén J, Hoke HA, Lin CY, Lau A, Orlando DA, Vakoc CR, et al. Selective inhibition of tumor oncogenes by disruption of super-enhancers. Cell. 2013;153(2):320-34.

17. Wang Y, Zhang T, Kwiatkowski N, Abraham BJ, Lee TI, Xie S, et al. CDK7-dependent transcriptional addiction in triple-negative breast cancer. Cell. 2015;163(1):174-86.

18. Zhang J, Liu W, Zou C, Zhao Z, Lai Y, Shi Z, et al. Targeting Super-Enhancer-Associated Oncogenes in Osteosarcoma with THZ2, a Covalent CDK7 Inhibitor. Clin Cancer Res. 2020;26(11):2681-92.

1. Isakoff MS, Bielack SS, Meltzer P, Gorlick R. Osteosarcoma: Current Treatment and a Collaborative Pathway to Success. J Clin Oncol. 2015;33(27):3029-35.

2. Ferrari S, Smeland S, Mercuri M, Bertoni F, Longhi A, Ruggieri P, et al. Neoadjuvant chemotherapy with high-dose Ifosfamide, high-dose methotrexate, cisplatin, and doxorubicin for patients with localized osteosarcoma of the extremity: a joint study by the Italian and Scandinavian Sarcoma Groups. J Clin Oncol. 2005;23(34):8845-52.

3. Siegel RL, Miller KD, Jemal A. Cancer statistics, 2019. CA Cancer J Clin. 2019;69(1):7-34.

4. Ritter J, Bielack SS. Osteosarcoma. Ann Oncol. 2010;21 Suppl 7:vii320-5.

5. Gill J, Gorlick R. Advancing therapy for osteosarcoma. Nat Rev Clin Oncol. 2021;18(10):609-24.

6. Bielack SS, Kempf-Bielack B, Delling G, Exner GU, Flege S, Helmke K, et al. Prognostic factors in high-grade osteosarcoma of the extremities or trunk: an analysis of 1,702 patients treated on neoadjuvant cooperative osteosarcoma study group protocols. J Clin Oncol. 2002;20(3):776-90.

7. Han Z, Peng X, Yang Y, Yi J, Zhao D, Bao Q, et al. Integrated microfluidic-SERS for exosome biomarker profiling and osteosarcoma diagnosis. Biosens Bioelectron. 2022;217:114709.

8. Wu Y, Xu L, Yang P, Lin N, Huang X, Pan W, et al. Survival Prediction in High-grade Osteosarcoma Using Radiomics of Diagnostic Computed Tomography. EBioMedicine. 2018;34:27-34.

9. Chen W, Lin Y, Huang J, Yan Z, Cao H. A novel risk score model based on glycolysis-related genes and a prognostic model for predicting overall survival of osteosarcoma patients. J Orthop Res. 2022;40(10):2372-81.

10. Zhang M, Liu Y, Kong D. Identifying biomolecules and constructing a prognostic risk prediction model for recurrence in osteosarcoma. J Bone Oncol. 2021;26:100331.

11. Li L, Wang Y, He X, Li Z, Lu M, Gong T, et al. Hematological Prognostic Scoring System Can Predict Overall Survival and Can Indicate Response to Immunotherapy in Patients With Osteosarcoma. Front Immunol. 2022;13:879560.

12. Pelish HE, Liau BB, Nitulescu, II, Tangpeerachaikul A, Poss ZC, Da Silva DH, et al. Mediator kinase inhibition further activates super-enhancer-associated genes in AML. Nature. 2015;526(7572):273-6.

13. Whyte WA, Orlando DA, Hnisz D, Abraham BJ, Lin CY, Kagey MH, et al. Master transcription factors and mediator establish super-enhancers at key cell identity genes. Cell. 2013;153(2):307-19.

14. Tögel L, Nightingale R, Chueh AC, Jayachandran A, Tran H, Phesse T, et al. Dual Targeting of Bromodomain and Extraterminal Domain Proteins, and WNT or MAPK Signaling, Inhibits c-MYC Expression and Proliferation of Colorectal Cancer Cells. Mol Cancer Ther. 2016;15(6):1217-26.

15. Mansour MR, Abraham BJ, Anders L, Berezovskaya A, Gutierrez A, Durbin AD, et al. Oncogene regulation. An oncogenic super-enhancer formed through somatic mutation of a noncoding intergenic element. Science. 2014;346(6215):1373-7.

16. Lovén J, Hoke HA, Lin CY, Lau A, Orlando DA, Vakoc CR, et al. Selective inhibition of tumor oncogenes by disruption of super-enhancers. Cell. 2013;153(2):320-34.

17. Wang Y, Zhang T, Kwiatkowski N, Abraham BJ, Lee TI, Xie S, et al. CDK7-dependent transcriptional addiction in triple-negative breast cancer. Cell. 2015;163(1):174-86.

18. Zhang J, Liu W, Zou C, Zhao Z, Lai Y, Shi Z, et al. Targeting Super-Enhancer-Associated Oncogenes in Osteosarcoma with THZ2, a Covalent CDK7 Inhibitor. Clin Cancer Res. 2020;26(11):2681-92.

1. Isakoff MS, Bielack SS, Meltzer P, Gorlick R. Osteosarcoma: Current Treatment and a Collaborative Pathway to Success. J Clin Oncol. 2015;33(27):3029-35.

2. Ferrari S, Smeland S, Mercuri M, Bertoni F, Longhi A, Ruggieri P, et al. Neoadjuvant chemotherapy with high-dose Ifosfamide, high-dose methotrexate, cisplatin, and doxorubicin for patients with localized osteosarcoma of the extremity: a joint study by the Italian and Scandinavian Sarcoma Groups. J Clin Oncol. 2005;23(34):8845-52.

3. Siegel RL, Miller KD, Jemal A. Cancer statistics, 2019. CA Cancer J Clin. 2019;69(1):7-34.

4. Ritter J, Bielack SS. Osteosarcoma. Ann Oncol. 2010;21 Suppl 7:vii320-5.

5. Gill J, Gorlick R. Advancing therapy for osteosarcoma. Nat Rev Clin Oncol. 2021;18(10):609-24.

6. Bielack SS, Kempf-Bielack B, Delling G, Exner GU, Flege S, Helmke K, et al. Prognostic factors in high-grade osteosarcoma of the extremities or trunk: an analysis of 1,702 patients treated on neoadjuvant cooperative osteosarcoma study group protocols. J Clin Oncol. 2002;20(3):776-90.

7. Han Z, Peng X, Yang Y, Yi J, Zhao D, Bao Q, et al. Integrated microfluidic-SERS for exosome biomarker profiling and osteosarcoma diagnosis. Biosens Bioelectron. 2022;217:114709.

8. Wu Y, Xu L, Yang P, Lin N, Huang X, Pan W, et al. Survival Prediction in High-grade Osteosarcoma Using Radiomics of Diagnostic Computed Tomography. EBioMedicine. 2018;34:27-34.

9. Chen W, Lin Y, Huang J, Yan Z, Cao H. A novel risk score model based on glycolysis-related genes and a prognostic model for predicting overall survival of osteosarcoma patients. J Orthop Res. 2022;40(10):2372-81.

10. Zhang M, Liu Y, Kong D. Identifying biomolecules and constructing a prognostic risk prediction model for recurrence in osteosarcoma. J Bone Oncol. 2021;26:100331.

11. Li L, Wang Y, He X, Li Z, Lu M, Gong T, et al. Hematological Prognostic Scoring System Can Predict Overall Survival and Can Indicate Response to Immunotherapy in Patients With Osteosarcoma. Front Immunol. 2022;13:879560.

12. Pelish HE, Liau BB, Nitulescu, II, Tangpeerachaikul A, Poss ZC, Da Silva DH, et al. Mediator kinase inhibition further activates super-enhancer-associated genes in AML. Nature. 2015;526(7572):273-6.

13. Whyte WA, Orlando DA, Hnisz D, Abraham BJ, Lin CY, Kagey MH, et al. Master transcription factors and mediator establish super-enhancers at key cell identity genes. Cell. 2013;153(2):307-19.

14. Tögel L, Nightingale R, Chueh AC, Jayachandran A, Tran H, Phesse T, et al. Dual Targeting of Bromodomain and Extraterminal Domain Proteins, and WNT or MAPK Signaling, Inhibits c-MYC Expression and Proliferation of Colorectal Cancer Cells. Mol Cancer Ther. 2016;15(6):1217-26.

15. Mansour MR, Abraham BJ, Anders L, Berezovskaya A, Gutierrez A, Durbin AD, et al. Oncogene regulation. An oncogenic super-enhancer formed through somatic mutation of a noncoding intergenic element. Science. 2014;346(6215):1373-7.

16. Lovén J, Hoke HA, Lin CY, Lau A, Orlando DA, Vakoc CR, et al. Selective inhibition of tumor oncogenes by disruption of super-enhancers. Cell. 2013;153(2):320-34.

17. Wang Y, Zhang T, Kwiatkowski N, Abraham BJ, Lee TI, Xie S, et al. CDK7-dependent transcriptional addiction in triple-negative breast cancer. Cell. 2015;163(1):174-86.

18. Zhang J, Liu W, Zou C, Zhao Z, Lai Y, Shi Z, et al. Targeting Super-Enhancer-Associated Oncogenes in Osteosarcoma with THZ2, a Covalent CDK7 Inhibitor. Clin Cancer Res. 2020;26(11):2681-92.

19. Xing R, Zhou Y, Yu J, Yu Y, Nie Y, Luo W, et al. Whole-genome sequencing reveals novel tandem-duplication hotspots and a prognostic mutational signature in gastric cancer. Nat Commun. 2019;10(1):2037.

1. Isakoff MS, Bielack SS, Meltzer P, Gorlick R. Osteosarcoma: Current Treatment and a Collaborative Pathway to Success. J Clin Oncol. 2015;33(27):3029-35.

2. Ferrari S, Smeland S, Mercuri M, Bertoni F, Longhi A, Ruggieri P, et al. Neoadjuvant chemotherapy with high-dose Ifosfamide, high-dose methotrexate, cisplatin, and doxorubicin for patients with localized osteosarcoma of the extremity: a joint study by the Italian and Scandinavian Sarcoma Groups. J Clin Oncol. 2005;23(34):8845-52.

3. Siegel RL, Miller KD, Jemal A. Cancer statistics, 2019. CA Cancer J Clin. 2019;69(1):7-34.

4. Ritter J, Bielack SS. Osteosarcoma. Ann Oncol. 2010;21 Suppl 7:vii320-5.

5. Gill J, Gorlick R. Advancing therapy for osteosarcoma. Nat Rev Clin Oncol. 2021;18(10):609-24.

6. Bielack SS, Kempf-Bielack B, Delling G, Exner GU, Flege S, Helmke K, et al. Prognostic factors in high-grade osteosarcoma of the extremities or trunk: an analysis of 1,702 patients treated on neoadjuvant cooperative osteosarcoma study group protocols. J Clin Oncol. 2002;20(3):776-90.

7. Han Z, Peng X, Yang Y, Yi J, Zhao D, Bao Q, et al. Integrated microfluidic-SERS for exosome biomarker profiling and osteosarcoma diagnosis. Biosens Bioelectron. 2022;217:114709.

8. Wu Y, Xu L, Yang P, Lin N, Huang X, Pan W, et al. Survival Prediction in High-grade Osteosarcoma Using Radiomics of Diagnostic Computed Tomography. EBioMedicine. 2018;34:27-34.

9. Chen W, Lin Y, Huang J, Yan Z, Cao H. A novel risk score model based on glycolysis-related genes and a prognostic model for predicting overall survival of osteosarcoma patients. J Orthop Res. 2022;40(10):2372-81.

11. Li L, Wang Y, He X, Li Z, Lu M, Gong T, et al. Hematological Prognostic Scoring System Can Predict Overall Survival and Can Indicate Response to Immunotherapy in Patients With Osteosarcoma. Front Immunol. 2022;13:879560.

12. Pelish HE, Liau BB, Nitulescu, II, Tangpeerachaikul A, Poss ZC, Da Silva DH, et al. Mediator kinase inhibition further activates super-enhancer-associated genes in AML. Nature. 2015;526(7572):273-6.

13. Whyte WA, Orlando DA, Hnisz D, Abraham BJ, Lin CY, Kagey MH, et al. Master transcription factors and mediator establish super-enhancers at key cell identity genes. Cell. 2013;153(2):307-19.

14. Tögel L, Nightingale R, Chueh AC, Jayachandran A, Tran H, Phesse T, et al. Dual Targeting of Bromodomain and Extraterminal Domain Proteins, and WNT or MAPK Signaling, Inhibits c-MYC Expression and Proliferation of Colorectal Cancer Cells. Mol Cancer Ther. 2016;15(6):1217-26.

15. Mansour MR, Abraham BJ, Anders L, Berezovskaya A, Gutierrez A, Durbin AD, et al. Oncogene regulation. An oncogenic super-enhancer formed through somatic mutation of a noncoding intergenic element. Science. 2014;346(6215):1373-7.

16. Lovén J, Hoke HA, Lin CY, Lau A, Orlando DA, Vakoc CR, et al. Selective inhibition of tumor oncogenes by disruption of super-enhancers. Cell. 2013;153(2):320-34.

17. Wang Y, Zhang T, Kwiatkowski N, Abraham BJ, Lee TI, Xie S, et al. CDK7-dependent transcriptional addiction in triple-negative breast cancer. Cell. 2015;163(1):174-86.

18. Zhang J, Liu W, Zou C, Zhao Z, Lai Y, Shi Z, et al. Targeting Super-Enhancer-Associated Oncogenes in Osteosarcoma with THZ2, a Covalent CDK7 Inhibitor. Clin Cancer Res. 2020;26(11):2681-92.

19. Xing R, Zhou Y, Yu J, Yu Y, Nie Y, Luo W, et al. Whole-genome sequencing reveals novel tandem-duplication hotspots and a prognostic mutational signature in gastric cancer. Nat Commun. 2019;10(1):2037.

20. Zou CY, Wang J, Shen JN, Huang G, Jin S, Yin JQ, et al. Establishment and characteristics of two syngeneic human osteosarcoma cell lines from primary tumor and skip metastases. Acta Pharmacol Sin. 2008;29(3):325-32.

1. Isakoff MS, Bielack SS, Meltzer P, Gorlick R. Osteosarcoma: Current Treatment and a Collaborative Pathway to Success. J Clin Oncol. 2015;33(27):3029-35.

2. Ferrari S, Smeland S, Mercuri M, Bertoni F, Longhi A, Ruggieri P, et al. Neoadjuvant chemotherapy with high-dose Ifosfamide, high-dose methotrexate, cisplatin, and doxorubicin for patients with localized osteosarcoma of the extremity: a joint study by the Italian and Scandinavian Sarcoma Groups. J Clin Oncol. 2005;23(34):8845-52.

3. Siegel RL, Miller KD, Jemal A. Cancer statistics, 2019. CA Cancer J Clin. 2019;69(1):7-34.

4. Ritter J, Bielack SS. Osteosarcoma. Ann Oncol. 2010;21 Suppl 7:vii320-5.

5. Gill J, Gorlick R. Advancing therapy for osteosarcoma. Nat Rev Clin Oncol. 2021;18(10):609-24.

6. Bielack SS, Kempf-Bielack B, Delling G, Exner GU, Flege S, Helmke K, et al. Prognostic factors in high-grade osteosarcoma of the extremities or trunk: an analysis of 1,702 patients treated on neoadjuvant cooperative osteosarcoma study group protocols. J Clin Oncol. 2002;20(3):776-90.

7. Han Z, Peng X, Yang Y, Yi J, Zhao D, Bao Q, et al. Integrated microfluidic-SERS for exosome biomarker profiling and osteosarcoma diagnosis. Biosens Bioelectron. 2022;217:114709.

8. Wu Y, Xu L, Yang P, Lin N, Huang X, Pan W, et al. Survival Prediction in High-grade Osteosarcoma Using Radiomics of Diagnostic Computed Tomography. EBioMedicine. 2018;34:27-34.

9. Chen W, Lin Y, Huang J, Yan Z, Cao H. A novel risk score model based on glycolysis-related genes and a prognostic model for predicting overall survival of osteosarcoma patients. J Orthop Res. 2022;40(10):2372-81.

10. Zhang M, Liu Y, Kong D. Identifying biomolecules and constructing a prognostic risk prediction model for recurrence in osteosarcoma. J Bone Oncol. 2021;26:100331.

11. Li L, Wang Y, He X, Li Z, Lu M, Gong T, et al. Hematological Prognostic Scoring System Can Predict Overall Survival and Can Indicate Response to Immunotherapy in Patients With Osteosarcoma. Front Immunol. 2022;13:879560.

12. Pelish HE, Liau BB, Nitulescu, II, Tangpeerachaikul A, Poss ZC, Da Silva DH, et al. Mediator kinase inhibition further activates super-enhancer-associated genes in AML. Nature. 2015;526(7572):273-6.

13. Whyte WA, Orlando DA, Hnisz D, Abraham BJ, Lin CY, Kagey MH, et al. Master transcription factors and mediator establish super-enhancers at key cell identity genes. Cell. 2013;153(2):307-19.

14. Tögel L, Nightingale R, Chueh AC, Jayachandran A, Tran H, Phesse T, et al. Dual Targeting of Bromodomain and Extraterminal Domain Proteins, and WNT or MAPK Signaling, Inhibits c-MYC Expression and Proliferation of Colorectal Cancer Cells. Mol Cancer Ther. 2016;15(6):1217-26.

15. Mansour MR, Abraham BJ, Anders L, Berezovskaya A, Gutierrez A, Durbin AD, et al. Oncogene regulation. An oncogenic super-enhancer formed through somatic mutation of a noncoding intergenic element. Science. 2014;346(6215):1373-7.

16. Lovén J, Hoke HA, Lin CY, Lau A, Orlando DA, Vakoc CR, et al. Selective inhibition of tumor oncogenes by disruption of super-enhancers. Cell. 2013;153(2):320-34.

17. Wang Y, Zhang T, Kwiatkowski N, Abraham BJ, Lee TI, Xie S, et al. CDK7-dependent transcriptional addiction in triple-negative breast cancer. Cell. 2015;163(1):174-86.

18. Zhang J, Liu W, Zou C, Zhao Z, Lai Y, Shi Z, et al. Targeting Super-Enhancer-Associated Oncogenes in Osteosarcoma with THZ2, a Covalent CDK7 Inhibitor. Clin Cancer Res. 2020;26(11):2681-92.

19. Xing R, Zhou Y, Yu J, Yu Y, Nie Y, Luo W, et al. Whole-genome sequencing reveals novel tandem-duplication hotspots and a prognostic mutational signature in gastric cancer. Nat Commun. 2019;10(1):2037.

20. Zou CY, Wang J, Shen JN, Huang G, Jin S, Yin JQ, et al. Establishment and characteristics of two syngeneic human osteosarcoma cell lines from primary tumor and skip metastases. Acta Pharmacol Sin. 2008;29(3):325-32.

21. Han J, Yong B, Luo C, Tan P, Peng T, Shen J. High serum alkaline phosphatase cooperating with MMP-9 predicts metastasis and poor prognosis in patients with primary osteosarcoma in Southern China. World J Surg Oncol. 2012;10:37.

1. Isakoff MS, Bielack SS, Meltzer P, Gorlick R. Osteosarcoma: Current Treatment and a Collaborative Pathway to Success. J Clin Oncol. 2015;33(27):3029-35.

2. Ferrari S, Smeland S, Mercuri M, Bertoni F, Longhi A, Ruggieri P, et al. Neoadjuvant chemotherapy with high-dose Ifosfamide, high-dose methotrexate, cisplatin, and doxorubicin for patients with localized osteosarcoma of the extremity: a joint study by the Italian and Scandinavian Sarcoma Groups. J Clin Oncol. 2005;23(34):8845-52.

3. Siegel RL, Miller KD, Jemal A. Cancer statistics, 2019. CA Cancer J Clin. 2019;69(1):7-34.

4. Ritter J, Bielack SS. Osteosarcoma. Ann Oncol. 2010;21 Suppl 7:vii320-5.

5. Gill J, Gorlick R. Advancing therapy for osteosarcoma. Nat Rev Clin Oncol. 2021;18(10):609-24.

6. Bielack SS, Kempf-Bielack B, Delling G, Exner GU, Flege S, Helmke K, et al. Prognostic factors in high-grade osteosarcoma of the extremities or trunk: an analysis of 1,702 patients treated on neoadjuvant cooperative osteosarcoma study group protocols. J Clin Oncol. 2002;20(3):776-90.

7. Han Z, Peng X, Yang Y, Yi J, Zhao D, Bao Q, et al. Integrated microfluidic-SERS for exosome biomarker profiling and osteosarcoma diagnosis. Biosens Bioelectron. 2022;217:114709.

8. Wu Y, Xu L, Yang P, Lin N, Huang X, Pan W, et al. Survival Prediction in High-grade Osteosarcoma Using Radiomics of Diagnostic Computed Tomography. EBioMedicine. 2018;34:27-34.

9. Chen W, Lin Y, Huang J, Yan Z, Cao H. A novel risk score model based on glycolysis-related genes and a prognostic model for predicting overall survival of osteosarcoma patients. J Orthop Res. 2022;40(10):2372-81.

10. Zhang M, Liu Y, Kong D. Identifying biomolecules and constructing a prognostic risk prediction model for recurrence in osteosarcoma. J Bone Oncol. 2021;26:100331.

11. Li L, Wang Y, He X, Li Z, Lu M, Gong T, et al. Hematological Prognostic Scoring System Can Predict Overall Survival and Can Indicate Response to Immunotherapy in Patients With Osteosarcoma. Front Immunol. 2022;13:879560.

12. Pelish HE, Liau BB, Nitulescu, II, Tangpeerachaikul A, Poss ZC, Da Silva DH, et al. Mediator kinase inhibition further activates super-enhancer-associated genes in AML. Nature. 2015;526(7572):273-6.

13. Whyte WA, Orlando DA, Hnisz D, Abraham BJ, Lin CY, Kagey MH, et al. Master transcription factors and mediator establish super-enhancers at key cell identity genes. Cell. 2013;153(2):307-19.

14. Tögel L, Nightingale R, Chueh AC, Jayachandran A, Tran H, Phesse T, et al. Dual Targeting of Bromodomain and Extraterminal Domain Proteins, and WNT or MAPK Signaling, Inhibits c-MYC Expression and Proliferation of Colorectal Cancer Cells. Mol Cancer Ther. 2016;15(6):1217-26.

15. Mansour MR, Abraham BJ, Anders L, Berezovskaya A, Gutierrez A, Durbin AD, et al. Oncogene regulation. An oncogenic super-enhancer formed through somatic mutation of a noncoding intergenic element. Science. 2014;346(6215):1373-7.

16. Lovén J, Hoke HA, Lin CY, Lau A, Orlando DA, Vakoc CR, et al. Selective inhibition of tumor oncogenes by disruption of super-enhancers. Cell. 2013;153(2):320-34.

17. Wang Y, Zhang T, Kwiatkowski N, Abraham BJ, Lee TI, Xie S, et al. CDK7-dependent transcriptional addiction in triple-negative breast cancer. Cell. 2015;163(1):174-86.

19. Xing R, Zhou Y, Yu J, Yu Y, Nie Y, Luo W, et al. Whole-genome sequencing reveals novel tandem-duplication hotspots and a prognostic mutational signature in gastric cancer. Nat Commun. 2019;10(1):2037.

20. Zou CY, Wang J, Shen JN, Huang G, Jin S, Yin JQ, et al. Establishment and characteristics of two syngeneic human osteosarcoma cell lines from primary tumor and skip metastases. Acta Pharmacol Sin. 2008;29(3):325-32.

21. Han J, Yong B, Luo C, Tan P, Peng T, Shen J. High serum alkaline phosphatase cooperating with MMP-9 predicts metastasis and poor prognosis in patients with primary osteosarcoma in Southern China. World J Surg Oncol. 2012;10:37.

22. Szulc P, Seeman E, Delmas PD. Biochemical measurements of bone turnover in children and adolescents. Osteoporos Int. 2000;11(4):281-94.

1. Isakoff MS, Bielack SS, Meltzer P, Gorlick R. Osteosarcoma: Current Treatment and a Collaborative Pathway to Success. J Clin Oncol. 2015;33(27):3029-35.

2. Ferrari S, Smeland S, Mercuri M, Bertoni F, Longhi A, Ruggieri P, et al. Neoadjuvant chemotherapy with high-dose Ifosfamide, high-dose methotrexate, cisplatin, and doxorubicin for patients with localized osteosarcoma of the extremity: a joint study by the Italian and Scandinavian Sarcoma Groups. J Clin Oncol. 2005;23(34):8845-52.

3. Siegel RL, Miller KD, Jemal A. Cancer statistics, 2019. CA Cancer J Clin. 2019;69(1):7-34.

4. Ritter J, Bielack SS. Osteosarcoma. Ann Oncol. 2010;21 Suppl 7:vii320-5.

5. Gill J, Gorlick R. Advancing therapy for osteosarcoma. Nat Rev Clin Oncol. 2021;18(10):609-24.

6. Bielack SS, Kempf-Bielack B, Delling G, Exner GU, Flege S, Helmke K, et al. Prognostic factors in high-grade osteosarcoma of the extremities or trunk: an analysis of 1,702 patients treated on neoadjuvant cooperative osteosarcoma study group protocols. J Clin Oncol. 2002;20(3):776-90.

7. Han Z, Peng X, Yang Y, Yi J, Zhao D, Bao Q, et al. Integrated microfluidic-SERS for exosome biomarker profiling and osteosarcoma diagnosis. Biosens Bioelectron. 2022;217:114709.

8. Wu Y, Xu L, Yang P, Lin N, Huang X, Pan W, et al. Survival Prediction in High-grade Osteosarcoma Using Radiomics of Diagnostic Computed Tomography. EBioMedicine. 2018;34:27-34.

9. Chen W, Lin Y, Huang J, Yan Z, Cao H. A novel risk score model based on glycolysis-related genes and a prognostic model for predicting overall survival of osteosarcoma patients. J Orthop Res. 2022;40(10):2372-81.

10. Zhang M, Liu Y, Kong D. Identifying biomolecules and constructing a prognostic risk prediction model for recurrence in osteosarcoma. J Bone Oncol. 2021;26:100331.

11. Li L, Wang Y, He X, Li Z, Lu M, Gong T, et al. Hematological Prognostic Scoring System Can Predict Overall Survival and Can Indicate Response to Immunotherapy in Patients With Osteosarcoma. Front Immunol. 2022;13:879560.

12. Pelish HE, Liau BB, Nitulescu, II, Tangpeerachaikul A, Poss ZC, Da Silva DH, et al. Mediator kinase inhibition further activates super-enhancer-associated genes in AML. Nature. 2015;526(7572):273-6.

13. Whyte WA, Orlando DA, Hnisz D, Abraham BJ, Lin CY, Kagey MH, et al. Master transcription factors and mediator establish super-enhancers at key cell identity genes. Cell. 2013;153(2):307-19.

14. Tögel L, Nightingale R, Chueh AC, Jayachandran A, Tran H, Phesse T, et al. Dual Targeting of Bromodomain and Extraterminal Domain Proteins, and WNT or MAPK Signaling, Inhibits c-MYC Expression and Proliferation of Colorectal Cancer Cells. Mol Cancer Ther. 2016;15(6):1217-26.

15. Mansour MR, Abraham BJ, Anders L, Berezovskaya A, Gutierrez A, Durbin AD, et al. Oncogene regulation. An oncogenic super-enhancer formed through somatic mutation of a noncoding intergenic element. Science. 2014;346(6215):1373-7.

16. Lovén J, Hoke HA, Lin CY, Lau A, Orlando DA, Vakoc CR, et al. Selective inhibition of tumor oncogenes by disruption of super-enhancers. Cell. 2013;153(2):320-34.

17. Wang Y, Zhang T, Kwiatkowski N, Abraham BJ, Lee TI, Xie S, et al. CDK7-dependent transcriptional addiction in triple-negative breast cancer. Cell. 2015;163(1):174-86.

18. Zhang J, Liu W, Zou C, Zhao Z, Lai Y, Shi Z, et al. Targeting Super-Enhancer-Associated Oncogenes in Osteosarcoma with THZ2, a Covalent CDK7 Inhibitor. Clin Cancer Res. 2020;26(11):2681-92.

19. Xing R, Zhou Y, Yu J, Yu Y, Nie Y, Luo W, et al. Whole-genome sequencing reveals novel tandem-duplication hotspots and a prognostic mutational signature in gastric cancer. Nat Commun. 2019;10(1):2037.

20. Zou CY, Wang J, Shen JN, Huang G, Jin S, Yin JQ, et al. Establishment and characteristics of two syngeneic human osteosarcoma cell lines from primary tumor and skip metastases. Acta Pharmacol Sin. 2008;29(3):325-32.

21. Han J, Yong B, Luo C, Tan P, Peng T, Shen J. High serum alkaline phosphatase cooperating with MMP-9 predicts metastasis and poor prognosis in patients with primary osteosarcoma in Southern China. World J Surg Oncol. 2012;10:37.

22. Szulc P, Seeman E, Delmas PD. Biochemical measurements of bone turnover in children and adolescents. Osteoporos Int. 2000;11(4):281-94.

23. Camp RL, Dolled-Filhart M, Rimm DL. X-tile: a new bio-informatics tool for biomarker assessment and outcome-based cut-point optimization. Clin Cancer Res. 2004;10(21):7252-9.

1. Isakoff MS, Bielack SS, Meltzer P, Gorlick R. Osteosarcoma: Current Treatment and a Collaborative Pathway to Success. J Clin Oncol. 2015;33(27):3029-35.

2. Ferrari S, Smeland S, Mercuri M, Bertoni F, Longhi A, Ruggieri P, et al. Neoadjuvant chemotherapy with high-dose Ifosfamide, high-dose methotrexate, cisplatin, and doxorubicin for patients with localized osteosarcoma of the extremity: a joint study by the Italian and Scandinavian Sarcoma Groups. J Clin Oncol. 2005;23(34):8845-52.

3. Siegel RL, Miller KD, Jemal A. Cancer statistics, 2019. CA Cancer J Clin. 2019;69(1):7-34.

4. Ritter J, Bielack SS. Osteosarcoma. Ann Oncol. 2010;21 Suppl 7:vii320-5.

5. Gill J, Gorlick R. Advancing therapy for osteosarcoma. Nat Rev Clin Oncol. 2021;18(10):609-24.

6. Bielack SS, Kempf-Bielack B, Delling G, Exner GU, Flege S, Helmke K, et al. Prognostic factors in high-grade osteosarcoma of the extremities or trunk: an analysis of 1,702 patients treated on neoadjuvant cooperative osteosarcoma study group protocols. J Clin Oncol. 2002;20(3):776-90.

7. Han Z, Peng X, Yang Y, Yi J, Zhao D, Bao Q, et al. Integrated microfluidic-SERS for exosome biomarker profiling and osteosarcoma diagnosis. Biosens Bioelectron. 2022;217:114709.

8. Wu Y, Xu L, Yang P, Lin N, Huang X, Pan W, et al. Survival Prediction in High-grade Osteosarcoma Using Radiomics of Diagnostic Computed Tomography. EBioMedicine. 2018;34:27-34.

9. Chen W, Lin Y, Huang J, Yan Z, Cao H. A novel risk score model based on glycolysis-related genes and a prognostic model for predicting overall survival of osteosarcoma patients. J Orthop Res. 2022;40(10):2372-81.

10. Zhang M, Liu Y, Kong D. Identifying biomolecules and constructing a prognostic risk prediction model for recurrence in osteosarcoma. J Bone Oncol. 2021;26:100331.

11. Li L, Wang Y, He X, Li Z, Lu M, Gong T, et al. Hematological Prognostic Scoring System Can Predict Overall Survival and Can Indicate Response to Immunotherapy in Patients With Osteosarcoma. Front Immunol. 2022;13:879560.

12. Pelish HE, Liau BB, Nitulescu, II, Tangpeerachaikul A, Poss ZC, Da Silva DH, et al. Mediator kinase inhibition further activates super-enhancer-associated genes in AML. Nature. 2015;526(7572):273-6.

13. Whyte WA, Orlando DA, Hnisz D, Abraham BJ, Lin CY, Kagey MH, et al. Master transcription factors and mediator establish super-enhancers at key cell identity genes. Cell. 2013;153(2):307-19.

14. Tögel L, Nightingale R, Chueh AC, Jayachandran A, Tran H, Phesse T, et al. Dual Targeting of Bromodomain and Extraterminal Domain Proteins, and WNT or MAPK Signaling, Inhibits c-MYC Expression and Proliferation of Colorectal Cancer Cells. Mol Cancer Ther. 2016;15(6):1217-26.

15. Mansour MR, Abraham BJ, Anders L, Berezovskaya A, Gutierrez A, Durbin AD, et al. Oncogene regulation. An oncogenic super-enhancer formed through somatic mutation of a noncoding intergenic element. Science. 2014;346(6215):1373-7.

16. Lovén J, Hoke HA, Lin CY, Lau A, Orlando DA, Vakoc CR, et al. Selective inhibition of tumor oncogenes by disruption of super-enhancers. Cell. 2013;153(2):320-34.

17. Wang Y, Zhang T, Kwiatkowski N, Abraham BJ, Lee TI, Xie S, et al. CDK7-dependent transcriptional addiction in triple-negative breast cancer. Cell. 2015;163(1):174-86.

18. Zhang J, Liu W, Zou C, Zhao Z, Lai Y, Shi Z, et al. Targeting Super-Enhancer-Associated Oncogenes in Osteosarcoma with THZ2, a Covalent CDK7 Inhibitor. Clin Cancer Res. 2020;26(11):2681-92.

19. Xing R, Zhou Y, Yu J, Yu Y, Nie Y, Luo W, et al. Whole-genome sequencing reveals novel tandem-duplication hotspots and a prognostic mutational signature in gastric cancer. Nat Commun. 2019;10(1):2037.

20. Zou CY, Wang J, Shen JN, Huang G, Jin S, Yin JQ, et al. Establishment and characteristics of two syngeneic human osteosarcoma cell lines from primary tumor and skip metastases. Acta Pharmacol Sin. 2008;29(3):325-32.

21. Han J, Yong B, Luo C, Tan P, Peng T, Shen J. High serum alkaline phosphatase cooperating with MMP-9 predicts metastasis and poor prognosis in patients with primary osteosarcoma in Southern China. World J Surg Oncol. 2012;10:37.

22. Szulc P, Seeman E, Delmas PD. Biochemical measurements of bone turnover in children and adolescents. Osteoporos Int. 2000;11(4):281-94.

23. Camp RL, Dolled-Filhart M, Rimm DL. X-tile: a new bio-informatics tool for biomarker assessment and outcome-based cut-point optimization. Clin Cancer Res. 2004;10(21):7252-9.

1. Isakoff MS, Bielack SS, Meltzer P, Gorlick R. Osteosarcoma: Current Treatment and a Collaborative Pathway to Success. J Clin Oncol. 2015;33(27):3029-35.

2. Ferrari S, Smeland S, Mercuri M, Bertoni F, Longhi A, Ruggieri P, et al. Neoadjuvant chemotherapy with high-dose Ifosfamide, high-dose methotrexate, cisplatin, and doxorubicin for patients with localized osteosarcoma of the extremity: a joint study by the Italian and Scandinavian Sarcoma Groups. J Clin Oncol. 2005;23(34):8845-52.

3. Siegel RL, Miller KD, Jemal A. Cancer statistics, 2019. CA Cancer J Clin. 2019;69(1):7-34.

4. Ritter J, Bielack SS. Osteosarcoma. Ann Oncol. 2010;21 Suppl 7:vii320-5.

5. Gill J, Gorlick R. Advancing therapy for osteosarcoma. Nat Rev Clin Oncol. 2021;18(10):609-24.

6. Bielack SS, Kempf-Bielack B, Delling G, Exner GU, Flege S, Helmke K, et al. Prognostic factors in high-grade osteosarcoma of the extremities or trunk: an analysis of 1,702 patients treated on neoadjuvant cooperative osteosarcoma study group protocols. J Clin Oncol. 2002;20(3):776-90.

7. Han Z, Peng X, Yang Y, Yi J, Zhao D, Bao Q, et al. Integrated microfluidic-SERS for exosome biomarker profiling and osteosarcoma diagnosis. Biosens Bioelectron. 2022;217:114709.

8. Wu Y, Xu L, Yang P, Lin N, Huang X, Pan W, et al. Survival Prediction in High-grade Osteosarcoma Using Radiomics of Diagnostic Computed Tomography. EBioMedicine. 2018;34:27-34.

9. Chen W, Lin Y, Huang J, Yan Z, Cao H. A novel risk score model based on glycolysis-related genes and a prognostic model for predicting overall survival of osteosarcoma patients. J Orthop Res. 2022;40(10):2372-81.

10. Zhang M, Liu Y, Kong D. Identifying biomolecules and constructing a prognostic risk prediction model for recurrence in osteosarcoma. J Bone Oncol. 2021;26:100331.

11. Li L, Wang Y, He X, Li Z, Lu M, Gong T, et al. Hematological Prognostic Scoring System Can Predict Overall Survival and Can Indicate Response to Immunotherapy in Patients With Osteosarcoma. Front Immunol. 2022;13:879560.

12. Pelish HE, Liau BB, Nitulescu, II, Tangpeerachaikul A, Poss ZC, Da Silva DH, et al. Mediator kinase inhibition further activates super-enhancer-associated genes in AML. Nature. 2015;526(7572):273-6.

13. Whyte WA, Orlando DA, Hnisz D, Abraham BJ, Lin CY, Kagey MH, et al. Master transcription factors and mediator establish super-enhancers at key cell identity genes. Cell. 2013;153(2):307-19.

14. Tögel L, Nightingale R, Chueh AC, Jayachandran A, Tran H, Phesse T, et al. Dual Targeting of Bromodomain and Extraterminal Domain Proteins, and WNT or MAPK Signaling, Inhibits c-MYC Expression and Proliferation of Colorectal Cancer Cells. Mol Cancer Ther. 2016;15(6):1217-26.

15. Mansour MR, Abraham BJ, Anders L, Berezovskaya A, Gutierrez A, Durbin AD, et al. Oncogene regulation. An oncogenic super-enhancer formed through somatic mutation of a noncoding intergenic element. Science. 2014;346(6215):1373-7.

16. Lovén J, Hoke HA, Lin CY, Lau A, Orlando DA, Vakoc CR, et al. Selective inhibition of tumor oncogenes by disruption of super-enhancers. Cell. 2013;153(2):320-34.

17. Wang Y, Zhang T, Kwiatkowski N, Abraham BJ, Lee TI, Xie S, et al. CDK7-dependent transcriptional addiction in triple-negative breast cancer. Cell. 2015;163(1):174-86.

18. Zhang J, Liu W, Zou C, Zhao Z, Lai Y, Shi Z, et al. Targeting Super-Enhancer-Associated Oncogenes in Osteosarcoma with THZ2, a Covalent CDK7 Inhibitor. Clin Cancer Res. 2020;26(11):2681-92.

19. Xing R, Zhou Y, Yu J, Yu Y, Nie Y, Luo W, et al. Whole-genome sequencing reveals novel tandem-duplication hotspots and a prognostic mutational signature in gastric cancer. Nat Commun. 2019;10(1):2037.

20. Zou CY, Wang J, Shen JN, Huang G, Jin S, Yin JQ, et al. Establishment and characteristics of two syngeneic human osteosarcoma cell lines from primary tumor and skip metastases. Acta Pharmacol Sin. 2008;29(3):325-32.

21. Han J, Yong B, Luo C, Tan P, Peng T, Shen J. High serum alkaline phosphatase cooperating with MMP-9 predicts metastasis and poor prognosis in patients with primary osteosarcoma in Southern China. World J Surg Oncol. 2012;10:37.

22. Szulc P, Seeman E, Delmas PD. Biochemical measurements of bone turnover in children and adolescents. Osteoporos Int. 2000;11(4):281-94.

23. Camp RL, Dolled-Filhart M, Rimm DL. X-tile: a new bio-informatics tool for biomarker assessment and outcome-based cut-point optimization. Clin Cancer Res. 2004;10(21):7252-9.

24. Zhang T, Kwiatkowski N, Olson CM, Dixon-Clarke SE, Abraham BJ, Greifenberg AK, et al. Covalent targeting of remote cysteine residues to develop CDK12 and CDK13 inhibitors. Nat Chem Biol. 2016;12(10):876-84.

1. Isakoff MS, Bielack SS, Meltzer P, Gorlick R. Osteosarcoma: Current Treatment and a Collaborative Pathway to Success. J Clin Oncol. 2015;33(27):3029-35.

2. Ferrari S, Smeland S, Mercuri M, Bertoni F, Longhi A, Ruggieri P, et al. Neoadjuvant chemotherapy with high-dose Ifosfamide, high-dose methotrexate, cisplatin, and doxorubicin for patients with localized osteosarcoma of the extremity: a joint study by the Italian and Scandinavian Sarcoma Groups. J Clin Oncol. 2005;23(34):8845-52.

3. Siegel RL, Miller KD, Jemal A. Cancer statistics, 2019. CA Cancer J Clin. 2019;69(1):7-34.

4. Ritter J, Bielack SS. Osteosarcoma. Ann Oncol. 2010;21 Suppl 7:vii320-5.

5. Gill J, Gorlick R. Advancing therapy for osteosarcoma. Nat Rev Clin Oncol. 2021;18(10):609-24.

6. Bielack SS, Kempf-Bielack B, Delling G, Exner GU, Flege S, Helmke K, et al. Prognostic factors in high-grade osteosarcoma of the extremities or trunk: an analysis of 1,702 patients treated on neoadjuvant cooperative osteosarcoma study group protocols. J Clin Oncol. 2002;20(3):776-90.

7. Han Z, Peng X, Yang Y, Yi J, Zhao D, Bao Q, et al. Integrated microfluidic-SERS for exosome biomarker profiling and osteosarcoma diagnosis. Biosens Bioelectron. 2022;217:114709.

8. Wu Y, Xu L, Yang P, Lin N, Huang X, Pan W, et al. Survival Prediction in High-grade Osteosarcoma Using Radiomics of Diagnostic Computed Tomography. EBioMedicine. 2018;34:27-34.

9. Chen W, Lin Y, Huang J, Yan Z, Cao H. A novel risk score model based on glycolysis-related genes and a prognostic model for predicting overall survival of osteosarcoma patients. J Orthop Res. 2022;40(10):2372-81.

10. Zhang M, Liu Y, Kong D. Identifying biomolecules and constructing a prognostic risk prediction model for recurrence in osteosarcoma. J Bone Oncol. 2021;26:100331.

11. Li L, Wang Y, He X, Li Z, Lu M, Gong T, et al. Hematological Prognostic Scoring System Can Predict Overall Survival and Can Indicate Response to Immunotherapy in Patients With Osteosarcoma. Front Immunol. 2022;13:879560.

12. Pelish HE, Liau BB, Nitulescu, II, Tangpeerachaikul A, Poss ZC, Da Silva DH, et al. Mediator kinase inhibition further activates super-enhancer-associated genes in AML. Nature. 2015;526(7572):273-6.

13. Whyte WA, Orlando DA, Hnisz D, Abraham BJ, Lin CY, Kagey MH, et al. Master transcription factors and mediator establish super-enhancers at key cell identity genes. Cell. 2013;153(2):307-19.

14. Tögel L, Nightingale R, Chueh AC, Jayachandran A, Tran H, Phesse T, et al. Dual Targeting of Bromodomain and Extraterminal Domain Proteins, and WNT or MAPK Signaling, Inhibits c-MYC Expression and Proliferation of Colorectal Cancer Cells. Mol Cancer Ther. 2016;15(6):1217-26.

15. Mansour MR, Abraham BJ, Anders L, Berezovskaya A, Gutierrez A, Durbin AD, et al. Oncogene regulation. An oncogenic super-enhancer formed through somatic mutation of a noncoding intergenic element. Science. 2014;346(6215):1373-7.

16. Lovén J, Hoke HA, Lin CY, Lau A, Orlando DA, Vakoc CR, et al. Selective inhibition of tumor oncogenes by disruption of super-enhancers. Cell. 2013;153(2):320-34.

17. Wang Y, Zhang T, Kwiatkowski N, Abraham BJ, Lee TI, Xie S, et al. CDK7-dependent transcriptional addiction in triple-negative breast cancer. Cell. 2015;163(1):174-86.

18. Zhang J, Liu W, Zou C, Zhao Z, Lai Y, Shi Z, et al. Targeting Super-Enhancer-Associated Oncogenes in Osteosarcoma with THZ2, a Covalent CDK7 Inhibitor. Clin Cancer Res. 2020;26(11):2681-92.

19. Xing R, Zhou Y, Yu J, Yu Y, Nie Y, Luo W, et al. Whole-genome sequencing reveals novel tandem-duplication hotspots and a prognostic mutational signature in gastric cancer. Nat Commun. 2019;10(1):2037.

20. Zou CY, Wang J, Shen JN, Huang G, Jin S, Yin JQ, et al. Establishment and characteristics of two syngeneic human osteosarcoma cell lines from primary tumor and skip metastases. Acta Pharmacol Sin. 2008;29(3):325-32.

21. Han J, Yong B, Luo C, Tan P, Peng T, Shen J. High serum alkaline phosphatase cooperating with MMP-9 predicts metastasis and poor prognosis in patients with primary osteosarcoma in Southern China. World J Surg Oncol. 2012;10:37.

22. Szulc P, Seeman E, Delmas PD. Biochemical measurements of bone turnover in children and adolescents. Osteoporos Int. 2000;11(4):281-94.

23. Camp RL, Dolled-Filhart M, Rimm DL. X-tile: a new bio-informatics tool for biomarker assessment and outcome-based cut-point optimization. Clin Cancer Res. 2004;10(21):7252-9.

24. Zhang T, Kwiatkowski N, Olson CM, Dixon-Clarke SE, Abraham BJ, Greifenberg AK, et al. Covalent targeting of remote cysteine residues to develop CDK12 and CDK13 inhibitors. Nat Chem Biol. 2016;12(10):876-84.

1. Isakoff MS, Bielack SS, Meltzer P, Gorlick R. Osteosarcoma: Current Treatment and a Collaborative Pathway to Success. J Clin Oncol. 2015;33(27):3029-35.

2. Ferrari S, Smeland S, Mercuri M, Bertoni F, Longhi A, Ruggieri P, et al. Neoadjuvant chemotherapy with high-dose Ifosfamide, high-dose methotrexate, cisplatin, and doxorubicin for patients with localized osteosarcoma of the extremity: a joint study by the Italian and Scandinavian Sarcoma Groups. J Clin Oncol. 2005;23(34):8845-52.

3. Siegel RL, Miller KD, Jemal A. Cancer statistics, 2019. CA Cancer J Clin. 2019;69(1):7-34.

4. Ritter J, Bielack SS. Osteosarcoma. Ann Oncol. 2010;21 Suppl 7:vii320-5.

6. Bielack SS, Kempf-Bielack B, Delling G, Exner GU, Flege S, Helmke K, et al. Prognostic factors in high-grade osteosarcoma of the extremities or trunk: an analysis of 1,702 patients treated on neoadjuvant cooperative osteosarcoma study group protocols. J Clin Oncol. 2002;20(3):776-90.

7. Han Z, Peng X, Yang Y, Yi J, Zhao D, Bao Q, et al. Integrated microfluidic-SERS for exosome biomarker profiling and osteosarcoma diagnosis. Biosens Bioelectron. 2022;217:114709.

8. Wu Y, Xu L, Yang P, Lin N, Huang X, Pan W, et al. Survival Prediction in High-grade Osteosarcoma Using Radiomics of Diagnostic Computed Tomography. EBioMedicine. 2018;34:27-34.

9. Chen W, Lin Y, Huang J, Yan Z, Cao H. A novel risk score model based on glycolysis-related genes and a prognostic model for predicting overall survival of osteosarcoma patients. J Orthop Res. 2022;40(10):2372-81.

10. Zhang M, Liu Y, Kong D. Identifying biomolecules and constructing a prognostic risk prediction model for recurrence in osteosarcoma. J Bone Oncol. 2021;26:100331.

11. Li L, Wang Y, He X, Li Z, Lu M, Gong T, et al. Hematological Prognostic Scoring System Can Predict Overall Survival and Can Indicate Response to Immunotherapy in Patients With Osteosarcoma. Front Immunol. 2022;13:879560.

12. Pelish HE, Liau BB, Nitulescu, II, Tangpeerachaikul A, Poss ZC, Da Silva DH, et al. Mediator kinase inhibition further activates super-enhancer-associated genes in AML. Nature. 2015;526(7572):273-6.

13. Whyte WA, Orlando DA, Hnisz D, Abraham BJ, Lin CY, Kagey MH, et al. Master transcription factors and mediator establish super-enhancers at key cell identity genes. Cell. 2013;153(2):307-19.

14. Tögel L, Nightingale R, Chueh AC, Jayachandran A, Tran H, Phesse T, et al. Dual Targeting of Bromodomain and Extraterminal Domain Proteins, and WNT or MAPK Signaling, Inhibits c-MYC Expression and Proliferation of Colorectal Cancer Cells. Mol Cancer Ther. 2016;15(6):1217-26.

15. Mansour MR, Abraham BJ, Anders L, Berezovskaya A, Gutierrez A, Durbin AD, et al. Oncogene regulation. An oncogenic super-enhancer formed through somatic mutation of a noncoding intergenic element. Science. 2014;346(6215):1373-7.

16. Lovén J, Hoke HA, Lin CY, Lau A, Orlando DA, Vakoc CR, et al. Selective inhibition of tumor oncogenes by disruption of super-enhancers. Cell. 2013;153(2):320-34.

17. Wang Y, Zhang T, Kwiatkowski N, Abraham BJ, Lee TI, Xie S, et al. CDK7-dependent transcriptional addiction in triple-negative breast cancer. Cell. 2015;163(1):174-86.

18. Zhang J, Liu W, Zou C, Zhao Z, Lai Y, Shi Z, et al. Targeting Super-Enhancer-Associated Oncogenes in Osteosarcoma with THZ2, a Covalent CDK7 Inhibitor. Clin Cancer Res. 2020;26(11):2681-92.

19. Xing R, Zhou Y, Yu J, Yu Y, Nie Y, Luo W, et al. Whole-genome sequencing reveals novel tandem-duplication hotspots and a prognostic mutational signature in gastric cancer. Nat Commun. 2019;10(1):2037.

20. Zou CY, Wang J, Shen JN, Huang G, Jin S, Yin JQ, et al. Establishment and characteristics of two syngeneic human osteosarcoma cell lines from primary tumor and skip metastases. Acta Pharmacol Sin. 2008;29(3):325-32.

21. Han J, Yong B, Luo C, Tan P, Peng T, Shen J. High serum alkaline phosphatase cooperating with MMP-9 predicts metastasis and poor prognosis in patients with primary osteosarcoma in Southern China. World J Surg Oncol. 2012;10:37.

22. Szulc P, Seeman E, Delmas PD. Biochemical measurements of bone turnover in children and adolescents. Osteoporos Int. 2000;11(4):281-94.

23. Camp RL, Dolled-Filhart M, Rimm DL. X-tile: a new bio-informatics tool for biomarker assessment and outcome-based cut-point optimization. Clin Cancer Res. 2004;10(21):7252-9.

24. Zhang T, Kwiatkowski N, Olson CM, Dixon-Clarke SE, Abraham BJ, Greifenberg AK, et al. Covalent targeting of remote cysteine residues to develop CDK12 and CDK13 inhibitors. Nat Chem Biol. 2016;12(10):876-84.

1. Isakoff MS, Bielack SS, Meltzer P, Gorlick R. Osteosarcoma: Current Treatment and a Collaborative Pathway to Success. J Clin Oncol. 2015;33(27):3029-35.

2. Ferrari S, Smeland S, Mercuri M, Bertoni F, Longhi A, Ruggieri P, et al. Neoadjuvant chemotherapy with high-dose Ifosfamide, high-dose methotrexate, cisplatin, and doxorubicin for patients with localized osteosarcoma of the extremity: a joint study by the Italian and Scandinavian Sarcoma Groups. J Clin Oncol. 2005;23(34):8845-52.

3. Siegel RL, Miller KD, Jemal A. Cancer statistics, 2019. CA Cancer J Clin. 2019;69(1):7-34.

4. Ritter J, Bielack SS. Osteosarcoma. Ann Oncol. 2010;21 Suppl 7:vii320-5.

5. Gill J, Gorlick R. Advancing therapy for osteosarcoma. Nat Rev Clin Oncol. 2021;18(10):609-24.

6. Bielack SS, Kempf-Bielack B, Delling G, Exner GU, Flege S, Helmke K, et al. Prognostic factors in high-grade osteosarcoma of the extremities or trunk: an analysis of 1,702 patients treated on neoadjuvant cooperative osteosarcoma study group protocols. J Clin Oncol. 2002;20(3):776-90.

7. Han Z, Peng X, Yang Y, Yi J, Zhao D, Bao Q, et al. Integrated microfluidic-SERS for exosome biomarker profiling and osteosarcoma diagnosis. Biosens Bioelectron. 2022;217:114709.

8. Wu Y, Xu L, Yang P, Lin N, Huang X, Pan W, et al. Survival Prediction in High-grade Osteosarcoma Using Radiomics of Diagnostic Computed Tomography. EBioMedicine. 2018;34:27-34.

9. Chen W, Lin Y, Huang J, Yan Z, Cao H. A novel risk score model based on glycolysis-related genes and a prognostic model for predicting overall survival of osteosarcoma patients. J Orthop Res. 2022;40(10):2372-81.

10. Zhang M, Liu Y, Kong D. Identifying biomolecules and constructing a prognostic risk prediction model for recurrence in osteosarcoma. J Bone Oncol. 2021;26:100331.

11. Li L, Wang Y, He X, Li Z, Lu M, Gong T, et al. Hematological Prognostic Scoring System Can Predict Overall Survival and Can Indicate Response to Immunotherapy in Patients With Osteosarcoma. Front Immunol. 2022;13:879560.

12. Pelish HE, Liau BB, Nitulescu, II, Tangpeerachaikul A, Poss ZC, Da Silva DH, et al. Mediator kinase inhibition further activates super-enhancer-associated genes in AML. Nature. 2015;526(7572):273-6.

13. Whyte WA, Orlando DA, Hnisz D, Abraham BJ, Lin CY, Kagey MH, et al. Master transcription factors and mediator establish super-enhancers at key cell identity genes. Cell. 2013;153(2):307-19.

14. Tögel L, Nightingale R, Chueh AC, Jayachandran A, Tran H, Phesse T, et al. Dual Targeting of Bromodomain and Extraterminal Domain Proteins, and WNT or MAPK Signaling, Inhibits c-MYC Expression and Proliferation of Colorectal Cancer Cells. Mol Cancer Ther. 2016;15(6):1217-26.

15. Mansour MR, Abraham BJ, Anders L, Berezovskaya A, Gutierrez A, Durbin AD, et al. Oncogene regulation. An oncogenic super-enhancer formed through somatic mutation of a noncoding intergenic element. Science. 2014;346(6215):1373-7.

16. Lovén J, Hoke HA, Lin CY, Lau A, Orlando DA, Vakoc CR, et al. Selective inhibition of tumor oncogenes by disruption of super-enhancers. Cell. 2013;153(2):320-34.

17. Wang Y, Zhang T, Kwiatkowski N, Abraham BJ, Lee TI, Xie S, et al. CDK7-dependent transcriptional addiction in triple-negative breast cancer. Cell. 2015;163(1):174-86.

18. Zhang J, Liu W, Zou C, Zhao Z, Lai Y, Shi Z, et al. Targeting Super-Enhancer-Associated Oncogenes in Osteosarcoma with THZ2, a Covalent CDK7 Inhibitor. Clin Cancer Res. 2020;26(11):2681-92.

19. Xing R, Zhou Y, Yu J, Yu Y, Nie Y, Luo W, et al. Whole-genome sequencing reveals novel tandem-duplication hotspots and a prognostic mutational signature in gastric cancer. Nat Commun. 2019;10(1):2037.

20. Zou CY, Wang J, Shen JN, Huang G, Jin S, Yin JQ, et al. Establishment and characteristics of two syngeneic human osteosarcoma cell lines from primary tumor and skip metastases. Acta Pharmacol Sin. 2008;29(3):325-32.

21. Han J, Yong B, Luo C, Tan P, Peng T, Shen J. High serum alkaline phosphatase cooperating with MMP-9 predicts metastasis and poor prognosis in patients with primary osteosarcoma in Southern China. World J Surg Oncol. 2012;10:37.

22. Szulc P, Seeman E, Delmas PD. Biochemical measurements of bone turnover in children and adolescents. Osteoporos Int. 2000;11(4):281-94.

23. Camp RL, Dolled-Filhart M, Rimm DL. X-tile: a new bio-informatics tool for biomarker assessment and outcome-based cut-point optimization. Clin Cancer Res. 2004;10(21):7252-9.

24. Zhang T, Kwiatkowski N, Olson CM, Dixon-Clarke SE, Abraham BJ, Greifenberg AK, et al. Covalent targeting of remote cysteine residues to develop CDK12 and CDK13 inhibitors. Nat Chem Biol. 2016;12(10):876-84.

1. Isakoff MS, Bielack SS, Meltzer P, Gorlick R. Osteosarcoma: Current Treatment and a Collaborative Pathway to Success. J Clin Oncol. 2015;33(27):3029-35.

3. Siegel RL, Miller KD, Jemal A. Cancer statistics, 2019. CA Cancer J Clin. 2019;69(1):7-34.

4. Ritter J, Bielack SS. Osteosarcoma. Ann Oncol. 2010;21 Suppl 7:vii320-5.

5. Gill J, Gorlick R. Advancing therapy for osteosarcoma. Nat Rev Clin Oncol. 2021;18(10):609-24.

6. Bielack SS, Kempf-Bielack B, Delling G, Exner GU, Flege S, Helmke K, et al. Prognostic factors in high-grade osteosarcoma of the extremities or trunk: an analysis of 1,702 patients treated on neoadjuvant cooperative osteosarcoma study group protocols. J Clin Oncol. 2002;20(3):776-90.

7. Han Z, Peng X, Yang Y, Yi J, Zhao D, Bao Q, et al. Integrated microfluidic-SERS for exosome biomarker profiling and osteosarcoma diagnosis. Biosens Bioelectron. 2022;217:114709.

8. Wu Y, Xu L, Yang P, Lin N, Huang X, Pan W, et al. Survival Prediction in High-grade Osteosarcoma Using Radiomics of Diagnostic Computed Tomography. EBioMedicine. 2018;34:27-34.

9. Chen W, Lin Y, Huang J, Yan Z, Cao H. A novel risk score model based on glycolysis-related genes and a prognostic model for predicting overall survival of osteosarcoma patients. J Orthop Res. 2022;40(10):2372-81.

10. Zhang M, Liu Y, Kong D. Identifying biomolecules and constructing a prognostic risk prediction model for recurrence in osteosarcoma. J Bone Oncol. 2021;26:100331.

11. Li L, Wang Y, He X, Li Z, Lu M, Gong T, et al. Hematological Prognostic Scoring System Can Predict Overall Survival and Can Indicate Response to Immunotherapy in Patients With Osteosarcoma. Front Immunol. 2022;13:879560.

12. Pelish HE, Liau BB, Nitulescu, II, Tangpeerachaikul A, Poss ZC, Da Silva DH, et al. Mediator kinase inhibition further activates super-enhancer-associated genes in AML. Nature. 2015;526(7572):273-6.

13. Whyte WA, Orlando DA, Hnisz D, Abraham BJ, Lin CY, Kagey MH, et al. Master transcription factors and mediator establish super-enhancers at key cell identity genes. Cell. 2013;153(2):307-19.

14. Tögel L, Nightingale R, Chueh AC, Jayachandran A, Tran H, Phesse T, et al. Dual Targeting of Bromodomain and Extraterminal Domain Proteins, and WNT or MAPK Signaling, Inhibits c-MYC Expression and Proliferation of Colorectal Cancer Cells. Mol Cancer Ther. 2016;15(6):1217-26.

15. Mansour MR, Abraham BJ, Anders L, Berezovskaya A, Gutierrez A, Durbin AD, et al. Oncogene regulation. An oncogenic super-enhancer formed through somatic mutation of a noncoding intergenic element. Science. 2014;346(6215):1373-7.

16. Lovén J, Hoke HA, Lin CY, Lau A, Orlando DA, Vakoc CR, et al. Selective inhibition of tumor oncogenes by disruption of super-enhancers. Cell. 2013;153(2):320-34.

17. Wang Y, Zhang T, Kwiatkowski N, Abraham BJ, Lee TI, Xie S, et al. CDK7-dependent transcriptional addiction in triple-negative breast cancer. Cell. 2015;163(1):174-86.

18. Zhang J, Liu W, Zou C, Zhao Z, Lai Y, Shi Z, et al. Targeting Super-Enhancer-Associated Oncogenes in Osteosarcoma with THZ2, a Covalent CDK7 Inhibitor. Clin Cancer Res. 2020;26(11):2681-92.

19. Xing R, Zhou Y, Yu J, Yu Y, Nie Y, Luo W, et al. Whole-genome sequencing reveals novel tandem-duplication hotspots and a prognostic mutational signature in gastric cancer. Nat Commun. 2019;10(1):2037.

20. Zou CY, Wang J, Shen JN, Huang G, Jin S, Yin JQ, et al. Establishment and characteristics of two syngeneic human osteosarcoma cell lines from primary tumor and skip metastases. Acta Pharmacol Sin. 2008;29(3):325-32.

21. Han J, Yong B, Luo C, Tan P, Peng T, Shen J. High serum alkaline phosphatase cooperating with MMP-9 predicts metastasis and poor prognosis in patients with primary osteosarcoma in Southern China. World J Surg Oncol. 2012;10:37.

22. Szulc P, Seeman E, Delmas PD. Biochemical measurements of bone turnover in children and adolescents. Osteoporos Int. 2000;11(4):281-94.

23. Camp RL, Dolled-Filhart M, Rimm DL. X-tile: a new bio-informatics tool for biomarker assessment and outcome-based cut-point optimization. Clin Cancer Res. 2004;10(21):7252-9.

24. Zhang T, Kwiatkowski N, Olson CM, Dixon-Clarke SE, Abraham BJ, Greifenberg AK, et al. Covalent targeting of remote cysteine residues to develop CDK12 and CDK13 inhibitors. Nat Chem Biol. 2016;12(10):876-84.

1. Isakoff MS, Bielack SS, Meltzer P, Gorlick R. Osteosarcoma: Current Treatment and a Collaborative Pathway to Success. J Clin Oncol. 2015;33(27):3029-35.

2. Ferrari S, Smeland S, Mercuri M, Bertoni F, Longhi A, Ruggieri P, et al. Neoadjuvant chemotherapy with high-dose Ifosfamide, high-dose methotrexate, cisplatin, and doxorubicin for patients with localized osteosarcoma of the extremity: a joint study by the Italian and Scandinavian Sarcoma Groups. J Clin Oncol. 2005;23(34):8845-52.

3. Siegel RL, Miller KD, Jemal A. Cancer statistics, 2019. CA Cancer J Clin. 2019;69(1):7-34.

4. Ritter J, Bielack SS. Osteosarcoma. Ann Oncol. 2010;21 Suppl 7:vii320-5.

5. Gill J, Gorlick R. Advancing therapy for osteosarcoma. Nat Rev Clin Oncol. 2021;18(10):609-24.

6. Bielack SS, Kempf-Bielack B, Delling G, Exner GU, Flege S, Helmke K, et al. Prognostic factors in high-grade osteosarcoma of the extremities or trunk: an analysis of 1,702 patients treated on neoadjuvant cooperative osteosarcoma study group protocols. J Clin Oncol. 2002;20(3):776-90.

7. Han Z, Peng X, Yang Y, Yi J, Zhao D, Bao Q, et al. Integrated microfluidic-SERS for exosome biomarker profiling and osteosarcoma diagnosis. Biosens Bioelectron. 2022;217:114709.

8. Wu Y, Xu L, Yang P, Lin N, Huang X, Pan W, et al. Survival Prediction in High-grade Osteosarcoma Using Radiomics of Diagnostic Computed Tomography. EBioMedicine. 2018;34:27-34.

9. Chen W, Lin Y, Huang J, Yan Z, Cao H. A novel risk score model based on glycolysis-related genes and a prognostic model for predicting overall survival of osteosarcoma patients. J Orthop Res. 2022;40(10):2372-81.

10. Zhang M, Liu Y, Kong D. Identifying biomolecules and constructing a prognostic risk prediction model for recurrence in osteosarcoma. J Bone Oncol. 2021;26:100331.

11. Li L, Wang Y, He X, Li Z, Lu M, Gong T, et al. Hematological Prognostic Scoring System Can Predict Overall Survival and Can Indicate Response to Immunotherapy in Patients With Osteosarcoma. Front Immunol. 2022;13:879560.

12. Pelish HE, Liau BB, Nitulescu, II, Tangpeerachaikul A, Poss ZC, Da Silva DH, et al. Mediator kinase inhibition further activates super-enhancer-associated genes in AML. Nature. 2015;526(7572):273-6.

13. Whyte WA, Orlando DA, Hnisz D, Abraham BJ, Lin CY, Kagey MH, et al. Master transcription factors and mediator establish super-enhancers at key cell identity genes. Cell. 2013;153(2):307-19.

14. Tögel L, Nightingale R, Chueh AC, Jayachandran A, Tran H, Phesse T, et al. Dual Targeting of Bromodomain and Extraterminal Domain Proteins, and WNT or MAPK Signaling, Inhibits c-MYC Expression and Proliferation of Colorectal Cancer Cells. Mol Cancer Ther. 2016;15(6):1217-26.

15. Mansour MR, Abraham BJ, Anders L, Berezovskaya A, Gutierrez A, Durbin AD, et al. Oncogene regulation. An oncogenic super-enhancer formed through somatic mutation of a noncoding intergenic element. Science. 2014;346(6215):1373-7.

16. Lovén J, Hoke HA, Lin CY, Lau A, Orlando DA, Vakoc CR, et al. Selective inhibition of tumor oncogenes by disruption of super-enhancers. Cell. 2013;153(2):320-34.

17. Wang Y, Zhang T, Kwiatkowski N, Abraham BJ, Lee TI, Xie S, et al. CDK7-dependent transcriptional addiction in triple-negative breast cancer. Cell. 2015;163(1):174-86.

18. Zhang J, Liu W, Zou C, Zhao Z, Lai Y, Shi Z, et al. Targeting Super-Enhancer-Associated Oncogenes in Osteosarcoma with THZ2, a Covalent CDK7 Inhibitor. Clin Cancer Res. 2020;26(11):2681-92.

19. Xing R, Zhou Y, Yu J, Yu Y, Nie Y, Luo W, et al. Whole-genome sequencing reveals novel tandem-duplication hotspots and a prognostic mutational signature in gastric cancer. Nat Commun. 2019;10(1):2037.

20. Zou CY, Wang J, Shen JN, Huang G, Jin S, Yin JQ, et al. Establishment and characteristics of two syngeneic human osteosarcoma cell lines from primary tumor and skip metastases. Acta Pharmacol Sin. 2008;29(3):325-32.

21. Han J, Yong B, Luo C, Tan P, Peng T, Shen J. High serum alkaline phosphatase cooperating with MMP-9 predicts metastasis and poor prognosis in patients with primary osteosarcoma in Southern China. World J Surg Oncol. 2012;10:37.

22. Szulc P, Seeman E, Delmas PD. Biochemical measurements of bone turnover in children and adolescents. Osteoporos Int. 2000;11(4):281-94.

23. Camp RL, Dolled-Filhart M, Rimm DL. X-tile: a new bio-informatics tool for biomarker assessment and outcome-based cut-point optimization. Clin Cancer Res. 2004;10(21):7252-9.

24. Zhang T, Kwiatkowski N, Olson CM, Dixon-Clarke SE, Abraham BJ, Greifenberg AK, et al. Covalent targeting of remote cysteine residues to develop CDK12 and CDK13 inhibitors. Nat Chem Biol. 2016;12(10):876-84.

25. Jiang YY, Lin DC, Mayakonda A, Hazawa M, Ding LW, Chien WW, et al. Targeting super-enhancer-associated oncogenes in oesophageal squamous cell carcinoma. Gut. 2017;66(8):1358-68.

1. Isakoff MS, Bielack SS, Meltzer P, Gorlick R. Osteosarcoma: Current Treatment and a Collaborative Pathway to Success. J Clin Oncol. 2015;33(27):3029-35.

2. Ferrari S, Smeland S, Mercuri M, Bertoni F, Longhi A, Ruggieri P, et al. Neoadjuvant chemotherapy with high-dose Ifosfamide, high-dose methotrexate, cisplatin, and doxorubicin for patients with localized osteosarcoma of the extremity: a joint study by the Italian and Scandinavian Sarcoma Groups. J Clin Oncol. 2005;23(34):8845-52.

3. Siegel RL, Miller KD, Jemal A. Cancer statistics, 2019. CA Cancer J Clin. 2019;69(1):7-34.

4. Ritter J, Bielack SS. Osteosarcoma. Ann Oncol. 2010;21 Suppl 7:vii320-5.

5. Gill J, Gorlick R. Advancing therapy for osteosarcoma. Nat Rev Clin Oncol. 2021;18(10):609-24.

6. Bielack SS, Kempf-Bielack B, Delling G, Exner GU, Flege S, Helmke K, et al. Prognostic factors in high-grade osteosarcoma of the extremities or trunk: an analysis of 1,702 patients treated on neoadjuvant cooperative osteosarcoma study group protocols. J Clin Oncol. 2002;20(3):776-90.

7. Han Z, Peng X, Yang Y, Yi J, Zhao D, Bao Q, et al. Integrated microfluidic-SERS for exosome biomarker profiling and osteosarcoma diagnosis. Biosens Bioelectron. 2022;217:114709.

8. Wu Y, Xu L, Yang P, Lin N, Huang X, Pan W, et al. Survival Prediction in High-grade Osteosarcoma Using Radiomics of Diagnostic Computed Tomography. EBioMedicine. 2018;34:27-34.

9. Chen W, Lin Y, Huang J, Yan Z, Cao H. A novel risk score model based on glycolysis-related genes and a prognostic model for predicting overall survival of osteosarcoma patients. J Orthop Res. 2022;40(10):2372-81.

10. Zhang M, Liu Y, Kong D. Identifying biomolecules and constructing a prognostic risk prediction model for recurrence in osteosarcoma. J Bone Oncol. 2021;26:100331.

11. Li L, Wang Y, He X, Li Z, Lu M, Gong T, et al. Hematological Prognostic Scoring System Can Predict Overall Survival and Can Indicate Response to Immunotherapy in Patients With Osteosarcoma. Front Immunol. 2022;13:879560.

12. Pelish HE, Liau BB, Nitulescu, II, Tangpeerachaikul A, Poss ZC, Da Silva DH, et al. Mediator kinase inhibition further activates super-enhancer-associated genes in AML. Nature. 2015;526(7572):273-6.

13. Whyte WA, Orlando DA, Hnisz D, Abraham BJ, Lin CY, Kagey MH, et al. Master transcription factors and mediator establish super-enhancers at key cell identity genes. Cell. 2013;153(2):307-19.

14. Tögel L, Nightingale R, Chueh AC, Jayachandran A, Tran H, Phesse T, et al. Dual Targeting of Bromodomain and Extraterminal Domain Proteins, and WNT or MAPK Signaling, Inhibits c-MYC Expression and Proliferation of Colorectal Cancer Cells. Mol Cancer Ther. 2016;15(6):1217-26.

15. Mansour MR, Abraham BJ, Anders L, Berezovskaya A, Gutierrez A, Durbin AD, et al. Oncogene regulation. An oncogenic super-enhancer formed through somatic mutation of a noncoding intergenic element. Science. 2014;346(6215):1373-7.

16. Lovén J, Hoke HA, Lin CY, Lau A, Orlando DA, Vakoc CR, et al. Selective inhibition of tumor oncogenes by disruption of super-enhancers. Cell. 2013;153(2):320-34.

17. Wang Y, Zhang T, Kwiatkowski N, Abraham BJ, Lee TI, Xie S, et al. CDK7-dependent transcriptional addiction in triple-negative breast cancer. Cell. 2015;163(1):174-86.

18. Zhang J, Liu W, Zou C, Zhao Z, Lai Y, Shi Z, et al. Targeting Super-Enhancer-Associated Oncogenes in Osteosarcoma with THZ2, a Covalent CDK7 Inhibitor. Clin Cancer Res. 2020;26(11):2681-92.

19. Xing R, Zhou Y, Yu J, Yu Y, Nie Y, Luo W, et al. Whole-genome sequencing reveals novel tandem-duplication hotspots and a prognostic mutational signature in gastric cancer. Nat Commun. 2019;10(1):2037.

20. Zou CY, Wang J, Shen JN, Huang G, Jin S, Yin JQ, et al. Establishment and characteristics of two syngeneic human osteosarcoma cell lines from primary tumor and skip metastases. Acta Pharmacol Sin. 2008;29(3):325-32.

21. Han J, Yong B, Luo C, Tan P, Peng T, Shen J. High serum alkaline phosphatase cooperating with MMP-9 predicts metastasis and poor prognosis in patients with primary osteosarcoma in Southern China. World J Surg Oncol. 2012;10:37.

22. Szulc P, Seeman E, Delmas PD. Biochemical measurements of bone turnover in children and adolescents. Osteoporos Int. 2000;11(4):281-94.

23. Camp RL, Dolled-Filhart M, Rimm DL. X-tile: a new bio-informatics tool for biomarker assessment and outcome-based cut-point optimization. Clin Cancer Res. 2004;10(21):7252-9.

24. Zhang T, Kwiatkowski N, Olson CM, Dixon-Clarke SE, Abraham BJ, Greifenberg AK, et al. Covalent targeting of remote cysteine residues to develop CDK12 and CDK13 inhibitors. Nat Chem Biol. 2016;12(10):876-84.

25. Jiang YY, Lin DC, Mayakonda A, Hazawa M, Ding LW, Chien WW, et al. Targeting super-enhancer-associated oncogenes in oesophageal squamous cell carcinoma. Gut. 2017;66(8):1358-68.

26. Meyers PA, Schwartz CL, Krailo M, Kleinerman ES, Betcher D, Bernstein ML, et al. Osteosarcoma: a randomized, prospective trial of the addition of ifosfamide and/or muramyl tripeptide to cisplatin, doxorubicin, and high-dose methotrexate. J Clin Oncol. 2005;23(9):2004-11.

1. Isakoff MS, Bielack SS, Meltzer P, Gorlick R. Osteosarcoma: Current Treatment and a Collaborative Pathway to Success. J Clin Oncol. 2015;33(27):3029-35.

2. Ferrari S, Smeland S, Mercuri M, Bertoni F, Longhi A, Ruggieri P, et al. Neoadjuvant chemotherapy with high-dose Ifosfamide, high-dose methotrexate, cisplatin, and doxorubicin for patients with localized osteosarcoma of the extremity: a joint study by the Italian and Scandinavian Sarcoma Groups. J Clin Oncol. 2005;23(34):8845-52.

3. Siegel RL, Miller KD, Jemal A. Cancer statistics, 2019. CA Cancer J Clin. 2019;69(1):7-34.

4. Ritter J, Bielack SS. Osteosarcoma. Ann Oncol. 2010;21 Suppl 7:vii320-5.

5. Gill J, Gorlick R. Advancing therapy for osteosarcoma. Nat Rev Clin Oncol. 2021;18(10):609-24.

6. Bielack SS, Kempf-Bielack B, Delling G, Exner GU, Flege S, Helmke K, et al. Prognostic factors in high-grade osteosarcoma of the extremities or trunk: an analysis of 1,702 patients treated on neoadjuvant cooperative osteosarcoma study group protocols. J Clin Oncol. 2002;20(3):776-90.

7. Han Z, Peng X, Yang Y, Yi J, Zhao D, Bao Q, et al. Integrated microfluidic-SERS for exosome biomarker profiling and osteosarcoma diagnosis. Biosens Bioelectron. 2022;217:114709.

8. Wu Y, Xu L, Yang P, Lin N, Huang X, Pan W, et al. Survival Prediction in High-grade Osteosarcoma Using Radiomics of Diagnostic Computed Tomography. EBioMedicine. 2018;34:27-34.

9. Chen W, Lin Y, Huang J, Yan Z, Cao H. A novel risk score model based on glycolysis-related genes and a prognostic model for predicting overall survival of osteosarcoma patients. J Orthop Res. 2022;40(10):2372-81.

10. Zhang M, Liu Y, Kong D. Identifying biomolecules and constructing a prognostic risk prediction model for recurrence in osteosarcoma. J Bone Oncol. 2021;26:100331.

11. Li L, Wang Y, He X, Li Z, Lu M, Gong T, et al. Hematological Prognostic Scoring System Can Predict Overall Survival and Can Indicate Response to Immunotherapy in Patients With Osteosarcoma. Front Immunol. 2022;13:879560.

12. Pelish HE, Liau BB, Nitulescu, II, Tangpeerachaikul A, Poss ZC, Da Silva DH, et al. Mediator kinase inhibition further activates super-enhancer-associated genes in AML. Nature. 2015;526(7572):273-6.

13. Whyte WA, Orlando DA, Hnisz D, Abraham BJ, Lin CY, Kagey MH, et al. Master transcription factors and mediator establish super-enhancers at key cell identity genes. Cell. 2013;153(2):307-19.

14. Tögel L, Nightingale R, Chueh AC, Jayachandran A, Tran H, Phesse T, et al. Dual Targeting of Bromodomain and Extraterminal Domain Proteins, and WNT or MAPK Signaling, Inhibits c-MYC Expression and Proliferation of Colorectal Cancer Cells. Mol Cancer Ther. 2016;15(6):1217-26.

15. Mansour MR, Abraham BJ, Anders L, Berezovskaya A, Gutierrez A, Durbin AD, et al. Oncogene regulation. An oncogenic super-enhancer formed through somatic mutation of a noncoding intergenic element. Science. 2014;346(6215):1373-7.

16. Lovén J, Hoke HA, Lin CY, Lau A, Orlando DA, Vakoc CR, et al. Selective inhibition of tumor oncogenes by disruption of super-enhancers. Cell. 2013;153(2):320-34.

17. Wang Y, Zhang T, Kwiatkowski N, Abraham BJ, Lee TI, Xie S, et al. CDK7-dependent transcriptional addiction in triple-negative breast cancer. Cell. 2015;163(1):174-86.

18. Zhang J, Liu W, Zou C, Zhao Z, Lai Y, Shi Z, et al. Targeting Super-Enhancer-Associated Oncogenes in Osteosarcoma with THZ2, a Covalent CDK7 Inhibitor. Clin Cancer Res. 2020;26(11):2681-92.

19. Xing R, Zhou Y, Yu J, Yu Y, Nie Y, Luo W, et al. Whole-genome sequencing reveals novel tandem-duplication hotspots and a prognostic mutational signature in gastric cancer. Nat Commun. 2019;10(1):2037.

20. Zou CY, Wang J, Shen JN, Huang G, Jin S, Yin JQ, et al. Establishment and characteristics of two syngeneic human osteosarcoma cell lines from primary tumor and skip metastases. Acta Pharmacol Sin. 2008;29(3):325-32.

21. Han J, Yong B, Luo C, Tan P, Peng T, Shen J. High serum alkaline phosphatase cooperating with MMP-9 predicts metastasis and poor prognosis in patients with primary osteosarcoma in Southern China. World J Surg Oncol. 2012;10:37.

22. Szulc P, Seeman E, Delmas PD. Biochemical measurements of bone turnover in children and adolescents. Osteoporos Int. 2000;11(4):281-94.

23. Camp RL, Dolled-Filhart M, Rimm DL. X-tile: a new bio-informatics tool for biomarker assessment and outcome-based cut-point optimization. Clin Cancer Res. 2004;10(21):7252-9.

24. Zhang T, Kwiatkowski N, Olson CM, Dixon-Clarke SE, Abraham BJ, Greifenberg AK, et al. Covalent targeting of remote cysteine residues to develop CDK12 and CDK13 inhibitors. Nat Chem Biol. 2016;12(10):876-84.

25. Jiang YY, Lin DC, Mayakonda A, Hazawa M, Ding LW, Chien WW, et al. Targeting super-enhancer-associated oncogenes in oesophageal squamous cell carcinoma. Gut. 2017;66(8):1358-68.

26. Meyers PA, Schwartz CL, Krailo M, Kleinerman ES, Betcher D, Bernstein ML, et al. Osteosarcoma: a randomized, prospective trial of the addition of ifosfamide and/or muramyl tripeptide to cisplatin, doxorubicin, and high-dose methotrexate. J Clin Oncol. 2005;23(9):2004-11.

27. Aksnes LH, Bauer HC, Jebsen NL, Follerås G, Allert C, Haugen GS, et al. Limb-sparing surgery preserves more function than amputation: a Scandinavian sarcoma group study of 118 patients. J Bone Joint Surg Br. 2008;90(6):786-94.

1. Isakoff MS, Bielack SS, Meltzer P, Gorlick R. Osteosarcoma: Current Treatment and a Collaborative Pathway to Success. J Clin Oncol. 2015;33(27):3029-35.

2. Ferrari S, Smeland S, Mercuri M, Bertoni F, Longhi A, Ruggieri P, et al. Neoadjuvant chemotherapy with high-dose Ifosfamide, high-dose methotrexate, cisplatin, and doxorubicin for patients with localized osteosarcoma of the extremity: a joint study by the Italian and Scandinavian Sarcoma Groups. J Clin Oncol. 2005;23(34):8845-52.

3. Siegel RL, Miller KD, Jemal A. Cancer statistics, 2019. CA Cancer J Clin. 2019;69(1):7-34.

4. Ritter J, Bielack SS. Osteosarcoma. Ann Oncol. 2010;21 Suppl 7:vii320-5.

5. Gill J, Gorlick R. Advancing therapy for osteosarcoma. Nat Rev Clin Oncol. 2021;18(10):609-24.

6. Bielack SS, Kempf-Bielack B, Delling G, Exner GU, Flege S, Helmke K, et al. Prognostic factors in high-grade osteosarcoma of the extremities or trunk: an analysis of 1,702 patients treated on neoadjuvant cooperative osteosarcoma study group protocols. J Clin Oncol. 2002;20(3):776-90.

7. Han Z, Peng X, Yang Y, Yi J, Zhao D, Bao Q, et al. Integrated microfluidic-SERS for exosome biomarker profiling and osteosarcoma diagnosis. Biosens Bioelectron. 2022;217:114709.

8. Wu Y, Xu L, Yang P, Lin N, Huang X, Pan W, et al. Survival Prediction in High-grade Osteosarcoma Using Radiomics of Diagnostic Computed Tomography. EBioMedicine. 2018;34:27-34.

9. Chen W, Lin Y, Huang J, Yan Z, Cao H. A novel risk score model based on glycolysis-related genes and a prognostic model for predicting overall survival of osteosarcoma patients. J Orthop Res. 2022;40(10):2372-81.

10. Zhang M, Liu Y, Kong D. Identifying biomolecules and constructing a prognostic risk prediction model for recurrence in osteosarcoma. J Bone Oncol. 2021;26:100331.

11. Li L, Wang Y, He X, Li Z, Lu M, Gong T, et al. Hematological Prognostic Scoring System Can Predict Overall Survival and Can Indicate Response to Immunotherapy in Patients With Osteosarcoma. Front Immunol. 2022;13:879560.

12. Pelish HE, Liau BB, Nitulescu, II, Tangpeerachaikul A, Poss ZC, Da Silva DH, et al. Mediator kinase inhibition further activates super-enhancer-associated genes in AML. Nature. 2015;526(7572):273-6.

13. Whyte WA, Orlando DA, Hnisz D, Abraham BJ, Lin CY, Kagey MH, et al. Master transcription factors and mediator establish super-enhancers at key cell identity genes. Cell. 2013;153(2):307-19.

14. Tögel L, Nightingale R, Chueh AC, Jayachandran A, Tran H, Phesse T, et al. Dual Targeting of Bromodomain and Extraterminal Domain Proteins, and WNT or MAPK Signaling, Inhibits c-MYC Expression and Proliferation of Colorectal Cancer Cells. Mol Cancer Ther. 2016;15(6):1217-26.

15. Mansour MR, Abraham BJ, Anders L, Berezovskaya A, Gutierrez A, Durbin AD, et al. Oncogene regulation. An oncogenic super-enhancer formed through somatic mutation of a noncoding intergenic element. Science. 2014;346(6215):1373-7.

16. Lovén J, Hoke HA, Lin CY, Lau A, Orlando DA, Vakoc CR, et al. Selective inhibition of tumor oncogenes by disruption of super-enhancers. Cell. 2013;153(2):320-34.

17. Wang Y, Zhang T, Kwiatkowski N, Abraham BJ, Lee TI, Xie S, et al. CDK7-dependent transcriptional addiction in triple-negative breast cancer. Cell. 2015;163(1):174-86.

18. Zhang J, Liu W, Zou C, Zhao Z, Lai Y, Shi Z, et al. Targeting Super-Enhancer-Associated Oncogenes in Osteosarcoma with THZ2, a Covalent CDK7 Inhibitor. Clin Cancer Res. 2020;26(11):2681-92.

19. Xing R, Zhou Y, Yu J, Yu Y, Nie Y, Luo W, et al. Whole-genome sequencing reveals novel tandem-duplication hotspots and a prognostic mutational signature in gastric cancer. Nat Commun. 2019;10(1):2037.

20. Zou CY, Wang J, Shen JN, Huang G, Jin S, Yin JQ, et al. Establishment and characteristics of two syngeneic human osteosarcoma cell lines from primary tumor and skip metastases. Acta Pharmacol Sin. 2008;29(3):325-32.

21. Han J, Yong B, Luo C, Tan P, Peng T, Shen J. High serum alkaline phosphatase cooperating with MMP-9 predicts metastasis and poor prognosis in patients with primary osteosarcoma in Southern China. World J Surg Oncol. 2012;10:37.

22. Szulc P, Seeman E, Delmas PD. Biochemical measurements of bone turnover in children and adolescents. Osteoporos Int. 2000;11(4):281-94.

23. Camp RL, Dolled-Filhart M, Rimm DL. X-tile: a new bio-informatics tool for biomarker assessment and outcome-based cut-point optimization. Clin Cancer Res. 2004;10(21):7252-9.

24. Zhang T, Kwiatkowski N, Olson CM, Dixon-Clarke SE, Abraham BJ, Greifenberg AK, et al. Covalent targeting of remote cysteine residues to develop CDK12 and CDK13 inhibitors. Nat Chem Biol. 2016;12(10):876-84.

25. Jiang YY, Lin DC, Mayakonda A, Hazawa M, Ding LW, Chien WW, et al. Targeting super-enhancer-associated oncogenes in oesophageal squamous cell carcinoma. Gut. 2017;66(8):1358-68.

26. Meyers PA, Schwartz CL, Krailo M, Kleinerman ES, Betcher D, Bernstein ML, et al. Osteosarcoma: a randomized, prospective trial of the addition of ifosfamide and/or muramyl tripeptide to cisplatin, doxorubicin, and high-dose methotrexate. J Clin Oncol. 2005;23(9):2004-11.

27. Aksnes LH, Bauer HC, Jebsen NL, Follerås G, Allert C, Haugen GS, et al. Limb-sparing surgery preserves more function than amputation: a Scandinavian sarcoma group study of 118 patients. J Bone Joint Surg Br. 2008;90(6):786-94.

28. Winkler K, Beron G, Kotz R, Salzer-Kuntschik M, Beck J, Beck W, et al. Neoadjuvant chemotherapy for osteogenic sarcoma: results of a Cooperative German/Austrian study. J Clin Oncol. 1984;2(6):617-24.

1. Isakoff MS, Bielack SS, Meltzer P, Gorlick R. Osteosarcoma: Current Treatment and a Collaborative Pathway to Success. J Clin Oncol. 2015;33(27):3029-35.

2. Ferrari S, Smeland S, Mercuri M, Bertoni F, Longhi A, Ruggieri P, et al. Neoadjuvant chemotherapy with high-dose Ifosfamide, high-dose methotrexate, cisplatin, and doxorubicin for patients with localized osteosarcoma of the extremity: a joint study by the Italian and Scandinavian Sarcoma Groups. J Clin Oncol. 2005;23(34):8845-52.

3. Siegel RL, Miller KD, Jemal A. Cancer statistics, 2019. CA Cancer J Clin. 2019;69(1):7-34.

4. Ritter J, Bielack SS. Osteosarcoma. Ann Oncol. 2010;21 Suppl 7:vii320-5.

5. Gill J, Gorlick R. Advancing therapy for osteosarcoma. Nat Rev Clin Oncol. 2021;18(10):609-24.

6. Bielack SS, Kempf-Bielack B, Delling G, Exner GU, Flege S, Helmke K, et al. Prognostic factors in high-grade osteosarcoma of the extremities or trunk: an analysis of 1,702 patients treated on neoadjuvant cooperative osteosarcoma study group protocols. J Clin Oncol. 2002;20(3):776-90.

7. Han Z, Peng X, Yang Y, Yi J, Zhao D, Bao Q, et al. Integrated microfluidic-SERS for exosome biomarker profiling and osteosarcoma diagnosis. Biosens Bioelectron. 2022;217:114709.

8. Wu Y, Xu L, Yang P, Lin N, Huang X, Pan W, et al. Survival Prediction in High-grade Osteosarcoma Using Radiomics of Diagnostic Computed Tomography. EBioMedicine. 2018;34:27-34.

9. Chen W, Lin Y, Huang J, Yan Z, Cao H. A novel risk score model based on glycolysis-related genes and a prognostic model for predicting overall survival of osteosarcoma patients. J Orthop Res. 2022;40(10):2372-81.

10. Zhang M, Liu Y, Kong D. Identifying biomolecules and constructing a prognostic risk prediction model for recurrence in osteosarcoma. J Bone Oncol. 2021;26:100331.

11. Li L, Wang Y, He X, Li Z, Lu M, Gong T, et al. Hematological Prognostic Scoring System Can Predict Overall Survival and Can Indicate Response to Immunotherapy in Patients With Osteosarcoma. Front Immunol. 2022;13:879560.

12. Pelish HE, Liau BB, Nitulescu, II, Tangpeerachaikul A, Poss ZC, Da Silva DH, et al. Mediator kinase inhibition further activates super-enhancer-associated genes in AML. Nature. 2015;526(7572):273-6.

13. Whyte WA, Orlando DA, Hnisz D, Abraham BJ, Lin CY, Kagey MH, et al. Master transcription factors and mediator establish super-enhancers at key cell identity genes. Cell. 2013;153(2):307-19.

14. Tögel L, Nightingale R, Chueh AC, Jayachandran A, Tran H, Phesse T, et al. Dual Targeting of Bromodomain and Extraterminal Domain Proteins, and WNT or MAPK Signaling, Inhibits c-MYC Expression and Proliferation of Colorectal Cancer Cells. Mol Cancer Ther. 2016;15(6):1217-26.

15. Mansour MR, Abraham BJ, Anders L, Berezovskaya A, Gutierrez A, Durbin AD, et al. Oncogene regulation. An oncogenic super-enhancer formed through somatic mutation of a noncoding intergenic element. Science. 2014;346(6215):1373-7.

16. Lovén J, Hoke HA, Lin CY, Lau A, Orlando DA, Vakoc CR, et al. Selective inhibition of tumor oncogenes by disruption of super-enhancers. Cell. 2013;153(2):320-34.

17. Wang Y, Zhang T, Kwiatkowski N, Abraham BJ, Lee TI, Xie S, et al. CDK7-dependent transcriptional addiction in triple-negative breast cancer. Cell. 2015;163(1):174-86.

18. Zhang J, Liu W, Zou C, Zhao Z, Lai Y, Shi Z, et al. Targeting Super-Enhancer-Associated Oncogenes in Osteosarcoma with THZ2, a Covalent CDK7 Inhibitor. Clin Cancer Res. 2020;26(11):2681-92.

19. Xing R, Zhou Y, Yu J, Yu Y, Nie Y, Luo W, et al. Whole-genome sequencing reveals novel tandem-duplication hotspots and a prognostic mutational signature in gastric cancer. Nat Commun. 2019;10(1):2037.

20. Zou CY, Wang J, Shen JN, Huang G, Jin S, Yin JQ, et al. Establishment and characteristics of two syngeneic human osteosarcoma cell lines from primary tumor and skip metastases. Acta Pharmacol Sin. 2008;29(3):325-32.

21. Han J, Yong B, Luo C, Tan P, Peng T, Shen J. High serum alkaline phosphatase cooperating with MMP-9 predicts metastasis and poor prognosis in patients with primary osteosarcoma in Southern China. World J Surg Oncol. 2012;10:37.

22. Szulc P, Seeman E, Delmas PD. Biochemical measurements of bone turnover in children and adolescents. Osteoporos Int. 2000;11(4):281-94.

23. Camp RL, Dolled-Filhart M, Rimm DL. X-tile: a new bio-informatics tool for biomarker assessment and outcome-based cut-point optimization. Clin Cancer Res. 2004;10(21):7252-9.

24. Zhang T, Kwiatkowski N, Olson CM, Dixon-Clarke SE, Abraham BJ, Greifenberg AK, et al. Covalent targeting of remote cysteine residues to develop CDK12 and CDK13 inhibitors. Nat Chem Biol. 2016;12(10):876-84.

25. Jiang YY, Lin DC, Mayakonda A, Hazawa M, Ding LW, Chien WW, et al. Targeting super-enhancer-associated oncogenes in oesophageal squamous cell carcinoma. Gut. 2017;66(8):1358-68.

26. Meyers PA, Schwartz CL, Krailo M, Kleinerman ES, Betcher D, Bernstein ML, et al. Osteosarcoma: a randomized, prospective trial of the addition of ifosfamide and/or muramyl tripeptide to cisplatin, doxorubicin, and high-dose methotrexate. J Clin Oncol. 2005;23(9):2004-11.

27. Aksnes LH, Bauer HC, Jebsen NL, Follerås G, Allert C, Haugen GS, et al. Limb-sparing surgery preserves more function than amputation: a Scandinavian sarcoma group study of 118 patients. J Bone Joint Surg Br. 2008;90(6):786-94.

28. Winkler K, Beron G, Kotz R, Salzer-Kuntschik M, Beck J, Beck W, et al. Neoadjuvant chemotherapy for osteogenic sarcoma: results of a Cooperative German/Austrian study. J Clin Oncol. 1984;2(6):617-24.

1. Isakoff MS, Bielack SS, Meltzer P, Gorlick R. Osteosarcoma: Current Treatment and a Collaborative Pathway to Success. J Clin Oncol. 2015;33(27):3029-35.

2. Ferrari S, Smeland S, Mercuri M, Bertoni F, Longhi A, Ruggieri P, et al. Neoadjuvant chemotherapy with high-dose Ifosfamide, high-dose methotrexate, cisplatin, and doxorubicin for patients with localized osteosarcoma of the extremity: a joint study by the Italian and Scandinavian Sarcoma Groups. J Clin Oncol. 2005;23(34):8845-52.

3. Siegel RL, Miller KD, Jemal A. Cancer statistics, 2019. CA Cancer J Clin. 2019;69(1):7-34.

4. Ritter J, Bielack SS. Osteosarcoma. Ann Oncol. 2010;21 Suppl 7:vii320-5.

5. Gill J, Gorlick R. Advancing therapy for osteosarcoma. Nat Rev Clin Oncol. 2021;18(10):609-24.

6. Bielack SS, Kempf-Bielack B, Delling G, Exner GU, Flege S, Helmke K, et al. Prognostic factors in high-grade osteosarcoma of the extremities or trunk: an analysis of 1,702 patients treated on neoadjuvant cooperative osteosarcoma study group protocols. J Clin Oncol. 2002;20(3):776-90.

7. Han Z, Peng X, Yang Y, Yi J, Zhao D, Bao Q, et al. Integrated microfluidic-SERS for exosome biomarker profiling and osteosarcoma diagnosis. Biosens Bioelectron. 2022;217:114709.

8. Wu Y, Xu L, Yang P, Lin N, Huang X, Pan W, et al. Survival Prediction in High-grade Osteosarcoma Using Radiomics of Diagnostic Computed Tomography. EBioMedicine. 2018;34:27-34.

9. Chen W, Lin Y, Huang J, Yan Z, Cao H. A novel risk score model based on glycolysis-related genes and a prognostic model for predicting overall survival of osteosarcoma patients. J Orthop Res. 2022;40(10):2372-81.

10. Zhang M, Liu Y, Kong D. Identifying biomolecules and constructing a prognostic risk prediction model for recurrence in osteosarcoma. J Bone Oncol. 2021;26:100331.

11. Li L, Wang Y, He X, Li Z, Lu M, Gong T, et al. Hematological Prognostic Scoring System Can Predict Overall Survival and Can Indicate Response to Immunotherapy in Patients With Osteosarcoma. Front Immunol. 2022;13:879560.

12. Pelish HE, Liau BB, Nitulescu, II, Tangpeerachaikul A, Poss ZC, Da Silva DH, et al. Mediator kinase inhibition further activates super-enhancer-associated genes in AML. Nature. 2015;526(7572):273-6.

13. Whyte WA, Orlando DA, Hnisz D, Abraham BJ, Lin CY, Kagey MH, et al. Master transcription factors and mediator establish super-enhancers at key cell identity genes. Cell. 2013;153(2):307-19.

14. Tögel L, Nightingale R, Chueh AC, Jayachandran A, Tran H, Phesse T, et al. Dual Targeting of Bromodomain and Extraterminal Domain Proteins, and WNT or MAPK Signaling, Inhibits c-MYC Expression and Proliferation of Colorectal Cancer Cells. Mol Cancer Ther. 2016;15(6):1217-26.

15. Mansour MR, Abraham BJ, Anders L, Berezovskaya A, Gutierrez A, Durbin AD, et al. Oncogene regulation. An oncogenic super-enhancer formed through somatic mutation of a noncoding intergenic element. Science. 2014;346(6215):1373-7.

16. Lovén J, Hoke HA, Lin CY, Lau A, Orlando DA, Vakoc CR, et al. Selective inhibition of tumor oncogenes by disruption of super-enhancers. Cell. 2013;153(2):320-34.

17. Wang Y, Zhang T, Kwiatkowski N, Abraham BJ, Lee TI, Xie S, et al. CDK7-dependent transcriptional addiction in triple-negative breast cancer. Cell. 2015;163(1):174-86.

18. Zhang J, Liu W, Zou C, Zhao Z, Lai Y, Shi Z, et al. Targeting Super-Enhancer-Associated Oncogenes in Osteosarcoma with THZ2, a Covalent CDK7 Inhibitor. Clin Cancer Res. 2020;26(11):2681-92.

19. Xing R, Zhou Y, Yu J, Yu Y, Nie Y, Luo W, et al. Whole-genome sequencing reveals novel tandem-duplication hotspots and a prognostic mutational signature in gastric cancer. Nat Commun. 2019;10(1):2037.

20. Zou CY, Wang J, Shen JN, Huang G, Jin S, Yin JQ, et al. Establishment and characteristics of two syngeneic human osteosarcoma cell lines from primary tumor and skip metastases. Acta Pharmacol Sin. 2008;29(3):325-32.

21. Han J, Yong B, Luo C, Tan P, Peng T, Shen J. High serum alkaline phosphatase cooperating with MMP-9 predicts metastasis and poor prognosis in patients with primary osteosarcoma in Southern China. World J Surg Oncol. 2012;10:37.

22. Szulc P, Seeman E, Delmas PD. Biochemical measurements of bone turnover in children and adolescents. Osteoporos Int. 2000;11(4):281-94.

23. Camp RL, Dolled-Filhart M, Rimm DL. X-tile: a new bio-informatics tool for biomarker assessment and outcome-based cut-point optimization. Clin Cancer Res. 2004;10(21):7252-9.

24. Zhang T, Kwiatkowski N, Olson CM, Dixon-Clarke SE, Abraham BJ, Greifenberg AK, et al. Covalent targeting of remote cysteine residues to develop CDK12 and CDK13 inhibitors. Nat Chem Biol. 2016;12(10):876-84.

25. Jiang YY, Lin DC, Mayakonda A, Hazawa M, Ding LW, Chien WW, et al. Targeting super-enhancer-associated oncogenes in oesophageal squamous cell carcinoma. Gut. 2017;66(8):1358-68.

26. Meyers PA, Schwartz CL, Krailo M, Kleinerman ES, Betcher D, Bernstein ML, et al. Osteosarcoma: a randomized, prospective trial of the addition of ifosfamide and/or muramyl tripeptide to cisplatin, doxorubicin, and high-dose methotrexate. J Clin Oncol. 2005;23(9):2004-11.

27. Aksnes LH, Bauer HC, Jebsen NL, Follerås G, Allert C, Haugen GS, et al. Limb-sparing surgery preserves more function than amputation: a Scandinavian sarcoma group study of 118 patients. J Bone Joint Surg Br. 2008;90(6):786-94.

28. Winkler K, Beron G, Kotz R, Salzer-Kuntschik M, Beck J, Beck W, et al. Neoadjuvant chemotherapy for osteogenic sarcoma: results of a Cooperative German/Austrian study. J Clin Oncol. 1984;2(6):617-24.

29. Polites SF, Heaton TE, LaQuaglia MP, Kim ES, Barry WE, Goodhue CJ, et al. Pneumonectomy for Pediatric Tumors-a Pediatric Surgical Oncology Research Collaborative Study. Ann Surg. 2021;274(6):e605-e9.

1. Isakoff MS, Bielack SS, Meltzer P, Gorlick R. Osteosarcoma: Current Treatment and a Collaborative Pathway to Success. J Clin Oncol. 2015;33(27):3029-35.

2. Ferrari S, Smeland S, Mercuri M, Bertoni F, Longhi A, Ruggieri P, et al. Neoadjuvant chemotherapy with high-dose Ifosfamide, high-dose methotrexate, cisplatin, and doxorubicin for patients with localized osteosarcoma of the extremity: a joint study by the Italian and Scandinavian Sarcoma Groups. J Clin Oncol. 2005;23(34):8845-52.

3. Siegel RL, Miller KD, Jemal A. Cancer statistics, 2019. CA Cancer J Clin. 2019;69(1):7-34.

4. Ritter J, Bielack SS. Osteosarcoma. Ann Oncol. 2010;21 Suppl 7:vii320-5.

5. Gill J, Gorlick R. Advancing therapy for osteosarcoma. Nat Rev Clin Oncol. 2021;18(10):609-24.

6. Bielack SS, Kempf-Bielack B, Delling G, Exner GU, Flege S, Helmke K, et al. Prognostic factors in high-grade osteosarcoma of the extremities or trunk: an analysis of 1,702 patients treated on neoadjuvant cooperative osteosarcoma study group protocols. J Clin Oncol. 2002;20(3):776-90.

7. Han Z, Peng X, Yang Y, Yi J, Zhao D, Bao Q, et al. Integrated microfluidic-SERS for exosome biomarker profiling and osteosarcoma diagnosis. Biosens Bioelectron. 2022;217:114709.

8. Wu Y, Xu L, Yang P, Lin N, Huang X, Pan W, et al. Survival Prediction in High-grade Osteosarcoma Using Radiomics of Diagnostic Computed Tomography. EBioMedicine. 2018;34:27-34.

9. Chen W, Lin Y, Huang J, Yan Z, Cao H. A novel risk score model based on glycolysis-related genes and a prognostic model for predicting overall survival of osteosarcoma patients. J Orthop Res. 2022;40(10):2372-81.

10. Zhang M, Liu Y, Kong D. Identifying biomolecules and constructing a prognostic risk prediction model for recurrence in osteosarcoma. J Bone Oncol. 2021;26:100331.

11. Li L, Wang Y, He X, Li Z, Lu M, Gong T, et al. Hematological Prognostic Scoring System Can Predict Overall Survival and Can Indicate Response to Immunotherapy in Patients With Osteosarcoma. Front Immunol. 2022;13:879560.

12. Pelish HE, Liau BB, Nitulescu, II, Tangpeerachaikul A, Poss ZC, Da Silva DH, et al. Mediator kinase inhibition further activates super-enhancer-associated genes in AML. Nature. 2015;526(7572):273-6.

13. Whyte WA, Orlando DA, Hnisz D, Abraham BJ, Lin CY, Kagey MH, et al. Master transcription factors and mediator establish super-enhancers at key cell identity genes. Cell. 2013;153(2):307-19.

14. Tögel L, Nightingale R, Chueh AC, Jayachandran A, Tran H, Phesse T, et al. Dual Targeting of Bromodomain and Extraterminal Domain Proteins, and WNT or MAPK Signaling, Inhibits c-MYC Expression and Proliferation of Colorectal Cancer Cells. Mol Cancer Ther. 2016;15(6):1217-26.

15. Mansour MR, Abraham BJ, Anders L, Berezovskaya A, Gutierrez A, Durbin AD, et al. Oncogene regulation. An oncogenic super-enhancer formed through somatic mutation of a noncoding intergenic element. Science. 2014;346(6215):1373-7.

16. Lovén J, Hoke HA, Lin CY, Lau A, Orlando DA, Vakoc CR, et al. Selective inhibition of tumor oncogenes by disruption of super-enhancers. Cell. 2013;153(2):320-34.

17. Wang Y, Zhang T, Kwiatkowski N, Abraham BJ, Lee TI, Xie S, et al. CDK7-dependent transcriptional addiction in triple-negative breast cancer. Cell. 2015;163(1):174-86.

18. Zhang J, Liu W, Zou C, Zhao Z, Lai Y, Shi Z, et al. Targeting Super-Enhancer-Associated Oncogenes in Osteosarcoma with THZ2, a Covalent CDK7 Inhibitor. Clin Cancer Res. 2020;26(11):2681-92.

19. Xing R, Zhou Y, Yu J, Yu Y, Nie Y, Luo W, et al. Whole-genome sequencing reveals novel tandem-duplication hotspots and a prognostic mutational signature in gastric cancer. Nat Commun. 2019;10(1):2037.

20. Zou CY, Wang J, Shen JN, Huang G, Jin S, Yin JQ, et al. Establishment and characteristics of two syngeneic human osteosarcoma cell lines from primary tumor and skip metastases. Acta Pharmacol Sin. 2008;29(3):325-32.

21. Han J, Yong B, Luo C, Tan P, Peng T, Shen J. High serum alkaline phosphatase cooperating with MMP-9 predicts metastasis and poor prognosis in patients with primary osteosarcoma in Southern China. World J Surg Oncol. 2012;10:37.

22. Szulc P, Seeman E, Delmas PD. Biochemical measurements of bone turnover in children and adolescents. Osteoporos Int. 2000;11(4):281-94.

23. Camp RL, Dolled-Filhart M, Rimm DL. X-tile: a new bio-informatics tool for biomarker assessment and outcome-based cut-point optimization. Clin Cancer Res. 2004;10(21):7252-9.

24. Zhang T, Kwiatkowski N, Olson CM, Dixon-Clarke SE, Abraham BJ, Greifenberg AK, et al. Covalent targeting of remote cysteine residues to develop CDK12 and CDK13 inhibitors. Nat Chem Biol. 2016;12(10):876-84.

25. Jiang YY, Lin DC, Mayakonda A, Hazawa M, Ding LW, Chien WW, et al. Targeting super-enhancer-associated oncogenes in oesophageal squamous cell carcinoma. Gut. 2017;66(8):1358-68.

26. Meyers PA, Schwartz CL, Krailo M, Kleinerman ES, Betcher D, Bernstein ML, et al. Osteosarcoma: a randomized, prospective trial of the addition of ifosfamide and/or muramyl tripeptide to cisplatin, doxorubicin, and high-dose methotrexate. J Clin Oncol. 2005;23(9):2004-11.

27. Aksnes LH, Bauer HC, Jebsen NL, Follerås G, Allert C, Haugen GS, et al. Limb-sparing surgery preserves more function than amputation: a Scandinavian sarcoma group study of 118 patients. J Bone Joint Surg Br. 2008;90(6):786-94.

28. Winkler K, Beron G, Kotz R, Salzer-Kuntschik M, Beck J, Beck W, et al. Neoadjuvant chemotherapy for osteogenic sarcoma: results of a Cooperative German/Austrian study. J Clin Oncol. 1984;2(6):617-24.

29. Polites SF, Heaton TE, LaQuaglia MP, Kim ES, Barry WE, Goodhue CJ, et al. Pneumonectomy for Pediatric Tumors-a Pediatric Surgical Oncology Research Collaborative Study. Ann Surg. 2021;274(6):e605-e9.

30. Bacci G, Ferrari S, Lari S, Mercuri M, Donati D, Longhi A, et al. Osteosarcoma of the limb. Amputation or limb salvage in patients treated by neoadjuvant chemotherapy. J Bone Joint Surg Br. 2002;84(1):88-92.

1. Isakoff MS, Bielack SS, Meltzer P, Gorlick R. Osteosarcoma: Current Treatment and a Collaborative Pathway to Success. J Clin Oncol. 2015;33(27):3029-35.

2. Ferrari S, Smeland S, Mercuri M, Bertoni F, Longhi A, Ruggieri P, et al. Neoadjuvant chemotherapy with high-dose Ifosfamide, high-dose methotrexate, cisplatin, and doxorubicin for patients with localized osteosarcoma of the extremity: a joint study by the Italian and Scandinavian Sarcoma Groups. J Clin Oncol. 2005;23(34):8845-52.

3. Siegel RL, Miller KD, Jemal A. Cancer statistics, 2019. CA Cancer J Clin. 2019;69(1):7-34.

4. Ritter J, Bielack SS. Osteosarcoma. Ann Oncol. 2010;21 Suppl 7:vii320-5.

5. Gill J, Gorlick R. Advancing therapy for osteosarcoma. Nat Rev Clin Oncol. 2021;18(10):609-24.

6. Bielack SS, Kempf-Bielack B, Delling G, Exner GU, Flege S, Helmke K, et al. Prognostic factors in high-grade osteosarcoma of the extremities or trunk: an analysis of 1,702 patients treated on neoadjuvant cooperative osteosarcoma study group protocols. J Clin Oncol. 2002;20(3):776-90.

7. Han Z, Peng X, Yang Y, Yi J, Zhao D, Bao Q, et al. Integrated microfluidic-SERS for exosome biomarker profiling and osteosarcoma diagnosis. Biosens Bioelectron. 2022;217:114709.

8. Wu Y, Xu L, Yang P, Lin N, Huang X, Pan W, et al. Survival Prediction in High-grade Osteosarcoma Using Radiomics of Diagnostic Computed Tomography. EBioMedicine. 2018;34:27-34.

9. Chen W, Lin Y, Huang J, Yan Z, Cao H. A novel risk score model based on glycolysis-related genes and a prognostic model for predicting overall survival of osteosarcoma patients. J Orthop Res. 2022;40(10):2372-81.

10. Zhang M, Liu Y, Kong D. Identifying biomolecules and constructing a prognostic risk prediction model for recurrence in osteosarcoma. J Bone Oncol. 2021;26:100331.

11. Li L, Wang Y, He X, Li Z, Lu M, Gong T, et al. Hematological Prognostic Scoring System Can Predict Overall Survival and Can Indicate Response to Immunotherapy in Patients With Osteosarcoma. Front Immunol. 2022;13:879560.

12. Pelish HE, Liau BB, Nitulescu, II, Tangpeerachaikul A, Poss ZC, Da Silva DH, et al. Mediator kinase inhibition further activates super-enhancer-associated genes in AML. Nature. 2015;526(7572):273-6.

13. Whyte WA, Orlando DA, Hnisz D, Abraham BJ, Lin CY, Kagey MH, et al. Master transcription factors and mediator establish super-enhancers at key cell identity genes. Cell. 2013;153(2):307-19.

14. Tögel L, Nightingale R, Chueh AC, Jayachandran A, Tran H, Phesse T, et al. Dual Targeting of Bromodomain and Extraterminal Domain Proteins, and WNT or MAPK Signaling, Inhibits c-MYC Expression and Proliferation of Colorectal Cancer Cells. Mol Cancer Ther. 2016;15(6):1217-26.

15. Mansour MR, Abraham BJ, Anders L, Berezovskaya A, Gutierrez A, Durbin AD, et al. Oncogene regulation. An oncogenic super-enhancer formed through somatic mutation of a noncoding intergenic element. Science. 2014;346(6215):1373-7.

16. Lovén J, Hoke HA, Lin CY, Lau A, Orlando DA, Vakoc CR, et al. Selective inhibition of tumor oncogenes by disruption of super-enhancers. Cell. 2013;153(2):320-34.

17. Wang Y, Zhang T, Kwiatkowski N, Abraham BJ, Lee TI, Xie S, et al. CDK7-dependent transcriptional addiction in triple-negative breast cancer. Cell. 2015;163(1):174-86.

18. Zhang J, Liu W, Zou C, Zhao Z, Lai Y, Shi Z, et al. Targeting Super-Enhancer-Associated Oncogenes in Osteosarcoma with THZ2, a Covalent CDK7 Inhibitor. Clin Cancer Res. 2020;26(11):2681-92.

19. Xing R, Zhou Y, Yu J, Yu Y, Nie Y, Luo W, et al. Whole-genome sequencing reveals novel tandem-duplication hotspots and a prognostic mutational signature in gastric cancer. Nat Commun. 2019;10(1):2037.

20. Zou CY, Wang J, Shen JN, Huang G, Jin S, Yin JQ, et al. Establishment and characteristics of two syngeneic human osteosarcoma cell lines from primary tumor and skip metastases. Acta Pharmacol Sin. 2008;29(3):325-32.

21. Han J, Yong B, Luo C, Tan P, Peng T, Shen J. High serum alkaline phosphatase cooperating with MMP-9 predicts metastasis and poor prognosis in patients with primary osteosarcoma in Southern China. World J Surg Oncol. 2012;10:37.

22. Szulc P, Seeman E, Delmas PD. Biochemical measurements of bone turnover in children and adolescents. Osteoporos Int. 2000;11(4):281-94.

23. Camp RL, Dolled-Filhart M, Rimm DL. X-tile: a new bio-informatics tool for biomarker assessment and outcome-based cut-point optimization. Clin Cancer Res. 2004;10(21):7252-9.

24. Zhang T, Kwiatkowski N, Olson CM, Dixon-Clarke SE, Abraham BJ, Greifenberg AK, et al. Covalent targeting of remote cysteine residues to develop CDK12 and CDK13 inhibitors. Nat Chem Biol. 2016;12(10):876-84.

25. Jiang YY, Lin DC, Mayakonda A, Hazawa M, Ding LW, Chien WW, et al. Targeting super-enhancer-associated oncogenes in oesophageal squamous cell carcinoma. Gut. 2017;66(8):1358-68.

26. Meyers PA, Schwartz CL, Krailo M, Kleinerman ES, Betcher D, Bernstein ML, et al. Osteosarcoma: a randomized, prospective trial of the addition of ifosfamide and/or muramyl tripeptide to cisplatin, doxorubicin, and high-dose methotrexate. J Clin Oncol. 2005;23(9):2004-11.

27. Aksnes LH, Bauer HC, Jebsen NL, Follerås G, Allert C, Haugen GS, et al. Limb-sparing surgery preserves more function than amputation: a Scandinavian sarcoma group study of 118 patients. J Bone Joint Surg Br. 2008;90(6):786-94.

28. Winkler K, Beron G, Kotz R, Salzer-Kuntschik M, Beck J, Beck W, et al. Neoadjuvant chemotherapy for osteogenic sarcoma: results of a Cooperative German/Austrian study. J Clin Oncol. 1984;2(6):617-24.

29. Polites SF, Heaton TE, LaQuaglia MP, Kim ES, Barry WE, Goodhue CJ, et al. Pneumonectomy for Pediatric Tumors-a Pediatric Surgical Oncology Research Collaborative Study. Ann Surg. 2021;274(6):e605-e9.

30. Bacci G, Ferrari S, Lari S, Mercuri M, Donati D, Longhi A, et al. Osteosarcoma of the limb. Amputation or limb salvage in patients treated by neoadjuvant chemotherapy. J Bone Joint Surg Br. 2002;84(1):88-92.

1. Isakoff MS, Bielack SS, Meltzer P, Gorlick R. Osteosarcoma: Current Treatment and a Collaborative Pathway to Success. J Clin Oncol. 2015;33(27):3029-35.

2. Ferrari S, Smeland S, Mercuri M, Bertoni F, Longhi A, Ruggieri P, et al. Neoadjuvant chemotherapy with high-dose Ifosfamide, high-dose methotrexate, cisplatin, and doxorubicin for patients with localized osteosarcoma of the extremity: a joint study by the Italian and Scandinavian Sarcoma Groups. J Clin Oncol. 2005;23(34):8845-52.

3. Siegel RL, Miller KD, Jemal A. Cancer statistics, 2019. CA Cancer J Clin. 2019;69(1):7-34.

4. Ritter J, Bielack SS. Osteosarcoma. Ann Oncol. 2010;21 Suppl 7:vii320-5.

5. Gill J, Gorlick R. Advancing therapy for osteosarcoma. Nat Rev Clin Oncol. 2021;18(10):609-24.

6. Bielack SS, Kempf-Bielack B, Delling G, Exner GU, Flege S, Helmke K, et al. Prognostic factors in high-grade osteosarcoma of the extremities or trunk: an analysis of 1,702 patients treated on neoadjuvant cooperative osteosarcoma study group protocols. J Clin Oncol. 2002;20(3):776-90.

7. Han Z, Peng X, Yang Y, Yi J, Zhao D, Bao Q, et al. Integrated microfluidic-SERS for exosome biomarker profiling and osteosarcoma diagnosis. Biosens Bioelectron. 2022;217:114709.

8. Wu Y, Xu L, Yang P, Lin N, Huang X, Pan W, et al. Survival Prediction in High-grade Osteosarcoma Using Radiomics of Diagnostic Computed Tomography. EBioMedicine. 2018;34:27-34.

9. Chen W, Lin Y, Huang J, Yan Z, Cao H. A novel risk score model based on glycolysis-related genes and a prognostic model for predicting overall survival of osteosarcoma patients. J Orthop Res. 2022;40(10):2372-81.

10. Zhang M, Liu Y, Kong D. Identifying biomolecules and constructing a prognostic risk prediction model for recurrence in osteosarcoma. J Bone Oncol. 2021;26:100331.

11. Li L, Wang Y, He X, Li Z, Lu M, Gong T, et al. Hematological Prognostic Scoring System Can Predict Overall Survival and Can Indicate Response to Immunotherapy in Patients With Osteosarcoma. Front Immunol. 2022;13:879560.

12. Pelish HE, Liau BB, Nitulescu, II, Tangpeerachaikul A, Poss ZC, Da Silva DH, et al. Mediator kinase inhibition further activates super-enhancer-associated genes in AML. Nature. 2015;526(7572):273-6.

13. Whyte WA, Orlando DA, Hnisz D, Abraham BJ, Lin CY, Kagey MH, et al. Master transcription factors and mediator establish super-enhancers at key cell identity genes. Cell. 2013;153(2):307-19.

14. Tögel L, Nightingale R, Chueh AC, Jayachandran A, Tran H, Phesse T, et al. Dual Targeting of Bromodomain and Extraterminal Domain Proteins, and WNT or MAPK Signaling, Inhibits c-MYC Expression and Proliferation of Colorectal Cancer Cells. Mol Cancer Ther. 2016;15(6):1217-26.

15. Mansour MR, Abraham BJ, Anders L, Berezovskaya A, Gutierrez A, Durbin AD, et al. Oncogene regulation. An oncogenic super-enhancer formed through somatic mutation of a noncoding intergenic element. Science. 2014;346(6215):1373-7.

16. Lovén J, Hoke HA, Lin CY, Lau A, Orlando DA, Vakoc CR, et al. Selective inhibition of tumor oncogenes by disruption of super-enhancers. Cell. 2013;153(2):320-34.

17. Wang Y, Zhang T, Kwiatkowski N, Abraham BJ, Lee TI, Xie S, et al. CDK7-dependent transcriptional addiction in triple-negative breast cancer. Cell. 2015;163(1):174-86.

18. Zhang J, Liu W, Zou C, Zhao Z, Lai Y, Shi Z, et al. Targeting Super-Enhancer-Associated Oncogenes in Osteosarcoma with THZ2, a Covalent CDK7 Inhibitor. Clin Cancer Res. 2020;26(11):2681-92.

19. Xing R, Zhou Y, Yu J, Yu Y, Nie Y, Luo W, et al. Whole-genome sequencing reveals novel tandem-duplication hotspots and a prognostic mutational signature in gastric cancer. Nat Commun. 2019;10(1):2037.

20. Zou CY, Wang J, Shen JN, Huang G, Jin S, Yin JQ, et al. Establishment and characteristics of two syngeneic human osteosarcoma cell lines from primary tumor and skip metastases. Acta Pharmacol Sin. 2008;29(3):325-32.

21. Han J, Yong B, Luo C, Tan P, Peng T, Shen J. High serum alkaline phosphatase cooperating with MMP-9 predicts metastasis and poor prognosis in patients with primary osteosarcoma in Southern China. World J Surg Oncol. 2012;10:37.

22. Szulc P, Seeman E, Delmas PD. Biochemical measurements of bone turnover in children and adolescents. Osteoporos Int. 2000;11(4):281-94.

23. Camp RL, Dolled-Filhart M, Rimm DL. X-tile: a new bio-informatics tool for biomarker assessment and outcome-based cut-point optimization. Clin Cancer Res. 2004;10(21):7252-9.

24. Zhang T, Kwiatkowski N, Olson CM, Dixon-Clarke SE, Abraham BJ, Greifenberg AK, et al. Covalent targeting of remote cysteine residues to develop CDK12 and CDK13 inhibitors. Nat Chem Biol. 2016;12(10):876-84.

25. Jiang YY, Lin DC, Mayakonda A, Hazawa M, Ding LW, Chien WW, et al. Targeting super-enhancer-associated oncogenes in oesophageal squamous cell carcinoma. Gut. 2017;66(8):1358-68.

26. Meyers PA, Schwartz CL, Krailo M, Kleinerman ES, Betcher D, Bernstein ML, et al. Osteosarcoma: a randomized, prospective trial of the addition of ifosfamide and/or muramyl tripeptide to cisplatin, doxorubicin, and high-dose methotrexate. J Clin Oncol. 2005;23(9):2004-11.

27. Aksnes LH, Bauer HC, Jebsen NL, Follerås G, Allert C, Haugen GS, et al. Limb-sparing surgery preserves more function than amputation: a Scandinavian sarcoma group study of 118 patients. J Bone Joint Surg Br. 2008;90(6):786-94.

28. Winkler K, Beron G, Kotz R, Salzer-Kuntschik M, Beck J, Beck W, et al. Neoadjuvant chemotherapy for osteogenic sarcoma: results of a Cooperative German/Austrian study. J Clin Oncol. 1984;2(6):617-24.

29. Polites SF, Heaton TE, LaQuaglia MP, Kim ES, Barry WE, Goodhue CJ, et al. Pneumonectomy for Pediatric Tumors-a Pediatric Surgical Oncology Research Collaborative Study. Ann Surg. 2021;274(6):e605-e9.

30. Bacci G, Ferrari S, Lari S, Mercuri M, Donati D, Longhi A, et al. Osteosarcoma of the limb. Amputation or limb salvage in patients treated by neoadjuvant chemotherapy. J Bone Joint Surg Br. 2002;84(1):88-92.

31. Zhao X, Wu Q, Gong X, Liu J, Ma Y. Osteosarcoma: a review of current and future therapeutic approaches. Biomed Eng Online. 2021;20(1):24.

1. Isakoff MS, Bielack SS, Meltzer P, Gorlick R. Osteosarcoma: Current Treatment and a Collaborative Pathway to Success. J Clin Oncol. 2015;33(27):3029-35.

2. Ferrari S, Smeland S, Mercuri M, Bertoni F, Longhi A, Ruggieri P, et al. Neoadjuvant chemotherapy with high-dose Ifosfamide, high-dose methotrexate, cisplatin, and doxorubicin for patients with localized osteosarcoma of the extremity: a joint study by the Italian and Scandinavian Sarcoma Groups. J Clin Oncol. 2005;23(34):8845-52.

3. Siegel RL, Miller KD, Jemal A. Cancer statistics, 2019. CA Cancer J Clin. 2019;69(1):7-34.

4. Ritter J, Bielack SS. Osteosarcoma. Ann Oncol. 2010;21 Suppl 7:vii320-5.

5. Gill J, Gorlick R. Advancing therapy for osteosarcoma. Nat Rev Clin Oncol. 2021;18(10):609-24.

6. Bielack SS, Kempf-Bielack B, Delling G, Exner GU, Flege S, Helmke K, et al. Prognostic factors in high-grade osteosarcoma of the extremities or trunk: an analysis of 1,702 patients treated on neoadjuvant cooperative osteosarcoma study group protocols. J Clin Oncol. 2002;20(3):776-90.

7. Han Z, Peng X, Yang Y, Yi J, Zhao D, Bao Q, et al. Integrated microfluidic-SERS for exosome biomarker profiling and osteosarcoma diagnosis. Biosens Bioelectron. 2022;217:114709.

8. Wu Y, Xu L, Yang P, Lin N, Huang X, Pan W, et al. Survival Prediction in High-grade Osteosarcoma Using Radiomics of Diagnostic Computed Tomography. EBioMedicine. 2018;34:27-34.

9. Chen W, Lin Y, Huang J, Yan Z, Cao H. A novel risk score model based on glycolysis-related genes and a prognostic model for predicting overall survival of osteosarcoma patients. J Orthop Res. 2022;40(10):2372-81.

10. Zhang M, Liu Y, Kong D. Identifying biomolecules and constructing a prognostic risk prediction model for recurrence in osteosarcoma. J Bone Oncol. 2021;26:100331.

11. Li L, Wang Y, He X, Li Z, Lu M, Gong T, et al. Hematological Prognostic Scoring System Can Predict Overall Survival and Can Indicate Response to Immunotherapy in Patients With Osteosarcoma. Front Immunol. 2022;13:879560.

12. Pelish HE, Liau BB, Nitulescu, II, Tangpeerachaikul A, Poss ZC, Da Silva DH, et al. Mediator kinase inhibition further activates super-enhancer-associated genes in AML. Nature. 2015;526(7572):273-6.

13. Whyte WA, Orlando DA, Hnisz D, Abraham BJ, Lin CY, Kagey MH, et al. Master transcription factors and mediator establish super-enhancers at key cell identity genes. Cell. 2013;153(2):307-19.

14. Tögel L, Nightingale R, Chueh AC, Jayachandran A, Tran H, Phesse T, et al. Dual Targeting of Bromodomain and Extraterminal Domain Proteins, and WNT or MAPK Signaling, Inhibits c-MYC Expression and Proliferation of Colorectal Cancer Cells. Mol Cancer Ther. 2016;15(6):1217-26.

15. Mansour MR, Abraham BJ, Anders L, Berezovskaya A, Gutierrez A, Durbin AD, et al. Oncogene regulation. An oncogenic super-enhancer formed through somatic mutation of a noncoding intergenic element. Science. 2014;346(6215):1373-7.

16. Lovén J, Hoke HA, Lin CY, Lau A, Orlando DA, Vakoc CR, et al. Selective inhibition of tumor oncogenes by disruption of super-enhancers. Cell. 2013;153(2):320-34.

17. Wang Y, Zhang T, Kwiatkowski N, Abraham BJ, Lee TI, Xie S, et al. CDK7-dependent transcriptional addiction in triple-negative breast cancer. Cell. 2015;163(1):174-86.

18. Zhang J, Liu W, Zou C, Zhao Z, Lai Y, Shi Z, et al. Targeting Super-Enhancer-Associated Oncogenes in Osteosarcoma with THZ2, a Covalent CDK7 Inhibitor. Clin Cancer Res. 2020;26(11):2681-92.

19. Xing R, Zhou Y, Yu J, Yu Y, Nie Y, Luo W, et al. Whole-genome sequencing reveals novel tandem-duplication hotspots and a prognostic mutational signature in gastric cancer. Nat Commun. 2019;10(1):2037.

20. Zou CY, Wang J, Shen JN, Huang G, Jin S, Yin JQ, et al. Establishment and characteristics of two syngeneic human osteosarcoma cell lines from primary tumor and skip metastases. Acta Pharmacol Sin. 2008;29(3):325-32.

21. Han J, Yong B, Luo C, Tan P, Peng T, Shen J. High serum alkaline phosphatase cooperating with MMP-9 predicts metastasis and poor prognosis in patients with primary osteosarcoma in Southern China. World J Surg Oncol. 2012;10:37.

22. Szulc P, Seeman E, Delmas PD. Biochemical measurements of bone turnover in children and adolescents. Osteoporos Int. 2000;11(4):281-94.

23. Camp RL, Dolled-Filhart M, Rimm DL. X-tile: a new bio-informatics tool for biomarker assessment and outcome-based cut-point optimization. Clin Cancer Res. 2004;10(21):7252-9.

24. Zhang T, Kwiatkowski N, Olson CM, Dixon-Clarke SE, Abraham BJ, Greifenberg AK, et al. Covalent targeting of remote cysteine residues to develop CDK12 and CDK13 inhibitors. Nat Chem Biol. 2016;12(10):876-84.

25. Jiang YY, Lin DC, Mayakonda A, Hazawa M, Ding LW, Chien WW, et al. Targeting super-enhancer-associated oncogenes in oesophageal squamous cell carcinoma. Gut. 2017;66(8):1358-68.

26. Meyers PA, Schwartz CL, Krailo M, Kleinerman ES, Betcher D, Bernstein ML, et al. Osteosarcoma: a randomized, prospective trial of the addition of ifosfamide and/or muramyl tripeptide to cisplatin, doxorubicin, and high-dose methotrexate. J Clin Oncol. 2005;23(9):2004-11.

27. Aksnes LH, Bauer HC, Jebsen NL, Follerås G, Allert C, Haugen GS, et al. Limb-sparing surgery preserves more function than amputation: a Scandinavian sarcoma group study of 118 patients. J Bone Joint Surg Br. 2008;90(6):786-94.

28. Winkler K, Beron G, Kotz R, Salzer-Kuntschik M, Beck J, Beck W, et al. Neoadjuvant chemotherapy for osteogenic sarcoma: results of a Cooperative German/Austrian study. J Clin Oncol. 1984;2(6):617-24.

29. Polites SF, Heaton TE, LaQuaglia MP, Kim ES, Barry WE, Goodhue CJ, et al. Pneumonectomy for Pediatric Tumors-a Pediatric Surgical Oncology Research Collaborative Study. Ann Surg. 2021;274(6):e605-e9.

30. Bacci G, Ferrari S, Lari S, Mercuri M, Donati D, Longhi A, et al. Osteosarcoma of the limb. Amputation or limb salvage in patients treated by neoadjuvant chemotherapy. J Bone Joint Surg Br. 2002;84(1):88-92.

31. Zhao X, Wu Q, Gong X, Liu J, Ma Y. Osteosarcoma: a review of current and future therapeutic approaches. Biomed Eng Online. 2021;20(1):24.

32. Huang H, Hu J, Maryam A, Huang Q, Zhang Y, Ramakrishnan S, et al. Defining super-enhancer landscape in triple-negative breast cancer by multiomic profiling. Nat Commun. 2021;12(1):2242.

1. Isakoff MS, Bielack SS, Meltzer P, Gorlick R. Osteosarcoma: Current Treatment and a Collaborative Pathway to Success. J Clin Oncol. 2015;33(27):3029-35.

2. Ferrari S, Smeland S, Mercuri M, Bertoni F, Longhi A, Ruggieri P, et al. Neoadjuvant chemotherapy with high-dose Ifosfamide, high-dose methotrexate, cisplatin, and doxorubicin for patients with localized osteosarcoma of the extremity: a joint study by the Italian and Scandinavian Sarcoma Groups. J Clin Oncol. 2005;23(34):8845-52.

3. Siegel RL, Miller KD, Jemal A. Cancer statistics, 2019. CA Cancer J Clin. 2019;69(1):7-34.

4. Ritter J, Bielack SS. Osteosarcoma. Ann Oncol. 2010;21 Suppl 7:vii320-5.

5. Gill J, Gorlick R. Advancing therapy for osteosarcoma. Nat Rev Clin Oncol. 2021;18(10):609-24.

6. Bielack SS, Kempf-Bielack B, Delling G, Exner GU, Flege S, Helmke K, et al. Prognostic factors in high-grade osteosarcoma of the extremities or trunk: an analysis of 1,702 patients treated on neoadjuvant cooperative osteosarcoma study group protocols. J Clin Oncol. 2002;20(3):776-90.

7. Han Z, Peng X, Yang Y, Yi J, Zhao D, Bao Q, et al. Integrated microfluidic-SERS for exosome biomarker profiling and osteosarcoma diagnosis. Biosens Bioelectron. 2022;217:114709.

8. Wu Y, Xu L, Yang P, Lin N, Huang X, Pan W, et al. Survival Prediction in High-grade Osteosarcoma Using Radiomics of Diagnostic Computed Tomography. EBioMedicine. 2018;34:27-34.

9. Chen W, Lin Y, Huang J, Yan Z, Cao H. A novel risk score model based on glycolysis-related genes and a prognostic model for predicting overall survival of osteosarcoma patients. J Orthop Res. 2022;40(10):2372-81.

10. Zhang M, Liu Y, Kong D. Identifying biomolecules and constructing a prognostic risk prediction model for recurrence in osteosarcoma. J Bone Oncol. 2021;26:100331.

11. Li L, Wang Y, He X, Li Z, Lu M, Gong T, et al. Hematological Prognostic Scoring System Can Predict Overall Survival and Can Indicate Response to Immunotherapy in Patients With Osteosarcoma. Front Immunol. 2022;13:879560.

12. Pelish HE, Liau BB, Nitulescu, II, Tangpeerachaikul A, Poss ZC, Da Silva DH, et al. Mediator kinase inhibition further activates super-enhancer-associated genes in AML. Nature. 2015;526(7572):273-6.

13. Whyte WA, Orlando DA, Hnisz D, Abraham BJ, Lin CY, Kagey MH, et al. Master transcription factors and mediator establish super-enhancers at key cell identity genes. Cell. 2013;153(2):307-19.

14. Tögel L, Nightingale R, Chueh AC, Jayachandran A, Tran H, Phesse T, et al. Dual Targeting of Bromodomain and Extraterminal Domain Proteins, and WNT or MAPK Signaling, Inhibits c-MYC Expression and Proliferation of Colorectal Cancer Cells. Mol Cancer Ther. 2016;15(6):1217-26.

15. Mansour MR, Abraham BJ, Anders L, Berezovskaya A, Gutierrez A, Durbin AD, et al. Oncogene regulation. An oncogenic super-enhancer formed through somatic mutation of a noncoding intergenic element. Science. 2014;346(6215):1373-7.

16. Lovén J, Hoke HA, Lin CY, Lau A, Orlando DA, Vakoc CR, et al. Selective inhibition of tumor oncogenes by disruption of super-enhancers. Cell. 2013;153(2):320-34.

17. Wang Y, Zhang T, Kwiatkowski N, Abraham BJ, Lee TI, Xie S, et al. CDK7-dependent transcriptional addiction in triple-negative breast cancer. Cell. 2015;163(1):174-86.

18. Zhang J, Liu W, Zou C, Zhao Z, Lai Y, Shi Z, et al. Targeting Super-Enhancer-Associated Oncogenes in Osteosarcoma with THZ2, a Covalent CDK7 Inhibitor. Clin Cancer Res. 2020;26(11):2681-92.

19. Xing R, Zhou Y, Yu J, Yu Y, Nie Y, Luo W, et al. Whole-genome sequencing reveals novel tandem-duplication hotspots and a prognostic mutational signature in gastric cancer. Nat Commun. 2019;10(1):2037.

20. Zou CY, Wang J, Shen JN, Huang G, Jin S, Yin JQ, et al. Establishment and characteristics of two syngeneic human osteosarcoma cell lines from primary tumor and skip metastases. Acta Pharmacol Sin. 2008;29(3):325-32.

21. Han J, Yong B, Luo C, Tan P, Peng T, Shen J. High serum alkaline phosphatase cooperating with MMP-9 predicts metastasis and poor prognosis in patients with primary osteosarcoma in Southern China. World J Surg Oncol. 2012;10:37.

22. Szulc P, Seeman E, Delmas PD. Biochemical measurements of bone turnover in children and adolescents. Osteoporos Int. 2000;11(4):281-94.

23. Camp RL, Dolled-Filhart M, Rimm DL. X-tile: a new bio-informatics tool for biomarker assessment and outcome-based cut-point optimization. Clin Cancer Res. 2004;10(21):7252-9.

24. Zhang T, Kwiatkowski N, Olson CM, Dixon-Clarke SE, Abraham BJ, Greifenberg AK, et al. Covalent targeting of remote cysteine residues to develop CDK12 and CDK13 inhibitors. Nat Chem Biol. 2016;12(10):876-84.

25. Jiang YY, Lin DC, Mayakonda A, Hazawa M, Ding LW, Chien WW, et al. Targeting super-enhancer-associated oncogenes in oesophageal squamous cell carcinoma. Gut. 2017;66(8):1358-68.

26. Meyers PA, Schwartz CL, Krailo M, Kleinerman ES, Betcher D, Bernstein ML, et al. Osteosarcoma: a randomized, prospective trial of the addition of ifosfamide and/or muramyl tripeptide to cisplatin, doxorubicin, and high-dose methotrexate. J Clin Oncol. 2005;23(9):2004-11.

27. Aksnes LH, Bauer HC, Jebsen NL, Follerås G, Allert C, Haugen GS, et al. Limb-sparing surgery preserves more function than amputation: a Scandinavian sarcoma group study of 118 patients. J Bone Joint Surg Br. 2008;90(6):786-94.

28. Winkler K, Beron G, Kotz R, Salzer-Kuntschik M, Beck J, Beck W, et al. Neoadjuvant chemotherapy for osteogenic sarcoma: results of a Cooperative German/Austrian study. J Clin Oncol. 1984;2(6):617-24.

29. Polites SF, Heaton TE, LaQuaglia MP, Kim ES, Barry WE, Goodhue CJ, et al. Pneumonectomy for Pediatric Tumors-a Pediatric Surgical Oncology Research Collaborative Study. Ann Surg. 2021;274(6):e605-e9.

30. Bacci G, Ferrari S, Lari S, Mercuri M, Donati D, Longhi A, et al. Osteosarcoma of the limb. Amputation or limb salvage in patients treated by neoadjuvant chemotherapy. J Bone Joint Surg Br. 2002;84(1):88-92.

31. Zhao X, Wu Q, Gong X, Liu J, Ma Y. Osteosarcoma: a review of current and future therapeutic approaches. Biomed Eng Online. 2021;20(1):24.

32. Huang H, Hu J, Maryam A, Huang Q, Zhang Y, Ramakrishnan S, et al. Defining super-enhancer landscape in triple-negative breast cancer by multiomic profiling. Nat Commun. 2021;12(1):2242.

33. Wong M, Sun Y, Xi Z, Milazzo G, Poulos RC, Bartenhagen C, et al. JMJD6 is a tumorigenic factor and therapeutic target in neuroblastoma. Nat Commun. 2019;10(1):3319.

2. Ferrari S, Smeland S, Mercuri M, Bertoni F, Longhi A, Ruggieri P, et al. Neoadjuvant chemotherapy with high-dose Ifosfamide, high-dose methotrexate, cisplatin, and doxorubicin for patients with localized osteosarcoma of the extremity: a joint study by the Italian and Scandinavian Sarcoma Groups. J Clin Oncol. 2005;23(34):8845-52.

3. Siegel RL, Miller KD, Jemal A. Cancer statistics, 2019. CA Cancer J Clin. 2019;69(1):7-34.

4. Ritter J, Bielack SS. Osteosarcoma. Ann Oncol. 2010;21 Suppl 7:vii320-5.

5. Gill J, Gorlick R. Advancing therapy for osteosarcoma. Nat Rev Clin Oncol. 2021;18(10):609-24.

6. Bielack SS, Kempf-Bielack B, Delling G, Exner GU, Flege S, Helmke K, et al. Prognostic factors in high-grade osteosarcoma of the extremities or trunk: an analysis of 1,702 patients treated on neoadjuvant cooperative osteosarcoma study group protocols. J Clin Oncol. 2002;20(3):776-90.

7. Han Z, Peng X, Yang Y, Yi J, Zhao D, Bao Q, et al. Integrated microfluidic-SERS for exosome biomarker profiling and osteosarcoma diagnosis. Biosens Bioelectron. 2022;217:114709.

8. Wu Y, Xu L, Yang P, Lin N, Huang X, Pan W, et al. Survival Prediction in High-grade Osteosarcoma Using Radiomics of Diagnostic Computed Tomography. EBioMedicine. 2018;34:27-34.

9. Chen W, Lin Y, Huang J, Yan Z, Cao H. A novel risk score model based on glycolysis-related genes and a prognostic model for predicting overall survival of osteosarcoma patients. J Orthop Res. 2022;40(10):2372-81.

10. Zhang M, Liu Y, Kong D. Identifying biomolecules and constructing a prognostic risk prediction model for recurrence in osteosarcoma. J Bone Oncol. 2021;26:100331.

11. Li L, Wang Y, He X, Li Z, Lu M, Gong T, et al. Hematological Prognostic Scoring System Can Predict Overall Survival and Can Indicate Response to Immunotherapy in Patients With Osteosarcoma. Front Immunol. 2022;13:879560.

12. Pelish HE, Liau BB, Nitulescu, II, Tangpeerachaikul A, Poss ZC, Da Silva DH, et al. Mediator kinase inhibition further activates super-enhancer-associated genes in AML. Nature. 2015;526(7572):273-6.

13. Whyte WA, Orlando DA, Hnisz D, Abraham BJ, Lin CY, Kagey MH, et al. Master transcription factors and mediator establish super-enhancers at key cell identity genes. Cell. 2013;153(2):307-19.

14. Tögel L, Nightingale R, Chueh AC, Jayachandran A, Tran H, Phesse T, et al. Dual Targeting of Bromodomain and Extraterminal Domain Proteins, and WNT or MAPK Signaling, Inhibits c-MYC Expression and Proliferation of Colorectal Cancer Cells. Mol Cancer Ther. 2016;15(6):1217-26.

15. Mansour MR, Abraham BJ, Anders L, Berezovskaya A, Gutierrez A, Durbin AD, et al. Oncogene regulation. An oncogenic super-enhancer formed through somatic mutation of a noncoding intergenic element. Science. 2014;346(6215):1373-7.

16. Lovén J, Hoke HA, Lin CY, Lau A, Orlando DA, Vakoc CR, et al. Selective inhibition of tumor oncogenes by disruption of super-enhancers. Cell. 2013;153(2):320-34.

17. Wang Y, Zhang T, Kwiatkowski N, Abraham BJ, Lee TI, Xie S, et al. CDK7-dependent transcriptional addiction in triple-negative breast cancer. Cell. 2015;163(1):174-86.

18. Zhang J, Liu W, Zou C, Zhao Z, Lai Y, Shi Z, et al. Targeting Super-Enhancer-Associated Oncogenes in Osteosarcoma with THZ2, a Covalent CDK7 Inhibitor. Clin Cancer Res. 2020;26(11):2681-92.

19. Xing R, Zhou Y, Yu J, Yu Y, Nie Y, Luo W, et al. Whole-genome sequencing reveals novel tandem-duplication hotspots and a prognostic mutational signature in gastric cancer. Nat Commun. 2019;10(1):2037.

20. Zou CY, Wang J, Shen JN, Huang G, Jin S, Yin JQ, et al. Establishment and characteristics of two syngeneic human osteosarcoma cell lines from primary tumor and skip metastases. Acta Pharmacol Sin. 2008;29(3):325-32.

21. Han J, Yong B, Luo C, Tan P, Peng T, Shen J. High serum alkaline phosphatase cooperating with MMP-9 predicts metastasis and poor prognosis in patients with primary osteosarcoma in Southern China. World J Surg Oncol. 2012;10:37.

22. Szulc P, Seeman E, Delmas PD. Biochemical measurements of bone turnover in children and adolescents. Osteoporos Int. 2000;11(4):281-94.

23. Camp RL, Dolled-Filhart M, Rimm DL. X-tile: a new bio-informatics tool for biomarker assessment and outcome-based cut-point optimization. Clin Cancer Res. 2004;10(21):7252-9.

24. Zhang T, Kwiatkowski N, Olson CM, Dixon-Clarke SE, Abraham BJ, Greifenberg AK, et al. Covalent targeting of remote cysteine residues to develop CDK12 and CDK13 inhibitors. Nat Chem Biol. 2016;12(10):876-84.

25. Jiang YY, Lin DC, Mayakonda A, Hazawa M, Ding LW, Chien WW, et al. Targeting super-enhancer-associated oncogenes in oesophageal squamous cell carcinoma. Gut. 2017;66(8):1358-68.

26. Meyers PA, Schwartz CL, Krailo M, Kleinerman ES, Betcher D, Bernstein ML, et al. Osteosarcoma: a randomized, prospective trial of the addition of ifosfamide and/or muramyl tripeptide to cisplatin, doxorubicin, and high-dose methotrexate. J Clin Oncol. 2005;23(9):2004-11.

27. Aksnes LH, Bauer HC, Jebsen NL, Follerås G, Allert C, Haugen GS, et al. Limb-sparing surgery preserves more function than amputation: a Scandinavian sarcoma group study of 118 patients. J Bone Joint Surg Br. 2008;90(6):786-94.

28. Winkler K, Beron G, Kotz R, Salzer-Kuntschik M, Beck J, Beck W, et al. Neoadjuvant chemotherapy for osteogenic sarcoma: results of a Cooperative German/Austrian study. J Clin Oncol. 1984;2(6):617-24.

29. Polites SF, Heaton TE, LaQuaglia MP, Kim ES, Barry WE, Goodhue CJ, et al. Pneumonectomy for Pediatric Tumors-a Pediatric Surgical Oncology Research Collaborative Study. Ann Surg. 2021;274(6):e605-e9.

30. Bacci G, Ferrari S, Lari S, Mercuri M, Donati D, Longhi A, et al. Osteosarcoma of the limb. Amputation or limb salvage in patients treated by neoadjuvant chemotherapy. J Bone Joint Surg Br. 2002;84(1):88-92.

31. Zhao X, Wu Q, Gong X, Liu J, Ma Y. Osteosarcoma: a review of current and future therapeutic approaches. Biomed Eng Online. 2021;20(1):24.

32. Huang H, Hu J, Maryam A, Huang Q, Zhang Y, Ramakrishnan S, et al. Defining super-enhancer landscape in triple-negative breast cancer by multiomic profiling. Nat Commun. 2021;12(1):2242.

33. Wong M, Sun Y, Xi Z, Milazzo G, Poulos RC, Bartenhagen C, et al. JMJD6 is a tumorigenic factor and therapeutic target in neuroblastoma. Nat Commun. 2019;10(1):3319.

34. Zhang L, Xiong D, Liu Q, Luo Y, Tian Y, Xiao X, et al. Genome-Wide Histone H3K27 Acetylation Profiling Identified Genes Correlated With Prognosis in Papillary Thyroid Carcinoma. Front Cell Dev Biol. 2021;9:682561.

1. Isakoff MS, Bielack SS, Meltzer P, Gorlick R. Osteosarcoma: Current Treatment and a Collaborative Pathway to Success. J Clin Oncol. 2015;33(27):3029-35.

2. Ferrari S, Smeland S, Mercuri M, Bertoni F, Longhi A, Ruggieri P, et al. Neoadjuvant chemotherapy with high-dose Ifosfamide, high-dose methotrexate, cisplatin, and doxorubicin for patients with localized osteosarcoma of the extremity: a joint study by the Italian and Scandinavian Sarcoma Groups. J Clin Oncol. 2005;23(34):8845-52.

3. Siegel RL, Miller KD, Jemal A. Cancer statistics, 2019. CA Cancer J Clin. 2019;69(1):7-34.

4. Ritter J, Bielack SS. Osteosarcoma. Ann Oncol. 2010;21 Suppl 7:vii320-5.

5. Gill J, Gorlick R. Advancing therapy for osteosarcoma. Nat Rev Clin Oncol. 2021;18(10):609-24.

6. Bielack SS, Kempf-Bielack B, Delling G, Exner GU, Flege S, Helmke K, et al. Prognostic factors in high-grade osteosarcoma of the extremities or trunk: an analysis of 1,702 patients treated on neoadjuvant cooperative osteosarcoma study group protocols. J Clin Oncol. 2002;20(3):776-90.

7. Han Z, Peng X, Yang Y, Yi J, Zhao D, Bao Q, et al. Integrated microfluidic-SERS for exosome biomarker profiling and osteosarcoma diagnosis. Biosens Bioelectron. 2022;217:114709.

8. Wu Y, Xu L, Yang P, Lin N, Huang X, Pan W, et al. Survival Prediction in High-grade Osteosarcoma Using Radiomics of Diagnostic Computed Tomography. EBioMedicine. 2018;34:27-34.

9. Chen W, Lin Y, Huang J, Yan Z, Cao H. A novel risk score model based on glycolysis-related genes and a prognostic model for predicting overall survival of osteosarcoma patients. J Orthop Res. 2022;40(10):2372-81.

10. Zhang M, Liu Y, Kong D. Identifying biomolecules and constructing a prognostic risk prediction model for recurrence in osteosarcoma. J Bone Oncol. 2021;26:100331.

11. Li L, Wang Y, He X, Li Z, Lu M, Gong T, et al. Hematological Prognostic Scoring System Can Predict Overall Survival and Can Indicate Response to Immunotherapy in Patients With Osteosarcoma. Front Immunol. 2022;13:879560.

12. Pelish HE, Liau BB, Nitulescu, II, Tangpeerachaikul A, Poss ZC, Da Silva DH, et al. Mediator kinase inhibition further activates super-enhancer-associated genes in AML. Nature. 2015;526(7572):273-6.

13. Whyte WA, Orlando DA, Hnisz D, Abraham BJ, Lin CY, Kagey MH, et al. Master transcription factors and mediator establish super-enhancers at key cell identity genes. Cell. 2013;153(2):307-19.

14. Tögel L, Nightingale R, Chueh AC, Jayachandran A, Tran H, Phesse T, et al. Dual Targeting of Bromodomain and Extraterminal Domain Proteins, and WNT or MAPK Signaling, Inhibits c-MYC Expression and Proliferation of Colorectal Cancer Cells. Mol Cancer Ther. 2016;15(6):1217-26.

15. Mansour MR, Abraham BJ, Anders L, Berezovskaya A, Gutierrez A, Durbin AD, et al. Oncogene regulation. An oncogenic super-enhancer formed through somatic mutation of a noncoding intergenic element. Science. 2014;346(6215):1373-7.

16. Lovén J, Hoke HA, Lin CY, Lau A, Orlando DA, Vakoc CR, et al. Selective inhibition of tumor oncogenes by disruption of super-enhancers. Cell. 2013;153(2):320-34.

17. Wang Y, Zhang T, Kwiatkowski N, Abraham BJ, Lee TI, Xie S, et al. CDK7-dependent transcriptional addiction in triple-negative breast cancer. Cell. 2015;163(1):174-86.

18. Zhang J, Liu W, Zou C, Zhao Z, Lai Y, Shi Z, et al. Targeting Super-Enhancer-Associated Oncogenes in Osteosarcoma with THZ2, a Covalent CDK7 Inhibitor. Clin Cancer Res. 2020;26(11):2681-92.

19. Xing R, Zhou Y, Yu J, Yu Y, Nie Y, Luo W, et al. Whole-genome sequencing reveals novel tandem-duplication hotspots and a prognostic mutational signature in gastric cancer. Nat Commun. 2019;10(1):2037.

20. Zou CY, Wang J, Shen JN, Huang G, Jin S, Yin JQ, et al. Establishment and characteristics of two syngeneic human osteosarcoma cell lines from primary tumor and skip metastases. Acta Pharmacol Sin. 2008;29(3):325-32.

21. Han J, Yong B, Luo C, Tan P, Peng T, Shen J. High serum alkaline phosphatase cooperating with MMP-9 predicts metastasis and poor prognosis in patients with primary osteosarcoma in Southern China. World J Surg Oncol. 2012;10:37.

22. Szulc P, Seeman E, Delmas PD. Biochemical measurements of bone turnover in children and adolescents. Osteoporos Int. 2000;11(4):281-94.

23. Camp RL, Dolled-Filhart M, Rimm DL. X-tile: a new bio-informatics tool for biomarker assessment and outcome-based cut-point optimization. Clin Cancer Res. 2004;10(21):7252-9.

24. Zhang T, Kwiatkowski N, Olson CM, Dixon-Clarke SE, Abraham BJ, Greifenberg AK, et al. Covalent targeting of remote cysteine residues to develop CDK12 and CDK13 inhibitors. Nat Chem Biol. 2016;12(10):876-84.

25. Jiang YY, Lin DC, Mayakonda A, Hazawa M, Ding LW, Chien WW, et al. Targeting super-enhancer-associated oncogenes in oesophageal squamous cell carcinoma. Gut. 2017;66(8):1358-68.

26. Meyers PA, Schwartz CL, Krailo M, Kleinerman ES, Betcher D, Bernstein ML, et al. Osteosarcoma: a randomized, prospective trial of the addition of ifosfamide and/or muramyl tripeptide to cisplatin, doxorubicin, and high-dose methotrexate. J Clin Oncol. 2005;23(9):2004-11.

27. Aksnes LH, Bauer HC, Jebsen NL, Follerås G, Allert C, Haugen GS, et al. Limb-sparing surgery preserves more function than amputation: a Scandinavian sarcoma group study of 118 patients. J Bone Joint Surg Br. 2008;90(6):786-94.

28. Winkler K, Beron G, Kotz R, Salzer-Kuntschik M, Beck J, Beck W, et al. Neoadjuvant chemotherapy for osteogenic sarcoma: results of a Cooperative German/Austrian study. J Clin Oncol. 1984;2(6):617-24.

29. Polites SF, Heaton TE, LaQuaglia MP, Kim ES, Barry WE, Goodhue CJ, et al. Pneumonectomy for Pediatric Tumors-a Pediatric Surgical Oncology Research Collaborative Study. Ann Surg. 2021;274(6):e605-e9.

30. Bacci G, Ferrari S, Lari S, Mercuri M, Donati D, Longhi A, et al. Osteosarcoma of the limb. Amputation or limb salvage in patients treated by neoadjuvant chemotherapy. J Bone Joint Surg Br. 2002;84(1):88-92.

31. Zhao X, Wu Q, Gong X, Liu J, Ma Y. Osteosarcoma: a review of current and future therapeutic approaches. Biomed Eng Online. 2021;20(1):24.

32. Huang H, Hu J, Maryam A, Huang Q, Zhang Y, Ramakrishnan S, et al. Defining super-enhancer landscape in triple-negative breast cancer by multiomic profiling. Nat Commun. 2021;12(1):2242.

33. Wong M, Sun Y, Xi Z, Milazzo G, Poulos RC, Bartenhagen C, et al. JMJD6 is a tumorigenic factor and therapeutic target in neuroblastoma. Nat Commun. 2019;10(1):3319.

34. Zhang L, Xiong D, Liu Q, Luo Y, Tian Y, Xiao X, et al. Genome-Wide Histone H3K27 Acetylation Profiling Identified Genes Correlated With Prognosis in Papillary Thyroid Carcinoma. Front Cell Dev Biol. 2021;9:682561.

1. Isakoff MS, Bielack SS, Meltzer P, Gorlick R. Osteosarcoma: Current Treatment and a Collaborative Pathway to Success. J Clin Oncol. 2015;33(27):3029-35.

2. Ferrari S, Smeland S, Mercuri M, Bertoni F, Longhi A, Ruggieri P, et al. Neoadjuvant chemotherapy with high-dose Ifosfamide, high-dose methotrexate, cisplatin, and doxorubicin for patients with localized osteosarcoma of the extremity: a joint study by the Italian and Scandinavian Sarcoma Groups. J Clin Oncol. 2005;23(34):8845-52.

3. Siegel RL, Miller KD, Jemal A. Cancer statistics, 2019. CA Cancer J Clin. 2019;69(1):7-34.

4. Ritter J, Bielack SS. Osteosarcoma. Ann Oncol. 2010;21 Suppl 7:vii320-5.

5. Gill J, Gorlick R. Advancing therapy for osteosarcoma. Nat Rev Clin Oncol. 2021;18(10):609-24.

6. Bielack SS, Kempf-Bielack B, Delling G, Exner GU, Flege S, Helmke K, et al. Prognostic factors in high-grade osteosarcoma of the extremities or trunk: an analysis of 1,702 patients treated on neoadjuvant cooperative osteosarcoma study group protocols. J Clin Oncol. 2002;20(3):776-90.

7. Han Z, Peng X, Yang Y, Yi J, Zhao D, Bao Q, et al. Integrated microfluidic-SERS for exosome biomarker profiling and osteosarcoma diagnosis. Biosens Bioelectron. 2022;217:114709.

8. Wu Y, Xu L, Yang P, Lin N, Huang X, Pan W, et al. Survival Prediction in High-grade Osteosarcoma Using Radiomics of Diagnostic Computed Tomography. EBioMedicine. 2018;34:27-34.

9. Chen W, Lin Y, Huang J, Yan Z, Cao H. A novel risk score model based on glycolysis-related genes and a prognostic model for predicting overall survival of osteosarcoma patients. J Orthop Res. 2022;40(10):2372-81.

10. Zhang M, Liu Y, Kong D. Identifying biomolecules and constructing a prognostic risk prediction model for recurrence in osteosarcoma. J Bone Oncol. 2021;26:100331.

11. Li L, Wang Y, He X, Li Z, Lu M, Gong T, et al. Hematological Prognostic Scoring System Can Predict Overall Survival and Can Indicate Response to Immunotherapy in Patients With Osteosarcoma. Front Immunol. 2022;13:879560.

12. Pelish HE, Liau BB, Nitulescu, II, Tangpeerachaikul A, Poss ZC, Da Silva DH, et al. Mediator kinase inhibition further activates super-enhancer-associated genes in AML. Nature. 2015;526(7572):273-6.

13. Whyte WA, Orlando DA, Hnisz D, Abraham BJ, Lin CY, Kagey MH, et al. Master transcription factors and mediator establish super-enhancers at key cell identity genes. Cell. 2013;153(2):307-19.

14. Tögel L, Nightingale R, Chueh AC, Jayachandran A, Tran H, Phesse T, et al. Dual Targeting of Bromodomain and Extraterminal Domain Proteins, and WNT or MAPK Signaling, Inhibits c-MYC Expression and Proliferation of Colorectal Cancer Cells. Mol Cancer Ther. 2016;15(6):1217-26.

15. Mansour MR, Abraham BJ, Anders L, Berezovskaya A, Gutierrez A, Durbin AD, et al. Oncogene regulation. An oncogenic super-enhancer formed through somatic mutation of a noncoding intergenic element. Science. 2014;346(6215):1373-7.

16. Lovén J, Hoke HA, Lin CY, Lau A, Orlando DA, Vakoc CR, et al. Selective inhibition of tumor oncogenes by disruption of super-enhancers. Cell. 2013;153(2):320-34.

17. Wang Y, Zhang T, Kwiatkowski N, Abraham BJ, Lee TI, Xie S, et al. CDK7-dependent transcriptional addiction in triple-negative breast cancer. Cell. 2015;163(1):174-86.

18. Zhang J, Liu W, Zou C, Zhao Z, Lai Y, Shi Z, et al. Targeting Super-Enhancer-Associated Oncogenes in Osteosarcoma with THZ2, a Covalent CDK7 Inhibitor. Clin Cancer Res. 2020;26(11):2681-92.

19. Xing R, Zhou Y, Yu J, Yu Y, Nie Y, Luo W, et al. Whole-genome sequencing reveals novel tandem-duplication hotspots and a prognostic mutational signature in gastric cancer. Nat Commun. 2019;10(1):2037.

20. Zou CY, Wang J, Shen JN, Huang G, Jin S, Yin JQ, et al. Establishment and characteristics of two syngeneic human osteosarcoma cell lines from primary tumor and skip metastases. Acta Pharmacol Sin. 2008;29(3):325-32.

21. Han J, Yong B, Luo C, Tan P, Peng T, Shen J. High serum alkaline phosphatase cooperating with MMP-9 predicts metastasis and poor prognosis in patients with primary osteosarcoma in Southern China. World J Surg Oncol. 2012;10:37.

22. Szulc P, Seeman E, Delmas PD. Biochemical measurements of bone turnover in children and adolescents. Osteoporos Int. 2000;11(4):281-94.

23. Camp RL, Dolled-Filhart M, Rimm DL. X-tile: a new bio-informatics tool for biomarker assessment and outcome-based cut-point optimization. Clin Cancer Res. 2004;10(21):7252-9.

24. Zhang T, Kwiatkowski N, Olson CM, Dixon-Clarke SE, Abraham BJ, Greifenberg AK, et al. Covalent targeting of remote cysteine residues to develop CDK12 and CDK13 inhibitors. Nat Chem Biol. 2016;12(10):876-84.

25. Jiang YY, Lin DC, Mayakonda A, Hazawa M, Ding LW, Chien WW, et al. Targeting super-enhancer-associated oncogenes in oesophageal squamous cell carcinoma. Gut. 2017;66(8):1358-68.

26. Meyers PA, Schwartz CL, Krailo M, Kleinerman ES, Betcher D, Bernstein ML, et al. Osteosarcoma: a randomized, prospective trial of the addition of ifosfamide and/or muramyl tripeptide to cisplatin, doxorubicin, and high-dose methotrexate. J Clin Oncol. 2005;23(9):2004-11.

27. Aksnes LH, Bauer HC, Jebsen NL, Follerås G, Allert C, Haugen GS, et al. Limb-sparing surgery preserves more function than amputation: a Scandinavian sarcoma group study of 118 patients. J Bone Joint Surg Br. 2008;90(6):786-94.

28. Winkler K, Beron G, Kotz R, Salzer-Kuntschik M, Beck J, Beck W, et al. Neoadjuvant chemotherapy for osteogenic sarcoma: results of a Cooperative German/Austrian study. J Clin Oncol. 1984;2(6):617-24.

29. Polites SF, Heaton TE, LaQuaglia MP, Kim ES, Barry WE, Goodhue CJ, et al. Pneumonectomy for Pediatric Tumors-a Pediatric Surgical Oncology Research Collaborative Study. Ann Surg. 2021;274(6):e605-e9.

30. Bacci G, Ferrari S, Lari S, Mercuri M, Donati D, Longhi A, et al. Osteosarcoma of the limb. Amputation or limb salvage in patients treated by neoadjuvant chemotherapy. J Bone Joint Surg Br. 2002;84(1):88-92.

31. Zhao X, Wu Q, Gong X, Liu J, Ma Y. Osteosarcoma: a review of current and future therapeutic approaches. Biomed Eng Online. 2021;20(1):24.

32. Huang H, Hu J, Maryam A, Huang Q, Zhang Y, Ramakrishnan S, et al. Defining super-enhancer landscape in triple-negative breast cancer by multiomic profiling. Nat Commun. 2021;12(1):2242.

33. Wong M, Sun Y, Xi Z, Milazzo G, Poulos RC, Bartenhagen C, et al. JMJD6 is a tumorigenic factor and therapeutic target in neuroblastoma. Nat Commun. 2019;10(1):3319.

34. Zhang L, Xiong D, Liu Q, Luo Y, Tian Y, Xiao X, et al. Genome-Wide Histone H3K27 Acetylation Profiling Identified Genes Correlated With Prognosis in Papillary Thyroid Carcinoma. Front Cell Dev Biol. 2021;9:682561.

35. Fu Y, He G, Liu Z, Wang J, Zhang Z, Bao Q, et al. Exploration and Validation of a Novel Inflammatory Response-Associated Gene Signature to Predict Osteosarcoma Prognosis and Immune Infiltration. J Inflamm Res. 2021;14:6719-34.

1. Isakoff MS, Bielack SS, Meltzer P, Gorlick R. Osteosarcoma: Current Treatment and a Collaborative Pathway to Success. J Clin Oncol. 2015;33(27):3029-35.

2. Ferrari S, Smeland S, Mercuri M, Bertoni F, Longhi A, Ruggieri P, et al. Neoadjuvant chemotherapy with high-dose Ifosfamide, high-dose methotrexate, cisplatin, and doxorubicin for patients with localized osteosarcoma of the extremity: a joint study by the Italian and Scandinavian Sarcoma Groups. J Clin Oncol. 2005;23(34):8845-52.

3. Siegel RL, Miller KD, Jemal A. Cancer statistics, 2019. CA Cancer J Clin. 2019;69(1):7-34.

4. Ritter J, Bielack SS. Osteosarcoma. Ann Oncol. 2010;21 Suppl 7:vii320-5.

5. Gill J, Gorlick R. Advancing therapy for osteosarcoma. Nat Rev Clin Oncol. 2021;18(10):609-24.

6. Bielack SS, Kempf-Bielack B, Delling G, Exner GU, Flege S, Helmke K, et al. Prognostic factors in high-grade osteosarcoma of the extremities or trunk: an analysis of 1,702 patients treated on neoadjuvant cooperative osteosarcoma study group protocols. J Clin Oncol. 2002;20(3):776-90.

7. Han Z, Peng X, Yang Y, Yi J, Zhao D, Bao Q, et al. Integrated microfluidic-SERS for exosome biomarker profiling and osteosarcoma diagnosis. Biosens Bioelectron. 2022;217:114709.

8. Wu Y, Xu L, Yang P, Lin N, Huang X, Pan W, et al. Survival Prediction in High-grade Osteosarcoma Using Radiomics of Diagnostic Computed Tomography. EBioMedicine. 2018;34:27-34.

9. Chen W, Lin Y, Huang J, Yan Z, Cao H. A novel risk score model based on glycolysis-related genes and a prognostic model for predicting overall survival of osteosarcoma patients. J Orthop Res. 2022;40(10):2372-81.

10. Zhang M, Liu Y, Kong D. Identifying biomolecules and constructing a prognostic risk prediction model for recurrence in osteosarcoma. J Bone Oncol. 2021;26:100331.

11. Li L, Wang Y, He X, Li Z, Lu M, Gong T, et al. Hematological Prognostic Scoring System Can Predict Overall Survival and Can Indicate Response to Immunotherapy in Patients With Osteosarcoma. Front Immunol. 2022;13:879560.

12. Pelish HE, Liau BB, Nitulescu, II, Tangpeerachaikul A, Poss ZC, Da Silva DH, et al. Mediator kinase inhibition further activates super-enhancer-associated genes in AML. Nature. 2015;526(7572):273-6.

13. Whyte WA, Orlando DA, Hnisz D, Abraham BJ, Lin CY, Kagey MH, et al. Master transcription factors and mediator establish super-enhancers at key cell identity genes. Cell. 2013;153(2):307-19.

14. Tögel L, Nightingale R, Chueh AC, Jayachandran A, Tran H, Phesse T, et al. Dual Targeting of Bromodomain and Extraterminal Domain Proteins, and WNT or MAPK Signaling, Inhibits c-MYC Expression and Proliferation of Colorectal Cancer Cells. Mol Cancer Ther. 2016;15(6):1217-26.

15. Mansour MR, Abraham BJ, Anders L, Berezovskaya A, Gutierrez A, Durbin AD, et al. Oncogene regulation. An oncogenic super-enhancer formed through somatic mutation of a noncoding intergenic element. Science. 2014;346(6215):1373-7.

16. Lovén J, Hoke HA, Lin CY, Lau A, Orlando DA, Vakoc CR, et al. Selective inhibition of tumor oncogenes by disruption of super-enhancers. Cell. 2013;153(2):320-34.

17. Wang Y, Zhang T, Kwiatkowski N, Abraham BJ, Lee TI, Xie S, et al. CDK7-dependent transcriptional addiction in triple-negative breast cancer. Cell. 2015;163(1):174-86.

18. Zhang J, Liu W, Zou C, Zhao Z, Lai Y, Shi Z, et al. Targeting Super-Enhancer-Associated Oncogenes in Osteosarcoma with THZ2, a Covalent CDK7 Inhibitor. Clin Cancer Res. 2020;26(11):2681-92.

19. Xing R, Zhou Y, Yu J, Yu Y, Nie Y, Luo W, et al. Whole-genome sequencing reveals novel tandem-duplication hotspots and a prognostic mutational signature in gastric cancer. Nat Commun. 2019;10(1):2037.

20. Zou CY, Wang J, Shen JN, Huang G, Jin S, Yin JQ, et al. Establishment and characteristics of two syngeneic human osteosarcoma cell lines from primary tumor and skip metastases. Acta Pharmacol Sin. 2008;29(3):325-32.

21. Han J, Yong B, Luo C, Tan P, Peng T, Shen J. High serum alkaline phosphatase cooperating with MMP-9 predicts metastasis and poor prognosis in patients with primary osteosarcoma in Southern China. World J Surg Oncol. 2012;10:37.

22. Szulc P, Seeman E, Delmas PD. Biochemical measurements of bone turnover in children and adolescents. Osteoporos Int. 2000;11(4):281-94.

24. Zhang T, Kwiatkowski N, Olson CM, Dixon-Clarke SE, Abraham BJ, Greifenberg AK, et al. Covalent targeting of remote cysteine residues to develop CDK12 and CDK13 inhibitors. Nat Chem Biol. 2016;12(10):876-84.

25. Jiang YY, Lin DC, Mayakonda A, Hazawa M, Ding LW, Chien WW, et al. Targeting super-enhancer-associated oncogenes in oesophageal squamous cell carcinoma. Gut. 2017;66(8):1358-68.

26. Meyers PA, Schwartz CL, Krailo M, Kleinerman ES, Betcher D, Bernstein ML, et al. Osteosarcoma: a randomized, prospective trial of the addition of ifosfamide and/or muramyl tripeptide to cisplatin, doxorubicin, and high-dose methotrexate. J Clin Oncol. 2005;23(9):2004-11.

27. Aksnes LH, Bauer HC, Jebsen NL, Follerås G, Allert C, Haugen GS, et al. Limb-sparing surgery preserves more function than amputation: a Scandinavian sarcoma group study of 118 patients. J Bone Joint Surg Br. 2008;90(6):786-94.

28. Winkler K, Beron G, Kotz R, Salzer-Kuntschik M, Beck J, Beck W, et al. Neoadjuvant chemotherapy for osteogenic sarcoma: results of a Cooperative German/Austrian study. J Clin Oncol. 1984;2(6):617-24.

29. Polites SF, Heaton TE, LaQuaglia MP, Kim ES, Barry WE, Goodhue CJ, et al. Pneumonectomy for Pediatric Tumors-a Pediatric Surgical Oncology Research Collaborative Study. Ann Surg. 2021;274(6):e605-e9.

30. Bacci G, Ferrari S, Lari S, Mercuri M, Donati D, Longhi A, et al. Osteosarcoma of the limb. Amputation or limb salvage in patients treated by neoadjuvant chemotherapy. J Bone Joint Surg Br. 2002;84(1):88-92.

31. Zhao X, Wu Q, Gong X, Liu J, Ma Y. Osteosarcoma: a review of current and future therapeutic approaches. Biomed Eng Online. 2021;20(1):24.

32. Huang H, Hu J, Maryam A, Huang Q, Zhang Y, Ramakrishnan S, et al. Defining super-enhancer landscape in triple-negative breast cancer by multiomic profiling. Nat Commun. 2021;12(1):2242.

33. Wong M, Sun Y, Xi Z, Milazzo G, Poulos RC, Bartenhagen C, et al. JMJD6 is a tumorigenic factor and therapeutic target in neuroblastoma. Nat Commun. 2019;10(1):3319.

34. Zhang L, Xiong D, Liu Q, Luo Y, Tian Y, Xiao X, et al. Genome-Wide Histone H3K27 Acetylation Profiling Identified Genes Correlated With Prognosis in Papillary Thyroid Carcinoma. Front Cell Dev Biol. 2021;9:682561.

35. Fu Y, He G, Liu Z, Wang J, Zhang Z, Bao Q, et al. Exploration and Validation of a Novel Inflammatory Response-Associated Gene Signature to Predict Osteosarcoma Prognosis and Immune Infiltration. J Inflamm Res. 2021;14:6719-34.

36. Lei T, Qian H, Lei P, Hu Y. Ferroptosis-related gene signature associates with immunity and predicts prognosis accurately in patients with osteosarcoma. Cancer Sci. 2021;112(11):4785-98.

1. Isakoff MS, Bielack SS, Meltzer P, Gorlick R. Osteosarcoma: Current Treatment and a Collaborative Pathway to Success. J Clin Oncol. 2015;33(27):3029-35.

2. Ferrari S, Smeland S, Mercuri M, Bertoni F, Longhi A, Ruggieri P, et al. Neoadjuvant chemotherapy with high-dose Ifosfamide, high-dose methotrexate, cisplatin, and doxorubicin for patients with localized osteosarcoma of the extremity: a joint study by the Italian and Scandinavian Sarcoma Groups. J Clin Oncol. 2005;23(34):8845-52.

3. Siegel RL, Miller KD, Jemal A. Cancer statistics, 2019. CA Cancer J Clin. 2019;69(1):7-34.

4. Ritter J, Bielack SS. Osteosarcoma. Ann Oncol. 2010;21 Suppl 7:vii320-5.

5. Gill J, Gorlick R. Advancing therapy for osteosarcoma. Nat Rev Clin Oncol. 2021;18(10):609-24.

6. Bielack SS, Kempf-Bielack B, Delling G, Exner GU, Flege S, Helmke K, et al. Prognostic factors in high-grade osteosarcoma of the extremities or trunk: an analysis of 1,702 patients treated on neoadjuvant cooperative osteosarcoma study group protocols. J Clin Oncol. 2002;20(3):776-90.

7. Han Z, Peng X, Yang Y, Yi J, Zhao D, Bao Q, et al. Integrated microfluidic-SERS for exosome biomarker profiling and osteosarcoma diagnosis. Biosens Bioelectron. 2022;217:114709.

8. Wu Y, Xu L, Yang P, Lin N, Huang X, Pan W, et al. Survival Prediction in High-grade Osteosarcoma Using Radiomics of Diagnostic Computed Tomography. EBioMedicine. 2018;34:27-34.

9. Chen W, Lin Y, Huang J, Yan Z, Cao H. A novel risk score model based on glycolysis-related genes and a prognostic model for predicting overall survival of osteosarcoma patients. J Orthop Res. 2022;40(10):2372-81.

10. Zhang M, Liu Y, Kong D. Identifying biomolecules and constructing a prognostic risk prediction model for recurrence in osteosarcoma. J Bone Oncol. 2021;26:100331.

11. Li L, Wang Y, He X, Li Z, Lu M, Gong T, et al. Hematological Prognostic Scoring System Can Predict Overall Survival and Can Indicate Response to Immunotherapy in Patients With Osteosarcoma. Front Immunol. 2022;13:879560.

12. Pelish HE, Liau BB, Nitulescu, II, Tangpeerachaikul A, Poss ZC, Da Silva DH, et al. Mediator kinase inhibition further activates super-enhancer-associated genes in AML. Nature. 2015;526(7572):273-6.

13. Whyte WA, Orlando DA, Hnisz D, Abraham BJ, Lin CY, Kagey MH, et al. Master transcription factors and mediator establish super-enhancers at key cell identity genes. Cell. 2013;153(2):307-19.

15. Mansour MR, Abraham BJ, Anders L, Berezovskaya A, Gutierrez A, Durbin AD, et al. Oncogene regulation. An oncogenic super-enhancer formed through somatic mutation of a noncoding intergenic element. Science. 2014;346(6215):1373-7.

16. Lovén J, Hoke HA, Lin CY, Lau A, Orlando DA, Vakoc CR, et al. Selective inhibition of tumor oncogenes by disruption of super-enhancers. Cell. 2013;153(2):320-34.

17. Wang Y, Zhang T, Kwiatkowski N, Abraham BJ, Lee TI, Xie S, et al. CDK7-dependent transcriptional addiction in triple-negative breast cancer. Cell. 2015;163(1):174-86.

18. Zhang J, Liu W, Zou C, Zhao Z, Lai Y, Shi Z, et al. Targeting Super-Enhancer-Associated Oncogenes in Osteosarcoma with THZ2, a Covalent CDK7 Inhibitor. Clin Cancer Res. 2020;26(11):2681-92.

19. Xing R, Zhou Y, Yu J, Yu Y, Nie Y, Luo W, et al. Whole-genome sequencing reveals novel tandem-duplication hotspots and a prognostic mutational signature in gastric cancer. Nat Commun. 2019;10(1):2037.

20. Zou CY, Wang J, Shen JN, Huang G, Jin S, Yin JQ, et al. Establishment and characteristics of two syngeneic human osteosarcoma cell lines from primary tumor and skip metastases. Acta Pharmacol Sin. 2008;29(3):325-32.

21. Han J, Yong B, Luo C, Tan P, Peng T, Shen J. High serum alkaline phosphatase cooperating with MMP-9 predicts metastasis and poor prognosis in patients with primary osteosarcoma in Southern China. World J Surg Oncol. 2012;10:37.

22. Szulc P, Seeman E, Delmas PD. Biochemical measurements of bone turnover in children and adolescents. Osteoporos Int. 2000;11(4):281-94.

23. Camp RL, Dolled-Filhart M, Rimm DL. X-tile: a new bio-informatics tool for biomarker assessment and outcome-based cut-point optimization. Clin Cancer Res. 2004;10(21):7252-9.

24. Zhang T, Kwiatkowski N, Olson CM, Dixon-Clarke SE, Abraham BJ, Greifenberg AK, et al. Covalent targeting of remote cysteine residues to develop CDK12 and CDK13 inhibitors. Nat Chem Biol. 2016;12(10):876-84.

25. Jiang YY, Lin DC, Mayakonda A, Hazawa M, Ding LW, Chien WW, et al. Targeting super-enhancer-associated oncogenes in oesophageal squamous cell carcinoma. Gut. 2017;66(8):1358-68.

26. Meyers PA, Schwartz CL, Krailo M, Kleinerman ES, Betcher D, Bernstein ML, et al. Osteosarcoma: a randomized, prospective trial of the addition of ifosfamide and/or muramyl tripeptide to cisplatin, doxorubicin, and high-dose methotrexate. J Clin Oncol. 2005;23(9):2004-11.

27. Aksnes LH, Bauer HC, Jebsen NL, Follerås G, Allert C, Haugen GS, et al. Limb-sparing surgery preserves more function than amputation: a Scandinavian sarcoma group study of 118 patients. J Bone Joint Surg Br. 2008;90(6):786-94.

28. Winkler K, Beron G, Kotz R, Salzer-Kuntschik M, Beck J, Beck W, et al. Neoadjuvant chemotherapy for osteogenic sarcoma: results of a Cooperative German/Austrian study. J Clin Oncol. 1984;2(6):617-24.

29. Polites SF, Heaton TE, LaQuaglia MP, Kim ES, Barry WE, Goodhue CJ, et al. Pneumonectomy for Pediatric Tumors-a Pediatric Surgical Oncology Research Collaborative Study. Ann Surg. 2021;274(6):e605-e9.

30. Bacci G, Ferrari S, Lari S, Mercuri M, Donati D, Longhi A, et al. Osteosarcoma of the limb. Amputation or limb salvage in patients treated by neoadjuvant chemotherapy. J Bone Joint Surg Br. 2002;84(1):88-92.

31. Zhao X, Wu Q, Gong X, Liu J, Ma Y. Osteosarcoma: a review of current and future therapeutic approaches. Biomed Eng Online. 2021;20(1):24.

32. Huang H, Hu J, Maryam A, Huang Q, Zhang Y, Ramakrishnan S, et al. Defining super-enhancer landscape in triple-negative breast cancer by multiomic profiling. Nat Commun. 2021;12(1):2242.

33. Wong M, Sun Y, Xi Z, Milazzo G, Poulos RC, Bartenhagen C, et al. JMJD6 is a tumorigenic factor and therapeutic target in neuroblastoma. Nat Commun. 2019;10(1):3319.

34. Zhang L, Xiong D, Liu Q, Luo Y, Tian Y, Xiao X, et al. Genome-Wide Histone H3K27 Acetylation Profiling Identified Genes Correlated With Prognosis in Papillary Thyroid Carcinoma. Front Cell Dev Biol. 2021;9:682561.

35. Fu Y, He G, Liu Z, Wang J, Zhang Z, Bao Q, et al. Exploration and Validation of a Novel Inflammatory Response-Associated Gene Signature to Predict Osteosarcoma Prognosis and Immune Infiltration. J Inflamm Res. 2021;14:6719-34.

36. Lei T, Qian H, Lei P, Hu Y. Ferroptosis-related gene signature associates with immunity and predicts prognosis accurately in patients with osteosarcoma. Cancer Sci. 2021;112(11):4785-98.

37. Ouyang Z, Li G, Zhu H, Wang J, Qi T, Qu Q, et al. Construction of a Five-Super-Enhancer-Associated-Genes Prognostic Model for Osteosarcoma Patients. Front Cell Dev Biol. 2020;8:598660.

1. Isakoff MS, Bielack SS, Meltzer P, Gorlick R. Osteosarcoma: Current Treatment and a Collaborative Pathway to Success. J Clin Oncol. 2015;33(27):3029-35.

2. Ferrari S, Smeland S, Mercuri M, Bertoni F, Longhi A, Ruggieri P, et al. Neoadjuvant chemotherapy with high-dose Ifosfamide, high-dose methotrexate, cisplatin, and doxorubicin for patients with localized osteosarcoma of the extremity: a joint study by the Italian and Scandinavian Sarcoma Groups. J Clin Oncol. 2005;23(34):8845-52.

3. Siegel RL, Miller KD, Jemal A. Cancer statistics, 2019. CA Cancer J Clin. 2019;69(1):7-34.

4. Ritter J, Bielack SS. Osteosarcoma. Ann Oncol. 2010;21 Suppl 7:vii320-5.

5. Gill J, Gorlick R. Advancing therapy for osteosarcoma. Nat Rev Clin Oncol. 2021;18(10):609-24.

6. Bielack SS, Kempf-Bielack B, Delling G, Exner GU, Flege S, Helmke K, et al. Prognostic factors in high-grade osteosarcoma of the extremities or trunk: an analysis of 1,702 patients treated on neoadjuvant cooperative osteosarcoma study group protocols. J Clin Oncol. 2002;20(3):776-90.

7. Han Z, Peng X, Yang Y, Yi J, Zhao D, Bao Q, et al. Integrated microfluidic-SERS for exosome biomarker profiling and osteosarcoma diagnosis. Biosens Bioelectron. 2022;217:114709.

8. Wu Y, Xu L, Yang P, Lin N, Huang X, Pan W, et al. Survival Prediction in High-grade Osteosarcoma Using Radiomics of Diagnostic Computed Tomography. EBioMedicine. 2018;34:27-34.

9. Chen W, Lin Y, Huang J, Yan Z, Cao H. A novel risk score model based on glycolysis-related genes and a prognostic model for predicting overall survival of osteosarcoma patients. J Orthop Res. 2022;40(10):2372-81.

10. Zhang M, Liu Y, Kong D. Identifying biomolecules and constructing a prognostic risk prediction model for recurrence in osteosarcoma. J Bone Oncol. 2021;26:100331.

11. Li L, Wang Y, He X, Li Z, Lu M, Gong T, et al. Hematological Prognostic Scoring System Can Predict Overall Survival and Can Indicate Response to Immunotherapy in Patients With Osteosarcoma. Front Immunol. 2022;13:879560.

12. Pelish HE, Liau BB, Nitulescu, II, Tangpeerachaikul A, Poss ZC, Da Silva DH, et al. Mediator kinase inhibition further activates super-enhancer-associated genes in AML. Nature. 2015;526(7572):273-6.

13. Whyte WA, Orlando DA, Hnisz D, Abraham BJ, Lin CY, Kagey MH, et al. Master transcription factors and mediator establish super-enhancers at key cell identity genes. Cell. 2013;153(2):307-19.

14. Tögel L, Nightingale R, Chueh AC, Jayachandran A, Tran H, Phesse T, et al. Dual Targeting of Bromodomain and Extraterminal Domain Proteins, and WNT or MAPK Signaling, Inhibits c-MYC Expression and Proliferation of Colorectal Cancer Cells. Mol Cancer Ther. 2016;15(6):1217-26.

15. Mansour MR, Abraham BJ, Anders L, Berezovskaya A, Gutierrez A, Durbin AD, et al. Oncogene regulation. An oncogenic super-enhancer formed through somatic mutation of a noncoding intergenic element. Science. 2014;346(6215):1373-7.

16. Lovén J, Hoke HA, Lin CY, Lau A, Orlando DA, Vakoc CR, et al. Selective inhibition of tumor oncogenes by disruption of super-enhancers. Cell. 2013;153(2):320-34.

17. Wang Y, Zhang T, Kwiatkowski N, Abraham BJ, Lee TI, Xie S, et al. CDK7-dependent transcriptional addiction in triple-negative breast cancer. Cell. 2015;163(1):174-86.

18. Zhang J, Liu W, Zou C, Zhao Z, Lai Y, Shi Z, et al. Targeting Super-Enhancer-Associated Oncogenes in Osteosarcoma with THZ2, a Covalent CDK7 Inhibitor. Clin Cancer Res. 2020;26(11):2681-92.

19. Xing R, Zhou Y, Yu J, Yu Y, Nie Y, Luo W, et al. Whole-genome sequencing reveals novel tandem-duplication hotspots and a prognostic mutational signature in gastric cancer. Nat Commun. 2019;10(1):2037.

20. Zou CY, Wang J, Shen JN, Huang G, Jin S, Yin JQ, et al. Establishment and characteristics of two syngeneic human osteosarcoma cell lines from primary tumor and skip metastases. Acta Pharmacol Sin. 2008;29(3):325-32.

21. Han J, Yong B, Luo C, Tan P, Peng T, Shen J. High serum alkaline phosphatase cooperating with MMP-9 predicts metastasis and poor prognosis in patients with primary osteosarcoma in Southern China. World J Surg Oncol. 2012;10:37.

22. Szulc P, Seeman E, Delmas PD. Biochemical measurements of bone turnover in children and adolescents. Osteoporos Int. 2000;11(4):281-94.

23. Camp RL, Dolled-Filhart M, Rimm DL. X-tile: a new bio-informatics tool for biomarker assessment and outcome-based cut-point optimization. Clin Cancer Res. 2004;10(21):7252-9.

24. Zhang T, Kwiatkowski N, Olson CM, Dixon-Clarke SE, Abraham BJ, Greifenberg AK, et al. Covalent targeting of remote cysteine residues to develop CDK12 and CDK13 inhibitors. Nat Chem Biol. 2016;12(10):876-84.

25. Jiang YY, Lin DC, Mayakonda A, Hazawa M, Ding LW, Chien WW, et al. Targeting super-enhancer-associated oncogenes in oesophageal squamous cell carcinoma. Gut. 2017;66(8):1358-68.

26. Meyers PA, Schwartz CL, Krailo M, Kleinerman ES, Betcher D, Bernstein ML, et al. Osteosarcoma: a randomized, prospective trial of the addition of ifosfamide and/or muramyl tripeptide to cisplatin, doxorubicin, and high-dose methotrexate. J Clin Oncol. 2005;23(9):2004-11.

27. Aksnes LH, Bauer HC, Jebsen NL, Follerås G, Allert C, Haugen GS, et al. Limb-sparing surgery preserves more function than amputation: a Scandinavian sarcoma group study of 118 patients. J Bone Joint Surg Br. 2008;90(6):786-94.

28. Winkler K, Beron G, Kotz R, Salzer-Kuntschik M, Beck J, Beck W, et al. Neoadjuvant chemotherapy for osteogenic sarcoma: results of a Cooperative German/Austrian study. J Clin Oncol. 1984;2(6):617-24.

29. Polites SF, Heaton TE, LaQuaglia MP, Kim ES, Barry WE, Goodhue CJ, et al. Pneumonectomy for Pediatric Tumors-a Pediatric Surgical Oncology Research Collaborative Study. Ann Surg. 2021;274(6):e605-e9.

30. Bacci G, Ferrari S, Lari S, Mercuri M, Donati D, Longhi A, et al. Osteosarcoma of the limb. Amputation or limb salvage in patients treated by neoadjuvant chemotherapy. J Bone Joint Surg Br. 2002;84(1):88-92.

31. Zhao X, Wu Q, Gong X, Liu J, Ma Y. Osteosarcoma: a review of current and future therapeutic approaches. Biomed Eng Online. 2021;20(1):24.

32. Huang H, Hu J, Maryam A, Huang Q, Zhang Y, Ramakrishnan S, et al. Defining super-enhancer landscape in triple-negative breast cancer by multiomic profiling. Nat Commun. 2021;12(1):2242.

33. Wong M, Sun Y, Xi Z, Milazzo G, Poulos RC, Bartenhagen C, et al. JMJD6 is a tumorigenic factor and therapeutic target in neuroblastoma. Nat Commun. 2019;10(1):3319.

34. Zhang L, Xiong D, Liu Q, Luo Y, Tian Y, Xiao X, et al. Genome-Wide Histone H3K27 Acetylation Profiling Identified Genes Correlated With Prognosis in Papillary Thyroid Carcinoma. Front Cell Dev Biol. 2021;9:682561.

35. Fu Y, He G, Liu Z, Wang J, Zhang Z, Bao Q, et al. Exploration and Validation of a Novel Inflammatory Response-Associated Gene Signature to Predict Osteosarcoma Prognosis and Immune Infiltration. J Inflamm Res. 2021;14:6719-34.

36. Lei T, Qian H, Lei P, Hu Y. Ferroptosis-related gene signature associates with immunity and predicts prognosis accurately in patients with osteosarcoma. Cancer Sci. 2021;112(11):4785-98.

37. Ouyang Z, Li G, Zhu H, Wang J, Qi T, Qu Q, et al. Construction of a Five-Super-Enhancer-Associated-Genes Prognostic Model for Osteosarcoma Patients. Front Cell Dev Biol. 2020;8:598660.

38. Ferrari S, Bertoni F, Mercuri M, Picci P, Giacomini S, Longhi A, et al. Predictive factors of disease-free survival for non-metastatic osteosarcoma of the extremity: an analysis of 300 patients treated at the Rizzoli Institute. Ann Oncol. 2001;12(8):1145-50.

1. Isakoff MS, Bielack SS, Meltzer P, Gorlick R. Osteosarcoma: Current Treatment and a Collaborative Pathway to Success. J Clin Oncol. 2015;33(27):3029-35.

2. Ferrari S, Smeland S, Mercuri M, Bertoni F, Longhi A, Ruggieri P, et al. Neoadjuvant chemotherapy with high-dose Ifosfamide, high-dose methotrexate, cisplatin, and doxorubicin for patients with localized osteosarcoma of the extremity: a joint study by the Italian and Scandinavian Sarcoma Groups. J Clin Oncol. 2005;23(34):8845-52.

3. Siegel RL, Miller KD, Jemal A. Cancer statistics, 2019. CA Cancer J Clin. 2019;69(1):7-34.

4. Ritter J, Bielack SS. Osteosarcoma. Ann Oncol. 2010;21 Suppl 7:vii320-5.

5. Gill J, Gorlick R. Advancing therapy for osteosarcoma. Nat Rev Clin Oncol. 2021;18(10):609-24.

6. Bielack SS, Kempf-Bielack B, Delling G, Exner GU, Flege S, Helmke K, et al. Prognostic factors in high-grade osteosarcoma of the extremities or trunk: an analysis of 1,702 patients treated on neoadjuvant cooperative osteosarcoma study group protocols. J Clin Oncol. 2002;20(3):776-90.

7. Han Z, Peng X, Yang Y, Yi J, Zhao D, Bao Q, et al. Integrated microfluidic-SERS for exosome biomarker profiling and osteosarcoma diagnosis. Biosens Bioelectron. 2022;217:114709.

8. Wu Y, Xu L, Yang P, Lin N, Huang X, Pan W, et al. Survival Prediction in High-grade Osteosarcoma Using Radiomics of Diagnostic Computed Tomography. EBioMedicine. 2018;34:27-34.

9. Chen W, Lin Y, Huang J, Yan Z, Cao H. A novel risk score model based on glycolysis-related genes and a prognostic model for predicting overall survival of osteosarcoma patients. J Orthop Res. 2022;40(10):2372-81.

10. Zhang M, Liu Y, Kong D. Identifying biomolecules and constructing a prognostic risk prediction model for recurrence in osteosarcoma. J Bone Oncol. 2021;26:100331.

11. Li L, Wang Y, He X, Li Z, Lu M, Gong T, et al. Hematological Prognostic Scoring System Can Predict Overall Survival and Can Indicate Response to Immunotherapy in Patients With Osteosarcoma. Front Immunol. 2022;13:879560.

12. Pelish HE, Liau BB, Nitulescu, II, Tangpeerachaikul A, Poss ZC, Da Silva DH, et al. Mediator kinase inhibition further activates super-enhancer-associated genes in AML. Nature. 2015;526(7572):273-6.

13. Whyte WA, Orlando DA, Hnisz D, Abraham BJ, Lin CY, Kagey MH, et al. Master transcription factors and mediator establish super-enhancers at key cell identity genes. Cell. 2013;153(2):307-19.

14. Tögel L, Nightingale R, Chueh AC, Jayachandran A, Tran H, Phesse T, et al. Dual Targeting of Bromodomain and Extraterminal Domain Proteins, and WNT or MAPK Signaling, Inhibits c-MYC Expression and Proliferation of Colorectal Cancer Cells. Mol Cancer Ther. 2016;15(6):1217-26.

15. Mansour MR, Abraham BJ, Anders L, Berezovskaya A, Gutierrez A, Durbin AD, et al. Oncogene regulation. An oncogenic super-enhancer formed through somatic mutation of a noncoding intergenic element. Science. 2014;346(6215):1373-7.

16. Lovén J, Hoke HA, Lin CY, Lau A, Orlando DA, Vakoc CR, et al. Selective inhibition of tumor oncogenes by disruption of super-enhancers. Cell. 2013;153(2):320-34.

17. Wang Y, Zhang T, Kwiatkowski N, Abraham BJ, Lee TI, Xie S, et al. CDK7-dependent transcriptional addiction in triple-negative breast cancer. Cell. 2015;163(1):174-86.

18. Zhang J, Liu W, Zou C, Zhao Z, Lai Y, Shi Z, et al. Targeting Super-Enhancer-Associated Oncogenes in Osteosarcoma with THZ2, a Covalent CDK7 Inhibitor. Clin Cancer Res. 2020;26(11):2681-92.

19. Xing R, Zhou Y, Yu J, Yu Y, Nie Y, Luo W, et al. Whole-genome sequencing reveals novel tandem-duplication hotspots and a prognostic mutational signature in gastric cancer. Nat Commun. 2019;10(1):2037.

20. Zou CY, Wang J, Shen JN, Huang G, Jin S, Yin JQ, et al. Establishment and characteristics of two syngeneic human osteosarcoma cell lines from primary tumor and skip metastases. Acta Pharmacol Sin. 2008;29(3):325-32.

21. Han J, Yong B, Luo C, Tan P, Peng T, Shen J. High serum alkaline phosphatase cooperating with MMP-9 predicts metastasis and poor prognosis in patients with primary osteosarcoma in Southern China. World J Surg Oncol. 2012;10:37.

22. Szulc P, Seeman E, Delmas PD. Biochemical measurements of bone turnover in children and adolescents. Osteoporos Int. 2000;11(4):281-94.

23. Camp RL, Dolled-Filhart M, Rimm DL. X-tile: a new bio-informatics tool for biomarker assessment and outcome-based cut-point optimization. Clin Cancer Res. 2004;10(21):7252-9.

24. Zhang T, Kwiatkowski N, Olson CM, Dixon-Clarke SE, Abraham BJ, Greifenberg AK, et al. Covalent targeting of remote cysteine residues to develop CDK12 and CDK13 inhibitors. Nat Chem Biol. 2016;12(10):876-84.

25. Jiang YY, Lin DC, Mayakonda A, Hazawa M, Ding LW, Chien WW, et al. Targeting super-enhancer-associated oncogenes in oesophageal squamous cell carcinoma. Gut. 2017;66(8):1358-68.

26. Meyers PA, Schwartz CL, Krailo M, Kleinerman ES, Betcher D, Bernstein ML, et al. Osteosarcoma: a randomized, prospective trial of the addition of ifosfamide and/or muramyl tripeptide to cisplatin, doxorubicin, and high-dose methotrexate. J Clin Oncol. 2005;23(9):2004-11.

27. Aksnes LH, Bauer HC, Jebsen NL, Follerås G, Allert C, Haugen GS, et al. Limb-sparing surgery preserves more function than amputation: a Scandinavian sarcoma group study of 118 patients. J Bone Joint Surg Br. 2008;90(6):786-94.

28. Winkler K, Beron G, Kotz R, Salzer-Kuntschik M, Beck J, Beck W, et al. Neoadjuvant chemotherapy for osteogenic sarcoma: results of a Cooperative German/Austrian study. J Clin Oncol. 1984;2(6):617-24.

29. Polites SF, Heaton TE, LaQuaglia MP, Kim ES, Barry WE, Goodhue CJ, et al. Pneumonectomy for Pediatric Tumors-a Pediatric Surgical Oncology Research Collaborative Study. Ann Surg. 2021;274(6):e605-e9.

30. Bacci G, Ferrari S, Lari S, Mercuri M, Donati D, Longhi A, et al. Osteosarcoma of the limb. Amputation or limb salvage in patients treated by neoadjuvant chemotherapy. J Bone Joint Surg Br. 2002;84(1):88-92.

31. Zhao X, Wu Q, Gong X, Liu J, Ma Y. Osteosarcoma: a review of current and future therapeutic approaches. Biomed Eng Online. 2021;20(1):24.

32. Huang H, Hu J, Maryam A, Huang Q, Zhang Y, Ramakrishnan S, et al. Defining super-enhancer landscape in triple-negative breast cancer by multiomic profiling. Nat Commun. 2021;12(1):2242.

33. Wong M, Sun Y, Xi Z, Milazzo G, Poulos RC, Bartenhagen C, et al. JMJD6 is a tumorigenic factor and therapeutic target in neuroblastoma. Nat Commun. 2019;10(1):3319.

34. Zhang L, Xiong D, Liu Q, Luo Y, Tian Y, Xiao X, et al. Genome-Wide Histone H3K27 Acetylation Profiling Identified Genes Correlated With Prognosis in Papillary Thyroid Carcinoma. Front Cell Dev Biol. 2021;9:682561.

35. Fu Y, He G, Liu Z, Wang J, Zhang Z, Bao Q, et al. Exploration and Validation of a Novel Inflammatory Response-Associated Gene Signature to Predict Osteosarcoma Prognosis and Immune Infiltration. J Inflamm Res. 2021;14:6719-34.

36. Lei T, Qian H, Lei P, Hu Y. Ferroptosis-related gene signature associates with immunity and predicts prognosis accurately in patients with osteosarcoma. Cancer Sci. 2021;112(11):4785-98.

37. Ouyang Z, Li G, Zhu H, Wang J, Qi T, Qu Q, et al. Construction of a Five-Super-Enhancer-Associated-Genes Prognostic Model for Osteosarcoma Patients. Front Cell Dev Biol. 2020;8:598660.

38. Ferrari S, Bertoni F, Mercuri M, Picci P, Giacomini S, Longhi A, et al. Predictive factors of disease-free survival for non-metastatic osteosarcoma of the extremity: an analysis of 300 patients treated at the Rizzoli Institute. Ann Oncol. 2001;12(8):1145-50.

1. Isakoff MS, Bielack SS, Meltzer P, Gorlick R. Osteosarcoma: Current Treatment and a Collaborative Pathway to Success. J Clin Oncol. 2015;33(27):3029-35.

2. Ferrari S, Smeland S, Mercuri M, Bertoni F, Longhi A, Ruggieri P, et al. Neoadjuvant chemotherapy with high-dose Ifosfamide, high-dose methotrexate, cisplatin, and doxorubicin for patients with localized osteosarcoma of the extremity: a joint study by the Italian and Scandinavian Sarcoma Groups. J Clin Oncol. 2005;23(34):8845-52.

3. Siegel RL, Miller KD, Jemal A. Cancer statistics, 2019. CA Cancer J Clin. 2019;69(1):7-34.

4. Ritter J, Bielack SS. Osteosarcoma. Ann Oncol. 2010;21 Suppl 7:vii320-5.

5. Gill J, Gorlick R. Advancing therapy for osteosarcoma. Nat Rev Clin Oncol. 2021;18(10):609-24.

6. Bielack SS, Kempf-Bielack B, Delling G, Exner GU, Flege S, Helmke K, et al. Prognostic factors in high-grade osteosarcoma of the extremities or trunk: an analysis of 1,702 patients treated on neoadjuvant cooperative osteosarcoma study group protocols. J Clin Oncol. 2002;20(3):776-90.

7. Han Z, Peng X, Yang Y, Yi J, Zhao D, Bao Q, et al. Integrated microfluidic-SERS for exosome biomarker profiling and osteosarcoma diagnosis. Biosens Bioelectron. 2022;217:114709.

8. Wu Y, Xu L, Yang P, Lin N, Huang X, Pan W, et al. Survival Prediction in High-grade Osteosarcoma Using Radiomics of Diagnostic Computed Tomography. EBioMedicine. 2018;34:27-34.

9. Chen W, Lin Y, Huang J, Yan Z, Cao H. A novel risk score model based on glycolysis-related genes and a prognostic model for predicting overall survival of osteosarcoma patients. J Orthop Res. 2022;40(10):2372-81.

10. Zhang M, Liu Y, Kong D. Identifying biomolecules and constructing a prognostic risk prediction model for recurrence in osteosarcoma. J Bone Oncol. 2021;26:100331.

11. Li L, Wang Y, He X, Li Z, Lu M, Gong T, et al. Hematological Prognostic Scoring System Can Predict Overall Survival and Can Indicate Response to Immunotherapy in Patients With Osteosarcoma. Front Immunol. 2022;13:879560.

12. Pelish HE, Liau BB, Nitulescu, II, Tangpeerachaikul A, Poss ZC, Da Silva DH, et al. Mediator kinase inhibition further activates super-enhancer-associated genes in AML. Nature. 2015;526(7572):273-6.

13. Whyte WA, Orlando DA, Hnisz D, Abraham BJ, Lin CY, Kagey MH, et al. Master transcription factors and mediator establish super-enhancers at key cell identity genes. Cell. 2013;153(2):307-19.

14. Tögel L, Nightingale R, Chueh AC, Jayachandran A, Tran H, Phesse T, et al. Dual Targeting of Bromodomain and Extraterminal Domain Proteins, and WNT or MAPK Signaling, Inhibits c-MYC Expression and Proliferation of Colorectal Cancer Cells. Mol Cancer Ther. 2016;15(6):1217-26.

15. Mansour MR, Abraham BJ, Anders L, Berezovskaya A, Gutierrez A, Durbin AD, et al. Oncogene regulation. An oncogenic super-enhancer formed through somatic mutation of a noncoding intergenic element. Science. 2014;346(6215):1373-7.

16. Lovén J, Hoke HA, Lin CY, Lau A, Orlando DA, Vakoc CR, et al. Selective inhibition of tumor oncogenes by disruption of super-enhancers. Cell. 2013;153(2):320-34.

17. Wang Y, Zhang T, Kwiatkowski N, Abraham BJ, Lee TI, Xie S, et al. CDK7-dependent transcriptional addiction in triple-negative breast cancer. Cell. 2015;163(1):174-86.

18. Zhang J, Liu W, Zou C, Zhao Z, Lai Y, Shi Z, et al. Targeting Super-Enhancer-Associated Oncogenes in Osteosarcoma with THZ2, a Covalent CDK7 Inhibitor. Clin Cancer Res. 2020;26(11):2681-92.

19. Xing R, Zhou Y, Yu J, Yu Y, Nie Y, Luo W, et al. Whole-genome sequencing reveals novel tandem-duplication hotspots and a prognostic mutational signature in gastric cancer. Nat Commun. 2019;10(1):2037.

20. Zou CY, Wang J, Shen JN, Huang G, Jin S, Yin JQ, et al. Establishment and characteristics of two syngeneic human osteosarcoma cell lines from primary tumor and skip metastases. Acta Pharmacol Sin. 2008;29(3):325-32.

21. Han J, Yong B, Luo C, Tan P, Peng T, Shen J. High serum alkaline phosphatase cooperating with MMP-9 predicts metastasis and poor prognosis in patients with primary osteosarcoma in Southern China. World J Surg Oncol. 2012;10:37.

22. Szulc P, Seeman E, Delmas PD. Biochemical measurements of bone turnover in children and adolescents. Osteoporos Int. 2000;11(4):281-94.

23. Camp RL, Dolled-Filhart M, Rimm DL. X-tile: a new bio-informatics tool for biomarker assessment and outcome-based cut-point optimization. Clin Cancer Res. 2004;10(21):7252-9.

24. Zhang T, Kwiatkowski N, Olson CM, Dixon-Clarke SE, Abraham BJ, Greifenberg AK, et al. Covalent targeting of remote cysteine residues to develop CDK12 and CDK13 inhibitors. Nat Chem Biol. 2016;12(10):876-84.

25. Jiang YY, Lin DC, Mayakonda A, Hazawa M, Ding LW, Chien WW, et al. Targeting super-enhancer-associated oncogenes in oesophageal squamous cell carcinoma. Gut. 2017;66(8):1358-68.

26. Meyers PA, Schwartz CL, Krailo M, Kleinerman ES, Betcher D, Bernstein ML, et al. Osteosarcoma: a randomized, prospective trial of the addition of ifosfamide and/or muramyl tripeptide to cisplatin, doxorubicin, and high-dose methotrexate. J Clin Oncol. 2005;23(9):2004-11.

27. Aksnes LH, Bauer HC, Jebsen NL, Follerås G, Allert C, Haugen GS, et al. Limb-sparing surgery preserves more function than amputation: a Scandinavian sarcoma group study of 118 patients. J Bone Joint Surg Br. 2008;90(6):786-94.

28. Winkler K, Beron G, Kotz R, Salzer-Kuntschik M, Beck J, Beck W, et al. Neoadjuvant chemotherapy for osteogenic sarcoma: results of a Cooperative German/Austrian study. J Clin Oncol. 1984;2(6):617-24.

29. Polites SF, Heaton TE, LaQuaglia MP, Kim ES, Barry WE, Goodhue CJ, et al. Pneumonectomy for Pediatric Tumors-a Pediatric Surgical Oncology Research Collaborative Study. Ann Surg. 2021;274(6):e605-e9.

30. Bacci G, Ferrari S, Lari S, Mercuri M, Donati D, Longhi A, et al. Osteosarcoma of the limb. Amputation or limb salvage in patients treated by neoadjuvant chemotherapy. J Bone Joint Surg Br. 2002;84(1):88-92.

31. Zhao X, Wu Q, Gong X, Liu J, Ma Y. Osteosarcoma: a review of current and future therapeutic approaches. Biomed Eng Online. 2021;20(1):24.

32. Huang H, Hu J, Maryam A, Huang Q, Zhang Y, Ramakrishnan S, et al. Defining super-enhancer landscape in triple-negative breast cancer by multiomic profiling. Nat Commun. 2021;12(1):2242.

33. Wong M, Sun Y, Xi Z, Milazzo G, Poulos RC, Bartenhagen C, et al. JMJD6 is a tumorigenic factor and therapeutic target in neuroblastoma. Nat Commun. 2019;10(1):3319.

34. Zhang L, Xiong D, Liu Q, Luo Y, Tian Y, Xiao X, et al. Genome-Wide Histone H3K27 Acetylation Profiling Identified Genes Correlated With Prognosis in Papillary Thyroid Carcinoma. Front Cell Dev Biol. 2021;9:682561.

35. Fu Y, He G, Liu Z, Wang J, Zhang Z, Bao Q, et al. Exploration and Validation of a Novel Inflammatory Response-Associated Gene Signature to Predict Osteosarcoma Prognosis and Immune Infiltration. J Inflamm Res. 2021;14:6719-34.

36. Lei T, Qian H, Lei P, Hu Y. Ferroptosis-related gene signature associates with immunity and predicts prognosis accurately in patients with osteosarcoma. Cancer Sci. 2021;112(11):4785-98.

37. Ouyang Z, Li G, Zhu H, Wang J, Qi T, Qu Q, et al. Construction of a Five-Super-Enhancer-Associated-Genes Prognostic Model for Osteosarcoma Patients. Front Cell Dev Biol. 2020;8:598660.

38. Ferrari S, Bertoni F, Mercuri M, Picci P, Giacomini S, Longhi A, et al. Predictive factors of disease-free survival for non-metastatic osteosarcoma of the extremity: an analysis of 300 patients treated at the Rizzoli Institute. Ann Oncol. 2001;12(8):1145-50.

39. Bacci G, Longhi A, Ferrari S, Briccoli A, Donati D, De Paolis M, et al. Prognostic significance of serum lactate dehydrogenase in osteosarcoma of the extremity: experience at Rizzoli on 1421 patients treated over the last 30 years. Tumori. 2004;90(5):478-84.

1. Isakoff MS, Bielack SS, Meltzer P, Gorlick R. Osteosarcoma: Current Treatment and a Collaborative Pathway to Success. J Clin Oncol. 2015;33(27):3029-35.

2. Ferrari S, Smeland S, Mercuri M, Bertoni F, Longhi A, Ruggieri P, et al. Neoadjuvant chemotherapy with high-dose Ifosfamide, high-dose methotrexate, cisplatin, and doxorubicin for patients with localized osteosarcoma of the extremity: a joint study by the Italian and Scandinavian Sarcoma Groups. J Clin Oncol. 2005;23(34):8845-52.

3. Siegel RL, Miller KD, Jemal A. Cancer statistics, 2019. CA Cancer J Clin. 2019;69(1):7-34.

4. Ritter J, Bielack SS. Osteosarcoma. Ann Oncol. 2010;21 Suppl 7:vii320-5.

5. Gill J, Gorlick R. Advancing therapy for osteosarcoma. Nat Rev Clin Oncol. 2021;18(10):609-24.

6. Bielack SS, Kempf-Bielack B, Delling G, Exner GU, Flege S, Helmke K, et al. Prognostic factors in high-grade osteosarcoma of the extremities or trunk: an analysis of 1,702 patients treated on neoadjuvant cooperative osteosarcoma study group protocols. J Clin Oncol. 2002;20(3):776-90.

7. Han Z, Peng X, Yang Y, Yi J, Zhao D, Bao Q, et al. Integrated microfluidic-SERS for exosome biomarker profiling and osteosarcoma diagnosis. Biosens Bioelectron. 2022;217:114709.

8. Wu Y, Xu L, Yang P, Lin N, Huang X, Pan W, et al. Survival Prediction in High-grade Osteosarcoma Using Radiomics of Diagnostic Computed Tomography. EBioMedicine. 2018;34:27-34.

9. Chen W, Lin Y, Huang J, Yan Z, Cao H. A novel risk score model based on glycolysis-related genes and a prognostic model for predicting overall survival of osteosarcoma patients. J Orthop Res. 2022;40(10):2372-81.

10. Zhang M, Liu Y, Kong D. Identifying biomolecules and constructing a prognostic risk prediction model for recurrence in osteosarcoma. J Bone Oncol. 2021;26:100331.

11. Li L, Wang Y, He X, Li Z, Lu M, Gong T, et al. Hematological Prognostic Scoring System Can Predict Overall Survival and Can Indicate Response to Immunotherapy in Patients With Osteosarcoma. Front Immunol. 2022;13:879560.

12. Pelish HE, Liau BB, Nitulescu, II, Tangpeerachaikul A, Poss ZC, Da Silva DH, et al. Mediator kinase inhibition further activates super-enhancer-associated genes in AML. Nature. 2015;526(7572):273-6.

13. Whyte WA, Orlando DA, Hnisz D, Abraham BJ, Lin CY, Kagey MH, et al. Master transcription factors and mediator establish super-enhancers at key cell identity genes. Cell. 2013;153(2):307-19.

14. Tögel L, Nightingale R, Chueh AC, Jayachandran A, Tran H, Phesse T, et al. Dual Targeting of Bromodomain and Extraterminal Domain Proteins, and WNT or MAPK Signaling, Inhibits c-MYC Expression and Proliferation of Colorectal Cancer Cells. Mol Cancer Ther. 2016;15(6):1217-26.

15. Mansour MR, Abraham BJ, Anders L, Berezovskaya A, Gutierrez A, Durbin AD, et al. Oncogene regulation. An oncogenic super-enhancer formed through somatic mutation of a noncoding intergenic element. Science. 2014;346(6215):1373-7.

16. Lovén J, Hoke HA, Lin CY, Lau A, Orlando DA, Vakoc CR, et al. Selective inhibition of tumor oncogenes by disruption of super-enhancers. Cell. 2013;153(2):320-34.

17. Wang Y, Zhang T, Kwiatkowski N, Abraham BJ, Lee TI, Xie S, et al. CDK7-dependent transcriptional addiction in triple-negative breast cancer. Cell. 2015;163(1):174-86.

18. Zhang J, Liu W, Zou C, Zhao Z, Lai Y, Shi Z, et al. Targeting Super-Enhancer-Associated Oncogenes in Osteosarcoma with THZ2, a Covalent CDK7 Inhibitor. Clin Cancer Res. 2020;26(11):2681-92.

19. Xing R, Zhou Y, Yu J, Yu Y, Nie Y, Luo W, et al. Whole-genome sequencing reveals novel tandem-duplication hotspots and a prognostic mutational signature in gastric cancer. Nat Commun. 2019;10(1):2037.

20. Zou CY, Wang J, Shen JN, Huang G, Jin S, Yin JQ, et al. Establishment and characteristics of two syngeneic human osteosarcoma cell lines from primary tumor and skip metastases. Acta Pharmacol Sin. 2008;29(3):325-32.

21. Han J, Yong B, Luo C, Tan P, Peng T, Shen J. High serum alkaline phosphatase cooperating with MMP-9 predicts metastasis and poor prognosis in patients with primary osteosarcoma in Southern China. World J Surg Oncol. 2012;10:37.

22. Szulc P, Seeman E, Delmas PD. Biochemical measurements of bone turnover in children and adolescents. Osteoporos Int. 2000;11(4):281-94.

23. Camp RL, Dolled-Filhart M, Rimm DL. X-tile: a new bio-informatics tool for biomarker assessment and outcome-based cut-point optimization. Clin Cancer Res. 2004;10(21):7252-9.

24. Zhang T, Kwiatkowski N, Olson CM, Dixon-Clarke SE, Abraham BJ, Greifenberg AK, et al. Covalent targeting of remote cysteine residues to develop CDK12 and CDK13 inhibitors. Nat Chem Biol. 2016;12(10):876-84.

25. Jiang YY, Lin DC, Mayakonda A, Hazawa M, Ding LW, Chien WW, et al. Targeting super-enhancer-associated oncogenes in oesophageal squamous cell carcinoma. Gut. 2017;66(8):1358-68.

26. Meyers PA, Schwartz CL, Krailo M, Kleinerman ES, Betcher D, Bernstein ML, et al. Osteosarcoma: a randomized, prospective trial of the addition of ifosfamide and/or muramyl tripeptide to cisplatin, doxorubicin, and high-dose methotrexate. J Clin Oncol. 2005;23(9):2004-11.

27. Aksnes LH, Bauer HC, Jebsen NL, Follerås G, Allert C, Haugen GS, et al. Limb-sparing surgery preserves more function than amputation: a Scandinavian sarcoma group study of 118 patients. J Bone Joint Surg Br. 2008;90(6):786-94.

28. Winkler K, Beron G, Kotz R, Salzer-Kuntschik M, Beck J, Beck W, et al. Neoadjuvant chemotherapy for osteogenic sarcoma: results of a Cooperative German/Austrian study. J Clin Oncol. 1984;2(6):617-24.

30. Bacci G, Ferrari S, Lari S, Mercuri M, Donati D, Longhi A, et al. Osteosarcoma of the limb. Amputation or limb salvage in patients treated by neoadjuvant chemotherapy. J Bone Joint Surg Br. 2002;84(1):88-92.

31. Zhao X, Wu Q, Gong X, Liu J, Ma Y. Osteosarcoma: a review of current and future therapeutic approaches. Biomed Eng Online. 2021;20(1):24.

32. Huang H, Hu J, Maryam A, Huang Q, Zhang Y, Ramakrishnan S, et al. Defining super-enhancer landscape in triple-negative breast cancer by multiomic profiling. Nat Commun. 2021;12(1):2242.

33. Wong M, Sun Y, Xi Z, Milazzo G, Poulos RC, Bartenhagen C, et al. JMJD6 is a tumorigenic factor and therapeutic target in neuroblastoma. Nat Commun. 2019;10(1):3319.

34. Zhang L, Xiong D, Liu Q, Luo Y, Tian Y, Xiao X, et al. Genome-Wide Histone H3K27 Acetylation Profiling Identified Genes Correlated With Prognosis in Papillary Thyroid Carcinoma. Front Cell Dev Biol. 2021;9:682561.

35. Fu Y, He G, Liu Z, Wang J, Zhang Z, Bao Q, et al. Exploration and Validation of a Novel Inflammatory Response-Associated Gene Signature to Predict Osteosarcoma Prognosis and Immune Infiltration. J Inflamm Res. 2021;14:6719-34.

36. Lei T, Qian H, Lei P, Hu Y. Ferroptosis-related gene signature associates with immunity and predicts prognosis accurately in patients with osteosarcoma. Cancer Sci. 2021;112(11):4785-98.

37. Ouyang Z, Li G, Zhu H, Wang J, Qi T, Qu Q, et al. Construction of a Five-Super-Enhancer-Associated-Genes Prognostic Model for Osteosarcoma Patients. Front Cell Dev Biol. 2020;8:598660.

38. Ferrari S, Bertoni F, Mercuri M, Picci P, Giacomini S, Longhi A, et al. Predictive factors of disease-free survival for non-metastatic osteosarcoma of the extremity: an analysis of 300 patients treated at the Rizzoli Institute. Ann Oncol. 2001;12(8):1145-50.

39. Bacci G, Longhi A, Ferrari S, Briccoli A, Donati D, De Paolis M, et al. Prognostic significance of serum lactate dehydrogenase in osteosarcoma of the extremity: experience at Rizzoli on 1421 patients treated over the last 30 years. Tumori. 2004;90(5):478-84.

40. Jia R, Li C, McCoy JP, Deng CX, Zheng ZM. SRp20 is a proto-oncogene critical for cell proliferation and tumor induction and maintenance. Int J Biol Sci. 2010;6(7):806-26.

1. Isakoff MS, Bielack SS, Meltzer P, Gorlick R. Osteosarcoma: Current Treatment and a Collaborative Pathway to Success. J Clin Oncol. 2015;33(27):3029-35.

2. Ferrari S, Smeland S, Mercuri M, Bertoni F, Longhi A, Ruggieri P, et al. Neoadjuvant chemotherapy with high-dose Ifosfamide, high-dose methotrexate, cisplatin, and doxorubicin for patients with localized osteosarcoma of the extremity: a joint study by the Italian and Scandinavian Sarcoma Groups. J Clin Oncol. 2005;23(34):8845-52.

3. Siegel RL, Miller KD, Jemal A. Cancer statistics, 2019. CA Cancer J Clin. 2019;69(1):7-34.

4. Ritter J, Bielack SS. Osteosarcoma. Ann Oncol. 2010;21 Suppl 7:vii320-5.

5. Gill J, Gorlick R. Advancing therapy for osteosarcoma. Nat Rev Clin Oncol. 2021;18(10):609-24.

6. Bielack SS, Kempf-Bielack B, Delling G, Exner GU, Flege S, Helmke K, et al. Prognostic factors in high-grade osteosarcoma of the extremities or trunk: an analysis of 1,702 patients treated on neoadjuvant cooperative osteosarcoma study group protocols. J Clin Oncol. 2002;20(3):776-90.

7. Han Z, Peng X, Yang Y, Yi J, Zhao D, Bao Q, et al. Integrated microfluidic-SERS for exosome biomarker profiling and osteosarcoma diagnosis. Biosens Bioelectron. 2022;217:114709.

8. Wu Y, Xu L, Yang P, Lin N, Huang X, Pan W, et al. Survival Prediction in High-grade Osteosarcoma Using Radiomics of Diagnostic Computed Tomography. EBioMedicine. 2018;34:27-34.

9. Chen W, Lin Y, Huang J, Yan Z, Cao H. A novel risk score model based on glycolysis-related genes and a prognostic model for predicting overall survival of osteosarcoma patients. J Orthop Res. 2022;40(10):2372-81.

10. Zhang M, Liu Y, Kong D. Identifying biomolecules and constructing a prognostic risk prediction model for recurrence in osteosarcoma. J Bone Oncol. 2021;26:100331.

11. Li L, Wang Y, He X, Li Z, Lu M, Gong T, et al. Hematological Prognostic Scoring System Can Predict Overall Survival and Can Indicate Response to Immunotherapy in Patients With Osteosarcoma. Front Immunol. 2022;13:879560.

12. Pelish HE, Liau BB, Nitulescu, II, Tangpeerachaikul A, Poss ZC, Da Silva DH, et al. Mediator kinase inhibition further activates super-enhancer-associated genes in AML. Nature. 2015;526(7572):273-6.

13. Whyte WA, Orlando DA, Hnisz D, Abraham BJ, Lin CY, Kagey MH, et al. Master transcription factors and mediator establish super-enhancers at key cell identity genes. Cell. 2013;153(2):307-19.

14. Tögel L, Nightingale R, Chueh AC, Jayachandran A, Tran H, Phesse T, et al. Dual Targeting of Bromodomain and Extraterminal Domain Proteins, and WNT or MAPK Signaling, Inhibits c-MYC Expression and Proliferation of Colorectal Cancer Cells. Mol Cancer Ther. 2016;15(6):1217-26.

15. Mansour MR, Abraham BJ, Anders L, Berezovskaya A, Gutierrez A, Durbin AD, et al. Oncogene regulation. An oncogenic super-enhancer formed through somatic mutation of a noncoding intergenic element. Science. 2014;346(6215):1373-7.

16. Lovén J, Hoke HA, Lin CY, Lau A, Orlando DA, Vakoc CR, et al. Selective inhibition of tumor oncogenes by disruption of super-enhancers. Cell. 2013;153(2):320-34.

17. Wang Y, Zhang T, Kwiatkowski N, Abraham BJ, Lee TI, Xie S, et al. CDK7-dependent transcriptional addiction in triple-negative breast cancer. Cell. 2015;163(1):174-86.

18. Zhang J, Liu W, Zou C, Zhao Z, Lai Y, Shi Z, et al. Targeting Super-Enhancer-Associated Oncogenes in Osteosarcoma with THZ2, a Covalent CDK7 Inhibitor. Clin Cancer Res. 2020;26(11):2681-92.

19. Xing R, Zhou Y, Yu J, Yu Y, Nie Y, Luo W, et al. Whole-genome sequencing reveals novel tandem-duplication hotspots and a prognostic mutational signature in gastric cancer. Nat Commun. 2019;10(1):2037.

20. Zou CY, Wang J, Shen JN, Huang G, Jin S, Yin JQ, et al. Establishment and characteristics of two syngeneic human osteosarcoma cell lines from primary tumor and skip metastases. Acta Pharmacol Sin. 2008;29(3):325-32.

21. Han J, Yong B, Luo C, Tan P, Peng T, Shen J. High serum alkaline phosphatase cooperating with MMP-9 predicts metastasis and poor prognosis in patients with primary osteosarcoma in Southern China. World J Surg Oncol. 2012;10:37.

22. Szulc P, Seeman E, Delmas PD. Biochemical measurements of bone turnover in children and adolescents. Osteoporos Int. 2000;11(4):281-94.

23. Camp RL, Dolled-Filhart M, Rimm DL. X-tile: a new bio-informatics tool for biomarker assessment and outcome-based cut-point optimization. Clin Cancer Res. 2004;10(21):7252-9.

24. Zhang T, Kwiatkowski N, Olson CM, Dixon-Clarke SE, Abraham BJ, Greifenberg AK, et al. Covalent targeting of remote cysteine residues to develop CDK12 and CDK13 inhibitors. Nat Chem Biol. 2016;12(10):876-84.

25. Jiang YY, Lin DC, Mayakonda A, Hazawa M, Ding LW, Chien WW, et al. Targeting super-enhancer-associated oncogenes in oesophageal squamous cell carcinoma. Gut. 2017;66(8):1358-68.

26. Meyers PA, Schwartz CL, Krailo M, Kleinerman ES, Betcher D, Bernstein ML, et al. Osteosarcoma: a randomized, prospective trial of the addition of ifosfamide and/or muramyl tripeptide to cisplatin, doxorubicin, and high-dose methotrexate. J Clin Oncol. 2005;23(9):2004-11.

27. Aksnes LH, Bauer HC, Jebsen NL, Follerås G, Allert C, Haugen GS, et al. Limb-sparing surgery preserves more function than amputation: a Scandinavian sarcoma group study of 118 patients. J Bone Joint Surg Br. 2008;90(6):786-94.

28. Winkler K, Beron G, Kotz R, Salzer-Kuntschik M, Beck J, Beck W, et al. Neoadjuvant chemotherapy for osteogenic sarcoma: results of a Cooperative German/Austrian study. J Clin Oncol. 1984;2(6):617-24.

29. Polites SF, Heaton TE, LaQuaglia MP, Kim ES, Barry WE, Goodhue CJ, et al. Pneumonectomy for Pediatric Tumors-a Pediatric Surgical Oncology Research Collaborative Study. Ann Surg. 2021;274(6):e605-e9.

30. Bacci G, Ferrari S, Lari S, Mercuri M, Donati D, Longhi A, et al. Osteosarcoma of the limb. Amputation or limb salvage in patients treated by neoadjuvant chemotherapy. J Bone Joint Surg Br. 2002;84(1):88-92.

31. Zhao X, Wu Q, Gong X, Liu J, Ma Y. Osteosarcoma: a review of current and future therapeutic approaches. Biomed Eng Online. 2021;20(1):24.

32. Huang H, Hu J, Maryam A, Huang Q, Zhang Y, Ramakrishnan S, et al. Defining super-enhancer landscape in triple-negative breast cancer by multiomic profiling. Nat Commun. 2021;12(1):2242.

33. Wong M, Sun Y, Xi Z, Milazzo G, Poulos RC, Bartenhagen C, et al. JMJD6 is a tumorigenic factor and therapeutic target in neuroblastoma. Nat Commun. 2019;10(1):3319.

34. Zhang L, Xiong D, Liu Q, Luo Y, Tian Y, Xiao X, et al. Genome-Wide Histone H3K27 Acetylation Profiling Identified Genes Correlated With Prognosis in Papillary Thyroid Carcinoma. Front Cell Dev Biol. 2021;9:682561.

35. Fu Y, He G, Liu Z, Wang J, Zhang Z, Bao Q, et al. Exploration and Validation of a Novel Inflammatory Response-Associated Gene Signature to Predict Osteosarcoma Prognosis and Immune Infiltration. J Inflamm Res. 2021;14:6719-34.

36. Lei T, Qian H, Lei P, Hu Y. Ferroptosis-related gene signature associates with immunity and predicts prognosis accurately in patients with osteosarcoma. Cancer Sci. 2021;112(11):4785-98.

37. Ouyang Z, Li G, Zhu H, Wang J, Qi T, Qu Q, et al. Construction of a Five-Super-Enhancer-Associated-Genes Prognostic Model for Osteosarcoma Patients. Front Cell Dev Biol. 2020;8:598660.

38. Ferrari S, Bertoni F, Mercuri M, Picci P, Giacomini S, Longhi A, et al. Predictive factors of disease-free survival for non-metastatic osteosarcoma of the extremity: an analysis of 300 patients treated at the Rizzoli Institute. Ann Oncol. 2001;12(8):1145-50.

39. Bacci G, Longhi A, Ferrari S, Briccoli A, Donati D, De Paolis M, et al. Prognostic significance of serum lactate dehydrogenase in osteosarcoma of the extremity: experience at Rizzoli on 1421 patients treated over the last 30 years. Tumori. 2004;90(5):478-84.

40. Jia R, Li C, McCoy JP, Deng CX, Zheng ZM. SRp20 is a proto-oncogene critical for cell proliferation and tumor induction and maintenance. Int J Biol Sci. 2010;6(7):806-26.

41. Xue Y, Guo Y, Liu N, Meng X. MicroRNA-22-3p targeted regulating transcription factor 7-like 2 (TCF7L2) constrains the Wnt/β-catenin pathway and malignant behavior in osteosarcoma. Bioengineered. 2022;13(4):9135-47.

1. Isakoff MS, Bielack SS, Meltzer P, Gorlick R. Osteosarcoma: Current Treatment and a Collaborative Pathway to Success. J Clin Oncol. 2015;33(27):3029-35.

2. Ferrari S, Smeland S, Mercuri M, Bertoni F, Longhi A, Ruggieri P, et al. Neoadjuvant chemotherapy with high-dose Ifosfamide, high-dose methotrexate, cisplatin, and doxorubicin for patients with localized osteosarcoma of the extremity: a joint study by the Italian and Scandinavian Sarcoma Groups. J Clin Oncol. 2005;23(34):8845-52.

3. Siegel RL, Miller KD, Jemal A. Cancer statistics, 2019. CA Cancer J Clin. 2019;69(1):7-34.

4. Ritter J, Bielack SS. Osteosarcoma. Ann Oncol. 2010;21 Suppl 7:vii320-5.

5. Gill J, Gorlick R. Advancing therapy for osteosarcoma. Nat Rev Clin Oncol. 2021;18(10):609-24.

6. Bielack SS, Kempf-Bielack B, Delling G, Exner GU, Flege S, Helmke K, et al. Prognostic factors in high-grade osteosarcoma of the extremities or trunk: an analysis of 1,702 patients treated on neoadjuvant cooperative osteosarcoma study group protocols. J Clin Oncol. 2002;20(3):776-90.

7. Han Z, Peng X, Yang Y, Yi J, Zhao D, Bao Q, et al. Integrated microfluidic-SERS for exosome biomarker profiling and osteosarcoma diagnosis. Biosens Bioelectron. 2022;217:114709.

8. Wu Y, Xu L, Yang P, Lin N, Huang X, Pan W, et al. Survival Prediction in High-grade Osteosarcoma Using Radiomics of Diagnostic Computed Tomography. EBioMedicine. 2018;34:27-34.

9. Chen W, Lin Y, Huang J, Yan Z, Cao H. A novel risk score model based on glycolysis-related genes and a prognostic model for predicting overall survival of osteosarcoma patients. J Orthop Res. 2022;40(10):2372-81.

10. Zhang M, Liu Y, Kong D. Identifying biomolecules and constructing a prognostic risk prediction model for recurrence in osteosarcoma. J Bone Oncol. 2021;26:100331.

11. Li L, Wang Y, He X, Li Z, Lu M, Gong T, et al. Hematological Prognostic Scoring System Can Predict Overall Survival and Can Indicate Response to Immunotherapy in Patients With Osteosarcoma. Front Immunol. 2022;13:879560.

12. Pelish HE, Liau BB, Nitulescu, II, Tangpeerachaikul A, Poss ZC, Da Silva DH, et al. Mediator kinase inhibition further activates super-enhancer-associated genes in AML. Nature. 2015;526(7572):273-6.

13. Whyte WA, Orlando DA, Hnisz D, Abraham BJ, Lin CY, Kagey MH, et al. Master transcription factors and mediator establish super-enhancers at key cell identity genes. Cell. 2013;153(2):307-19.

14. Tögel L, Nightingale R, Chueh AC, Jayachandran A, Tran H, Phesse T, et al. Dual Targeting of Bromodomain and Extraterminal Domain Proteins, and WNT or MAPK Signaling, Inhibits c-MYC Expression and Proliferation of Colorectal Cancer Cells. Mol Cancer Ther. 2016;15(6):1217-26.

16. Lovén J, Hoke HA, Lin CY, Lau A, Orlando DA, Vakoc CR, et al. Selective inhibition of tumor oncogenes by disruption of super-enhancers. Cell. 2013;153(2):320-34.

17. Wang Y, Zhang T, Kwiatkowski N, Abraham BJ, Lee TI, Xie S, et al. CDK7-dependent transcriptional addiction in triple-negative breast cancer. Cell. 2015;163(1):174-86.

18. Zhang J, Liu W, Zou C, Zhao Z, Lai Y, Shi Z, et al. Targeting Super-Enhancer-Associated Oncogenes in Osteosarcoma with THZ2, a Covalent CDK7 Inhibitor. Clin Cancer Res. 2020;26(11):2681-92.

19. Xing R, Zhou Y, Yu J, Yu Y, Nie Y, Luo W, et al. Whole-genome sequencing reveals novel tandem-duplication hotspots and a prognostic mutational signature in gastric cancer. Nat Commun. 2019;10(1):2037.

20. Zou CY, Wang J, Shen JN, Huang G, Jin S, Yin JQ, et al. Establishment and characteristics of two syngeneic human osteosarcoma cell lines from primary tumor and skip metastases. Acta Pharmacol Sin. 2008;29(3):325-32.

21. Han J, Yong B, Luo C, Tan P, Peng T, Shen J. High serum alkaline phosphatase cooperating with MMP-9 predicts metastasis and poor prognosis in patients with primary osteosarcoma in Southern China. World J Surg Oncol. 2012;10:37.

22. Szulc P, Seeman E, Delmas PD. Biochemical measurements of bone turnover in children and adolescents. Osteoporos Int. 2000;11(4):281-94.

23. Camp RL, Dolled-Filhart M, Rimm DL. X-tile: a new bio-informatics tool for biomarker assessment and outcome-based cut-point optimization. Clin Cancer Res. 2004;10(21):7252-9.

24. Zhang T, Kwiatkowski N, Olson CM, Dixon-Clarke SE, Abraham BJ, Greifenberg AK, et al. Covalent targeting of remote cysteine residues to develop CDK12 and CDK13 inhibitors. Nat Chem Biol. 2016;12(10):876-84.

25. Jiang YY, Lin DC, Mayakonda A, Hazawa M, Ding LW, Chien WW, et al. Targeting super-enhancer-associated oncogenes in oesophageal squamous cell carcinoma. Gut. 2017;66(8):1358-68.

26. Meyers PA, Schwartz CL, Krailo M, Kleinerman ES, Betcher D, Bernstein ML, et al. Osteosarcoma: a randomized, prospective trial of the addition of ifosfamide and/or muramyl tripeptide to cisplatin, doxorubicin, and high-dose methotrexate. J Clin Oncol. 2005;23(9):2004-11.

27. Aksnes LH, Bauer HC, Jebsen NL, Follerås G, Allert C, Haugen GS, et al. Limb-sparing surgery preserves more function than amputation: a Scandinavian sarcoma group study of 118 patients. J Bone Joint Surg Br. 2008;90(6):786-94.

28. Winkler K, Beron G, Kotz R, Salzer-Kuntschik M, Beck J, Beck W, et al. Neoadjuvant chemotherapy for osteogenic sarcoma: results of a Cooperative German/Austrian study. J Clin Oncol. 1984;2(6):617-24.

29. Polites SF, Heaton TE, LaQuaglia MP, Kim ES, Barry WE, Goodhue CJ, et al. Pneumonectomy for Pediatric Tumors-a Pediatric Surgical Oncology Research Collaborative Study. Ann Surg. 2021;274(6):e605-e9.

30. Bacci G, Ferrari S, Lari S, Mercuri M, Donati D, Longhi A, et al. Osteosarcoma of the limb. Amputation or limb salvage in patients treated by neoadjuvant chemotherapy. J Bone Joint Surg Br. 2002;84(1):88-92.

31. Zhao X, Wu Q, Gong X, Liu J, Ma Y. Osteosarcoma: a review of current and future therapeutic approaches. Biomed Eng Online. 2021;20(1):24.

32. Huang H, Hu J, Maryam A, Huang Q, Zhang Y, Ramakrishnan S, et al. Defining super-enhancer landscape in triple-negative breast cancer by multiomic profiling. Nat Commun. 2021;12(1):2242.

33. Wong M, Sun Y, Xi Z, Milazzo G, Poulos RC, Bartenhagen C, et al. JMJD6 is a tumorigenic factor and therapeutic target in neuroblastoma. Nat Commun. 2019;10(1):3319.

34. Zhang L, Xiong D, Liu Q, Luo Y, Tian Y, Xiao X, et al. Genome-Wide Histone H3K27 Acetylation Profiling Identified Genes Correlated With Prognosis in Papillary Thyroid Carcinoma. Front Cell Dev Biol. 2021;9:682561.

35. Fu Y, He G, Liu Z, Wang J, Zhang Z, Bao Q, et al. Exploration and Validation of a Novel Inflammatory Response-Associated Gene Signature to Predict Osteosarcoma Prognosis and Immune Infiltration. J Inflamm Res. 2021;14:6719-34.

36. Lei T, Qian H, Lei P, Hu Y. Ferroptosis-related gene signature associates with immunity and predicts prognosis accurately in patients with osteosarcoma. Cancer Sci. 2021;112(11):4785-98.

37. Ouyang Z, Li G, Zhu H, Wang J, Qi T, Qu Q, et al. Construction of a Five-Super-Enhancer-Associated-Genes Prognostic Model for Osteosarcoma Patients. Front Cell Dev Biol. 2020;8:598660.

38. Ferrari S, Bertoni F, Mercuri M, Picci P, Giacomini S, Longhi A, et al. Predictive factors of disease-free survival for non-metastatic osteosarcoma of the extremity: an analysis of 300 patients treated at the Rizzoli Institute. Ann Oncol. 2001;12(8):1145-50.

39. Bacci G, Longhi A, Ferrari S, Briccoli A, Donati D, De Paolis M, et al. Prognostic significance of serum lactate dehydrogenase in osteosarcoma of the extremity: experience at Rizzoli on 1421 patients treated over the last 30 years. Tumori. 2004;90(5):478-84.

40. Jia R, Li C, McCoy JP, Deng CX, Zheng ZM. SRp20 is a proto-oncogene critical for cell proliferation and tumor induction and maintenance. Int J Biol Sci. 2010;6(7):806-26.

41. Xue Y, Guo Y, Liu N, Meng X. MicroRNA-22-3p targeted regulating transcription factor 7-like 2 (TCF7L2) constrains the Wnt/β-catenin pathway and malignant behavior in osteosarcoma. Bioengineered. 2022;13(4):9135-47.

42. Li H, Han X, Yang S, Wang Y, Dong Y, Tang T. FOXP1 drives osteosarcoma development by repressing P21 and RB transcription downstream of P53. Oncogene. 2021;40(15):2785-802.

1. Isakoff MS, Bielack SS, Meltzer P, Gorlick R. Osteosarcoma: Current Treatment and a Collaborative Pathway to Success. J Clin Oncol. 2015;33(27):3029-35.

2. Ferrari S, Smeland S, Mercuri M, Bertoni F, Longhi A, Ruggieri P, et al. Neoadjuvant chemotherapy with high-dose Ifosfamide, high-dose methotrexate, cisplatin, and doxorubicin for patients with localized osteosarcoma of the extremity: a joint study by the Italian and Scandinavian Sarcoma Groups. J Clin Oncol. 2005;23(34):8845-52.

3. Siegel RL, Miller KD, Jemal A. Cancer statistics, 2019. CA Cancer J Clin. 2019;69(1):7-34.

4. Ritter J, Bielack SS. Osteosarcoma. Ann Oncol. 2010;21 Suppl 7:vii320-5.

6. Bielack SS, Kempf-Bielack B, Delling G, Exner GU, Flege S, Helmke K, et al. Prognostic factors in high-grade osteosarcoma of the extremities or trunk: an analysis of 1,702 patients treated on neoadjuvant cooperative osteosarcoma study group protocols. J Clin Oncol. 2002;20(3):776-90.

7. Han Z, Peng X, Yang Y, Yi J, Zhao D, Bao Q, et al. Integrated microfluidic-SERS for exosome biomarker profiling and osteosarcoma diagnosis. Biosens Bioelectron. 2022;217:114709.

8. Wu Y, Xu L, Yang P, Lin N, Huang X, Pan W, et al. Survival Prediction in High-grade Osteosarcoma Using Radiomics of Diagnostic Computed Tomography. EBioMedicine. 2018;34:27-34.

9. Chen W, Lin Y, Huang J, Yan Z, Cao H. A novel risk score model based on glycolysis-related genes and a prognostic model for predicting overall survival of osteosarcoma patients. J Orthop Res. 2022;40(10):2372-81.

10. Zhang M, Liu Y, Kong D. Identifying biomolecules and constructing a prognostic risk prediction model for recurrence in osteosarcoma. J Bone Oncol. 2021;26:100331.

11. Li L, Wang Y, He X, Li Z, Lu M, Gong T, et al. Hematological Prognostic Scoring System Can Predict Overall Survival and Can Indicate Response to Immunotherapy in Patients With Osteosarcoma. Front Immunol. 2022;13:879560.

12. Pelish HE, Liau BB, Nitulescu, II, Tangpeerachaikul A, Poss ZC, Da Silva DH, et al. Mediator kinase inhibition further activates super-enhancer-associated genes in AML. Nature. 2015;526(7572):273-6.

13. Whyte WA, Orlando DA, Hnisz D, Abraham BJ, Lin CY, Kagey MH, et al. Master transcription factors and mediator establish super-enhancers at key cell identity genes. Cell. 2013;153(2):307-19.

14. Tögel L, Nightingale R, Chueh AC, Jayachandran A, Tran H, Phesse T, et al. Dual Targeting of Bromodomain and Extraterminal Domain Proteins, and WNT or MAPK Signaling, Inhibits c-MYC Expression and Proliferation of Colorectal Cancer Cells. Mol Cancer Ther. 2016;15(6):1217-26.

15. Mansour MR, Abraham BJ, Anders L, Berezovskaya A, Gutierrez A, Durbin AD, et al. Oncogene regulation. An oncogenic super-enhancer formed through somatic mutation of a noncoding intergenic element. Science. 2014;346(6215):1373-7.

16. Lovén J, Hoke HA, Lin CY, Lau A, Orlando DA, Vakoc CR, et al. Selective inhibition of tumor oncogenes by disruption of super-enhancers. Cell. 2013;153(2):320-34.

17. Wang Y, Zhang T, Kwiatkowski N, Abraham BJ, Lee TI, Xie S, et al. CDK7-dependent transcriptional addiction in triple-negative breast cancer. Cell. 2015;163(1):174-86.

18. Zhang J, Liu W, Zou C, Zhao Z, Lai Y, Shi Z, et al. Targeting Super-Enhancer-Associated Oncogenes in Osteosarcoma with THZ2, a Covalent CDK7 Inhibitor. Clin Cancer Res. 2020;26(11):2681-92.

19. Xing R, Zhou Y, Yu J, Yu Y, Nie Y, Luo W, et al. Whole-genome sequencing reveals novel tandem-duplication hotspots and a prognostic mutational signature in gastric cancer. Nat Commun. 2019;10(1):2037.

20. Zou CY, Wang J, Shen JN, Huang G, Jin S, Yin JQ, et al. Establishment and characteristics of two syngeneic human osteosarcoma cell lines from primary tumor and skip metastases. Acta Pharmacol Sin. 2008;29(3):325-32.

21. Han J, Yong B, Luo C, Tan P, Peng T, Shen J. High serum alkaline phosphatase cooperating with MMP-9 predicts metastasis and poor prognosis in patients with primary osteosarcoma in Southern China. World J Surg Oncol. 2012;10:37.

22. Szulc P, Seeman E, Delmas PD. Biochemical measurements of bone turnover in children and adolescents. Osteoporos Int. 2000;11(4):281-94.

23. Camp RL, Dolled-Filhart M, Rimm DL. X-tile: a new bio-informatics tool for biomarker assessment and outcome-based cut-point optimization. Clin Cancer Res. 2004;10(21):7252-9.

24. Zhang T, Kwiatkowski N, Olson CM, Dixon-Clarke SE, Abraham BJ, Greifenberg AK, et al. Covalent targeting of remote cysteine residues to develop CDK12 and CDK13 inhibitors. Nat Chem Biol. 2016;12(10):876-84.

25. Jiang YY, Lin DC, Mayakonda A, Hazawa M, Ding LW, Chien WW, et al. Targeting super-enhancer-associated oncogenes in oesophageal squamous cell carcinoma. Gut. 2017;66(8):1358-68.

26. Meyers PA, Schwartz CL, Krailo M, Kleinerman ES, Betcher D, Bernstein ML, et al. Osteosarcoma: a randomized, prospective trial of the addition of ifosfamide and/or muramyl tripeptide to cisplatin, doxorubicin, and high-dose methotrexate. J Clin Oncol. 2005;23(9):2004-11.

27. Aksnes LH, Bauer HC, Jebsen NL, Follerås G, Allert C, Haugen GS, et al. Limb-sparing surgery preserves more function than amputation: a Scandinavian sarcoma group study of 118 patients. J Bone Joint Surg Br. 2008;90(6):786-94.

28. Winkler K, Beron G, Kotz R, Salzer-Kuntschik M, Beck J, Beck W, et al. Neoadjuvant chemotherapy for osteogenic sarcoma: results of a Cooperative German/Austrian study. J Clin Oncol. 1984;2(6):617-24.

29. Polites SF, Heaton TE, LaQuaglia MP, Kim ES, Barry WE, Goodhue CJ, et al. Pneumonectomy for Pediatric Tumors-a Pediatric Surgical Oncology Research Collaborative Study. Ann Surg. 2021;274(6):e605-e9.

30. Bacci G, Ferrari S, Lari S, Mercuri M, Donati D, Longhi A, et al. Osteosarcoma of the limb. Amputation or limb salvage in patients treated by neoadjuvant chemotherapy. J Bone Joint Surg Br. 2002;84(1):88-92.

31. Zhao X, Wu Q, Gong X, Liu J, Ma Y. Osteosarcoma: a review of current and future therapeutic approaches. Biomed Eng Online. 2021;20(1):24.

32. Huang H, Hu J, Maryam A, Huang Q, Zhang Y, Ramakrishnan S, et al. Defining super-enhancer landscape in triple-negative breast cancer by multiomic profiling. Nat Commun. 2021;12(1):2242.

33. Wong M, Sun Y, Xi Z, Milazzo G, Poulos RC, Bartenhagen C, et al. JMJD6 is a tumorigenic factor and therapeutic target in neuroblastoma. Nat Commun. 2019;10(1):3319.

34. Zhang L, Xiong D, Liu Q, Luo Y, Tian Y, Xiao X, et al. Genome-Wide Histone H3K27 Acetylation Profiling Identified Genes Correlated With Prognosis in Papillary Thyroid Carcinoma. Front Cell Dev Biol. 2021;9:682561.

35. Fu Y, He G, Liu Z, Wang J, Zhang Z, Bao Q, et al. Exploration and Validation of a Novel Inflammatory Response-Associated Gene Signature to Predict Osteosarcoma Prognosis and Immune Infiltration. J Inflamm Res. 2021;14:6719-34.

36. Lei T, Qian H, Lei P, Hu Y. Ferroptosis-related gene signature associates with immunity and predicts prognosis accurately in patients with osteosarcoma. Cancer Sci. 2021;112(11):4785-98.

37. Ouyang Z, Li G, Zhu H, Wang J, Qi T, Qu Q, et al. Construction of a Five-Super-Enhancer-Associated-Genes Prognostic Model for Osteosarcoma Patients. Front Cell Dev Biol. 2020;8:598660.

38. Ferrari S, Bertoni F, Mercuri M, Picci P, Giacomini S, Longhi A, et al. Predictive factors of disease-free survival for non-metastatic osteosarcoma of the extremity: an analysis of 300 patients treated at the Rizzoli Institute. Ann Oncol. 2001;12(8):1145-50.

39. Bacci G, Longhi A, Ferrari S, Briccoli A, Donati D, De Paolis M, et al. Prognostic significance of serum lactate dehydrogenase in osteosarcoma of the extremity: experience at Rizzoli on 1421 patients treated over the last 30 years. Tumori. 2004;90(5):478-84.

40. Jia R, Li C, McCoy JP, Deng CX, Zheng ZM. SRp20 is a proto-oncogene critical for cell proliferation and tumor induction and maintenance. Int J Biol Sci. 2010;6(7):806-26.

41. Xue Y, Guo Y, Liu N, Meng X. MicroRNA-22-3p targeted regulating transcription factor 7-like 2 (TCF7L2) constrains the Wnt/β-catenin pathway and malignant behavior in osteosarcoma. Bioengineered. 2022;13(4):9135-47.

42. Li H, Han X, Yang S, Wang Y, Dong Y, Tang T. FOXP1 drives osteosarcoma development by repressing P21 and RB transcription downstream of P53. Oncogene. 2021;40(15):2785-802.

43. Xu L, Xia C, Sheng F, Sun Q, Xiong J, Wang S. CEP55 promotes the proliferation and invasion of tumour cells via the AKT signalling pathway in osteosarcoma. Carcinogenesis. 2018;39(4):623-31.

1. Isakoff MS, Bielack SS, Meltzer P, Gorlick R. Osteosarcoma: Current Treatment and a Collaborative Pathway to Success. J Clin Oncol. 2015;33(27):3029-35.

2. Ferrari S, Smeland S, Mercuri M, Bertoni F, Longhi A, Ruggieri P, et al. Neoadjuvant chemotherapy with high-dose Ifosfamide, high-dose methotrexate, cisplatin, and doxorubicin for patients with localized osteosarcoma of the extremity: a joint study by the Italian and Scandinavian Sarcoma Groups. J Clin Oncol. 2005;23(34):8845-52.

3. Siegel RL, Miller KD, Jemal A. Cancer statistics, 2019. CA Cancer J Clin. 2019;69(1):7-34.

4. Ritter J, Bielack SS. Osteosarcoma. Ann Oncol. 2010;21 Suppl 7:vii320-5.

5. Gill J, Gorlick R. Advancing therapy for osteosarcoma. Nat Rev Clin Oncol. 2021;18(10):609-24.

6. Bielack SS, Kempf-Bielack B, Delling G, Exner GU, Flege S, Helmke K, et al. Prognostic factors in high-grade osteosarcoma of the extremities or trunk: an analysis of 1,702 patients treated on neoadjuvant cooperative osteosarcoma study group protocols. J Clin Oncol. 2002;20(3):776-90.

7. Han Z, Peng X, Yang Y, Yi J, Zhao D, Bao Q, et al. Integrated microfluidic-SERS for exosome biomarker profiling and osteosarcoma diagnosis. Biosens Bioelectron. 2022;217:114709.

8. Wu Y, Xu L, Yang P, Lin N, Huang X, Pan W, et al. Survival Prediction in High-grade Osteosarcoma Using Radiomics of Diagnostic Computed Tomography. EBioMedicine. 2018;34:27-34.

9. Chen W, Lin Y, Huang J, Yan Z, Cao H. A novel risk score model based on glycolysis-related genes and a prognostic model for predicting overall survival of osteosarcoma patients. J Orthop Res. 2022;40(10):2372-81.

10. Zhang M, Liu Y, Kong D. Identifying biomolecules and constructing a prognostic risk prediction model for recurrence in osteosarcoma. J Bone Oncol. 2021;26:100331.

11. Li L, Wang Y, He X, Li Z, Lu M, Gong T, et al. Hematological Prognostic Scoring System Can Predict Overall Survival and Can Indicate Response to Immunotherapy in Patients With Osteosarcoma. Front Immunol. 2022;13:879560.

12. Pelish HE, Liau BB, Nitulescu, II, Tangpeerachaikul A, Poss ZC, Da Silva DH, et al. Mediator kinase inhibition further activates super-enhancer-associated genes in AML. Nature. 2015;526(7572):273-6.

13. Whyte WA, Orlando DA, Hnisz D, Abraham BJ, Lin CY, Kagey MH, et al. Master transcription factors and mediator establish super-enhancers at key cell identity genes. Cell. 2013;153(2):307-19.

14. Tögel L, Nightingale R, Chueh AC, Jayachandran A, Tran H, Phesse T, et al. Dual Targeting of Bromodomain and Extraterminal Domain Proteins, and WNT or MAPK Signaling, Inhibits c-MYC Expression and Proliferation of Colorectal Cancer Cells. Mol Cancer Ther. 2016;15(6):1217-26.

15. Mansour MR, Abraham BJ, Anders L, Berezovskaya A, Gutierrez A, Durbin AD, et al. Oncogene regulation. An oncogenic super-enhancer formed through somatic mutation of a noncoding intergenic element. Science. 2014;346(6215):1373-7.

16. Lovén J, Hoke HA, Lin CY, Lau A, Orlando DA, Vakoc CR, et al. Selective inhibition of tumor oncogenes by disruption of super-enhancers. Cell. 2013;153(2):320-34.

17. Wang Y, Zhang T, Kwiatkowski N, Abraham BJ, Lee TI, Xie S, et al. CDK7-dependent transcriptional addiction in triple-negative breast cancer. Cell. 2015;163(1):174-86.

18. Zhang J, Liu W, Zou C, Zhao Z, Lai Y, Shi Z, et al. Targeting Super-Enhancer-Associated Oncogenes in Osteosarcoma with THZ2, a Covalent CDK7 Inhibitor. Clin Cancer Res. 2020;26(11):2681-92.

19. Xing R, Zhou Y, Yu J, Yu Y, Nie Y, Luo W, et al. Whole-genome sequencing reveals novel tandem-duplication hotspots and a prognostic mutational signature in gastric cancer. Nat Commun. 2019;10(1):2037.

20. Zou CY, Wang J, Shen JN, Huang G, Jin S, Yin JQ, et al. Establishment and characteristics of two syngeneic human osteosarcoma cell lines from primary tumor and skip metastases. Acta Pharmacol Sin. 2008;29(3):325-32.

21. Han J, Yong B, Luo C, Tan P, Peng T, Shen J. High serum alkaline phosphatase cooperating with MMP-9 predicts metastasis and poor prognosis in patients with primary osteosarcoma in Southern China. World J Surg Oncol. 2012;10:37.

22. Szulc P, Seeman E, Delmas PD. Biochemical measurements of bone turnover in children and adolescents. Osteoporos Int. 2000;11(4):281-94.

23. Camp RL, Dolled-Filhart M, Rimm DL. X-tile: a new bio-informatics tool for biomarker assessment and outcome-based cut-point optimization. Clin Cancer Res. 2004;10(21):7252-9.

24. Zhang T, Kwiatkowski N, Olson CM, Dixon-Clarke SE, Abraham BJ, Greifenberg AK, et al. Covalent targeting of remote cysteine residues to develop CDK12 and CDK13 inhibitors. Nat Chem Biol. 2016;12(10):876-84.

25. Jiang YY, Lin DC, Mayakonda A, Hazawa M, Ding LW, Chien WW, et al. Targeting super-enhancer-associated oncogenes in oesophageal squamous cell carcinoma. Gut. 2017;66(8):1358-68.

26. Meyers PA, Schwartz CL, Krailo M, Kleinerman ES, Betcher D, Bernstein ML, et al. Osteosarcoma: a randomized, prospective trial of the addition of ifosfamide and/or muramyl tripeptide to cisplatin, doxorubicin, and high-dose methotrexate. J Clin Oncol. 2005;23(9):2004-11.

27. Aksnes LH, Bauer HC, Jebsen NL, Follerås G, Allert C, Haugen GS, et al. Limb-sparing surgery preserves more function than amputation: a Scandinavian sarcoma group study of 118 patients. J Bone Joint Surg Br. 2008;90(6):786-94.

28. Winkler K, Beron G, Kotz R, Salzer-Kuntschik M, Beck J, Beck W, et al. Neoadjuvant chemotherapy for osteogenic sarcoma: results of a Cooperative German/Austrian study. J Clin Oncol. 1984;2(6):617-24.

29. Polites SF, Heaton TE, LaQuaglia MP, Kim ES, Barry WE, Goodhue CJ, et al. Pneumonectomy for Pediatric Tumors-a Pediatric Surgical Oncology Research Collaborative Study. Ann Surg. 2021;274(6):e605-e9.

30. Bacci G, Ferrari S, Lari S, Mercuri M, Donati D, Longhi A, et al. Osteosarcoma of the limb. Amputation or limb salvage in patients treated by neoadjuvant chemotherapy. J Bone Joint Surg Br. 2002;84(1):88-92.

31. Zhao X, Wu Q, Gong X, Liu J, Ma Y. Osteosarcoma: a review of current and future therapeutic approaches. Biomed Eng Online. 2021;20(1):24.

32. Huang H, Hu J, Maryam A, Huang Q, Zhang Y, Ramakrishnan S, et al. Defining super-enhancer landscape in triple-negative breast cancer by multiomic profiling. Nat Commun. 2021;12(1):2242.

33. Wong M, Sun Y, Xi Z, Milazzo G, Poulos RC, Bartenhagen C, et al. JMJD6 is a tumorigenic factor and therapeutic target in neuroblastoma. Nat Commun. 2019;10(1):3319.

34. Zhang L, Xiong D, Liu Q, Luo Y, Tian Y, Xiao X, et al. Genome-Wide Histone H3K27 Acetylation Profiling Identified Genes Correlated With Prognosis in Papillary Thyroid Carcinoma. Front Cell Dev Biol. 2021;9:682561.

35. Fu Y, He G, Liu Z, Wang J, Zhang Z, Bao Q, et al. Exploration and Validation of a Novel Inflammatory Response-Associated Gene Signature to Predict Osteosarcoma Prognosis and Immune Infiltration. J Inflamm Res. 2021;14:6719-34.

36. Lei T, Qian H, Lei P, Hu Y. Ferroptosis-related gene signature associates with immunity and predicts prognosis accurately in patients with osteosarcoma. Cancer Sci. 2021;112(11):4785-98.

37. Ouyang Z, Li G, Zhu H, Wang J, Qi T, Qu Q, et al. Construction of a Five-Super-Enhancer-Associated-Genes Prognostic Model for Osteosarcoma Patients. Front Cell Dev Biol. 2020;8:598660.

38. Ferrari S, Bertoni F, Mercuri M, Picci P, Giacomini S, Longhi A, et al. Predictive factors of disease-free survival for non-metastatic osteosarcoma of the extremity: an analysis of 300 patients treated at the Rizzoli Institute. Ann Oncol. 2001;12(8):1145-50.

39. Bacci G, Longhi A, Ferrari S, Briccoli A, Donati D, De Paolis M, et al. Prognostic significance of serum lactate dehydrogenase in osteosarcoma of the extremity: experience at Rizzoli on 1421 patients treated over the last 30 years. Tumori. 2004;90(5):478-84.

40. Jia R, Li C, McCoy JP, Deng CX, Zheng ZM. SRp20 is a proto-oncogene critical for cell proliferation and tumor induction and maintenance. Int J Biol Sci. 2010;6(7):806-26.

41. Xue Y, Guo Y, Liu N, Meng X. MicroRNA-22-3p targeted regulating transcription factor 7-like 2 (TCF7L2) constrains the Wnt/β-catenin pathway and malignant behavior in osteosarcoma. Bioengineered. 2022;13(4):9135-47.

42. Li H, Han X, Yang S, Wang Y, Dong Y, Tang T. FOXP1 drives osteosarcoma development by repressing P21 and RB transcription downstream of P53. Oncogene. 2021;40(15):2785-802.

43. Xu L, Xia C, Sheng F, Sun Q, Xiong J, Wang S. CEP55 promotes the proliferation and invasion of tumour cells via the AKT signalling pathway in osteosarcoma. Carcinogenesis. 2018;39(4):623-31.

44. Regard JB, Scheek S, Borbiev T, Lanahan AA, Schneider A, Demetriades AM, et al. Verge: a novel vascular early response gene. J Neurosci. 2004;24(16):4092-103.

1. Isakoff MS, Bielack SS, Meltzer P, Gorlick R. Osteosarcoma: Current Treatment and a Collaborative Pathway to Success. J Clin Oncol. 2015;33(27):3029-35.

2. Ferrari S, Smeland S, Mercuri M, Bertoni F, Longhi A, Ruggieri P, et al. Neoadjuvant chemotherapy with high-dose Ifosfamide, high-dose methotrexate, cisplatin, and doxorubicin for patients with localized osteosarcoma of the extremity: a joint study by the Italian and Scandinavian Sarcoma Groups. J Clin Oncol. 2005;23(34):8845-52.

3. Siegel RL, Miller KD, Jemal A. Cancer statistics, 2019. CA Cancer J Clin. 2019;69(1):7-34.

4. Ritter J, Bielack SS. Osteosarcoma. Ann Oncol. 2010;21 Suppl 7:vii320-5.

5. Gill J, Gorlick R. Advancing therapy for osteosarcoma. Nat Rev Clin Oncol. 2021;18(10):609-24.

6. Bielack SS, Kempf-Bielack B, Delling G, Exner GU, Flege S, Helmke K, et al. Prognostic factors in high-grade osteosarcoma of the extremities or trunk: an analysis of 1,702 patients treated on neoadjuvant cooperative osteosarcoma study group protocols. J Clin Oncol. 2002;20(3):776-90.

7. Han Z, Peng X, Yang Y, Yi J, Zhao D, Bao Q, et al. Integrated microfluidic-SERS for exosome biomarker profiling and osteosarcoma diagnosis. Biosens Bioelectron. 2022;217:114709.

8. Wu Y, Xu L, Yang P, Lin N, Huang X, Pan W, et al. Survival Prediction in High-grade Osteosarcoma Using Radiomics of Diagnostic Computed Tomography. EBioMedicine. 2018;34:27-34.

9. Chen W, Lin Y, Huang J, Yan Z, Cao H. A novel risk score model based on glycolysis-related genes and a prognostic model for predicting overall survival of osteosarcoma patients. J Orthop Res. 2022;40(10):2372-81.

10. Zhang M, Liu Y, Kong D. Identifying biomolecules and constructing a prognostic risk prediction model for recurrence in osteosarcoma. J Bone Oncol. 2021;26:100331.

11. Li L, Wang Y, He X, Li Z, Lu M, Gong T, et al. Hematological Prognostic Scoring System Can Predict Overall Survival and Can Indicate Response to Immunotherapy in Patients With Osteosarcoma. Front Immunol. 2022;13:879560.

12. Pelish HE, Liau BB, Nitulescu, II, Tangpeerachaikul A, Poss ZC, Da Silva DH, et al. Mediator kinase inhibition further activates super-enhancer-associated genes in AML. Nature. 2015;526(7572):273-6.

13. Whyte WA, Orlando DA, Hnisz D, Abraham BJ, Lin CY, Kagey MH, et al. Master transcription factors and mediator establish super-enhancers at key cell identity genes. Cell. 2013;153(2):307-19.

14. Tögel L, Nightingale R, Chueh AC, Jayachandran A, Tran H, Phesse T, et al. Dual Targeting of Bromodomain and Extraterminal Domain Proteins, and WNT or MAPK Signaling, Inhibits c-MYC Expression and Proliferation of Colorectal Cancer Cells. Mol Cancer Ther. 2016;15(6):1217-26.

15. Mansour MR, Abraham BJ, Anders L, Berezovskaya A, Gutierrez A, Durbin AD, et al. Oncogene regulation. An oncogenic super-enhancer formed through somatic mutation of a noncoding intergenic element. Science. 2014;346(6215):1373-7.

16. Lovén J, Hoke HA, Lin CY, Lau A, Orlando DA, Vakoc CR, et al. Selective inhibition of tumor oncogenes by disruption of super-enhancers. Cell. 2013;153(2):320-34.

17. Wang Y, Zhang T, Kwiatkowski N, Abraham BJ, Lee TI, Xie S, et al. CDK7-dependent transcriptional addiction in triple-negative breast cancer. Cell. 2015;163(1):174-86.

18. Zhang J, Liu W, Zou C, Zhao Z, Lai Y, Shi Z, et al. Targeting Super-Enhancer-Associated Oncogenes in Osteosarcoma with THZ2, a Covalent CDK7 Inhibitor. Clin Cancer Res. 2020;26(11):2681-92.

19. Xing R, Zhou Y, Yu J, Yu Y, Nie Y, Luo W, et al. Whole-genome sequencing reveals novel tandem-duplication hotspots and a prognostic mutational signature in gastric cancer. Nat Commun. 2019;10(1):2037.

20. Zou CY, Wang J, Shen JN, Huang G, Jin S, Yin JQ, et al. Establishment and characteristics of two syngeneic human osteosarcoma cell lines from primary tumor and skip metastases. Acta Pharmacol Sin. 2008;29(3):325-32.

21. Han J, Yong B, Luo C, Tan P, Peng T, Shen J. High serum alkaline phosphatase cooperating with MMP-9 predicts metastasis and poor prognosis in patients with primary osteosarcoma in Southern China. World J Surg Oncol. 2012;10:37.

22. Szulc P, Seeman E, Delmas PD. Biochemical measurements of bone turnover in children and adolescents. Osteoporos Int. 2000;11(4):281-94.

23. Camp RL, Dolled-Filhart M, Rimm DL. X-tile: a new bio-informatics tool for biomarker assessment and outcome-based cut-point optimization. Clin Cancer Res. 2004;10(21):7252-9.

24. Zhang T, Kwiatkowski N, Olson CM, Dixon-Clarke SE, Abraham BJ, Greifenberg AK, et al. Covalent targeting of remote cysteine residues to develop CDK12 and CDK13 inhibitors. Nat Chem Biol. 2016;12(10):876-84.

25. Jiang YY, Lin DC, Mayakonda A, Hazawa M, Ding LW, Chien WW, et al. Targeting super-enhancer-associated oncogenes in oesophageal squamous cell carcinoma. Gut. 2017;66(8):1358-68.

27. Aksnes LH, Bauer HC, Jebsen NL, Follerås G, Allert C, Haugen GS, et al. Limb-sparing surgery preserves more function than amputation: a Scandinavian sarcoma group study of 118 patients. J Bone Joint Surg Br. 2008;90(6):786-94.

28. Winkler K, Beron G, Kotz R, Salzer-Kuntschik M, Beck J, Beck W, et al. Neoadjuvant chemotherapy for osteogenic sarcoma: results of a Cooperative German/Austrian study. J Clin Oncol. 1984;2(6):617-24.

29. Polites SF, Heaton TE, LaQuaglia MP, Kim ES, Barry WE, Goodhue CJ, et al. Pneumonectomy for Pediatric Tumors-a Pediatric Surgical Oncology Research Collaborative Study. Ann Surg. 2021;274(6):e605-e9.

30. Bacci G, Ferrari S, Lari S, Mercuri M, Donati D, Longhi A, et al. Osteosarcoma of the limb. Amputation or limb salvage in patients treated by neoadjuvant chemotherapy. J Bone Joint Surg Br. 2002;84(1):88-92.

31. Zhao X, Wu Q, Gong X, Liu J, Ma Y. Osteosarcoma: a review of current and future therapeutic approaches. Biomed Eng Online. 2021;20(1):24.

32. Huang H, Hu J, Maryam A, Huang Q, Zhang Y, Ramakrishnan S, et al. Defining super-enhancer landscape in triple-negative breast cancer by multiomic profiling. Nat Commun. 2021;12(1):2242.

33. Wong M, Sun Y, Xi Z, Milazzo G, Poulos RC, Bartenhagen C, et al. JMJD6 is a tumorigenic factor and therapeutic target in neuroblastoma. Nat Commun. 2019;10(1):3319.

34. Zhang L, Xiong D, Liu Q, Luo Y, Tian Y, Xiao X, et al. Genome-Wide Histone H3K27 Acetylation Profiling Identified Genes Correlated With Prognosis in Papillary Thyroid Carcinoma. Front Cell Dev Biol. 2021;9:682561.

35. Fu Y, He G, Liu Z, Wang J, Zhang Z, Bao Q, et al. Exploration and Validation of a Novel Inflammatory Response-Associated Gene Signature to Predict Osteosarcoma Prognosis and Immune Infiltration. J Inflamm Res. 2021;14:6719-34.

36. Lei T, Qian H, Lei P, Hu Y. Ferroptosis-related gene signature associates with immunity and predicts prognosis accurately in patients with osteosarcoma. Cancer Sci. 2021;112(11):4785-98.

37. Ouyang Z, Li G, Zhu H, Wang J, Qi T, Qu Q, et al. Construction of a Five-Super-Enhancer-Associated-Genes Prognostic Model for Osteosarcoma Patients. Front Cell Dev Biol. 2020;8:598660.

38. Ferrari S, Bertoni F, Mercuri M, Picci P, Giacomini S, Longhi A, et al. Predictive factors of disease-free survival for non-metastatic osteosarcoma of the extremity: an analysis of 300 patients treated at the Rizzoli Institute. Ann Oncol. 2001;12(8):1145-50.

39. Bacci G, Longhi A, Ferrari S, Briccoli A, Donati D, De Paolis M, et al. Prognostic significance of serum lactate dehydrogenase in osteosarcoma of the extremity: experience at Rizzoli on 1421 patients treated over the last 30 years. Tumori. 2004;90(5):478-84.

40. Jia R, Li C, McCoy JP, Deng CX, Zheng ZM. SRp20 is a proto-oncogene critical for cell proliferation and tumor induction and maintenance. Int J Biol Sci. 2010;6(7):806-26.

41. Xue Y, Guo Y, Liu N, Meng X. MicroRNA-22-3p targeted regulating transcription factor 7-like 2 (TCF7L2) constrains the Wnt/β-catenin pathway and malignant behavior in osteosarcoma. Bioengineered. 2022;13(4):9135-47.

42. Li H, Han X, Yang S, Wang Y, Dong Y, Tang T. FOXP1 drives osteosarcoma development by repressing P21 and RB transcription downstream of P53. Oncogene. 2021;40(15):2785-802.

43. Xu L, Xia C, Sheng F, Sun Q, Xiong J, Wang S. CEP55 promotes the proliferation and invasion of tumour cells via the AKT signalling pathway in osteosarcoma. Carcinogenesis. 2018;39(4):623-31.

44. Regard JB, Scheek S, Borbiev T, Lanahan AA, Schneider A, Demetriades AM, et al. Verge: a novel vascular early response gene. J Neurosci. 2004;24(16):4092-103.

45. Smith TS, Southan C, Ellington K, Campbell D, Tew DG, Debouck C. Identification, genomic organization, and mRNA expression of LACTB, encoding a serine beta-lactamase-like protein with an amino-terminal transmembrane domain. Genomics. 2001;78(1-2):12-4.

1. Isakoff MS, Bielack SS, Meltzer P, Gorlick R. Osteosarcoma: Current Treatment and a Collaborative Pathway to Success. J Clin Oncol. 2015;33(27):3029-35.

2. Ferrari S, Smeland S, Mercuri M, Bertoni F, Longhi A, Ruggieri P, et al. Neoadjuvant chemotherapy with high-dose Ifosfamide, high-dose methotrexate, cisplatin, and doxorubicin for patients with localized osteosarcoma of the extremity: a joint study by the Italian and Scandinavian Sarcoma Groups. J Clin Oncol. 2005;23(34):8845-52.

3. Siegel RL, Miller KD, Jemal A. Cancer statistics, 2019. CA Cancer J Clin. 2019;69(1):7-34.

4. Ritter J, Bielack SS. Osteosarcoma. Ann Oncol. 2010;21 Suppl 7:vii320-5.

5. Gill J, Gorlick R. Advancing therapy for osteosarcoma. Nat Rev Clin Oncol. 2021;18(10):609-24.

6. Bielack SS, Kempf-Bielack B, Delling G, Exner GU, Flege S, Helmke K, et al. Prognostic factors in high-grade osteosarcoma of the extremities or trunk: an analysis of 1,702 patients treated on neoadjuvant cooperative osteosarcoma study group protocols. J Clin Oncol. 2002;20(3):776-90.

7. Han Z, Peng X, Yang Y, Yi J, Zhao D, Bao Q, et al. Integrated microfluidic-SERS for exosome biomarker profiling and osteosarcoma diagnosis. Biosens Bioelectron. 2022;217:114709.

8. Wu Y, Xu L, Yang P, Lin N, Huang X, Pan W, et al. Survival Prediction in High-grade Osteosarcoma Using Radiomics of Diagnostic Computed Tomography. EBioMedicine. 2018;34:27-34.

9. Chen W, Lin Y, Huang J, Yan Z, Cao H. A novel risk score model based on glycolysis-related genes and a prognostic model for predicting overall survival of osteosarcoma patients. J Orthop Res. 2022;40(10):2372-81.

10. Zhang M, Liu Y, Kong D. Identifying biomolecules and constructing a prognostic risk prediction model for recurrence in osteosarcoma. J Bone Oncol. 2021;26:100331.

11. Li L, Wang Y, He X, Li Z, Lu M, Gong T, et al. Hematological Prognostic Scoring System Can Predict Overall Survival and Can Indicate Response to Immunotherapy in Patients With Osteosarcoma. Front Immunol. 2022;13:879560.

12. Pelish HE, Liau BB, Nitulescu, II, Tangpeerachaikul A, Poss ZC, Da Silva DH, et al. Mediator kinase inhibition further activates super-enhancer-associated genes in AML. Nature. 2015;526(7572):273-6.

13. Whyte WA, Orlando DA, Hnisz D, Abraham BJ, Lin CY, Kagey MH, et al. Master transcription factors and mediator establish super-enhancers at key cell identity genes. Cell. 2013;153(2):307-19.

14. Tögel L, Nightingale R, Chueh AC, Jayachandran A, Tran H, Phesse T, et al. Dual Targeting of Bromodomain and Extraterminal Domain Proteins, and WNT or MAPK Signaling, Inhibits c-MYC Expression and Proliferation of Colorectal Cancer Cells. Mol Cancer Ther. 2016;15(6):1217-26.

15. Mansour MR, Abraham BJ, Anders L, Berezovskaya A, Gutierrez A, Durbin AD, et al. Oncogene regulation. An oncogenic super-enhancer formed through somatic mutation of a noncoding intergenic element. Science. 2014;346(6215):1373-7.

16. Lovén J, Hoke HA, Lin CY, Lau A, Orlando DA, Vakoc CR, et al. Selective inhibition of tumor oncogenes by disruption of super-enhancers. Cell. 2013;153(2):320-34.

17. Wang Y, Zhang T, Kwiatkowski N, Abraham BJ, Lee TI, Xie S, et al. CDK7-dependent transcriptional addiction in triple-negative breast cancer. Cell. 2015;163(1):174-86.

18. Zhang J, Liu W, Zou C, Zhao Z, Lai Y, Shi Z, et al. Targeting Super-Enhancer-Associated Oncogenes in Osteosarcoma with THZ2, a Covalent CDK7 Inhibitor. Clin Cancer Res. 2020;26(11):2681-92.

19. Xing R, Zhou Y, Yu J, Yu Y, Nie Y, Luo W, et al. Whole-genome sequencing reveals novel tandem-duplication hotspots and a prognostic mutational signature in gastric cancer. Nat Commun. 2019;10(1):2037.

20. Zou CY, Wang J, Shen JN, Huang G, Jin S, Yin JQ, et al. Establishment and characteristics of two syngeneic human osteosarcoma cell lines from primary tumor and skip metastases. Acta Pharmacol Sin. 2008;29(3):325-32.

21. Han J, Yong B, Luo C, Tan P, Peng T, Shen J. High serum alkaline phosphatase cooperating with MMP-9 predicts metastasis and poor prognosis in patients with primary osteosarcoma in Southern China. World J Surg Oncol. 2012;10:37.

22. Szulc P, Seeman E, Delmas PD. Biochemical measurements of bone turnover in children and adolescents. Osteoporos Int. 2000;11(4):281-94.

23. Camp RL, Dolled-Filhart M, Rimm DL. X-tile: a new bio-informatics tool for biomarker assessment and outcome-based cut-point optimization. Clin Cancer Res. 2004;10(21):7252-9.

24. Zhang T, Kwiatkowski N, Olson CM, Dixon-Clarke SE, Abraham BJ, Greifenberg AK, et al. Covalent targeting of remote cysteine residues to develop CDK12 and CDK13 inhibitors. Nat Chem Biol. 2016;12(10):876-84.

25. Jiang YY, Lin DC, Mayakonda A, Hazawa M, Ding LW, Chien WW, et al. Targeting super-enhancer-associated oncogenes in oesophageal squamous cell carcinoma. Gut. 2017;66(8):1358-68.

26. Meyers PA, Schwartz CL, Krailo M, Kleinerman ES, Betcher D, Bernstein ML, et al. Osteosarcoma: a randomized, prospective trial of the addition of ifosfamide and/or muramyl tripeptide to cisplatin, doxorubicin, and high-dose methotrexate. J Clin Oncol. 2005;23(9):2004-11.

27. Aksnes LH, Bauer HC, Jebsen NL, Follerås G, Allert C, Haugen GS, et al. Limb-sparing surgery preserves more function than amputation: a Scandinavian sarcoma group study of 118 patients. J Bone Joint Surg Br. 2008;90(6):786-94.

28. Winkler K, Beron G, Kotz R, Salzer-Kuntschik M, Beck J, Beck W, et al. Neoadjuvant chemotherapy for osteogenic sarcoma: results of a Cooperative German/Austrian study. J Clin Oncol. 1984;2(6):617-24.

29. Polites SF, Heaton TE, LaQuaglia MP, Kim ES, Barry WE, Goodhue CJ, et al. Pneumonectomy for Pediatric Tumors-a Pediatric Surgical Oncology Research Collaborative Study. Ann Surg. 2021;274(6):e605-e9.

30. Bacci G, Ferrari S, Lari S, Mercuri M, Donati D, Longhi A, et al. Osteosarcoma of the limb. Amputation or limb salvage in patients treated by neoadjuvant chemotherapy. J Bone Joint Surg Br. 2002;84(1):88-92.

31. Zhao X, Wu Q, Gong X, Liu J, Ma Y. Osteosarcoma: a review of current and future therapeutic approaches. Biomed Eng Online. 2021;20(1):24.

32. Huang H, Hu J, Maryam A, Huang Q, Zhang Y, Ramakrishnan S, et al. Defining super-enhancer landscape in triple-negative breast cancer by multiomic profiling. Nat Commun. 2021;12(1):2242.

33. Wong M, Sun Y, Xi Z, Milazzo G, Poulos RC, Bartenhagen C, et al. JMJD6 is a tumorigenic factor and therapeutic target in neuroblastoma. Nat Commun. 2019;10(1):3319.

34. Zhang L, Xiong D, Liu Q, Luo Y, Tian Y, Xiao X, et al. Genome-Wide Histone H3K27 Acetylation Profiling Identified Genes Correlated With Prognosis in Papillary Thyroid Carcinoma. Front Cell Dev Biol. 2021;9:682561.

35. Fu Y, He G, Liu Z, Wang J, Zhang Z, Bao Q, et al. Exploration and Validation of a Novel Inflammatory Response-Associated Gene Signature to Predict Osteosarcoma Prognosis and Immune Infiltration. J Inflamm Res. 2021;14:6719-34.

36. Lei T, Qian H, Lei P, Hu Y. Ferroptosis-related gene signature associates with immunity and predicts prognosis accurately in patients with osteosarcoma. Cancer Sci. 2021;112(11):4785-98.

37. Ouyang Z, Li G, Zhu H, Wang J, Qi T, Qu Q, et al. Construction of a Five-Super-Enhancer-Associated-Genes Prognostic Model for Osteosarcoma Patients. Front Cell Dev Biol. 2020;8:598660.

38. Ferrari S, Bertoni F, Mercuri M, Picci P, Giacomini S, Longhi A, et al. Predictive factors of disease-free survival for non-metastatic osteosarcoma of the extremity: an analysis of 300 patients treated at the Rizzoli Institute. Ann Oncol. 2001;12(8):1145-50.

39. Bacci G, Longhi A, Ferrari S, Briccoli A, Donati D, De Paolis M, et al. Prognostic significance of serum lactate dehydrogenase in osteosarcoma of the extremity: experience at Rizzoli on 1421 patients treated over the last 30 years. Tumori. 2004;90(5):478-84.

40. Jia R, Li C, McCoy JP, Deng CX, Zheng ZM. SRp20 is a proto-oncogene critical for cell proliferation and tumor induction and maintenance. Int J Biol Sci. 2010;6(7):806-26.

41. Xue Y, Guo Y, Liu N, Meng X. MicroRNA-22-3p targeted regulating transcription factor 7-like 2 (TCF7L2) constrains the Wnt/β-catenin pathway and malignant behavior in osteosarcoma. Bioengineered. 2022;13(4):9135-47.

42. Li H, Han X, Yang S, Wang Y, Dong Y, Tang T. FOXP1 drives osteosarcoma development by repressing P21 and RB transcription downstream of P53. Oncogene. 2021;40(15):2785-802.

43. Xu L, Xia C, Sheng F, Sun Q, Xiong J, Wang S. CEP55 promotes the proliferation and invasion of tumour cells via the AKT signalling pathway in osteosarcoma. Carcinogenesis. 2018;39(4):623-31.

44. Regard JB, Scheek S, Borbiev T, Lanahan AA, Schneider A, Demetriades AM, et al. Verge: a novel vascular early response gene. J Neurosci. 2004;24(16):4092-103.

45. Smith TS, Southan C, Ellington K, Campbell D, Tew DG, Debouck C. Identification, genomic organization, and mRNA expression of LACTB, encoding a serine beta-lactamase-like protein with an amino-terminal transmembrane domain. Genomics. 2001;78(1-2):12-4.

46. Keckesova Z, Donaher JL, De Cock J, Freinkman E, Lingrell S, Bachovchin DA, et al. LACTB is a tumour suppressor that modulates lipid metabolism and cell state. Nature. 2017;543(7647):681-6.

1. Isakoff MS, Bielack SS, Meltzer P, Gorlick R. Osteosarcoma: Current Treatment and a Collaborative Pathway to Success. J Clin Oncol. 2015;33(27):3029-35.

2. Ferrari S, Smeland S, Mercuri M, Bertoni F, Longhi A, Ruggieri P, et al. Neoadjuvant chemotherapy with high-dose Ifosfamide, high-dose methotrexate, cisplatin, and doxorubicin for patients with localized osteosarcoma of the extremity: a joint study by the Italian and Scandinavian Sarcoma Groups. J Clin Oncol. 2005;23(34):8845-52.

3. Siegel RL, Miller KD, Jemal A. Cancer statistics, 2019. CA Cancer J Clin. 2019;69(1):7-34.

4. Ritter J, Bielack SS. Osteosarcoma. Ann Oncol. 2010;21 Suppl 7:vii320-5.

5. Gill J, Gorlick R. Advancing therapy for osteosarcoma. Nat Rev Clin Oncol. 2021;18(10):609-24.

6. Bielack SS, Kempf-Bielack B, Delling G, Exner GU, Flege S, Helmke K, et al. Prognostic factors in high-grade osteosarcoma of the extremities or trunk: an analysis of 1,702 patients treated on neoadjuvant cooperative osteosarcoma study group protocols. J Clin Oncol. 2002;20(3):776-90.

7. Han Z, Peng X, Yang Y, Yi J, Zhao D, Bao Q, et al. Integrated microfluidic-SERS for exosome biomarker profiling and osteosarcoma diagnosis. Biosens Bioelectron. 2022;217:114709.

8. Wu Y, Xu L, Yang P, Lin N, Huang X, Pan W, et al. Survival Prediction in High-grade Osteosarcoma Using Radiomics of Diagnostic Computed Tomography. EBioMedicine. 2018;34:27-34.

9. Chen W, Lin Y, Huang J, Yan Z, Cao H. A novel risk score model based on glycolysis-related genes and a prognostic model for predicting overall survival of osteosarcoma patients. J Orthop Res. 2022;40(10):2372-81.

10. Zhang M, Liu Y, Kong D. Identifying biomolecules and constructing a prognostic risk prediction model for recurrence in osteosarcoma. J Bone Oncol. 2021;26:100331.

11. Li L, Wang Y, He X, Li Z, Lu M, Gong T, et al. Hematological Prognostic Scoring System Can Predict Overall Survival and Can Indicate Response to Immunotherapy in Patients With Osteosarcoma. Front Immunol. 2022;13:879560.

12. Pelish HE, Liau BB, Nitulescu, II, Tangpeerachaikul A, Poss ZC, Da Silva DH, et al. Mediator kinase inhibition further activates super-enhancer-associated genes in AML. Nature. 2015;526(7572):273-6.

13. Whyte WA, Orlando DA, Hnisz D, Abraham BJ, Lin CY, Kagey MH, et al. Master transcription factors and mediator establish super-enhancers at key cell identity genes. Cell. 2013;153(2):307-19.

14. Tögel L, Nightingale R, Chueh AC, Jayachandran A, Tran H, Phesse T, et al. Dual Targeting of Bromodomain and Extraterminal Domain Proteins, and WNT or MAPK Signaling, Inhibits c-MYC Expression and Proliferation of Colorectal Cancer Cells. Mol Cancer Ther. 2016;15(6):1217-26.

15. Mansour MR, Abraham BJ, Anders L, Berezovskaya A, Gutierrez A, Durbin AD, et al. Oncogene regulation. An oncogenic super-enhancer formed through somatic mutation of a noncoding intergenic element. Science. 2014;346(6215):1373-7.

16. Lovén J, Hoke HA, Lin CY, Lau A, Orlando DA, Vakoc CR, et al. Selective inhibition of tumor oncogenes by disruption of super-enhancers. Cell. 2013;153(2):320-34.

17. Wang Y, Zhang T, Kwiatkowski N, Abraham BJ, Lee TI, Xie S, et al. CDK7-dependent transcriptional addiction in triple-negative breast cancer. Cell. 2015;163(1):174-86.

18. Zhang J, Liu W, Zou C, Zhao Z, Lai Y, Shi Z, et al. Targeting Super-Enhancer-Associated Oncogenes in Osteosarcoma with THZ2, a Covalent CDK7 Inhibitor. Clin Cancer Res. 2020;26(11):2681-92.

19. Xing R, Zhou Y, Yu J, Yu Y, Nie Y, Luo W, et al. Whole-genome sequencing reveals novel tandem-duplication hotspots and a prognostic mutational signature in gastric cancer. Nat Commun. 2019;10(1):2037.

21. Han J, Yong B, Luo C, Tan P, Peng T, Shen J. High serum alkaline phosphatase cooperating with MMP-9 predicts metastasis and poor prognosis in patients with primary osteosarcoma in Southern China. World J Surg Oncol. 2012;10:37.

22. Szulc P, Seeman E, Delmas PD. Biochemical measurements of bone turnover in children and adolescents. Osteoporos Int. 2000;11(4):281-94.

23. Camp RL, Dolled-Filhart M, Rimm DL. X-tile: a new bio-informatics tool for biomarker assessment and outcome-based cut-point optimization. Clin Cancer Res. 2004;10(21):7252-9.

24. Zhang T, Kwiatkowski N, Olson CM, Dixon-Clarke SE, Abraham BJ, Greifenberg AK, et al. Covalent targeting of remote cysteine residues to develop CDK12 and CDK13 inhibitors. Nat Chem Biol. 2016;12(10):876-84.

25. Jiang YY, Lin DC, Mayakonda A, Hazawa M, Ding LW, Chien WW, et al. Targeting super-enhancer-associated oncogenes in oesophageal squamous cell carcinoma. Gut. 2017;66(8):1358-68.

26. Meyers PA, Schwartz CL, Krailo M, Kleinerman ES, Betcher D, Bernstein ML, et al. Osteosarcoma: a randomized, prospective trial of the addition of ifosfamide and/or muramyl tripeptide to cisplatin, doxorubicin, and high-dose methotrexate. J Clin Oncol. 2005;23(9):2004-11.

27. Aksnes LH, Bauer HC, Jebsen NL, Follerås G, Allert C, Haugen GS, et al. Limb-sparing surgery preserves more function than amputation: a Scandinavian sarcoma group study of 118 patients. J Bone Joint Surg Br. 2008;90(6):786-94.

28. Winkler K, Beron G, Kotz R, Salzer-Kuntschik M, Beck J, Beck W, et al. Neoadjuvant chemotherapy for osteogenic sarcoma: results of a Cooperative German/Austrian study. J Clin Oncol. 1984;2(6):617-24.

29. Polites SF, Heaton TE, LaQuaglia MP, Kim ES, Barry WE, Goodhue CJ, et al. Pneumonectomy for Pediatric Tumors-a Pediatric Surgical Oncology Research Collaborative Study. Ann Surg. 2021;274(6):e605-e9.

30. Bacci G, Ferrari S, Lari S, Mercuri M, Donati D, Longhi A, et al. Osteosarcoma of the limb. Amputation or limb salvage in patients treated by neoadjuvant chemotherapy. J Bone Joint Surg Br. 2002;84(1):88-92.

31. Zhao X, Wu Q, Gong X, Liu J, Ma Y. Osteosarcoma: a review of current and future therapeutic approaches. Biomed Eng Online. 2021;20(1):24.

32. Huang H, Hu J, Maryam A, Huang Q, Zhang Y, Ramakrishnan S, et al. Defining super-enhancer landscape in triple-negative breast cancer by multiomic profiling. Nat Commun. 2021;12(1):2242.

33. Wong M, Sun Y, Xi Z, Milazzo G, Poulos RC, Bartenhagen C, et al. JMJD6 is a tumorigenic factor and therapeutic target in neuroblastoma. Nat Commun. 2019;10(1):3319.

34. Zhang L, Xiong D, Liu Q, Luo Y, Tian Y, Xiao X, et al. Genome-Wide Histone H3K27 Acetylation Profiling Identified Genes Correlated With Prognosis in Papillary Thyroid Carcinoma. Front Cell Dev Biol. 2021;9:682561.

35. Fu Y, He G, Liu Z, Wang J, Zhang Z, Bao Q, et al. Exploration and Validation of a Novel Inflammatory Response-Associated Gene Signature to Predict Osteosarcoma Prognosis and Immune Infiltration. J Inflamm Res. 2021;14:6719-34.

36. Lei T, Qian H, Lei P, Hu Y. Ferroptosis-related gene signature associates with immunity and predicts prognosis accurately in patients with osteosarcoma. Cancer Sci. 2021;112(11):4785-98.

37. Ouyang Z, Li G, Zhu H, Wang J, Qi T, Qu Q, et al. Construction of a Five-Super-Enhancer-Associated-Genes Prognostic Model for Osteosarcoma Patients. Front Cell Dev Biol. 2020;8:598660.

38. Ferrari S, Bertoni F, Mercuri M, Picci P, Giacomini S, Longhi A, et al. Predictive factors of disease-free survival for non-metastatic osteosarcoma of the extremity: an analysis of 300 patients treated at the Rizzoli Institute. Ann Oncol. 2001;12(8):1145-50.

39. Bacci G, Longhi A, Ferrari S, Briccoli A, Donati D, De Paolis M, et al. Prognostic significance of serum lactate dehydrogenase in osteosarcoma of the extremity: experience at Rizzoli on 1421 patients treated over the last 30 years. Tumori. 2004;90(5):478-84.

40. Jia R, Li C, McCoy JP, Deng CX, Zheng ZM. SRp20 is a proto-oncogene critical for cell proliferation and tumor induction and maintenance. Int J Biol Sci. 2010;6(7):806-26.

41. Xue Y, Guo Y, Liu N, Meng X. MicroRNA-22-3p targeted regulating transcription factor 7-like 2 (TCF7L2) constrains the Wnt/β-catenin pathway and malignant behavior in osteosarcoma. Bioengineered. 2022;13(4):9135-47.

42. Li H, Han X, Yang S, Wang Y, Dong Y, Tang T. FOXP1 drives osteosarcoma development by repressing P21 and RB transcription downstream of P53. Oncogene. 2021;40(15):2785-802.

43. Xu L, Xia C, Sheng F, Sun Q, Xiong J, Wang S. CEP55 promotes the proliferation and invasion of tumour cells via the AKT signalling pathway in osteosarcoma. Carcinogenesis. 2018;39(4):623-31.

44. Regard JB, Scheek S, Borbiev T, Lanahan AA, Schneider A, Demetriades AM, et al. Verge: a novel vascular early response gene. J Neurosci. 2004;24(16):4092-103.

45. Smith TS, Southan C, Ellington K, Campbell D, Tew DG, Debouck C. Identification, genomic organization, and mRNA expression of LACTB, encoding a serine beta-lactamase-like protein with an amino-terminal transmembrane domain. Genomics. 2001;78(1-2):12-4.

46. Keckesova Z, Donaher JL, De Cock J, Freinkman E, Lingrell S, Bachovchin DA, et al. LACTB is a tumour suppressor that modulates lipid metabolism and cell state. Nature. 2017;543(7647):681-6.

47. Zeng K, Chen X, Hu X, Liu X, Xu T, Sun H, et al. LACTB, a novel epigenetic silenced tumor suppressor, inhibits colorectal cancer progression by attenuating MDM2-mediated p53 ubiquitination and degradation. Oncogene. 2018;37(41):5534-51.

1. Isakoff MS, Bielack SS, Meltzer P, Gorlick R. Osteosarcoma: Current Treatment and a Collaborative Pathway to Success. J Clin Oncol. 2015;33(27):3029-35.

2. Ferrari S, Smeland S, Mercuri M, Bertoni F, Longhi A, Ruggieri P, et al. Neoadjuvant chemotherapy with high-dose Ifosfamide, high-dose methotrexate, cisplatin, and doxorubicin for patients with localized osteosarcoma of the extremity: a joint study by the Italian and Scandinavian Sarcoma Groups. J Clin Oncol. 2005;23(34):8845-52.

3. Siegel RL, Miller KD, Jemal A. Cancer statistics, 2019. CA Cancer J Clin. 2019;69(1):7-34.

4. Ritter J, Bielack SS. Osteosarcoma. Ann Oncol. 2010;21 Suppl 7:vii320-5.

5. Gill J, Gorlick R. Advancing therapy for osteosarcoma. Nat Rev Clin Oncol. 2021;18(10):609-24.

6. Bielack SS, Kempf-Bielack B, Delling G, Exner GU, Flege S, Helmke K, et al. Prognostic factors in high-grade osteosarcoma of the extremities or trunk: an analysis of 1,702 patients treated on neoadjuvant cooperative osteosarcoma study group protocols. J Clin Oncol. 2002;20(3):776-90.

7. Han Z, Peng X, Yang Y, Yi J, Zhao D, Bao Q, et al. Integrated microfluidic-SERS for exosome biomarker profiling and osteosarcoma diagnosis. Biosens Bioelectron. 2022;217:114709.

8. Wu Y, Xu L, Yang P, Lin N, Huang X, Pan W, et al. Survival Prediction in High-grade Osteosarcoma Using Radiomics of Diagnostic Computed Tomography. EBioMedicine. 2018;34:27-34.

9. Chen W, Lin Y, Huang J, Yan Z, Cao H. A novel risk score model based on glycolysis-related genes and a prognostic model for predicting overall survival of osteosarcoma patients. J Orthop Res. 2022;40(10):2372-81.

10. Zhang M, Liu Y, Kong D. Identifying biomolecules and constructing a prognostic risk prediction model for recurrence in osteosarcoma. J Bone Oncol. 2021;26:100331.

11. Li L, Wang Y, He X, Li Z, Lu M, Gong T, et al. Hematological Prognostic Scoring System Can Predict Overall Survival and Can Indicate Response to Immunotherapy in Patients With Osteosarcoma. Front Immunol. 2022;13:879560.

12. Pelish HE, Liau BB, Nitulescu, II, Tangpeerachaikul A, Poss ZC, Da Silva DH, et al. Mediator kinase inhibition further activates super-enhancer-associated genes in AML. Nature. 2015;526(7572):273-6.

13. Whyte WA, Orlando DA, Hnisz D, Abraham BJ, Lin CY, Kagey MH, et al. Master transcription factors and mediator establish super-enhancers at key cell identity genes. Cell. 2013;153(2):307-19.

14. Tögel L, Nightingale R, Chueh AC, Jayachandran A, Tran H, Phesse T, et al. Dual Targeting of Bromodomain and Extraterminal Domain Proteins, and WNT or MAPK Signaling, Inhibits c-MYC Expression and Proliferation of Colorectal Cancer Cells. Mol Cancer Ther. 2016;15(6):1217-26.

15. Mansour MR, Abraham BJ, Anders L, Berezovskaya A, Gutierrez A, Durbin AD, et al. Oncogene regulation. An oncogenic super-enhancer formed through somatic mutation of a noncoding intergenic element. Science. 2014;346(6215):1373-7.

16. Lovén J, Hoke HA, Lin CY, Lau A, Orlando DA, Vakoc CR, et al. Selective inhibition of tumor oncogenes by disruption of super-enhancers. Cell. 2013;153(2):320-34.

17. Wang Y, Zhang T, Kwiatkowski N, Abraham BJ, Lee TI, Xie S, et al. CDK7-dependent transcriptional addiction in triple-negative breast cancer. Cell. 2015;163(1):174-86.

18. Zhang J, Liu W, Zou C, Zhao Z, Lai Y, Shi Z, et al. Targeting Super-Enhancer-Associated Oncogenes in Osteosarcoma with THZ2, a Covalent CDK7 Inhibitor. Clin Cancer Res. 2020;26(11):2681-92.

19. Xing R, Zhou Y, Yu J, Yu Y, Nie Y, Luo W, et al. Whole-genome sequencing reveals novel tandem-duplication hotspots and a prognostic mutational signature in gastric cancer. Nat Commun. 2019;10(1):2037.

20. Zou CY, Wang J, Shen JN, Huang G, Jin S, Yin JQ, et al. Establishment and characteristics of two syngeneic human osteosarcoma cell lines from primary tumor and skip metastases. Acta Pharmacol Sin. 2008;29(3):325-32.

21. Han J, Yong B, Luo C, Tan P, Peng T, Shen J. High serum alkaline phosphatase cooperating with MMP-9 predicts metastasis and poor prognosis in patients with primary osteosarcoma in Southern China. World J Surg Oncol. 2012;10:37.

22. Szulc P, Seeman E, Delmas PD. Biochemical measurements of bone turnover in children and adolescents. Osteoporos Int. 2000;11(4):281-94.

23. Camp RL, Dolled-Filhart M, Rimm DL. X-tile: a new bio-informatics tool for biomarker assessment and outcome-based cut-point optimization. Clin Cancer Res. 2004;10(21):7252-9.

24. Zhang T, Kwiatkowski N, Olson CM, Dixon-Clarke SE, Abraham BJ, Greifenberg AK, et al. Covalent targeting of remote cysteine residues to develop CDK12 and CDK13 inhibitors. Nat Chem Biol. 2016;12(10):876-84.

25. Jiang YY, Lin DC, Mayakonda A, Hazawa M, Ding LW, Chien WW, et al. Targeting super-enhancer-associated oncogenes in oesophageal squamous cell carcinoma. Gut. 2017;66(8):1358-68.

26. Meyers PA, Schwartz CL, Krailo M, Kleinerman ES, Betcher D, Bernstein ML, et al. Osteosarcoma: a randomized, prospective trial of the addition of ifosfamide and/or muramyl tripeptide to cisplatin, doxorubicin, and high-dose methotrexate. J Clin Oncol. 2005;23(9):2004-11.

27. Aksnes LH, Bauer HC, Jebsen NL, Follerås G, Allert C, Haugen GS, et al. Limb-sparing surgery preserves more function than amputation: a Scandinavian sarcoma group study of 118 patients. J Bone Joint Surg Br. 2008;90(6):786-94.

28. Winkler K, Beron G, Kotz R, Salzer-Kuntschik M, Beck J, Beck W, et al. Neoadjuvant chemotherapy for osteogenic sarcoma: results of a Cooperative German/Austrian study. J Clin Oncol. 1984;2(6):617-24.

29. Polites SF, Heaton TE, LaQuaglia MP, Kim ES, Barry WE, Goodhue CJ, et al. Pneumonectomy for Pediatric Tumors-a Pediatric Surgical Oncology Research Collaborative Study. Ann Surg. 2021;274(6):e605-e9.

30. Bacci G, Ferrari S, Lari S, Mercuri M, Donati D, Longhi A, et al. Osteosarcoma of the limb. Amputation or limb salvage in patients treated by neoadjuvant chemotherapy. J Bone Joint Surg Br. 2002;84(1):88-92.

31. Zhao X, Wu Q, Gong X, Liu J, Ma Y. Osteosarcoma: a review of current and future therapeutic approaches. Biomed Eng Online. 2021;20(1):24.

32. Huang H, Hu J, Maryam A, Huang Q, Zhang Y, Ramakrishnan S, et al. Defining super-enhancer landscape in triple-negative breast cancer by multiomic profiling. Nat Commun. 2021;12(1):2242.

33. Wong M, Sun Y, Xi Z, Milazzo G, Poulos RC, Bartenhagen C, et al. JMJD6 is a tumorigenic factor and therapeutic target in neuroblastoma. Nat Commun. 2019;10(1):3319.

34. Zhang L, Xiong D, Liu Q, Luo Y, Tian Y, Xiao X, et al. Genome-Wide Histone H3K27 Acetylation Profiling Identified Genes Correlated With Prognosis in Papillary Thyroid Carcinoma. Front Cell Dev Biol. 2021;9:682561.

35. Fu Y, He G, Liu Z, Wang J, Zhang Z, Bao Q, et al. Exploration and Validation of a Novel Inflammatory Response-Associated Gene Signature to Predict Osteosarcoma Prognosis and Immune Infiltration. J Inflamm Res. 2021;14:6719-34.

36. Lei T, Qian H, Lei P, Hu Y. Ferroptosis-related gene signature associates with immunity and predicts prognosis accurately in patients with osteosarcoma. Cancer Sci. 2021;112(11):4785-98.

37. Ouyang Z, Li G, Zhu H, Wang J, Qi T, Qu Q, et al. Construction of a Five-Super-Enhancer-Associated-Genes Prognostic Model for Osteosarcoma Patients. Front Cell Dev Biol. 2020;8:598660.

38. Ferrari S, Bertoni F, Mercuri M, Picci P, Giacomini S, Longhi A, et al. Predictive factors of disease-free survival for non-metastatic osteosarcoma of the extremity: an analysis of 300 patients treated at the Rizzoli Institute. Ann Oncol. 2001;12(8):1145-50.

39. Bacci G, Longhi A, Ferrari S, Briccoli A, Donati D, De Paolis M, et al. Prognostic significance of serum lactate dehydrogenase in osteosarcoma of the extremity: experience at Rizzoli on 1421 patients treated over the last 30 years. Tumori. 2004;90(5):478-84.

40. Jia R, Li C, McCoy JP, Deng CX, Zheng ZM. SRp20 is a proto-oncogene critical for cell proliferation and tumor induction and maintenance. Int J Biol Sci. 2010;6(7):806-26.

42. Li H, Han X, Yang S, Wang Y, Dong Y, Tang T. FOXP1 drives osteosarcoma development by repressing P21 and RB transcription downstream of P53. Oncogene. 2021;40(15):2785-802.

43. Xu L, Xia C, Sheng F, Sun Q, Xiong J, Wang S. CEP55 promotes the proliferation and invasion of tumour cells via the AKT signalling pathway in osteosarcoma. Carcinogenesis. 2018;39(4):623-31.

44. Regard JB, Scheek S, Borbiev T, Lanahan AA, Schneider A, Demetriades AM, et al. Verge: a novel vascular early response gene. J Neurosci. 2004;24(16):4092-103.

45. Smith TS, Southan C, Ellington K, Campbell D, Tew DG, Debouck C. Identification, genomic organization, and mRNA expression of LACTB, encoding a serine beta-lactamase-like protein with an amino-terminal transmembrane domain. Genomics. 2001;78(1-2):12-4.

46. Keckesova Z, Donaher JL, De Cock J, Freinkman E, Lingrell S, Bachovchin DA, et al. LACTB is a tumour suppressor that modulates lipid metabolism and cell state. Nature. 2017;543(7647):681-6.

47. Zeng K, Chen X, Hu X, Liu X, Xu T, Sun H, et al. LACTB, a novel epigenetic silenced tumor suppressor, inhibits colorectal cancer progression by attenuating MDM2-mediated p53 ubiquitination and degradation. Oncogene. 2018;37(41):5534-51.

48. Peng LX, Wang MD, Xie P, Yang JP, Sun R, Zheng LS, et al. LACTB promotes metastasis of nasopharyngeal carcinoma via activation of ERBB3/EGFR-ERK signaling resulting in unfavorable patient survival. Cancer Lett. 2021;498:165-77.

1. Isakoff MS, Bielack SS, Meltzer P, Gorlick R. Osteosarcoma: Current Treatment and a Collaborative Pathway to Success. J Clin Oncol. 2015;33(27):3029-35.

2. Ferrari S, Smeland S, Mercuri M, Bertoni F, Longhi A, Ruggieri P, et al. Neoadjuvant chemotherapy with high-dose Ifosfamide, high-dose methotrexate, cisplatin, and doxorubicin for patients with localized osteosarcoma of the extremity: a joint study by the Italian and Scandinavian Sarcoma Groups. J Clin Oncol. 2005;23(34):8845-52.

3. Siegel RL, Miller KD, Jemal A. Cancer statistics, 2019. CA Cancer J Clin. 2019;69(1):7-34.

4. Ritter J, Bielack SS. Osteosarcoma. Ann Oncol. 2010;21 Suppl 7:vii320-5.

5. Gill J, Gorlick R. Advancing therapy for osteosarcoma. Nat Rev Clin Oncol. 2021;18(10):609-24.

6. Bielack SS, Kempf-Bielack B, Delling G, Exner GU, Flege S, Helmke K, et al. Prognostic factors in high-grade osteosarcoma of the extremities or trunk: an analysis of 1,702 patients treated on neoadjuvant cooperative osteosarcoma study group protocols. J Clin Oncol. 2002;20(3):776-90.

7. Han Z, Peng X, Yang Y, Yi J, Zhao D, Bao Q, et al. Integrated microfluidic-SERS for exosome biomarker profiling and osteosarcoma diagnosis. Biosens Bioelectron. 2022;217:114709.

8. Wu Y, Xu L, Yang P, Lin N, Huang X, Pan W, et al. Survival Prediction in High-grade Osteosarcoma Using Radiomics of Diagnostic Computed Tomography. EBioMedicine. 2018;34:27-34.

9. Chen W, Lin Y, Huang J, Yan Z, Cao H. A novel risk score model based on glycolysis-related genes and a prognostic model for predicting overall survival of osteosarcoma patients. J Orthop Res. 2022;40(10):2372-81.

10. Zhang M, Liu Y, Kong D. Identifying biomolecules and constructing a prognostic risk prediction model for recurrence in osteosarcoma. J Bone Oncol. 2021;26:100331.

11. Li L, Wang Y, He X, Li Z, Lu M, Gong T, et al. Hematological Prognostic Scoring System Can Predict Overall Survival and Can Indicate Response to Immunotherapy in Patients With Osteosarcoma. Front Immunol. 2022;13:879560.

12. Pelish HE, Liau BB, Nitulescu, II, Tangpeerachaikul A, Poss ZC, Da Silva DH, et al. Mediator kinase inhibition further activates super-enhancer-associated genes in AML. Nature. 2015;526(7572):273-6.

14. Tögel L, Nightingale R, Chueh AC, Jayachandran A, Tran H, Phesse T, et al. Dual Targeting of Bromodomain and Extraterminal Domain Proteins, and WNT or MAPK Signaling, Inhibits c-MYC Expression and Proliferation of Colorectal Cancer Cells. Mol Cancer Ther. 2016;15(6):1217-26.

15. Mansour MR, Abraham BJ, Anders L, Berezovskaya A, Gutierrez A, Durbin AD, et al. Oncogene regulation. An oncogenic super-enhancer formed through somatic mutation of a noncoding intergenic element. Science. 2014;346(6215):1373-7.

16. Lovén J, Hoke HA, Lin CY, Lau A, Orlando DA, Vakoc CR, et al. Selective inhibition of tumor oncogenes by disruption of super-enhancers. Cell. 2013;153(2):320-34.

17. Wang Y, Zhang T, Kwiatkowski N, Abraham BJ, Lee TI, Xie S, et al. CDK7-dependent transcriptional addiction in triple-negative breast cancer. Cell. 2015;163(1):174-86.

18. Zhang J, Liu W, Zou C, Zhao Z, Lai Y, Shi Z, et al. Targeting Super-Enhancer-Associated Oncogenes in Osteosarcoma with THZ2, a Covalent CDK7 Inhibitor. Clin Cancer Res. 2020;26(11):2681-92.

13. Whyte WA, Orlando DA, Hnisz D, Abraham BJ, Lin CY, Kagey MH, et al. Master transcription factors and mediator establish super-enhancers at key cell identity genes. Cell. 2013;153(2):307-19.

19. Xing R, Zhou Y, Yu J, Yu Y, Nie Y, Luo W, et al. Whole-genome sequencing reveals novel tandem-duplication hotspots and a prognostic mutational signature in gastric cancer. Nat Commun. 2019;10(1):2037.

20. Zou CY, Wang J, Shen JN, Huang G, Jin S, Yin JQ, et al. Establishment and characteristics of two syngeneic human osteosarcoma cell lines from primary tumor and skip metastases. Acta Pharmacol Sin. 2008;29(3):325-32.

21. Han J, Yong B, Luo C, Tan P, Peng T, Shen J. High serum alkaline phosphatase cooperating with MMP-9 predicts metastasis and poor prognosis in patients with primary osteosarcoma in Southern China. World J Surg Oncol. 2012;10:37.

22. Szulc P, Seeman E, Delmas PD. Biochemical measurements of bone turnover in children and adolescents. Osteoporos Int. 2000;11(4):281-94.

23. Camp RL, Dolled-Filhart M, Rimm DL. X-tile: a new bio-informatics tool for biomarker assessment and outcome-based cut-point optimization. Clin Cancer Res. 2004;10(21):7252-9.

24. Zhang T, Kwiatkowski N, Olson CM, Dixon-Clarke SE, Abraham BJ, Greifenberg AK, et al. Covalent targeting of remote cysteine residues to develop CDK12 and CDK13 inhibitors. Nat Chem Biol. 2016;12(10):876-84.

25. Jiang YY, Lin DC, Mayakonda A, Hazawa M, Ding LW, Chien WW, et al. Targeting super-enhancer-associated oncogenes in oesophageal squamous cell carcinoma. Gut. 2017;66(8):1358-68.

26. Meyers PA, Schwartz CL, Krailo M, Kleinerman ES, Betcher D, Bernstein ML, et al. Osteosarcoma: a randomized, prospective trial of the addition of ifosfamide and/or muramyl tripeptide to cisplatin, doxorubicin, and high-dose methotrexate. J Clin Oncol. 2005;23(9):2004-11.

27. Aksnes LH, Bauer HC, Jebsen NL, Follerås G, Allert C, Haugen GS, et al. Limb-sparing surgery preserves more function than amputation: a Scandinavian sarcoma group study of 118 patients. J Bone Joint Surg Br. 2008;90(6):786-94.

28. Winkler K, Beron G, Kotz R, Salzer-Kuntschik M, Beck J, Beck W, et al. Neoadjuvant chemotherapy for osteogenic sarcoma: results of a Cooperative German/Austrian study. J Clin Oncol. 1984;2(6):617-24.

29. Polites SF, Heaton TE, LaQuaglia MP, Kim ES, Barry WE, Goodhue CJ, et al. Pneumonectomy for Pediatric Tumors-a Pediatric Surgical Oncology Research Collaborative Study. Ann Surg. 2021;274(6):e605-e9.

30. Bacci G, Ferrari S, Lari S, Mercuri M, Donati D, Longhi A, et al. Osteosarcoma of the limb. Amputation or limb salvage in patients treated by neoadjuvant chemotherapy. J Bone Joint Surg Br. 2002;84(1):88-92.

31. Zhao X, Wu Q, Gong X, Liu J, Ma Y. Osteosarcoma: a review of current and future therapeutic approaches. Biomed Eng Online. 2021;20(1):24.

32. Huang H, Hu J, Maryam A, Huang Q, Zhang Y, Ramakrishnan S, et al. Defining super-enhancer landscape in triple-negative breast cancer by multiomic profiling. Nat Commun. 2021;12(1):2242.

33. Wong M, Sun Y, Xi Z, Milazzo G, Poulos RC, Bartenhagen C, et al. JMJD6 is a tumorigenic factor and therapeutic target in neuroblastoma. Nat Commun. 2019;10(1):3319.

34. Zhang L, Xiong D, Liu Q, Luo Y, Tian Y, Xiao X, et al. Genome-Wide Histone H3K27 Acetylation Profiling Identified Genes Correlated With Prognosis in Papillary Thyroid Carcinoma. Front Cell Dev Biol. 2021;9:682561.

35. Fu Y, He G, Liu Z, Wang J, Zhang Z, Bao Q, et al. Exploration and Validation of a Novel Inflammatory Response-Associated Gene Signature to Predict Osteosarcoma Prognosis and Immune Infiltration. J Inflamm Res. 2021;14:6719-34.

36. Lei T, Qian H, Lei P, Hu Y. Ferroptosis-related gene signature associates with immunity and predicts prognosis accurately in patients with osteosarcoma. Cancer Sci. 2021;112(11):4785-98.

37. Ouyang Z, Li G, Zhu H, Wang J, Qi T, Qu Q, et al. Construction of a Five-Super-Enhancer-Associated-Genes Prognostic Model for Osteosarcoma Patients. Front Cell Dev Biol. 2020;8:598660.

38. Ferrari S, Bertoni F, Mercuri M, Picci P, Giacomini S, Longhi A, et al. Predictive factors of disease-free survival for non-metastatic osteosarcoma of the extremity: an analysis of 300 patients treated at the Rizzoli Institute. Ann Oncol. 2001;12(8):1145-50.

39. Bacci G, Longhi A, Ferrari S, Briccoli A, Donati D, De Paolis M, et al. Prognostic significance of serum lactate dehydrogenase in osteosarcoma of the extremity: experience at Rizzoli on 1421 patients treated over the last 30 years. Tumori. 2004;90(5):478-84.

40. Jia R, Li C, McCoy JP, Deng CX, Zheng ZM. SRp20 is a proto-oncogene critical for cell proliferation and tumor induction and maintenance. Int J Biol Sci. 2010;6(7):806-26.

41. Xue Y, Guo Y, Liu N, Meng X. MicroRNA-22-3p targeted regulating transcription factor 7-like 2 (TCF7L2) constrains the Wnt/β-catenin pathway and malignant behavior in osteosarcoma. Bioengineered. 2022;13(4):9135-47.

42. Li H, Han X, Yang S, Wang Y, Dong Y, Tang T. FOXP1 drives osteosarcoma development by repressing P21 and RB transcription downstream of P53. Oncogene. 2021;40(15):2785-802.

43. Xu L, Xia C, Sheng F, Sun Q, Xiong J, Wang S. CEP55 promotes the proliferation and invasion of tumour cells via the AKT signalling pathway in osteosarcoma. Carcinogenesis. 2018;39(4):623-31.

44. Regard JB, Scheek S, Borbiev T, Lanahan AA, Schneider A, Demetriades AM, et al. Verge: a novel vascular early response gene. J Neurosci. 2004;24(16):4092-103.

45. Smith TS, Southan C, Ellington K, Campbell D, Tew DG, Debouck C. Identification, genomic organization, and mRNA expression of LACTB, encoding a serine beta-lactamase-like protein with an amino-terminal transmembrane domain. Genomics. 2001;78(1-2):12-4.

46. Keckesova Z, Donaher JL, De Cock J, Freinkman E, Lingrell S, Bachovchin DA, et al. LACTB is a tumour suppressor that modulates lipid metabolism and cell state. Nature. 2017;543(7647):681-6.

47. Zeng K, Chen X, Hu X, Liu X, Xu T, Sun H, et al. LACTB, a novel epigenetic silenced tumor suppressor, inhibits colorectal cancer progression by attenuating MDM2-mediated p53 ubiquitination and degradation. Oncogene. 2018;37(41):5534-51.

48. Peng LX, Wang MD, Xie P, Yang JP, Sun R, Zheng LS, et al. LACTB promotes metastasis of nasopharyngeal carcinoma via activation of ERBB3/EGFR-ERK signaling resulting in unfavorable patient survival. Cancer Lett. 2021;498:165-77.

49. Xie J, Peng Y, Chen X, Li Q, Jian B, Wen Z, et al. LACTB mRNA expression is increased in pancreatic adenocarcinoma and high expression indicates a poor prognosis. PLoS One. 2021;16(1):e0245908.
